# Psychomotor Slowing in Psychosis and Inhibitory Repetitive Transcranial Magnetic Stimulation: A Randomized Clinical Trial

**DOI:** 10.1001/jamapsychiatry.2024.0026

**Published:** 2024-02-28

**Authors:** Sebastian Walther, Danai Alexaki, Florian Weiss, Daniel Baumann-Gama, Alexandra Kyrou, Melanie G. Nuoffer, Florian Wüthrich, Stephanie Lefebvre, Niluja Nadesalingam

**Affiliations:** 1 Translational Research Center, University Hospital of Psychiatry and Psychotherapy, University of Bern, Bern, Switzerland; 2 Graduate School for Health Sciences, University of Bern, Bern, Switzerland

## Abstract

**Importance:**

Psychomotor slowing is a frequent symptom of psychosis, impairing gross and fine motor behavior. It is associated with poor outcomes and functioning, and no treatment is available.

**Objective:**

To investigate whether 15 sessions of inhibitory repetitive transcranial magnetic stimulation (rTMS) may reduce psychomotor slowing.

**Design, Setting, and Participants:**

This was a 4-arm, double-blind, randomized, sham-controlled trial at a university hospital in Switzerland. Enrollment took place from March 2019 to August 2022. Adults aged 18 to 60 years with schizophrenia spectrum disorders and severe psychomotor slowing were eligible. All patients continued existing medications, including antipsychotics and benzodiazepines. Those with substance misuse (other than nicotine), conditions associated with impaired or aberrant movement, convulsions, history of hearing problems, other conditions typically excluded from magnetic resonance imaging or TMS, any TMS treatment in the past 3 months, or those who were pregnant or breastfeeding were excluded. Of 615 patients screened for eligibility, 103 were randomized and 88 received at least 1 session of rTMS: 22 were assigned to 1-Hz rTMS, 22 to iTBS, 22 to sham, and 22 to the waiting group. Follow-up was conducted at 6 weeks and 24 weeks following the week 3 assessments including clinical, functional, and motor measures.

**Interventions:**

Fifteen sessions of rTMS in 3 weeks over the supplementary motor area: 1-Hz rTMS, iTBS, sham, or no treatment (waiting). After 3 weeks, the waiting group received 15 sessions of 1-Hz rTMS over the supplementary motor area.

**Main Outcomes and Measures:**

The main outcome was the proportion of responders at week 3 in the Salpêtrière Retardation Rating Scale (SRRS) defined as a 30% or greater reduction from baseline (last-observation-carried-forward). The SRRS has 15 items and a maximum total score of 60.

**Results:**

Of the 88 participants analyzed, 45 were men and 43 were women. The mean (SD) age was 36.3 (12.4) years and the mean (SD) SRRS score was 24.0 (5.9). A total of 69 participants completed the study. At week 3, response rates differed between groups: 15 of 22 (68%) in the 1-Hz rTMS group, 8 of 22 (36%) in the iTBS group, 7 of 22 (32%) in the sham group, and 4 of 22 (18%) in the waiting group (χ^2^_3_ = 12.1; *P* = .007). The 1-Hz rTMS group had more responders than sham (odds ratio [OR], 0.13; 95% CI, 0.02-0.65; *P* = .03), iTBS (OR, 0.12; 95% CI, 0.02-0.61; *P* = .02), and waiting (OR, 0.04; 95% CI, 0.01-0.22; *P* = .003). In the waiting group, 10 of 16 participants (63%) responded after receiving 15 sessions of 1-Hz rTMS. No serious adverse events occurred.

**Conclusions and Relevance:**

In this study, inhibitory add-on rTMS safely alleviated psychomotor slowing in psychosis compared with iTBS, sham, and no treatment. The treatment was also effective with delayed onset. Future studies need to explore the neural changes associated with supplementary motor area rTMS in psychosis.

**Trial Registration:**

ClinicalTrials.gov Identifier: NCT03921450

## Key Points

**Question:**

Can inhibitory transcranial magnetic stimulation ameliorate psychomotor slowing in psychosis?

**Findings:**

In this 4-arm randomized clinical trial including 88 patients, 15 sessions of 1-Hz repetitive transcranial magnetic stimulation (rTMS) on the supplementary motor area led to response in significantly more patients than intermittent theta burst stimulation (iTBS), sham, or no treatment. Most of the patients in the waiting group responded to delayed-onset 1-Hz rTMS.

**Meaning:**

The findings indicate that add-on inhibitory rTMS may be an effective treatment for patients with psychosis and psychomotor slowing; further studies are needed to assess neural changes associated with this treatment.

## Teaser

This randomized clinical trial compares repetitive transcranial magnetic stimulation with intermittent theta burst stimulation, sham, and no treatment for reducing psychomotor slowing in individuals with psychosis.

## Introduction

Schizophrenia is a severe mental disorder affecting 1% of the population and leading to adverse outcomes and poor quality of life.^1^ Core schizophrenia symptom dimensions include hallucinations, delusions, disorganized speech, negative symptoms, impaired cognition, and abnormal psychomotor behavior.^2^ Motor abnormalities have been reported across all stages of schizophrenia spectrum disorders, including untreated patients experiencing a first episode, and indicate poor clinical and functional outcomes.^3,4,5,6^ One of these motor abnormalities is psychomotor slowing that impairs both fine and gross motor behavior, facial expression, and speech. Psychomotor slowing impacts movement initiation, quantity, and velocity; furthermore, it is associated with lower cognitive processing speed.^7,8^ Hypokinetic catatonia episodes are considered an extreme form of psychomotor slowing.^9,10^ Psychomotor slowing often comes with multiple disadvantages, such as cognitive impairment, sedentary behavior, cardiometabolic risks, poor quality of life, lower subjective well-being, and impaired functioning.^7,11,12,13,14^ No specific treatment is available to target psychomotor slowing.

Psychomotor slowing in schizophrenia can be captured using clinical rating scales and instrumental measures, such as wrist actigraphy, gait analysis, or fine motor tasks.^15,16^ Multiple neural alterations in the motor circuit are thought to induce psychomotor slowing, a particularly aberrant function of the premotor cortex.^8,17^ For example, the supplementary motor area was shown to have increased neural activity and connectivity at rest in individuals with catatonia and psychomotor slowing.^18,19^ Therefore, the modulation of supplementary motor area activity with repetitive transcranial magnetic stimulation (rTMS) has been suggested as a potential treatment for psychomotor slowing, given that stimulation of the premotor cortex would likely also exert distant effects within the motor network.^20^

Protocols for rTMS have distinct effects on neural activity. For example, low-frequency rTMS (1 Hz) acts inhibitory, while intermittent theta burst stimulation (iTBS) has facilitatory effects.^21^ Our randomized, double-blind, sham-controlled trial of rTMS for psychomotor slowing in major depressive disorder and schizophrenia suggested that 15 sessions of 1-Hz rTMS may ameliorate psychomotor slowing.^22^ However, to understand this positive effect of 1-Hz rTMS on psychomotor slowing, we aimed at testing the intervention in a larger sample of patients with schizophrenia, including a comprehensive behavioral battery, 1 group with facilitatory stimulation (iTBS), and 1 group without rTMS treatment, to disentangle unspecific effects of the TMS procedure from treatment as usual. Therefore, we tested in a randomized, double-blind, sham-controlled 4-arm trial whether 3 weeks of add-on rTMS would ameliorate psychomotor slowing in psychosis. We hypothesized that inhibitory 1-Hz rTMS would be superior to facilitatory iTBS, sham-rTMS, or no add-on rTMS.

## Methods

### Trial Design

This 4-arm, double-blind, randomized, placebo-controlled clinical trial of add-on rTMS was conducted at the University Hospital of Psychiatry and Psychotherapy, Bern, Switzerland. The protocol (Supplement 1) adhered to the Declaration of Helsinki and was approved by the cantonal ethics committee of Bern. Written informed consent was provided by all participants. Sample size estimations are given in eMethods 1 in Supplement 2. There were no relevant changes to the protocol after the trial commencement. The trial was registered on April 16, 2019, when 3 patients were receiving treatment. Trial registration included 2 primary outcomes instead of 1 in the study protocol (eMethods 2 in Supplement 2).

### Participants

From March 22, 2019, to August 29, 2022, we screened 615 patients for eligibility. Of these, 103 patients were randomized: 26 to 1-Hz rTMS, 25 to iTBS, 28 to sham, and 24 to the waiting group. We included patients aged between 18 and 60 years who were diagnosed with schizophrenia spectrum disorders according to *DSM-5* criteria and had psychomotor slowing as per the Salpêtrière Retardation Rating Scale^23^ (SRRS score ≥15). Exclusions included substance misuse (other than nicotine), conditions associated with impaired or aberrant movement, convulsions, history of hearing problems, other conditions typically excluded from magnetic resonance imaging or TMS, any TMS treatment in the past 3 months, or those who were pregnant or breastfeeding. The full list of inclusion and exclusion criteria are provided in eTable 1 in Supplement 2. Patients continued preexisting medication, including antipsychotics and benzodiazepines. A total of 88 patients received at least 1 rTMS session or completed the waiting period (intention-to-treat population). Follow-up was completed October 31, 2022.

### TMS Protocols

All stimulations were delivered using either MagPro X100, including MagOption or MagPro R30 with theta burst option, both manufactured by Tonica Electronik and distributed by MagVenture. We used the MCF-B70 coil for the real TMS stimulations and the MCF-P-B65 coil for sham stimulations. rTMS application followed published guidelines.^24,25^ Before each session, resting motor thresholds were acquired.^25^ All protocols were delivered in 15 daily sessions over 3 weeks targeting the left supplementary motor area by moving the coil 3 cm anterior from the leg motor area along the midline, which stimulates the bilateral supplementary motor area.^26,27,28^ The coil handle was pointing backward along the midline. Each protocol had identical looking coils and identical duration (eMethods 3 in Supplement 2).

One-hertz rTMS included 960 pulses at an intensity of 110% resting motor thresholds (16:00-minute duration). This protocol is similar to our previous study^22^ and a study in Parkinson disease.^27^ The sham control used the identical stimulation protocol as 1-Hz rTMS for 16 minutes with a placebo coil that looks and sounds identical to the real coil but has no magnetic emissions. The active control with iTBS included 2 series of 600 pulses at 50 Hz (stimulation in 2-second trains every 10 seconds for a total of 190 seconds)^29^ at 80% resting motor threshold separated by a 10-minute pause between the series, totaling 16 minutes, 20 seconds, and 1200 pulses. We applied these changes to the iTBS protocol to harmonize treatment duration.

### Outcomes

The primary outcome was the proportion of responders per treatment arm at week 3, defined as 30% or greater reduction from baseline in SRRS total score (higher values indicating more slowing), as in the previous trial.^22^ The change in SRRS scores from baseline to week 3 was another primary outcome.

Secondary outcomes included responder rates and the course of SRRS in the waiting group following the rTMS treatment phase. We also computed changes from baseline to week 3 in SRRS scores and expert ratings covering general illness severity (Positive And Negative Syndrome Scale [PANSS]),^30^ negative symptoms (Brief Negative Symptom Scale [BNSS]),^31^ catatonia (Bush-Francis Catatonia Rating Scale [BFCRS]),^32^ parkinsonism (Unified Parkinson’s Disease Rating Scale Part III [UPDRS]),^33^ dyskinesia (Abnormal Involuntary Movement Scale [AIMS]),^34^ global functioning (Global Assessment of Functioning [GAF]),^35^ social functioning (Social and Occupational Functioning Assessment Scale [SOFAS]),^36^ and functional capacity (University of California San Diego Performance-Based Skills Assessment [UPSA brief])^37^ (eMethods 4 in Supplement 2). In addition, we acquired self-reported negative symptoms (Self-evaluation of Negative Symptoms [SNS])^38^ and physical activity (International Physical Activity Questionnaire [IPAQ]).^39^ Moreover, the change in total physical activity during waking hours was measured with wrist actigraphy on the nondominant arm (Move4 [movisens])^40^ (eMethods 5 in Supplement 2). Manual dexterity of both hands was tested using the coin rotation task^41^ (eMethods 6 in Supplement 2).

### Procedures

After baseline assessments, the 1-Hz rTMS, iTBS, and sham groups received their allocated interventions for 3 weeks with weekly assessments of SRRS scores and safety. Safety outcomes included adverse stimulation effects after each rTMS session and adverse effect rating scale after 5, 10, and 15 sessions (ie, weeks 1, 2, and 3). The waiting group did not receive any rTMS intervention in the first 3 weeks, but after completing a second baseline assessment, these participants received the 1-Hz rTMS protocol daily until week 6. At baseline, week 3, and week 6 (waiting group only), patients were assessed with clinical and motor rating scales, tasks assessing fine and gross motor behavior, and a test of functional capacity. Daily medication was summarized as mean olanzapine equivalents^42^ for antipsychotics or mean diazepam equivalents^43^ for benzodiazepines. Follow-up assessments including clinical, functional, and motor measures were conducted at 6 weeks and 24 weeks following the week 3 assessments.

### Randomization

After providing informed consent and before baseline measurements, patients were randomized 1:1:1:1 to 1 of the 4 treatment arms. Permutated block randomization lists were generated by research randomizer software (Social Psychology Network) and kept secured, only accessible to one person (S.W.). Treatment allocation was communicated only to the person administering rTMS (N.N.). Allocation was kept in writing in a sealed envelope for each patient (eMethods 7 in Supplement 2).

### Blinding

Outcome assessors, clinical staff, and patients were blind to treatment, except for the waiting group, who expected to receive 1-Hz rTMS after the waiting period. Treatment duration, setting, and TMS machinery were identical for all patients. In 41 patients, we assessed the suspected type of stimulation received at week 3.

### Statistical Analysis

Primary and secondary outcomes were analyzed by 2 researchers (S.W. and N.N.) with SPSS Statistics version 28.0.0.0(190) (IBM). All analyses were run in the intention-to-treat sample (N = 88 with at least 1 rTMS session^44,45,46,47,48^) using the last observation carried forward method to account for missing data. Response rates between treatment arms were compared using a χ^2^ test. Logistic regressions were calculated to obtain odds ratios for responder rates with bootstrapping for CIs using 1000 iterations and Bonferroni correction for post hoc tests. We compared improvement in SRRS scores over 3 weeks between treatment arms using repeated-measures analysis of covariance (ANCOVA) covaried for sex, baseline antipsychotics, baseline benzodiazepines, and the mean dosages of antipsychotics and benzodiazepines during the 3-week intervention. For the 2 primary outcome analyses, 2-sided *P* < .025 was considered significant (0.05 / 2). We corrected ANCOVA post hoc tests for multiple comparisons using Sidak tests. In addition, we explored post hoc tests with least significant difference correction. To test rTMS effects on secondary outcomes (PANSS, BNSS, BFCRS, AIMS, UPDRS, SOFAS, GAF, UPSA brief, IPAQ, and SNS scores; actigraphy; and coin rotation), we calculated repeated measures ANCOVAs with factors group and time, including the covariates sex, baseline antipsychotics, baseline benzodiazepines, and the mean dosages of antipsychotics and benzodiazepines. Frequencies of adverse events and blinding evaluation were calculated using χ^2^ tests with 2-sided *P* ≤ .05 as significance threshold.

## Results

### Dates of Recruitment

**Figure 1.**
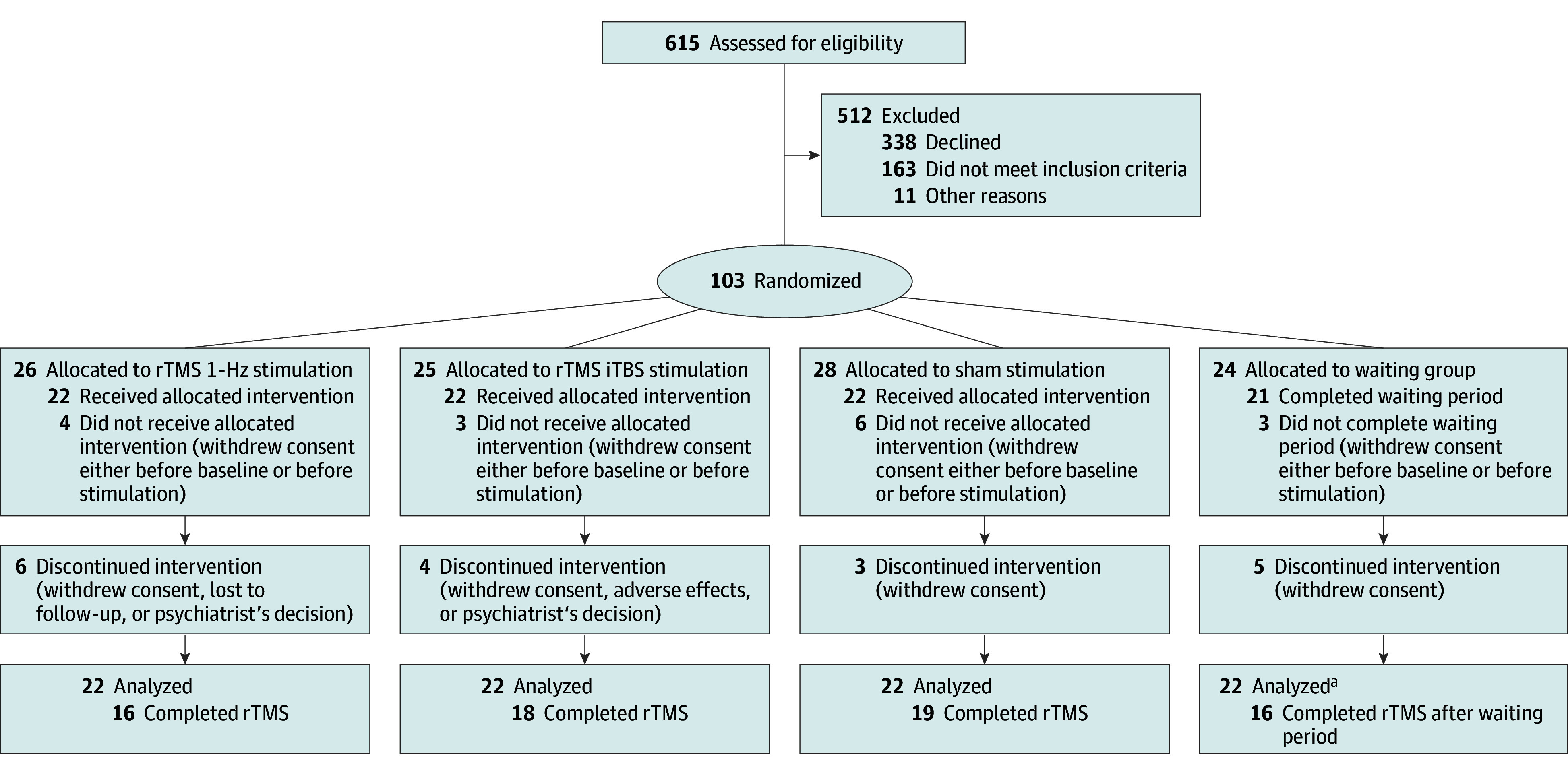
CONSORT Flow Diagram Numbers for dropout reasons were summarized within groups to ensure data privacy. iTBS indicates intermittent theta burst stimulation; rTMS, repetitive transcranial magnetic stimulation. ^a^In the TMS groups, last observation carried forward analyses were conducted for all participants with at least 1 stimulation. In the waiting group, we did last observation carried forward analyses in all participants with baseline data. Thus, 22 were analyzed in this group.

Of the 88 patients analyzed, 45 were men and 43 were women. The mean (SD) age was 36.3 (12.4) years. A total of 69 patients completed the intervention period as well as the week 3 assessments (16 in the 1-Hz rTMS group, 18 in the iTBS group, 19 in sham, and 16 in the waiting group). Reasons for discontinuation were withdrawal of consent (n = 15), treating psychiatrist’s decision (n = 2), lost to follow-up (n = 1), and adverse effects (n = 1) (Figure 1; eTable 2 in Supplement 2).

Baseline demographic and clinical data are given in Table 1. Differences between treatment arms included sex, baseline antipsychotics, baseline benzodiazepines, and the mean dosages of antipsychotics and benzodiazepines.

**Table 1.** Clinical and Demographic Characteristics

Characteristic	Mean (SD)			
	1-Hz rTMS	iTBS	Sham	Waiting
No.	22	22	22	22
Age, y	39.5 (13.2)	33.5 (11.5)	38.2 (12.2)	33.8 (12.4)
Sex, No. (%)^a^				
Male	16 (73)	12 (55)	9 (41)	8 (36)
Female	6 (27)	10 (45)	13 (59)	14 (64)
Education, y	13.0 (3.0)	13.1 (2.2)	12.7 (2.2)	13.2 (2.0)
Duration of illness, y	12.0 (11.9)	7.0 (8.4)	12.9 (12.4)	9.7 (7.0)
No. of episodes	4.5 (4.3)	3.4 (2.9)	5.9 (5.7)	4.7 (3.9)
BMI	26.3 (4.0)	24.2 (4.7)	25.9 (6.6)	24.3 (5.0)
Medication^b^				
Baseline olanzapine equivalents, mg/d^c^	13.3 (10.3)	13.4 (10.6)	22.6 (12.9)	15.8 (11.0)
Baseline diazepam equivalents, mg/d^d^	6.1 (14.6)	5.1 (8.3)	0.5 (1.6)	0.5 (1.5)
Mean olanzapine equivalents, mg/d^e^	14.5 (9.9)	12.0 (7.9)	22.9 (12.9)	15.4 (9.8)
Mean diazepam equivalents, mg/d^f^	3.6 (8.5)	2.3 (4.5)	0.7 (1.4)	0.1 (0.2)
Monotherapy, No. (%)	11 (50)	15 (68)	10 (45)	14 (64)
First-generation antipsychotics, No. (%)	5 (23)	5 (23)	4 (18)	3 (14)
Clozapine, No. (%)	5 (23)	7 (32)	3 (14)	6 (27)
Clinical rating scales				
SRRS total score	24.7 (5.8)	23.3 (7.1)	24.2 (5.7)	23.7 (5.0)
PANSS total score	76.3 (17.1)	81.8 (19.9)	77.3 (14.1)	84.6 (17.8)
PANSS positive score	14.8 (5.1)	16.9 (6.4)	15.0 (4.2)	16.9 (5.8)
PANSS negative score	22.9 (5.4)	23.2 (6.1)	24.7 (6.8)	24.5 (6.0)
BNSS total score	42.0 (14.4)	39.3 (12.2)	45.7 (11.8)	42.2 (12.4)
BFCRS total score	4.9 (3.7)	5.9 (4.9)	7.1 (3.7)	4.2 (3.0)
NCRS total score	9.9 (4.8)	10.6 (5.8)	10.3 (3.3)	9.0 (4.4)
UPDRS total score	18.7 (8.7)	21.3 (13.8)	23.4 (11.4)	20.0 (9.7)
AIMS total score	1.4 (3.1)	1.1 (2.0)	0.9 (2.2)	0.1 (0.4)
NES total score	18.4 (11.6)	16.4 (11.4)	15.0 (7.6)	12.3 (6.5)
GAF	43.7 (11.7)	39.1 (14.1)	39.0 (9.9)	42.1 (12.7)
SOFAS	41.0 (14.9)	40.3 (12.6)	38.0 (10.7)	42.2 (11.7)
UPSA brief	70.6 (13.9)	71.2 (17.9)	74.9 (14.6)	75.3 (12.0)
SNS total score	18.9 (9.3)	21.1 (8.0)	22.0 (8.0)	19.7 (7.8)
IPAQ total score	1008 (1283)	815 (940)	2191 (3732)	875 (1304)
Activity level, counts/h	12 978 (4795)	13 548 (4961)	12 028 (3493)	11 921 (4924)
CR dominant	10.8 (3.2)	10.9 (3.5)	12.2 (4.4)	10.9 (3.4)
CR nondominant	9.6 (3.8)	9.8 (3.3)	10.1 (4.3)	9.4 (2.3)
Cortical excitability, RMT	42 (7)	45 (9)	42 (10)	43 (10)

Abbreviations: AIMS, Abnormal Involuntary Movement Scale; BFCRS, Bush-Francis Catatonia Rating Scale; BMI, body mass index (calculated as weight in kilograms divided by height in meters squared); BNSS, Brief Negative Symptom Scale; CR, coin rotations; GAF, Global Assessment of Functioning Scale; IPAQ, International Physical Activity Questionnaire; iTBS, intermittent theta burst stimulation; NCRS, Northoff Catatonia Rating Scale; NES, Neurological Evaluation Scale; PANSS, Positive and Negative Syndrome Scale; RMT, resting motor threshold; rTMS, repetitive transcranial magnetic stimulation; SNS, Self-evaluation of Negative Symptoms; SOFAS, Social and Occupational Functioning Assessment Scale; SRRS, Salpêtrière Retardation Rating Scale; UPDRS, Unified Parkinson Disease Rating Scale Part III; UPSA brief, University of California San Diego Performance-Based Skills Assessment.

^a^ Group difference: *P* = .07.

^b^ None of the participants received anticholinergics.

^c^ Group difference: *P* = .02.

^d^ Group difference: *P* = .04.

^e^ Group difference: *P* = .005.

^f^ Group difference: *P* = .07.

### Primary Outcomes

**Figure 2.**
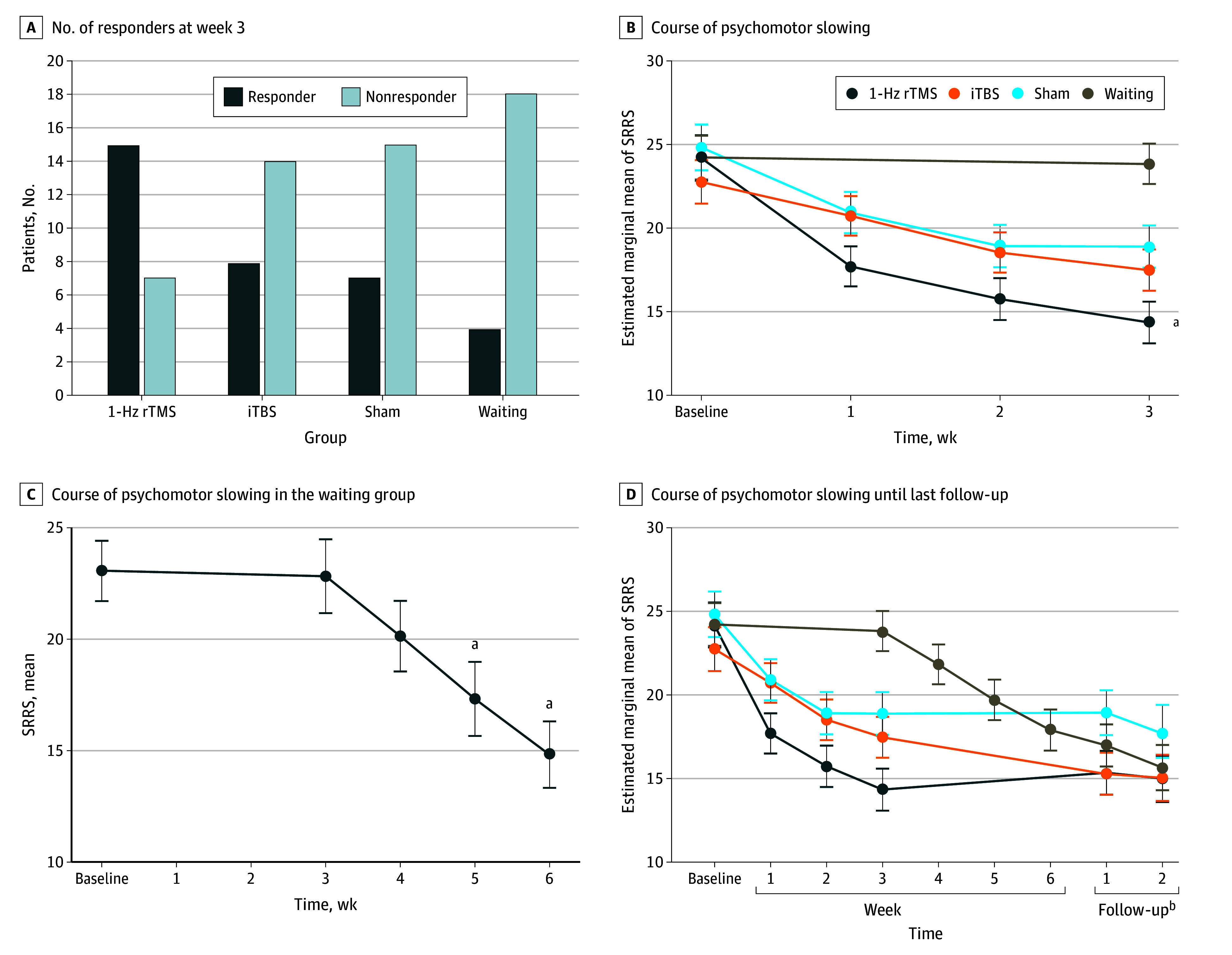
Responder Rates and Course of Psychomotor Slowing Treatment arm covariates (B and D) included age and medication (baseline antipsychotics, baseline benzodiazepines, and mean dosages of antipsychotics and benzodiazepines), using last observation carried forward for missing values. The waiting sample consisted of 16 patients completing the repetitive transcranial magnetic stimulation (rTMS) treatment phase after the 3-week waiting period (C). Error bars represent SEMs. iTBS indicates intermittent theta burst stimulation. ^a^Significant at *P* < .05. ^b^Follow-up 1 took place 6 weeks after end of treatment; follow-up 2, 24 weeks after end of treatment.

In the intention-to-treat analysis with last observation carried forward, we found a significant difference in the proportion of responders following 3 weeks of rTMS (1-Hz rTMS: 15 of 22 [68%], iTBS: 8 of 22 [36%], sham: 7 of 22 [32%]; waiting: 4 of 22 [18%]; χ^2^_3_ = 12.1; *P* = .007) (Figure 2A). The 1-Hz rTMS group had more responders than sham (odds ratio [OR], 0.13; 95% CI, 0.02-0.65; *P* = .03), iTBS (OR, 0.12; 95% CI, 0.02-0.61; *P* = .02), and waiting (OR, 0.04; 95% CI, 0.01-0.22; *P* = .003). One-hertz stimulation was superior to all other protocols (Table 2).

**Table 2.** Raw and Adjusted Odds Ratios (ORs) for Treatment Response at Week 3 (N = 88)

Treatment group comparison	Raw OR (95% CI)^a^^,^^b^	Raw *P* value^c^	Adjusted OR (95% CI)^b^^,^^d^^,^^e^	Adjusted *P* value^c^^,^^d^
1-Hz rTMS vs sham	0.22 (0.06-0.08)	.03	0.13 (0.02-0.65)	.03
1-Hz rTMS vs iTBS	0.27 (0.08-0.93)	.09	0.12 (0.02-0.61)	.02
1-Hz rTMS vs waiting	0.10 (0.03-0.42)	.009	0.04 (0.01-0.22)	.003

Abbreviations: iTBS, intermittent theta burst stimulation; rTMS, repetitive transcranial magnetic stimulation.

^a^ χ^2^_3_ = 11.17; *P* = .01. Omnibus test raw: χ^2^_3_ = 12.66; *P* = .005.

^b^ Confidence intervals have been bootstrapped with 1000 iterations.

^c^ *P* values are Bonferroni corrected.

^d^ Adjustment covariates include baseline sex, baseline antipsychotics, baseline benzodiazepines, and mean dosages of antipsychotics and benzodiazepines.

^e^ χ^2^_3_ = 13.55; *P* = .004. Omnibus test adjusted: χ^2^_8_ = 26.51; *P* = .001.

Repeated-measures ANCOVA of SRRS scores between baseline and week 3 comparing the 4 treatment arms indicated significant effects of time (*F*_1,79_ = 26.6; , η^2^ = 0.25; *P* < .001) and a time-by–treatment arm interaction (*F*_3,79_ = 8.5; η^2^ = 0.25; *P* < .001). Post hoc comparisons demonstrated a pronounced decrease in SRRS scores in the 1-Hz rTMS group vs the waiting group. In addition, least significant difference tests indicated superior performance of 1-Hz rTMS vs sham (Figure 2B; eTables 5 and 6 in Supplement 2).

### Secondary Outcomes

A repeated-measures ANCOVA with weekly SRRS ratings revealed an effect of time (*F*_3,237_ = 16.5; η^2^ = 0.17; *P* < .001) and a time-by–treatment arm interaction (*F*_9,237_ = 6.3; η^2^ = 0.19; *P* < .001). In post hoc tests with Sidak correction, 1-Hz rTMS reduced SRRS scores more compared with the waiting group and sham at trend level. With least significant difference correction, 1-Hz rTMS was superior to sham (eTables 3-6 in Supplement 2). In the waiting group, 10 of 16 participants (63%) responded following 3 weeks of 1-Hz rTMS after the waiting period. In addition, ANOVA with repeated measures of SRRS score demonstrated improvement over time (*F*_4,60_ = 9.9; η^2^ = 0.40; *P* < .001) (Figure 2C).

Follow-up assessments were acquired in 54 patients after 6 weeks and 32 patients after 6 months. A repeated-measures ANCOVA with SRRS last observation carried forward including follow-up assessments still resulted in significant effects of time (*F*_1,237_ = 17.4; η^2^ = 0.18; *P* < .001) and a time-by–treatment arm interaction (*F*_9,237_ = 5.0; η^2^ = 0.16; *P* < .001) (Figure 2D; eTable 7 in Supplement 2). However, Sidak-corrected post hoc tests indicated no differences between treatment arms.

Repeated-measures ANCOVAs of the secondary outcomes demonstrated effects of time in PANSS total score, PANSS positive score, PANSS negative score, PANSS general score, BNSS total score, BNSS asocial score, BFCRS total score, BFCRS abnormal score, BFCRS decreased score, GAF, UPSA brief, and IPAQ. However, time-by–treatment arm interactions were limited to BNSS anhedonia score, BNSS distress score, BFCRS total score, BFCRS abnormal score, and BFCRS decreased score (for last observation carried forward, see eTables 3-7 in Supplement 2; for completers see eTables 8-10 in Supplement 2). The post hoc tests identified a greater decrease in catatonia severity (BFCRS total score) for 1-Hz rTMS vs waiting. Similarly, reductions in BFCRS decreased score were noted in 1-Hz rTMS vs waiting and iTBS vs waiting with Sidak correction, but 1-Hz rTMS was superior to sham with least significant difference correction. iTBS outperformed sham and waiting in decreasing BNSS anhedonia score at week 3 with least significant difference but not with Sidak correction (eTables 3 and 6 in Supplement 2).

#### Blinding Efficacy

Patients receiving rTMS from baseline to week 3 (1-Hz rTMS, iTBS, and sham) were unable to identify the assigned treatment (n = 41; χ^2^_4_ = 1.5; *P* = .82). Thirteen patients (31.7%) correctly guessed that they had received real rTMS or sham.

#### Safety

There were no severe adverse events during the study period and follow-up. Furthermore, no differences appeared in the number of reported adverse effects per treatment (χ^2^_2_ = 0.50; *P* = .78) (Table 3). Adverse effects experienced by participants in the waiting group during rTMS treatment phase are presented in eTable 11 in Supplement 2.

**Table 3.** Adverse Effects During Intervention Period (Baseline to Week 3)

Variable	No. (%)		
	1-Hz rTMS (n = 22)	iTBS (n = 22)	Sham (n = 22)
No adverse effects	6 (27)	10 (45.5)	10 (45.5)
Dizziness	6 (27)	4 (18)	3 (13.5)
Headache or neck pain	6 (27)	10 (45.5)	4 (18)
Fatigue	10 (45.5)	5 (23)	9 (41)
Other	<10 (45.5)^a^	<10 (45.5)^b^	<10 (45.5)^c^

Abbreviations: iTBS, intermittent theta burst stimulation; rTMS, repetitive transcranial magnetic stimulation.

^a^ Including fatigue and flash of light, combined to avoid compromising data identifiability requirements.

^b^ Including inner restlessness, pressure on head, burning sensation on skin of head, and memory difficulties, combined to avoid compromising data identifiability requirements.

^c^ Including eye blinking, bizarre thoughts, suicidal ideation, restlessness, and difficulties swallowing, combined to avoid compromising data identifiability requirements.

## Discussion

Psychomotor slowing is a frequent and troubling symptom of psychosis, associated with poor social functioning and minimally responsive to standard treatment. Neuroimaging work suggests higher neural activity in the supplementary motor area in psychomotor slowing.^18,19,20^ In this randomized clinical trial, we tested whether 15 sessions of add-on daily inhibitory 1-Hz rTMS over the supplementary motor area would reduce psychomotor slowing in psychosis. As hypothesized, 1-Hz stimulation ameliorated psychomotor slowing. This study corroborates the previous transdiagnostic randomized clinical trial on add-on daily 1-Hz rTMS in a new sample of patients with schizophrenia spectrum disorders.^22^ Within 3 weeks of 1-Hz rTMS, 68% of participants achieved response, compared with patients receiving facilitatory iTBS (36%), sham (32%), or no add-on treatment (18%). Furthermore, 63% of patients responded when the 1-Hz rTMS was commenced after a 3-week waiting period. SRRS scores declined in all groups receiving rTMS, but only the 1-Hz stimulation achieved more decrease than sham. The data suggest beneficial effects with rTMS treatment to last until 6 months’ follow-up, although these findings must be interpreted with caution given the low rate of individuals with 6-month follow-up assessments (36%).

While all participants experienced severe psychomotor slowing and received standard care, including medication, daily add-on rTMS had beneficial effects on psychomotor slowing compared to the waiting group. The reason for this general rTMS effect could be the daily routines involved, such as being taken to the rTMS facilities, enjoying extra social interaction with the study team, and treatment expectations.^49,50^ Clearly, the data speak to specifically beneficial effects of the inhibitory supplementary motor area stimulation, which is expected to reduce or modulate resting-state hyperactivity in this region.^18,19^ Still, the effects of the iTBS and sham groups were larger than those of the waiting group. As in the pilot study, approximately one-third of the patients on sham stimulation achieved response.^22^ However, the iTBS effect was much larger than in the previous randomized clinical trial, when we administered only 1 train of 600 pulses per session.^22^ iTBS should have opposite effects (facilitation) than the inhibitory 1-Hz stimulation.^21^ But doubling the iTBS pulses to 1200 on the motor cortex had inhibitory effects in healthy individuals.^51^ In the current study, we repeated iTBS after 10 minutes to harmonize groups regarding the duration and number of stimuli per session. This may have increased individual variability of iTBS effects with few achieving response. While the neural changes associated with rTMS are out of the scope of this report, we need to explore the probability that repeated iTBS with a pause of 10 minutes may exert inhibitory effects in some participants.^51,52,53,54,55^

Changes in the severity of catatonia or negative symptoms were noted between treatment arms. BFCRS total scores and BFCRS subscores indicating decreased motor activity were ameliorated with 1-Hz rTMS, whereas BNSS anhedonia scores improved with iTBS stimulation (eFigure 1 in Supplement 2). This rather unexpected finding speaks to recent work suggesting that iTBS might reduce negative symptoms of schizophrenia when applied on the cerebellar vermis^56,57^ or the dorsolateral prefrontal cortex.^58^ However, we failed to see this effect on any other BNSS subscore or the PANSS negative score.

In general, specific treatment effects on motor rating scales and actigraphy were lacking. While this could argue for a specific effect of the supplementary motor area stimulation on psychomotor slowing, it might be explained by insufficient statistical power for secondary outcomes. The sample size per group was moderate and the last observation carried forward method is very conservative.

rTMS was well tolerated without any severe adverse events. Mild and transient adverse effects were noted in all treatment arms. This study suggests great potential of noninvasive brain stimulation interventions in the motor system.^4,20^ Multiple other target regions and symptoms could be tested in this regard, including transdiagnostic studies.^22,59,60,61,62^

Ideally, the current findings would be replicated in large multicenter trials. Future studies may also test accelerated rTMS protocols with more sessions per day and more stimuli.^63,64^ Continuous theta burst could deliver larger numbers of inhibitory stimuli on the supplementary motor area. Furthermore, future studies should also test whether cognitive slowing could be improved with inhibitory supplementary motor area stimulation.

### Limitations

Using a 4-arm parallel design, we were able to test multiple stimulation types for psychomotor slowing in psychosis. Particularly the inclusion of a waiting group in addition to sham offered valuable insights.^49^ However, some limitations require consideration when interpreting the results. First, the choice of 2 primary outcomes was made during clinical trial registration and is a deviation from the study protocol. Second, blinding is challenging in rTMS trials. Duration, machinery, and setup were identical across study arms. Most participants were unable to identify the rTMS protocol received at week 3. However, the waiting group was aware of their treatment regimen. Third, randomization was conducted before baseline assessments and thus the intention-to-treat population included all patients with at least 1 rTMS session. This is in line with rTMS studies in psychiatry but slightly different from drug trials.^44,45,46,47,48^ Results might indicate greater effects than analyses including individuals who had never received the assigned treatment (eTables 12 and 13 in Supplement 2). Fourth, randomization skewed the distribution of medication regimens and sex between the 4 groups, therefore these variables were included as covariates. Fifth, the sample size was calculated for the continuous primary outcomes and fell short in achieving sufficient power for the secondary outcomes. Sixth, randomization also led to some variance in the levels of catatonia severity suggesting floor effects of the treatment in some groups. Seventh, 14 individuals (16% of the intention-to-treat population) dropped out in the first 3 weeks, which is comparable to other trials.^44^ Last observation carried forward analysis accounted for dropouts.

## Conclusions

In this randomized clinical trial, inhibitory rTMS on the supplementary motor area was safe and effective in reducing psychomotor slowing in patients with psychosis. The exact mechanism of this clinical effect remains to be understood. Larger studies will be needed to disentangle secondary effects in the future.

## Notes

Supplement 1. Trial protocol

Supplement 2. eMethods 1. Sample size estimation eMethods 2. Trial registration eTable 1. Inclusion/Exclusion criteria eMethods 3. Coils eMethods 4. Secondary outcome rating scale subscores eMethods 5. Actigraphy eMethods 6. Coin rotation task eMethods 7. Randomization and allocation concealment eResults eTable 2. Dropout reasons eTable 3. Primary and secondary outcomes according to treatment arm for ITT with LOCF and covariates eTable 4. Primary and secondary outcomes according to treatment arms with LOCF (raw) eTable 5. Post-hoc-p-values of Time with SIDAK correction eTable 6. Post-hoc-p-values the interaction between Time & Treatment arm eTable 7. Follow-ups with LOCF and covariates eTable 8. Primary and secondary outcomes according to treatment arm of completers with covariates eTable 9. Primary and secondary outcomes according to treatment arm with completers (raw) eTable 10. Follow ups of completers with covariates eTable 11. Side-effects in the waiting group eFigure 1. Course of anhedonia eFigure 2. Course of global functioning eTable 12. Baseline variables in the three sample versions eTable 13. Results of the primary outcomes in n=88 (ITT) vs. n=97 (with baseline), covaried for sex, baseline and treatment medication eReferences

Supplement 3. Data sharing statement
