# Supplementary material for: Psychomotor Slowing in Psychosis and Inhibitory Repetitive Transcranial Magnetic Stimulation: A Randomized Clinical Trial
Source: JAMA Psychiatry. 2024 Feb 28;81(6):563–71. doi: 10.1001/jamapsychiatry.2024.0026 (PMC10902782; doi:10.1001/jamapsychiatry.2024.0026)
Supplement: Supplement 1. — Trial protocol [file jamapsychiatry-e240026-s001.pdf]

Overcoming psychomotor slowing in psychosis (OCoPS-P) – A 3-week, randomized, double-blind, placebo-controlled trial of add-on repetitive transcranial magnetic stimulation for psychomotor slowing in psychosis

Clinical Study Protocol

Short Title:	OCoPS-P
Translation	Überwinden von psychomotorischer Verlangsamung bei Psychosen - eine randomisierte, doppelblinde, plazebo-kontrollierte Studie zur Wirkung von transkranieller Magnetstimulation auf die psychomotorischer Verlangsamung bei Psychosen
Study Type:	Clinical trial testing the effects of 15 sessions of repetitive transcranial magnetic stimulation on psychomotor slowing in psychosis
Study Categorisation:	Risk category A
Study Registration:	Intended registry: clinicaltrials.gov<br>Registration number (from FOPH portal) eventually other registries and numbers if applicable
Study Identifier:	OCoPS-P
Sponsor, Sponsor-Investigator or Principal Investigator:	Prof. Dr. med. Sebastian Walther, University Hospital of Psychiatry, University of Bern, Murtenstrasse 21, 3008 Switzerland<br>Phone: 031 632 8979, fax: 031 632 8950, Email: sebastian.walther@upd.unibe.ch
Investigational Product:	Transcranial Magnetic Stimulation
Protocol Version and Date:	Version 1.0 November 20th 2018

CONFIDENTIAL

The information contained in this document is confidential and the property of Prof. Dr. med. Sebastian Walther (the sponsor). The information may not - in full or in part - be transmitted, reproduced, published, or disclosed to others than the applicable Competent Ethics Committee(s) and Regulatory Authority(ies) without prior written authorisation from the sponsor except to the extent necessary to obtain informed consent from those who will participate in the study.

Signature Page(s)

Study number	Study registry and registration number
Study Title	Overcoming psychomotor slowing in psychosis (OCoPS-P) – A 3-week, randomized, double-blind, placebo-controlled trial of add-on repetitive transcranial magnetic stimulation for psychomotor slowing in psychosis

The Sponsor-Investigator and trial statistician have approved the protocol version 1.0 (20.11.2018), and confirm hereby to conduct the study according to the protocol, current version of the World Medical Association Declaration of Helsinki, ICH-GCP guidelines or ISO 14155 norm if applicable and the local legally applicable requirements.

Sponsor-Investigator:
Prof. Dr. med. Sebastian Walther

Bern 22.11.2018

Place/Date   Signature

Local Principal Investigator at study site*:

I have read and understood this trial protocol and agree to conduct the trial as set out in this study protocol, the current version of the World Medical Association Declaration of Helsinki, ICH-GCP guidelines or ISO 14155 norm and the local legally applicable requirements.

Site	University Hospital of Psychiatry, Bern
Principal investigator	Prof. Dr. med. Sebastian Walther

Bern, 22.11.2018

Place/Date

Signature

Note: In multicentre studies, this page must be individually signed by all participating Local Principal Investigators.

Table of Contents

STUDY SYNOPSIS **8**
ABBREVIATIONS **12**
STUDY SCHEDULE **13**
1. STUDY ADMINISTRATIVE STRUCTURE **15**
1.1 Sponsor, Sponsor-Investigator 15
1.2 Principal Investigator(s) 15
1.3 Statistician ("Biostatistician") 15
1.4 Laboratory 15
1.5 Monitoring institution 15
1.6 Data Safety Monitoring Committee 15
1.7 Any other relevant Committee, Person, Organisation, Institution 15
2. ETHICAL AND REGULATORY ASPECTS **16**
2.1 Study registration 16
2.2 Categorisation of study 16
2.3 Competent Ethics Committee (CEC) 16
2.4 Competent Authorities (CA) 16
2.5 Ethical Conduct of the Study 16
2.6 Declaration of interest 16
2.7 Patient Information and Informed Consent 16
2.8 Participant privacy and confidentiality 17
2.9 Early termination of the study 17
2.10 Protocol amendments 17
3. BACKGROUND AND RATIONALE **18**
3.1 Background and Rationale 18
3.1.1 Schizophrenia 18
3.1.2 Motor system pathology in schizophrenia 18
3.1.3 Psychomotor slowing 19
3.1.4 Current treatment options of psychomotor slowing 19
3.1.5 Psychomotor slowing and motor network dysfunction 19
3.1.6 Intracortical excitability 20
3.1.7 Brain stimulation for psychomotor slowing 21
3.1.8 State of research summary 21
3.2 Investigational Product (treatment, device) and Indication 22
3.3 Preclinical Evidence 22
3.4 Clinical Evidence to Date 22
3.5 Dose Rationale / Medical Device: Rationale for the intended purpose in study (pre-market MD)22
3.6 Explanation for choice of comparator (or placebo) 22
3.7 Risks / Benefits 22
3.8 Justification of choice of study population 23
4. STUDY OBJECTIVES **24**
4.1 Overall Objective 24
4.2 Primary Objective 24
4.3 Secondary Objectives 24
4.4 Safety Objectives 24

5. **STUDY OUTCOMES** **24**
5.1 Primary Outcome 24
5.2 Secondary Outcomes 24
5.2.1 Clinical outcomes 24
5.2.2 Behavioral outcomes 25
5.2.3 Physiological outcomes 25
5.2.4 Neuroimaging outcomes 25
5.3 Other Outcomes of Interest 25
5.4 Safety Outcomes 25
6. **STUDY DESIGN** **25**
6.1 General study design and justification of design 25
6.2 Methods of minimising bias 26
6.2.1 Randomisation 26
6.2.2 Blinding procedures 26
6.2.3 Other methods of minimising bias 26
6.3 Unblinding Procedures (Code break) 26
7. **STUDY POPULATION** **26**
7.1 Eligibility criteria 26
7.2 Recruitment and screening 27
7.3 Assignment to study groups 27
7.4 Criteria for withdrawal / discontinuation of participants 27
8. **STUDY INTERVENTION** **28**
8.1 Identity of Investigational Products (treatment / medical device) 28
8.1.1 Experimental Intervention (treatment / medical device) 28
8.1.2 Control Intervention (standard/routine/comparator treatment / medical device) 28
8.1.3 Packaging, Labelling and Supply (re-supply) 28
8.1.4 Storage Conditions 28
8.2 Administration of experimental and control interventions 28
8.2.1 Experimental Intervention 28
8.2.2 Control Intervention 29
8.3 Dose / Device modifications 29
8.4 Compliance with study intervention 29
8.5 Data Collection and Follow-up for withdrawn participants 29
8.6 Trial specific preventive measures 29
8.7 Concomitant Interventions (treatments) 29
8.8 Study Drug / Medical Device Accountability 29
8.9 Return or Destruction of Study Drug / Medical Device 29
9. **STUDY ASSESSMENTS** **30**
9.1 Study flow chart(s) / table of study procedures and assessments 30
9.1.1 Patients of treatment groups 1-3 30
9.1.2 Patients of treatment group 4 (waiting group) 33
9.1.3 Control subjects 36
9.2 Assessments of outcomes 37
9.2.1 Assessment of primary outcome 38
9.2.2 Assessment of secondary outcomes 38
9.2.3 Assessment of other outcomes of interest 39

9.2.4 Assessment of safety outcomes ... 39
9.2.5 Assessments in participants who prematurely stop the study ... 40
9.3 Procedures at each visit ... 40
9.3.1 Screening visit ... 40
9.3.2 Visit 2 (baseline, week 0) ... 40
9.3.3 Visit 3 and 4 (week 1 and 2) ... 40
9.3.4 Visit 5 (end of intervention, week 3) ... 40
9.3.5 Visit 6 and 7 (follow-up, week 6 and 24) ... 41
10. SAFETY ... 41
10.1 Medical Device Category A studies ... 41
10.1.1 Definition and Assessment of safety related events ... 41
10.1.2 Reporting of (Serious) Adverse Events and other safety related events ... 42
10.1.3 Follow up of (Serious) Adverse Events ... 43
11. STATISTICAL METHODS ... 43
11.1 Hypotheses ... 43
11.2 Determination of Sample Size ... 44
11.3 Statistical criteria of termination of trial ... 44
11.4 Planned Analyses ... 44
11.4.1 Datasets to be analysed, analysis populations ... 44
11.4.2 Primary Analysis ... 44
11.4.3 Secondary Analyses ... 45
11.4.4 Interim analyses ... 45
11.4.5 Safety analysis ... 45
11.4.6 Deviation(s) from the original statistical plan ... 45
11.5 Handling of missing data and drop-outs ... 45
12. QUALITY ASSURANCE AND CONTROL ... 46
12.1 Data handling and record keeping / archiving ... 46
12.1.1 Case Report Forms ... 46
12.1.2 Specification of source documents ... 46
12.1.3 Record keeping / archiving ... 46
12.2 Data management ... 46
12.2.1 Data Management System ... 46
12.2.2 Data security, access and back-up ... 47
12.2.3 Analysis and archiving ... 47
12.2.4 Electronic and central data validation ... 47
12.3 Monitoring ... 47
12.4 Audits and Inspections ... 48
12.5 Confidentiality, Data Protection ... 48
12.6 Storage of biological material and related health data ... 48
13. PUBLICATION AND DISSEMINATION POLICY ... 48
14. FUNDING AND SUPPORT ... 48
14.1 Funding ... 48
14.2 Other Support ... 48

15. INSURANCE 48
16. REFERENCES 49
17. APPENDICES 55

STUDY SYNOPSIS

**Sponsor / Sponsor-Investigator**	Prof. Dr. med. Sebastian Walther,<br>University Hospital of Psychiatry, University of Bern, Murtenstrasse 21, 3008 Switzerland<br>Phone: 031 632 8979, fax: 031 632 8950, Email: sebastian.walther@upd.unibe.ch
**Study Title:**	Overcoming psychomotor slowing in psychosis (OCoPS-P) – A 3-week, randomized, double-blind, placebo-controlled trial of add-on repetitive transcranial magnetic stimulation for psychomotor slowing in psychosis
**Short Title / Study ID:**	OCoPS-P
**Protocol Version and Date:**	Protocol version 1.0 (20th November 2018)
**Trial registration:**	Intended at clinicaltrials.gov
**Study category and Rationale**	Category A: TMS device with CE certificate and use according to guidelines and manual. In addition short cerebral MRI and TMS experiments at baseline and endpoint. Minimal risk involved.
**Clinical Phase:**	Not applicable.

**Background and Rationale:**	Schizophrenia is a chronic disorder causing tremendous burden to the patients, families and society. Besides prominent symptoms such as hallucinations, delusions, and thought disorder, the majority of patients also experiences motor abnormalities. Converging evidence links aberrant structure and function of the cerebral motor network to schizophrenia pathology, particularly to motor abnormalities. One of the most frequent motor abnormalities is psychomotor slowing (PS), which may impact both gross and fine motor behavior. While PS causes significant distress and predicts poor outcome, researchers are just starting to understand its pathobiology. First evidence points to aberrant functional and structural connectivity within the cerebral motor network in schizophrenia patients with PS, particularly in connections between premotor/motor cortex and thalamus, as well as between motor cortex and cerebellum. In addition, severe motor inhibition was linked to increased neural activity in the premotor cortex. Repetitive transcranial magnetic stimulation (rTMS) may temporarily alter brain activity. Pilot data from an ongoing double blind RCT indicate that 15 sessions of inhibitory rTMS on the premotor cortex alleviate PS. The pathobiology of PS, however, is still unknown. This study will combine a motor battery, advanced neuroimaging, and rTMS to probe the cerebral motor network contributions to PS.<br><br>The aims of this study are<br><br>(1) to investigate clinical and neural changes following 3 weeks of rTMS treatment (inhibitory, facilitatory or placebo), (2) to characterize neural correlates of psychomotor slowing (PS) on the network level, (3) to explore short term changes of PS by testing a waiting list cohort, and (4) to test the clinical outcome of rTMS for PS at 6-month follow-up.<br><br>To reach these aims, we plan to investigate four groups of schizophrenia patients (total 88) in a randomized, double blind, 4-arm sham-controlled trial of 15 rTMS sessions in 3 weeks with pre and post intervention MRI scans and a clinical follow-up at 6 months. One group will first be kept on a 3 week waiting list and then enter the study. Longitudinal MRI scans and motor tests separated by 3 weeks will also be applied to a control group of 40 healthy subjects for comparisons with the patient groups. We hypothesize that (1) PS would be linked to increased functional connectivity in motor cortical-basal ganglia loops as well as motor cortical-cerebellar loops, (2) inhibitory rTMS to the premotor cortex will reduce motor network functional connectivity and thus alleviate PS, (3) patients on the waiting list may experience stable PS severity, and finally, (4) patients responding to rTMS treatment of PS will have superior clinical and functional outcomes at 6-month follow-up. Thus, the study will substantially contribute to the understanding of PS by describing and probing the neural alterations in the motor network in schizophrenia associated with behavioral PS. Therefore, the study will impact future treatment strategies for PS and inform on the causal network pathology in schizophrenia
**Objective(s):**	1. investigate clinical and neural changes following 3 weeks of rTMS treatment (inhibitory, facilitatory or placebo)<br>2. characterize neural correlates of psychomotor slowing (PS) on the network level<br>3. explore short term changes of PS by testing a waiting list cohort<br>4. test the clinical outcome of rTMS for PS at 6-month follow-up.

**Outcome(s):**	1. Change from baseline to week 3 in SRRS scores. Furthermore, proportion of responders per study arm after 15 sessions rTMS (response = 30% reduction in SRRS scores from baseline)<br>2. Associations between multiple measures of motor function and neuroimaging markers, e.g. resting state perfusion or functional connectivity within the motor system<br>3. Change in SRRS from baseline to week 3 within the waiting list group, also comparison of SRRS change from baseline between waiting list group and placebo group.<br>4. Change in symptoms (PANSS, BNSS, SRRS) and functioning (SOFAS) from baseline to 6-month follow-up
**Study design:**	randomised, double-blind, four-arm, placebo-controlled trial of 3 weeks add-on rTMS for psychomotor slowing in schizophrenia spectrum disorders
**Inclusion / Exclusion criteria:**	Inclusion:<br>• Right-handed subjects, ages 18–60 years.<br>• Patients: schizophrenia spectrum disorders according to DSM-5 with psychomotor slowing (SRRS score ≥ 15). Patients are necessary, because only patients have the target symptoms, i.e. psychomotor slowing<br>• Controls: only for pre-/post comparisons of neuroimaging and physiology, **no intervention in controls**<br>Exclusion:<br>• General: Substance abuse or dependence other than nicotine. Past or current medical or neurological condition associated with impaired or aberrant movement, such as dystonia, idiopathic parkinsonism or stroke. History of head trauma with concurrent loss of consciousness. Standard exclusion criteria for MRI scanning and TMS; e.g. metal implants, pregnancy. History of any hearing problems or ringing in the ears<br>• Patients: rTMS treatment for any condition within the past 3 months.<br>Controls: history of any psychiatric disorder. First-degree relatives with schizophrenia spectrum disorders.
**Measurements and procedures:**	Participants will be screened and randomized to one of four arms before baseline assessments. Intervention period will be three weeks. Each week the primary outcome variable and safety will be assessed. At baseline and end of intervention (week 3), patients will be assessed with clinical and motor rating scales, tasks assessing fine and gross motor behaviour, TMS measures of cortical excitability, posturography, MRI neuroimaging, and tests of social and community functioning. Follow-ups will be conducted at week 6 and 24 including clinical and motor measures, cortical excitability, and social and community functioning. For cross-sectional comparisons of cortical excitability and neuroimaging, a group of 40 healthy control subjects matched for age, gender, and education, will be tested longitudinally with neuroimaging, motor tests, cortical excitability and posturography at baseline and week 3. Controls will not receive any intervention.
**Study Product / Intervention:**	low-frequency rTMS has inhibitory effects on brain function. We will apply 1'000 pulses at 1 Hz over the left SMA at an intensity of 110% of the resting motor threshold (approximate duration 17 minutes) in a total of 15 daily sessions (5 per week)

**Control Intervention (if applicable):**	**Active control:** Intermittent theta burst (iTBS) enhances local brain activity. We will apply 600 pulses at 50 Hz (stimulation in 2 sec trains every 10 sec for 190 sec in total) over the left SMA at an intensity of 80% of the resting motor threshold. iTBS will be repeated after15 min totaling to 1200 pulses per session in a total of 15 daily sessions (5 per week).<br><br>**Placebo control:** We will use a placebo-coil that looks identical to the real one and makes identical noises. Stimulation parameters are the same as in the active intervention, except that no magnetic pulse is emitted. Thus, placebo coil will be placed over the left SMA with 1000 clicks at 1 Hz (approximate duration 17 minutes) in a total of 15 daily sessions (5 per week).<br><br>**Waiting list:** This group will have baseline measures at baseline and week 3 and receive the active protocol from week 3 to week 6.
**Number of Participants with Rationale:**	Number of participants in the intervention: 88 patients (22 per protocol arm). In addition, to complement neuroimaging and neurophysiological analyses (no intervention) we will include 40 healthy control subjects matched for age, gender, and education.
**Study Duration:**	Total study duration will be 4 years. Total duration of participant recruitment will be 3 years.
**Study Schedule:**	Planned 03/2019 of First-Participant-In<br>Planned 06/2022 of Last-Participant-Out
**Investigator(s):**	- see Sponsor-Investigator
**Study Centre(s):**	Single-centre trial at the University Hospital of Psychiatry, Bern
**Statistical Considerations:**	The main effects of the 3-week interventions on PS (SRRS scores) will be calculated in a repeated measures design including 2 measures (Baseline, week 3) and four groups (lf-rTMS, iTBS, sham, waiting group-lf-rTMS). Assuming a moderate effect size (f = 0.23) as indicated by our pilot data in a repeated measures ANOVA with moderate correlation between time points (0.5), a power of 0.95 and an alpha = 0.05, we would need 88 patients (22 per group).
**GCP Statement:**	This study will be conducted in compliance with the protocol, the current version of the Declaration of Helsinki, the ICH-GCP or ISO EN 14155 (as far as applicable) as well as all national legal and regulatory requirements.

ABBREVIATIONS

AE	Adverse Event
BASEC	Business Administration System for Ethical Committees, (https://submissions.swissethics.ch/en/)
BNSS	Brief Negative Symptom Scale
CA	Competent Authority (e.g. Swissmedic)
CEC	Competent Ethics Committee
CRF	Case Report Form
ClinO	Ordinance on Clinical Trials in Human Research *(in German: KlinV, in French: OClin, in Italian: OSRUm)*
eCRF	Electronic Case Report Form
CTCAE	Common terminology criteria for adverse events
DSUR	Development safety update report
GCP	Good Clinical Practice
IB	Investigator's Brochure
Ho	Null hypothesis
H1	Alternative hypothesis
HRA	Federal Act on Research involving Human Beings *(in German: HFG, in French: LRH, in Italian: LRUm)*
IMP	Investigational Medicinal Product
IIT	Investigator-initiated Trial
ISO	International Organisation for Standardisation
iTBS	Intermittent Theta Burst Stimulation
ITT	Intention to treat
MD	Medical Device
MedDO	Medical Device Ordinance *(in German: MepV, in French: ODim)*
PANSS	Positive And Negative Syndrome Scale
PI	Principal Investigator
PS	Psychomotor Slowing
rTMS	Repetitive Transcranial Magnetic Stimulation
SAE	Serious Adverse Event
SDV	Source Data Verification
SMA	Supplementary Motor Area
SOP	Standard Operating Procedure
SPC	Summary of product characteristics
SRRS	Salpêtrière Retardation Rating Scale
SOFAS	Social and Occupational Functioning Scale
SUSAR	Suspected Unexpected Serious Adverse Reaction
TMF	Trial Master File
UPSA Brief	UCSD Performance-based Skills Assessment Brief version

STUDY SCHEDULE

Study Periods	Screening	Treatment, Intervention Period				Follow-up	
Visit	1	2*	3	4	5	6	7
Time (week)	-1	0	1	2	3	6	24
Patient Information and Informed Consent	x						
Demographics	x						
CASH and SCID (history)	x						
In- /Exclusion Criteria	x						
Physical Examination	x						
Pregnancy Test	x						
Psychopathology (TALD, NES)		x					
Randomisation		x					
Administer rTMS (5 sessions per week)		x	x	x			
Primary Variable SRRS		x	x	x	x	x	x
Motor scales UPDRS, BFCRS, AIMS, IPAQ		x			x	x	x
Psychopathology (PANSS, BNSS, SNS)		x			x	x	x
Actigraphy and coin rotation		x			x	x	x
Posturography		x			x		
TMS cortical excitability		x			x	x	x
Cerebral MRI		x			x		
Functional outcome (SOFAS, GAF, UPSA-brief)		x				x	x
Concomitant Therapy		x	x	x	x		
Adverse Events		x	x	x	x		

* please note that in the waiting group assessments of visit 2 will be repeated after 3 weeks and thereafter the protocol will be identical (see below).

Study protocol for patients in the waiting group

Study Periods	Scree ning	Treatment, Intervention Period					Follow-up	
Visit	1	2	3	4	5	6	7	8
Time (week)	-1	0	3	4	5	6	9	24
Patient Information and Informed Consent	x							
Demographics	x							
CASH and SCID (history)	x							
In- /Exclusion Criteria	x							
Physical Examination	x							
Pregnancy Test	x							
Psychopathology (TALD, NES)		x						
Randomisation		x						
Administer rTMS (5 sessions per week)			x	x	x			
Primary Variable SRRS		x	x	x	x	x	x	x
Motor scales UPDRS, BFCRS, AIMS, IPAQ		x	x			x	x	x
Psychopathology (PANSS, BNSS, SNS)		x	x			x	x	x
Actigraphy and coin rotation		x	x			x	x	x
Posturography		x	x			x		
TMS cortical excitability		x	x			x	x	x
Cerebral MRI		x	x			x		
Functional outcome (SOFAS, GAF, UPSA-brief)		x	x				x	x
Concomitant Therapy		x	x	x	x	x		
Adverse Events			x	x	x	x		

Study protocol for control subjects

Study Periods	Screening	Observation period	
Visit<br>Time (week)	1<br>-1	2<br>0	3<br>3
Proband Information and Informed Consent	x		
Demographics	x		
SCID	x		
In- /Exclusion Criteria	x		
Physical Examination	x		
Pregnancy Test	x		
Primary Variable SRRS		x	x
Motor scales UPDRS, BFCRS, AIMS, IPAQ		x	x
Actigraphy and coin rotation		x	x
Posturography		x	x
TMS cortical excitability		x	x
Cerebral MRI		x	x

1. STUDY ADMINISTRATIVE STRUCTURE

1.1 Sponsor, Sponsor-Investigator

Prof. Dr. med. Sebastian Walther,

University Hospital of Psychiatry, University of Bern, Murtenstrasse 21, 3008 Bern, Switzerland, phone: 031 632 4635, fax: 031 632 8950, Email: sebastian.walther@upd.unibe.ch

Prof. Walther will be Sponsor-Investigator; no further international sites are planned

Roles: protocol, study design, supervision of data collection and management, data analysis, data interpretation and writing of the report.

1.2 Principal Investigator(s)

Identical to sponsor, see 1.1.

1.3 Statistician ("Biostatistician")

Dr. Petra Viher, PhD, University Hospital of Psychiatry, University of Bern, Bolligenstrasse 111, 3000 Bern, Switzerland. Phone: 031 930 9757, Email: petra.viher@upd.unibe.ch

1.4 Laboratory

All of the procedures except neuroimaging will be performed at the University Hospital of Psychiatry, Bern. The devices and infrastructure are provided by the Translational research center at the University Hospital of Psychiatry, Bern, Bolligenstrasse 111, 3000 Bern 60, Switzerland.

Neuroimaging acquisition (magnetic resonance imaging – MRI) will be performed at the University Hospital Inselspital Bern, Institute of Diagnostic and Interventional Neuroradiology. Collaborator Prof. Dr. med. Roland Wiest.

1.5 Monitoring institution

Dr. phil. Jessica Peter, University Hospital of Old Age Psychiatry and Psychotherapy, University of Bern, Bolligenstrasse 111, 3000 Bern, Switzerland. Phone: 031 932 8903 Email: Jessica.peter@upd.unibe.ch

1.6 Data Safety Monitoring Committee

DSMC is not needed. The study aim is not to test the efficacy of a specific product. The objective is to test whether repetitive transcranial magnetic stimulation may improve psychomotor slowing and how it interferes with neurophysiology.

1.7 Any other relevant Committee, Person, Organisation, Institution

Study collaborators

Prof. Dr. Roland Wiest, University Institute of Diagnostic and Interventional Neuroradiology, Inselspital Bern, Switzerland. MRI acquisition

Prof. Dr. Andrea Federspiel, Neuroimaging Unit, Translational Research Center, University Hospital of Psychiatry, Bern, Switzerland, MRI analyses

Prof. Dr. Jessica Bernard, Department of Psychological and Brain Sciences, Texas A & M University, College Station, TX, USA, MRI analyses support

Prof. Dr. Roger Kalla, Department of Neurology, University Hospital Inselspital, Bern, Switzerland, posturography

2. ETHICAL AND REGULATORY ASPECTS

The decision of the CEC and Swissmedic/foreign competent authority concerning the conduct of the study will be made in writing to the Sponsor-Investigator before commencement of this study. The clinical study can only begin once approval from all required authorities has been received. Any additional requirements imposed by the authorities shall be implemented.

2.1 Study registration

Registration is planned in ClinicalTrials.gov and the Swiss KOFAM site

2.2 Categorisation of study

The trial is in Risk Category A. There is only minimal risk associated with the trial. The TMS device has CE certification and approved for clinical use. It will be applied according to the manual and guidelines[1, 2]. TMS has been widely used in neuroscience, and in clinical trials on depression, schizophrenia and chronic pain. Participants will receive 15 daily stimulations. Effects are expected to last for 2-4 weeks after the last stimulation. TMS is also safe in repeated administration[1, 2].

Assessments include standard clinical rating scales, short specific tests of motor behaviour, and a standard cerebral magnetic resonance imaging at baseline and after 3 weeks of stimulation. All assessments have been applied to schizophrenia patients before and are generally well tolerated.

2.3 Competent Ethics Committee (CEC)

The local Bern Ethics Committee is the Competent Ethics Committee (CEC). No sites outside the canton of Bern are planned.

The Bern Ethics Committee will receive reports of all changes in the research activity and all unanticipated problems involving risks to humans; including in case of planned or premature study end and the final report. No changes are made to the protocol without prior Sponsor and CEC approval, except where necessary to eliminate apparent immediate hazards to study participants. Premature study end or interruption of the study is reported within 15 days. The regular end of the study is reported to the CEC within 90 days, the final study report shall be submitted within one year after study end. Amendments are reported according to chapter 2.10.

2.4 Competent Authorities (CA)

Not applicable – Category A

2.5 Ethical Conduct of the Study

The study will be carried out in accordance to the protocol and with principles enunciated in the current version of the Declaration of Helsinki, the guidelines of Good Clinical Practice (GCP) issued by ICH, in case of medical device: the European Regulation on medical devices 2017/745 and the ISO Norm 14155 and ISO 14971, the Swiss Law and Swiss regulatory authority's requirements. The CEC and regulatory authorities will receive annual safety and interim reports and be informed about study stop/end in agreement with local requirements.

2.6 Declaration of interest

There is no conflict of interest. Funding is provided by independent grants and the Swiss National Science Foundation (see funding 14.1)

2.7 Patient Information and Informed Consent

Participants will be informed by members of the study team about the aims of the study, planned procedures and risks involved. They will receive written information on the study. This information will be provided prior to study inclusion during screening. The participants will also be informed about the compensation of 200 CHF after they completed the study procedures. The participant will be informed by the investigators that his/her medical records may be examined by authorised individuals other than their treating physician.

All participants for the study will be provided a participant information sheet and a consent form describing the study and providing sufficient information for participant to make an informed decision about their participation in the study. A minimum of 24 h time will be given to participants to decide on

whether to participate or not. Whenever necessary, the potential participant can take up to 2 weeks to decide on participation.

The patient information sheet and the consent form will be submitted to the CEC and to the competent authority (as applicable) to be reviewed and approved. The formal consent of a participant, using the approved consent form, must be obtained before the participant is submitted to any study procedure.

The participant should read and consider the statement before signing and dating the informed consent form, and should be given a copy of the signed document. The consent form must also be signed and dated by the investigator (or his designee) and it will be retained as part of the study records.

2.8 Participant privacy and confidentiality

The investigator affirms and upholds the principle of the participant's right to privacy and that they shall comply with applicable privacy laws. Especially, anonymity of the participants shall be guaranteed when presenting the data at scientific meetings or publishing them in scientific journals.

Individual subject medical information obtained as a result of this study is considered confidential and disclosure to third parties is prohibited. Subject confidentiality will be further ensured by utilising subject identification code numbers to correspond to treatment data in the computer files.

For data verification purposes, authorised representatives of the Sponsor (-Investigator), a competent authority (e.g. Swissmedic), or an ethics committee may require direct access to parts of the medical records relevant to the study, including participants' medical history.

2.9 Early termination of the study

The Sponsor-Investigator may terminate the study prematurely according to certain circumstances, for example:

- ethical concerns,
- insufficient participant recruitment,
- when the safety of the participants is doubtful or at risk, respectively,
- alterations in accepted clinical practice that make the continuation of a clinical trial unwise,
- early evidence of benefit or harm of the experimental intervention

2.10 Protocol amendments

Substantial amendments are only implemented after approval of the CEC and CA respectively.

Under emergency circumstances, deviations from the protocol to protect the rights, safety and well-being of human subjects may proceed without prior approval of the sponsor and the CEC/CA. Such deviations shall be documented and reported to the sponsor and the CEC/CA as soon as possible.

All non-substantial amendments are communicated to the CA as soon as possible if applicable and to the CEC within the Annual Safety Report (ASR).

3. BACKGROUND AND RATIONALE

3.1 Background and Rationale

3.1.1 Schizophrenia

Schizophrenia is a severe disorder with a life-long course affecting approximately 2-3% of the population. Even though the outcome is heterogeneous, schizophrenia usually causes tremendous individual burden, intense costs for society, reduced quality of life, impaired occupational performance and reduction of life expectancy by 10-20 years[3]. Schizophrenia is an adolescent-onset disorder with neurodevelopmental origins[3]. Current models suggest that genetic risk, early hazards to brain maturation, social adversities during childhood and the evolution of cognitive biases predispose subjects to psychosis in times of stress[4]. The schizophrenia syndrome is characterized by symptom clusters including positive symptoms (e.g. delusions and hallucinations), negative symptoms (e.g. avolition and affective flattening), disorganized thought, motor abnormalities, mood disturbances, impaired cognition, anxiety and lack of insight[5]. Brain structure and function in schizophrenia is abnormal at multiple levels. For example, schizophrenia patients share altered organization of functional brain networks[6] and underlying white matter fiber connections[7, 8]. Therefore, schizophrenia has been conceptualized as a disconnection syndrome, which may explain some of the typical symptoms[9]. Indeed, first evidence indicates an association between abnormal motor behavior and structural as well as functional alterations in the motor network in schizophrenia[10, 11]. Researchers are just starting to understand the network alterations linked to clinical symptoms in schizophrenia[12]. **The ultimate goal** would be to **normalize altered brain function at the network level** in order to **improve the clinical significant behavioral abnormalities**.

3.1.2 Motor system pathology in schizophrenia

A set of motor abnormalities including signs such as bizarre posture, peculiar gait, facial and limb dyskinesia, immobility, rigor, or excessive movement have been reported in schizophrenia.

Motor abnormalities have long been exclusively linked to medication side effects; however, converging evidence demonstrates that motor abnormalities frequently occur before medication is commenced and even long before the onset of psychosis[10, 13-15]. Up to 67% of first episode, treatment naïve patients experience at least one motor sign[16]. The contribution of medication is heterogeneous and probably overestimated.

Motor abnormalities are key cues for stigmatization and pose significant distress on patients[17, 18]. Furthermore, recent evidence supports the predictive value of motor abnormalities in psychosis for poor functional outcome[15, 19]. For example, motor abnormalities impact nonverbal behaviors such as gestures[20], which are critical for social functioning[21]. This way, motor abnormalities contribute to poor functional outcome in schizophrenia.

A large network of the brain is devoted to motor behavior, including cortical frontal motor and premotor areas, the basal ganglia, thalamus, brainstem and cerebellum (see figure 1). Evidence across multiple neuroimaging approaches supports that schizophrenia pathobiology is tied to alterations within the cerebral motor system[22].

Figure 1. Key components of the motor network

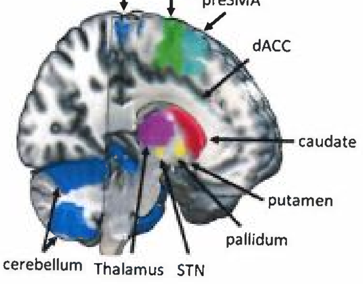

M1	primary motor cortex
SMA	supplementary motor area
pre-SMA	pre-supplementary motor area
dACC	dorsal anterior cingulate cortex
STN	subthalamic nucleus

3.1.3 Psychomotor slowing

One important domain of motor abnormalities in schizophrenia is psychomotor slowing (PS), which can be observed in fine motor behavior such as writing, gross motor behavior such as gait, and it may refer to single movements or continuous movement. PS includes movement planning, initiation, execution, timing and motor control [15, 23-25]. Typical examples of PS in schizophrenia are reduced levels of spontaneous gross motor activity as measured by actigraphy[11, 26-28], slowed gait[29], slowed aiming arm movements[30], slowing in fine motor tasks[26, 31, 32] or in bradykinesia of parkinsonism[31].

Multiple reports suggest that 30-50% of schizophrenia patients present with PS[33, 34]. Furthermore, PS is seen in all stages of the disorder. Even though, PS severity seems to increase with illness chronicity[35], it does also occur in early psychosis or in subjects at risk for psychosis[34, 36-40]. In psychosis, PS is critically linked to several disadvantages[23]. For example, PS is associated with poor cognition, sedentary behavior and cardiometabolic risks[41, 42]. Moreover, PS correlates with distress and poor quality of life[17, 43, 44]. Finally, several reports indicate that PS predicts poor outcome in terms of cognition, quality of life and real world functioning in early psychosis, although at baseline patients with PS were not specifically impaired in function[36, 45-47]. In sum, **PS is a frequently observed phenomenon across schizophrenia stages**, associated with poor quality of life, sedentary behavior and predictive of poor cognition and outcome. In order to design new treatment options to overcome this problematic symptom domain, it is essential to know the pathobiology of PS.

PS is particularly suitable for objective instrumental assessment. There are valid measures of fine motor and gross motor slowing that can be acquired by instrumentation[15]. These measures allow dimensional assessment of PS and are therefore ideal candidates when exploring associations between brain and behavior. Instrumentation is also not prone to conceptual overlap. We have applied wrist actigraphy in a series of studies and repeatedly demonstrated reduced gross motor activity in schizophrenia, which was linked to negative syndrome severity and psychomotor slowing as measured by the Salpêtrière Retardation Rating Scale (SRRS)[26, 48-50]. Likewise, finger tapping test as a measure of PS in fine motor behavior is also consistently poorer in schizophrenia patients and linked to negative symptoms[31]. In addition, the video rated coin rotation test is a simple and reliable measure of manual dexterity[51, 52]. Another interesting marker of motor behavior is increased postural sway, which results from poor cerebellar function and is increased in psychosis[53, 54]. Postural sway was correlated with negative syndrome severity in patients[53, 54]. Therefore, we expect increased postural sway to be associated with PS in schizophrenia. In sum, **PS can be reliably measured by observer ratings** such as the **SRRS** or by **instrumental measures** of gross motor behavior (**activity level** from actigraphy) or fine motor behavior (**finger tapping** and **coin rotation**).

3.1.4 Current treatment options of psychomotor slowing

As mentioned above, motor symptoms including PS are neither simply explained by antipsychotic drug effects[23] nor does PS disappear after antipsychotic drug withdrawal. An excellent study in treatment naïve first episode subjects demonstrated the heterogeneity of drug effects on motor abnormalities, with 30% of patients in whom parkinsonism or catatonia was ameliorated by antipsychotic drugs[55]. Likewise, in many studies of our own group and others we failed to detect a correlation between antipsychotic dosage and measures of PS[11, 26, 31, 49, 50]. However, symptoms tend to improve with treatment but from the naturalistic studies conducted it is currently unclear, whether the improvement is due to a direct effect on motor behavior or whether the benefit results from amelioration of other psychosis symptoms, such as disorganization or avolition[56, 57]. Our own study on the longitudinal course of PS in acute schizophrenia found an amelioration of PS with treatment, which was tightly linked to a decline of negative symptom scores[50]. Finally, there are no trials demonstrating a beneficial effect of antipsychotic medication or trainings to improve psychomotor slowing in schizophrenia. Thus, **alternative treatment options for PS are clearly needed.**

3.1.5 Psychomotor slowing and motor network dysfunction

Conceptually, various aspects of motor behavior are modulated by three key circuits: inhibition and

excitation of movements is related to a circuit including pre-/motor cortex and basal ganglia, timing of movements is linked to another circuit including motor cortex, thalamus and cerebellum, while psychomotor speed and planning involves a cortical network including medial prefrontal cortex, cingulate motor areas, SMA, M1, and posterior parietal cortex[12]. However, PS is not exclusively related to one of the abovementioned behaviors and probably involves all of the three circuits, even though the basal ganglia circuit is the most probable [22].

The current understanding of the pathology in PS is limited due to methodological issues, such as focus on single brain regions, single neuroimaging modalities, and heterogeneous patient samples[10, 22]. Two types of approaches have mainly been adopted: first, actigraphically assessed motor activity levels were correlated with structural and functional magnetic resonance imaging (MRI) markers in the brain. Results suggested that unlike in controls, motor activity levels are not associated with **structural connectivity within the basal ganglia loop** in schizophrenia. Instead, motor activity was linked to **cortical motor loops**, which was interpreted as compensatory mechanism because patients with PS (i.e. lower activity) had particularly lower CBF and reduced white matter integrity within these cortical motor loops[27, 58-60]. Likewise, white matter ultrastructure of the corpus callosum and cingulum mediated psychomotor speed in schizophrenia[61]. A second line of evidence stems from functional and structural MRI studies testing associations with finger movements such as finger tapping, sequential finger-thumb opposition, etc. These studies reported poorer tapping performance and reduced functional activation in SMA, M1, and cerebellum in schizophrenia[62]. Further evidence comes from the few studies on schizophrenia patients with catatonia, who usually present with behavioral motor inhibition. In a resting state perfusion MRI study, my group identified that catatonic schizophrenia is associated with specifically **increased cerebral blood flow (CBF)** in the supplementary motor area (**SMA**) and primary motor cortex (M1) in comparison to schizophrenia patients who had never experienced catatonia before. State CBF values were highest among those patients with severe motor inhibition. However, SMA perfusion was not different between state catatonia and controls[63]. The findings suggest a critical role of the SMA in movement initiation deficits in schizophrenia with state catatonia. However, from these data it was not possible to determine whether SMA hyperperfusion was the result of a motor network pathology that leads to inhibited motor output or whether SMA pathology drives this behavioral inhibition. Furthermore, finger tapping studies in catatonia patients indicated that M1 and SMA were less active during the task[64, 65]. Given the limitations of most previous studies focusing on one task or brain region, the next step must include **a network perspective to understand the pathobiology of motor inhibition as seen in PS**. A first cross-sectional study of my group applied resting state fMRI in order to test functional connectivity within the motor networks in schizophrenia. We found that schizophrenia was characterized by increased connectivity between key regions of the motor network, particularly between thalamus and motor/premotor cortex, M1 and cerebellum, cingulate seeds and STN[11]. Furthermore, the functional abnormalities between M1 and thalamus or cerebellum at rest were linked to observer ratings and instrumental measures of PS in schizophrenia. **Even though there is evidence suggesting that aberrant thalamocortical as well as cerebellar-cortical functional and structural connectivity may contribute to psychomotor slowing, the mechanism is still not entirely clear. This issue needs to be addressed at a network perspective and requires an exploration of the neural effects of interventions targeting psychomotor slowing.**

3.1.6 Intracortical excitability

Transcranial magnetic stimulation (TMS) allows for testing cortical neurophysiology in vivo. Converging evidence supports defective intracortical inhibition in M1 indexed by paired-pulse TMS in all stages of psychosis[66, 67]. Indeed, short-interval intracortical inhibition (SICI) is linked to GABAergic interneuron function. Reduced SICI values indicate **cortical disinhibition** in patients, which correlate with lower fractional anisotropy in motor tracts as well as lower processing speed[68]. Thus **psychomotor slowing** could be associated with **aberrant motor cortex excitability**, i.e. reduced SICI.

3.1.7 Brain stimulation for psychomotor slowing

Repetitive transcranial magnetic stimulation (rTMS) temporarily alters brain function in the targeted cortical areas. In addition, distant effects occur due to changes in network properties[69]. Depending on the frequency and type of stimulation, distinct effects on brain function are expected. Low-frequency rTMS (**lf-rTMS**) and continuous theta burst stimulation (cTBS) have **inhibitory** effects, while high-frequency rTMS (hf-rTMS) and intermittend theta burst (**iTBS**) have **facilitatory** effects. The preliminary knowledge of the pathobiology of PS suggests that rTMS could improve PS by changing aberrant motor network connectivity. Still, there are no published reports on the effects of non-invasive brain stimulation on PS. However, my group is conducting a randomized, double-blind, sham-controlled trial of rTMS for psychomotor slowing in major depressive disorder and schizophrenia (**NCT03275766**, clinicaltrials.gov). Treatment is based on rTMS in 4 arms: 1) facilitatory hf-rTMS over the left dorsolateral prefrontal cortex (DLPFC), 2) inhibitory lf-rTMS over the supplementary motor area (SMA), 3) facilitatory iTBS over the SMA, and 4) sham stimulation with a placebo coil. The primary outcome parameter is the percentage of responders (> 30% reduction in the Salpêtrière Retardation Rating Scale (SRRS) from baseline). Across the whole group of 34 randomized subjects (22 with schizophrenia and 12 with depression), there is a group difference in the number of responders after 15 rTMS sessions applying the last-observation-carried-forward method (Chi^2=11.3, df=3, p=.007) with **78% responders under lf-rTMS over SMA and no responder under iTBS over SMA**. When we exclusively focus on schizophrenia, this effect is also detected (Chi^2=6.6, df=3). Thus, lf-rTMS over SMA ameliorates PS.

The neurophysiologic effects of rTMS treatment can be probed by perfusion MRI for local effects on metabolism, by fMRI on the network level and by TMS paradigms testing changes of cortical excitability of M1. Studies on inhibitory rTMS over the left SMA indicated short-term alterations of functional connectivity from SMA to M1 in healthy subjects along with changes of local metabolic activity in focal dystonia patients and controls[70-72]. While we don't know whether similar effects would occur in patients with altered baseline functional connectivity in the motor network, we may expect to see neural changes (functional connectivity and regional perfusion) following rTMS treatment. Furthermore, single **inhibitory rTMS** for 15 mins over the premotor cortex led to **increased motor cortical excitability** in schizophrenia evidenced by reduced resting motor thresholds and increased motor evoked potentials, supporting our clinical findings of an amelioration of PS[73].

3.1.8 State of research summary

Motor abnormalities are a core feature of psychotic disorders, particularly in the schizophrenia spectrum, indicating poorer outcome[10, 15, 74]. Motor abnormalities are linked to alterations within the cerebral motor networks[22]. One important domain is psychomotor slowing (PS) that impacts gross and fine motor behavior. PS causes distress and functional disability. Moreover, PS can be reliably assessed by instrumentation[15]. But the underlying neurobiology of PS is not well understood. Prior work from correlational studies reported PS to be linked to aberrant functional and structural connectivity between M1 and thalamus or cerebellum[11, 60]. Particularly, patients with severe PS display hyperconnectivity and hyperperfusion in the premotor and motor cortices. Currently, no treatments specifically target PS in schizophrenia. But preliminary data from an rTMS study of my group indicate that inhibitory rTMS over the SMA alleviates PS. In addition, one session of inhibitory rTMS over SMA altered motor cortex excitability in psychosis[73]. **Given, that the motor circuitry is associated with PS and that inhibitory rTMS over SMA may improve PS in schizophrenia, we expect that inhibitory rTMS alters the relevant network for PS in patients**. The combination of resting state fMRI, perfusion MRI, diffusion MRI and rTMS is particularly suited to explore the neural changes in the motor networks[72]. Therefore, we will conduct a prospective RCT of rTMS for PS with neuroimaging assessments at baseline and week 3, focusing on the motor network. Furthermore, we will explore the effects of rTMS on cortical excitability, clinical and functional outcome. This is the first project to enable causal inferences on the pathobiology of PS and further supporting the use of effective rTMS treatment in schizophrenia by **proof of principle**.

3.2 Investigational Product (treatment, device) and Indication

TMS device: MagPro 30 with theta burst option

Manufacturer: MagVenture, Inc. Atlanta GA, USA

Device is intended for a broad range of neuroscientific research in humans.

There is CE conformity according to the ISO Norm 13485:2003.

3.3 Preclinical Evidence

Low-frequency rTMS (1Hz) has been tested in many studies of human neurophysiology, typically as single session rTMS with transient behavioral effects. Lf-rTMS over the SMA alters functional connectivity in the motor system in healthy subjects[72]. Likewise, lf-rTMS over M1 demonstrated increased local neural activity with increasing stimulus intensity in healthy controls, in addition to distant changes in neural activity within M1 connections[75]. Finally, the cortical excitability is altered in healthy subjects following lf-rTMS of M1[76].

3.4 Clinical Evidence to Date

rTMS treatment for psychomotor slowing has only been tested in Parkinson's disease[77, 78] and in a combined group of patients with major depression and schizophrenia (Walther et al. unpublished data). In our own randomized double-blind placebo-controlled trial, 15 sessions of 1 Hz rTMS were effective in ameliorating psychomotor slowing (see also Background). The active comparator iTBS was not effective, but also well-tolerated. There were no exceptional side-effects beyond those regularly reported in studies of rTMS.

The intended protocols (1Hz rTMS and iTBS as active control) have been widely used in neuropsychiatric cohorts to treat various symptoms[2]. Low-frequency rTMS (1Hz) is effective in reducing the severity of auditory verbal hallucinations in schizophrenia spectrum disorders[79-86]. There have been numerous reports demonstrating safety and efficacy of this protocol. The active comparator iTBS has been tested in studies in major depressive disorder and is effective in reducing depression severity with minimal side effects[87, 88]; most commonly transient headaches[2]. rTMS treatment has been applied between 10 and 30 sessions, usually 5 per week. There are international guidelines on the use of rTMS for clinical purposes, including safety measures and side effect assessments[1, 2].

3.5 Dose Rationale / Medical Device: Rationale for the intended purpose in study (pre-market MD)

As outlined in sections 3.1.7., 3.1.8., 3.3., and 3.4. rTMS has been used to modify psychomotor slowing in Parkinson's disease, depression, and schizophrenia. In addition, rTMS over the premotor and motor cortex alters brain function. Our previous study demonstrated clinical effectiveness of 1 Hz stimulation over left SMA on psychomotor slowing in 15 sessions. There are no other treatment studies on motor function in schizophrenia. However, studies applying less than 10 stimulation sessions to treat hallucinations were less effective in schizophrenia spectrum disorders[82, 89]. In major depression, rTMS studies with similar protocols usually treat patients for 4-6 weeks (20-30 sessions)[87].

3.6 Explanation for choice of comparator (or placebo)

This study will have two comparators: one placebo stimulation and an active stimulation that produces the opposite effect of the investigated protocol, i.e. iTBS over SMA which will facilitate neural activity. Both comparators are necessary to demonstrate clinical utility (placebo) and the neurophysiological changes associated with the three treatments (placebo and iTBS). Only this approach will finally allow testing whether inhibitory lf-rTMS is changing the motor system in a specific direction that is causal for clinically relevant changes of motor behavior. The waiting list will disentangle specific stimulation effects from effects of time or expectation. Placebo will disentangle specific rTMS treatment effects. Finally, iTBS will disentangle the direction of neurophysiological changes of the motor system. All stimulations will target the left SMA. Mode of application, localization, frequency of sessions and apparatus will be identical for all protocol arms. Arms will differ in the coil used (real or sham) and in the frequency of the applied stimulation (1 Hz vs. iTBS). The number of pulses is similar in all protocols (1'000-1'200).

3.7 Risks / Benefits

This study will follow the general recommendations for safety in rTMS protocols[1, 2] and the device manual. In addition, a screening standard questionnaire for rTMS candiates will be applied to ensure

that all strict exclusion criteria for safety in the protocol will be respected. The expected main adverse effects could be transient headache, transient local pain, transient neck pain, transient toothache and transient paraesthesia. Transient hearing changes have not been reported applying theta burst TMS but are likely possible. Therefore hearing protection will be used (earplugs). Furthermore we will make prompt referral for auditory assessment in case of any individual who complains of hearing loss, tinnitus or aural fullness following the rTMS. Seizure induction is rare but possible in epilepsy patients which are therefore excluded. Furthermore, in some patients transient impairment of working memory was reported. Taken together, if the safety regulations are followed, the risk for participants is minimal. All adverse events are only temporarily occurring and no severe adverse events have been reported in rTMS trials up to now.

Magnetic fields attenuate rapidly with distance, so it seems unlikely that the fetus of a pregnant woman might be directly affected by rTMS. Currently, no side effects to the child were reported in reports of repeated rTMS in pregnant women[90, 91]. Still, pregnant women will be excluded from this study.

Participation in the study does not pose a particular risk for patients, as the rTMS will be added on to the existing standard medical and non-medical interventions. It is likely that patients in the experimental group will experience better outcomes. However, most patients (in control arms) and healthy controls will not have any personal benefit from participation in the study. Still, the study will be an important step towards the design of specific trials to improve psychomotor slowing in schizophrenia. Given, that the experimental arm was superior in ameliorating psychomotor slowing, treatment will result in better outcomes and quality of lives. In addition, results of this study will be used to plan further interventional trials in patients with schizophrenia, which may then introduce rTMS as a standard add-on treatment of psychomotor slowing in schizophrenia spectrum disorders.

3.8 Justification of choice of study population

This study will include a group of patients with schizophrenia spectrum disorders, who will receive the assessments and interventions. In addition, a group of healthy control subjects is included only for the assessments, but not to receive an intervention. Patients will be included if they can provide consent on their own (no minors and no patients incapable of understanding the study information will be recruited). Participation in the study does not interfere with regular treatment. Patients' interests will be safeguarded by the treating physicians who are not part of the study team.

Study of patients is necessary in order to test the rTMS effect in a group with clear psychomotor slowing. These symptoms are only found in patients with severe psychiatric disorders.

Study of controls is needed in order to compare motor behaviour abnormalities and alterations in brain network structure and function between health and disease. Controls will not receive any intervention.

4. STUDY OBJECTIVES

4.1 Overall Objective

This study investigates the neural correlates of psychomotor slowing in schizophrenia spectrum disorders and the neurophysiologic changes associated with successful application of rTMS to ameliorate slowing. To reach this aim, the study combines a randomized, double-blind, placebo-controlled trial of rTMS in patients over 3 weeks and a cross-sectional and longitudinal study focussing on motor behaviour and brain imaging. Therefore, this study has four main aims. First, we aim to **investigate the clinical and functional neural changes following 3 weeks of daily rTMS treatment** (inhibitory, facilitatory, or placebo). An exploratory aim is to describe imaging markers of treatment response. Second, we aim to **characterize the pathophysiology of psychomotor slowing** (PS) in depth combining multimodal neuroimaging and electrophysiology with instrumental and observer-based measures of PS. Third, we aim **to characterize short-term changes of PS** by observing a waiting list cohort that is later allocated to treatment. And fourth, we aim to **describe the clinical outcome** of the rTMS intervention at 6-month follow-up.

4.2 Primary Objective

The study seeks to determine the clinical and neural effects of 15 sessions of inhibitory lf-rTMS over SMA on psychomotor slowing in schizophrenia spectrum disorders compared to facilitatory iTBS, placebo or patients on a waiting list.

4.3 Secondary Objectives

The study further seeks to determine, whether lf-rTMS (inhibitory) will be superior over placebo or iTBS (facilitatory) in all clinical and motor behavioral measures, e.g. greater reduction in BFCRS scores from baseline, greater increase in activity levels, or finger taps. In addition, the study will test the effects of rTMS on the cerebral motor network connectivity and activity, as well as on cerebellar function and motor cortex excitability. Furthermore, the study will explore the neuroimaging markers of response to treatment. Moreover, the study will test the association between measures of PS and neuroimaging markers of the motor system, e.g. network connectivity, activity, and structure. Next, the study will investigate the temporal dynamics of PS by comparing a waiting list group to the placebo group. Finally, the study will test whether lf-rTMS treatment for 3 weeks will have lasting effects on PS at week 6 or 6-months follow-up.

4.4 Safety Objectives

The study will also assess the tolerability of 15 of sessions rTMS in terms of stimulation side effects and duration of stimulation effects.

5. STUDY OUTCOMES

5.1 Primary Outcome

The primary endpoint will be the change in psychomotor slowing from baseline to week 3 measured with SRRS scores[92]. The SRRS is administered at baseline, week 1, week 2, week 3, week 6, and week 24. It is an observer rated scale quantifying the severity of psychomotor slowing with 15 items, each ranging 0-4. The SRRS has been developed to target this behavior and has been used in previous trials. Assessments will be performed by blinded raters, who have been trained to use the scale reliably.

5.2 Secondary Outcomes

5.2.1 Clinical outcomes

Proportion of responders (>= 30% reduction from baseline SRRS) between groups at week 1, 2, 3, 6, and 24. This will provide an additional categorical measure of who benefits from the intervention in terms of the main target (psychomotor slowing).

Change in other commonly observed motor symptoms from baseline, including rating scales on

abnormal involuntary movements, Parkinsonism, and catatonia at week 3, 6, and 24. See section 9 for details on instruments.

Change in general psychopathology using the positive and negative syndrome scale PANSS[93] and the brief negative symptom scale BNSS[94] between baseline and week 3, week 6, and week 24.

Change in self-reported physical activity and experienced negative symptoms using the International Physical Activity Questionnaire (IPAQ)[95] and the self-evaluation of negative symptoms (SNS)[96].

5.2.2 Behavioral outcomes

Change in objective gross motor activity as measured with 24 hours of wrist actigraphy at baseline, week 3, 6, and 24.

Change in fine motor function as measured with the coin rotation task at baseline, week 3, 6, and 24.

5.2.3 Physiological outcomes

Changes in motor cortex excitability from baseline to week 3, 6, and 24 using a TMS paradigm of short interval intracortical inhibition (SICI), intracortical facilitation (ICF) and 1 mV motor evoked potentials (MEP).

Changes in postural sway from baseline at week 3, using the Kistler platform and assessments with eyes open as well as eyes closed.

5.2.4 Neuroimaging outcomes

Change in resting state perfusion within the motor network from baseline to week 3.

Change in motor network resting state connectivity from baseline to week 3.

Change in neural activation patterns during a finger tapping task using functional MRI at baseline and week 3.

5.3 Other Outcomes of Interest

Change in social and community functioning using the global assessment of functioning GAF[97], the Social and Occupational Functioning SOFAS[98], and the UPSA-brief[99] short assessment of functional capacity at baseline, week 6, and week 24. Social and community functioning is a relevant distal outcome for any psychiatric intervention.

5.4 Safety Outcomes

After each rTMS session, participants are inquired about stimulation side effects. After sessions 5, 10 and 15, we will apply a short rating scale to assess side effects according to the guidelines[1, 2].

6. STUDY DESIGN

6.1 General study design and justification of design

The design of this single-site study is a 4 parallel-arm, double-blind, randomized, placebo-controlled trial. Patients will be randomized to one of four treatment arms. Patients will all receive TMS over the left SMA in 15 daily sessions over 3 weeks as add-on treament. In three groups, assessments will be conducted immediately before and after the three weeks treatment (1 Hz, iTBS, and Placebo), while the fourth group will receive active treatment (1Hz) only after a 3 week waiting period with no additional intervention. After 6 months we will perform a clinical follow-up assessment. The interventions will be conducted by a small team (2 persons) who will know the stimulation parameters. All assessments will be conducted by a different team, who is completely blind to treatment and specifically trained to use the instruments and rating scales. Patients are also blind to treatment, because stimulation site is the same for all groups and there will be no change of protocols during the study. The placebo group will be stimulated with a placebo coil that looks identical and emits identical noises compared to the real TMS coil, but without any magnetic impulse emission. Assessments with clinical rating scales will be complemented by objective and instrumental motor tests that are not prone to rater bias. Therefore, both the patients and the assessors are blind to treatment protocol.

The study will include patients with schizophrenia spectrum disorders who currently have psychomotor slowing according to the SRRS. The protocol arms are chosen to test the clinical efficacy and neurophysiological changes of 15 sessions 1 Hz rTMS for psychomotor slowing in schizophrenia spectrum disorders. The placebo-arm is required to demonstrate an effect of rTMS as add-on treatment. The active control is required to demonstrate an opposite effect at the neural level compared to active treatment. Finally, the waiting list is important to characterize potential self-limitation of the condition under study. Because these subjects are referred to a waiting list, they will receive active treatment thereafter. This group will not be completely blinded, as they will know to be in the active treatment group from week 3 through week 6.

We intend to enrol 88 patients with schizophrenia spectrum disorders. For the neuroimaging and motor behavioural assessments, we also plan to include 40 matched healthy control subjects.

For each patient in group 1-3 the study is 3 weeks and a follow-up interview after 6 months. Patients in group 4 (waiting list) will have three assessments (baseline, week 3 and week 6) and a follow-up interview at 6 months. The total duration of the study is 4 years.

6.2 Methods of minimising bias

6.2.1 Randomisation

Using the free software research randomizer, we will generate for the patients a list with four numbers indicating the group allocation, i.e. stimulation types. Allocation will be 1:1:1:1 across the whole intended sample. Subsequent numbers of study inclusion will therefore determine to which study arm the patient will be randomized. The lists will only be available for the principal investigator and locked in his office. He will perform randomization and give the written group allocation to the investigators.

6.2.2 Blinding procedures

As described in 6.1, patients will receive 15 sessions of rTMS over the left SMA in a blinded fashion. They will not be able to see which protocol is used (coil placement on top of their head, machine display behind the patients). Because of the parallel arm design, they will not know how the other protocols feel. Patients will be eye-blinded and ear-plugged during rTMS.

The teams performing assessments and rTMS stimulations will be strictly distinct. Therefore, patients and outcome assessors will be blinded to treatment arm.

6.2.3 Other methods of minimising bias

Outcomes are assessed with instrumental means or validated questionnaires. All assessments will follow a standard routine. Assessors will be trained to use instruments by the PI.

6.3 Unblinding Procedures (Code break)

In case of severe adverse events, an unblinding can take place at the responsibility of the principal investigator. In this case, the participant will not be able to further participate in the study. The allocated intervention order will be kept in a sealed envelope. The sealed envelopes will be stored at a central place, i.e. the office of the lab, so they can be accessed in case of any emergency.

7. STUDY POPULATION

The aim is to test 88 subjects with schizophrenia spectrum disorders and 40 healthy control subjects. Both genders will be included, age range 18 – 65 years.

7.1 Eligibility criteria

Participants fulfilling all of the following **<u>inclusion</u>** criteria are eligible for the study:

- ages 18–60 years
- Right-handed subjects
- Ability and willingness to participate in the study
- Ability to provide written informed consent
- Informed Consent as documented by signature (Appendix Informed Consent Form)

- **Patients only**: schizophrenia spectrum disorders according to DSM-5 **with** psychomotor slowing (SRRS score ≥ 15)

The presence of any one of the following **exclusion** criteria will lead to exclusion of the participant:

- Substance abuse or dependence other than nicotine
- Past or current medical or neurological condition associated with impaired or aberrant movement, such as dystonia, idiopathic parkinsonism or stroke.
- History of head trauma with concurrent loss of consciousness.
- Epilepsy
- History of any hearing problems or ringing in the ears
- Standard exclusion criteria for MRI scanning and TMS; e.g. metal implants, claustrophobia
- Patients only: any TMS treatment in the past 3 months
- Women who are pregnant or breast feeding,
- Intention to become pregnant during the course of the study,
- Female participants who are surgically sterilised / hysterectomised or post-menopausal for longer than 2 years are not considered as being of child bearing potential.
- Previous enrolment into the current study,
- Enrolment of the investigator, his/her family members, employees and other dependent persons
- **Controls only**: history of any psychiatric disorder or first-degree relatives with schizophrenia spectrum disorders.

7.2 Recruitment and screening

Healthy participants will be recruited by word-of-mouth, an internet link at the homepage of the department and flyers at supermarkets or at the University of Bern. Staff of the University Hospital of Psychiatry Bern will not be recruited. Furthermore, patients will be asked for participation at the inpatient and outpatient departments of the University Hospital of Psychiatry, Bern. All participants will spend approximately a total of 18 hours in the study on 7 different assessment days (screening, baseline, 3x during interventions and 2 follow-ups). Thus, they will be compensated by a single payment of CHF 200,-. Screening is described in section 9.1 and performed by master-level psychologists or psychiatrists.

7.3 Assignment to study groups

Randomization procedure is described in section 6.2.1. When the investigators have recruited a patient-participant, the principal investigator will allocate the person to a study group based on the randomization list and the sequence of enrolment in the study. From the list of numbers, patients are sequentially allocated by the principal investigator to one of the four treatment arms. The principal investigator will provide written information to the investigators concerning the treatment arm. The lists with the group allocations are only accessible for the principal investigator.

7.4 Criteria for withdrawal / discontinuation of participants

Participants may discontinue the trial at any time or withdraw consent. Furthermore, the treating physicians may request study discontinuation in case of significant deterioration of the condition at any time during the intervention period. All data from subjects in the intent-to-treat population, who received at least one dose of rTMS will be further used in the analyses. We will apply the last-observation-carried-forward (LOCF) method in the final analyses. In case a patient withdraws consent before the first rTMS stimulation, we will recruit an additional patient. The number of all patients recruited and randomized will be reported in the publications. All data from neuroimaging acquisition and motor behaviour will be used whenever possible, e.g. for analyses of baseline associations between brain imaging and behavior. In case of withdrawal due to adverse events or serious adverse events there will be follow-up examinations after 14 days and repeatedly until the problem is resolved, see 9.2.5.

8. STUDY INTERVENTION

8.1 Identity of Investigational Products (treatment / medical device)

8.1.1 Experimental Intervention (treatment / medical device)

Name: MagPro R30 with theta burst option

Manufacturer: MagVentrue, Inc. Atlanta GA, USA

The device is CE certified according to ISO-Norm 13485:2003 and approved for clinical use.

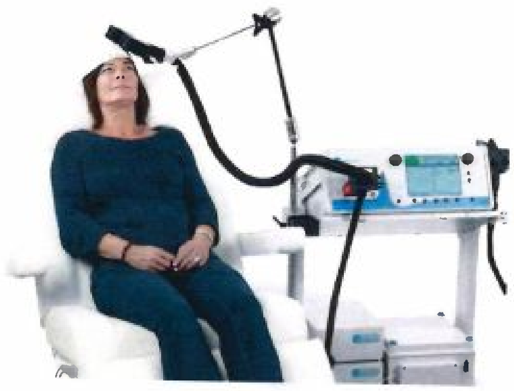

Active stimulation: 1 Hz stimulation of 17 mins over left SMA, 15 sessions in total (5 per week)

8.1.2 Control Intervention (standard/routine/comparator treatment / medical device)

All stimulations are delivered with the same device. The stimulation sites are the same (left SMA) for all groups. The iTBS protocol is shorter (3 mins), the placebo protocol will be delivered with a specific placebo-coil that looks identical, makes identical sounds but produces no magnetic pulses.

Placebo control: 1 Hz stimulation of 17 mins over left SMA without magnetic emission, 15 sessions in total (5 per week)

Active control: iTBS stimulation over left SMA, 21 mins per session, 15 sessions in total (5 per week)

Waiting list: no intervention during the first three weeks, afterwards active stimulation: 1 Hz stimulation of 17 mins over left SMA, 15 sessions in total (5 per week)

8.1.3 Packaging, Labelling and Supply (re-supply)

Not applicable.

8.1.4 Storage Conditions

TMS devices are stored in the institution (translational research center at the University Hospital of Psychiatry, Bern) according to internal regulations and the device manual.

8.2 Administration of experimental and control interventions

8.2.1 Experimental Intervention

The application of the TMS device follows the published guidelines[1, 2] and the device manual. Before each session, the resting motor threshold is determined to identify the individual intensity of stimulation. The position for the coil placement is left SMA, which is determined either via neuronavigation using individual brain anatomy or 3 cm anterior of the leg motor area, which is individually determined through single pulse stimulations causing leg movements.

Both experimenter and participants will wear earplugs for auditory safety.

Active experimental protocol:

Lf-rTMS at 1Hz will be used with 1'000 pulses at an intensity of 110% of the resting motor threshold (approximate duration 17 minutes). The protocol is identical to that of our previous study and a study in Parkinson's disease[77]. 15 sessions in total (5 per week)

8.2.2 Control Intervention

Placebo control: 1 Hz stimulation of 17 mins over left SMA without magnetic emission, using the Placebo-coil. 15 sessions in total (5 per week)

Active control: Stimulation parameters for iTBS will follow those of Huang et al.[100], including 600 pulses at 50 Hz (stimulation in 2 sec trains every 10 sec for 190 sec in total). To increase the effect, we will deliver two iTBS series in one session separated by 15 min with a total of 1200 pulses. During the experiment, iTBS pulse intensity is adjusted to 80% of the motor threshold. iTBS stimulation of 21 mins over left SMA, 15 sessions in total (5 per week)

Waiting list: same protocol as active experimental protocol but only between week 3 and 6.

8.3 Dose / Device modifications

In case of intolerable side effects, the study will be discontinued for the participant. No dose or device modifications are planned.

8.4 Compliance with study intervention

Investigators have to document the order of stimulation. This will be checked for consistency with the group allocation. No further strategies are needed. Participants are always under observation during the rTMS sessions and the planned assessments. Non-compliance on the side of the participant would lead to study discontinuation for this person. Use of concomitant medication will be retrieved from the medical files of the patients and transferred to the CRF.

8.5 Data Collection and Follow-up for withdrawn participants

If participants withdraw their consent, they will be contacted immediately to clarify whether there were intolerance issues with the study. See section 9.2.5 for safety follow-up measures. In case of withdrawal the data cannot be anonymized, but will be kept encrypted as all the data of the other participants. Patients who withdrew consent will be invited for the 6 month follow-up interview using their preferred way of contact (Email, letter).

8.6 Trial specific preventive measures

No specific preventive measures are needed.

8.7 Concomitant Interventions (treatments)

Current medication will be recorded in the CRF at study entry and throughout the 3 week intervention period. Patients are expected to comply with the treatment they are receiving from their treating physicians.

Because this is an add-on treatment, we expect relevant changes from concomitant interventions. Therefore, we apply placebo, waiting-list, and an active control group.

8.8 Study Drug / Medical Device Accountability

Not applicable.

8.9 Return or Destruction of Study Drug / Medical Device

The TMS devices are already in use in the clinic. No study specific procedures are intended.

9. STUDY ASSESSMENTS

9.1 Study flow chart(s) / table of study procedures and assessments

Flow charts are distinct for controls and patients, because controls receive no intervention. Furthermore, three patient groups will start interventions immediately (lf-rTMS, iTBS, and Placebo; i.e. groups 1-3), but one group will be waiting for 3 weeks and then enter the intervention phase (waiting group; i.e. group 4).

9.1.1 Patients of treatment groups 1-3

Study Periods	Screening	Treatment, Intervention Period				Follow-up	
Visit	1	2	3	4	5	6	7
Time (week)	-1	0	1	2	3	6	24
Patient Information and Informed Consent	x						
Demographics	x						
CASH and SCID (history)	x						
In- /Exclusion Criteria	x						
Physical Examination	x						
Pregnancy Test	x						
Psychopathology (TALD, NES)		x					
Randomisation		x					
Administer rTMS (5 sessions per week)		x	x	x			
Primary Variable SRRS		x	x	x	x	x	x
Motor scales UPDRS, BFCRS, AIMS, IPAQ		x			x	x	x
Psychopathology (PANSS, BNSS, SNS)		x			x	x	x
Actigraphy and coin rotation		x			x	x	x
Posturography		x			x		
TMS cortical excitability		x			x	x	x
Cerebral MRI		x			x		
Functional outcome (SOFAS, GAF, UPSA-brief)		x				x	x
Concomitant Therapy		x	x	x	x		
Adverse Events		x	x	x	x		

Study events patient groups 1-3

Visit	Information	Instrument used	Expected time
1 (Screening)	Check inclusion / exclusion criteria	CRF	5 min
	Demographic data	CRF	5 min
	Medical history	CRF	10 min
	Handedness	Edinburgh Handedness Inventory[101]	5 min
	Pregnancy test	Urine based test	10 min
	Diagnoses ascertainment	SCID interview	60 min
2 (baseline)	Psychopathology	Positive And Negative Syndrome Scale (PANSS)[93] interview, CASH[102] interview, Brief Negative Symptom Scale (BNSS)[94],	90 min

		Thought and Language Disorder (TALD)[103], Neurological Evaluation Scale (NES)[104]	
	Primary outcome	SRRS[92]	5 min
	Motor scales	Unified Parkinson's Disease Rating Scale (UPDRS)[105], Bush Francis Catatonia Rating Scale (BFCRS)[106], Abnormal Involuntary Movement Scale (AIMS)[107]	15 min
	Self Report	Self-Evaluation of negative symptoms (SNS)[96], International Physical Actitvity Questionnaire (IPAQ)[95]	10 min
	Motor tests	Actigraphy and coin rotation	5 min
	Posturography	Kistler platform at Dept. of Neurology	30 min
	Cortical excitability	TMS paradigm with paired pulse stimulation	30 min
	Neuroimaging	MRI scan including structural T1, Arterial spin labelling, BOLD resting state and task fMRI	60 min
	Functional outcome	Global Assessment of Functioning GAF, Social and Occupational Functioning Scale SOFAS, UPSA-brief assessment of functional capacity	15 min
	rTMS	5 rTMS daily sessions à 20 min	100 min
3 (week 1)	Primary outcome	SRRS	5 min
	rTMS	5 rTMS daily sessions à 20 min	100 min
	Check side effects	CRF	5 min
	Concomitant therapy	CRF	5 min
4 (week 2)	Primary outcome	SRRS	5 min
	rTMS	5 rTMS daily sessions à 20 min	100 min
	Check side effects	CRF	5 min
	Concomitant therapy	CRF	5 min
5 (week 3)	Psychopathology	Positive And Negative Syndrome Scale (PANSS)[93] interview, Brief Negative Symptom Scale (BNSS)[94]	30 min
	Primary outcome	SRRS[92]	5 min
	Motor scales	Unified Parkinson's Disease Rating Scale (UPDRS)[105], Bush Francis Catatonia Rating Scale (BFCRS)[106], Abnormal Involuntary Movement Scale (AIMS)[107]	15 min
	Self Report	Self-Evaluation of negative symptoms (SNS)[96], International Physical Actitvity Questionnaire (IPAQ)[95]	10 min
	Motor tests	Actigraphy and coin rotation	5 min

	Posturography	Kistler platform at Dept. of Neurology	30 min
	Cortical excitability	TMS paradigm with paired pulse stimulation	30 min
	Neuroimaging	MRI scan including structural T1, Arterial spin labelling, BOLD resting state and task fMRI	60 min
	Functional outcome	Global Assessment of Functioning GAF, Social and Occupational Functioning Scale SOFAS, UPSA-brief assessment of functional capacity	15 min
	Check side effects	CRF	5 min
	Concomitant therapy	CRF	5 min
6 (week 6)	Psychopathology	Positive And Negative Syndrome Scale (PANSS)[93] interview, Brief Negative Symptom Scale (BNSS)[94]	30 min
	Primary outcome	SRRS[92]	5 min
	Motor scales	Unified Parkinson's Disease Rating Scale (UPDRS)[105], Bush Francis Catatonia Rating Scale (BFCRS)[106], Abnormal Involuntary Movement Scale (AIMS)[107]	15 min
	Self Report	Self-Evaluation of negative symptoms (SNS)[96], International Physical Actitvity Questionnaire (IPAQ)[95]	10 min
	Motor tests	Actigraphy and coin rotation	5 min
	Cortical excitability	TMS paradigm with paired pulse stimulation	30 min
	Functional outcome	Global Assessment of Functioning GAF, Social and Occupational Functioning Scale SOFAS, UPSA-brief assessment of functional capacity	15 min
7 (week 24)	Psychopathology	Positive And Negative Syndrome Scale (PANSS)[93] interview, Brief Negative Symptom Scale (BNSS)[94]	30 min
	Primary outcome	SRRS[92]	5 min
	Motor scales	Unified Parkinson's Disease Rating Scale (UPDRS)[105], Bush Francis Catatonia Rating Scale (BFCRS)[106], Abnormal Involuntary Movement Scale (AIMS)[107]	15 min
	Self Report	Self-Evaluation of negative symptoms (SNS)[96], International Physical Actitvity Questionnaire (IPAQ)[95]	10 min
	Motor tests	Actigraphy and coin rotation	5 min
	Cortical excitability	TMS paradigm with paired pulse stimulation	30 min
	Functional outcome	Global Assessment of Functioning GAF, Social and Occupational Functioning	15 min

		Scale SOFAS, UPSA-brief assessment of functional capacity	

9.1.2 Patients of treatment group 4 (waiting group)

Study Periods	Screening	Treatment, Intervention Period					Follow-up	
Visit	1	2	3	4	5	6	7	8
Time (week)	-1	0	3	4	5	6	9	24
Patient Information and Informed Consent	x							
Demographics	x							
CASH and SCID (history)	x							
In- /Exclusion Criteria	x							
Physical Examination	x							
Pregnancy Test	x							
Psychopathology (TALD, NES)		x						
Randomisation		x						
Administer rTMS (5 sessions per week)			x	x	x			
Primary Variable SRRS		x	x	x	x	x	x	x
Motor scales UPDRS, BFCRS, AIMS, IPAQ		x	x			x	x	x
Psychopathology (PANSS, BNSS, SNS)		x	x			x	x	x
Actigraphy and coin rotation		x	x			x	x	x
Posturography		x	x			x		
TMS cortical excitability		x	x			x	x	x
Cerebral MRI		x	x			x		
Functional outcome (SOFAS, GAF, UPSA-brief)		x	x				x	x
Concomitant Therapy		x	x	x	x	x		
Adverse Events			x	x	x	x		

Study events for patients of the waiting group

Visit	Information	Instrument used	Expected time
1 (Screening)	Check inclusion / exclusion criteria	CRF	5 min
	Demographic data	CRF	5 min
	Medical history	CRF	10 min
	Handedness	Edinburgh Handedness Inventory[101]	5 min
	Pregnancy test	Urine based test	10 min
	Diagnoses ascertainment	SCID interview	60 min
2 (baseline)	Psychopathology	Positive And Negative Syndrome Scale (PANSS)[93] interview, CASH[102] interview, Brief Negative Symptom Scale (BNSS)[94], Thought and Language Disorder (TALD)[103], Neurological Evaluation Scale (NES)[104]	90 min

	Primary outcome	SRRS[92]	5 min
	Motor scales	Unified Parkinson's Disease Rating Scale (UPDRS)[105], Bush Francis Catatonia Rating Scale (BFCRS)[106], Abnormal Involuntary Movement Scale (AIMS)[107]	15 min
	Self Report	Self-Evaluation of negative symptoms (SNS)[96], International Physical Actitvity Questionnaire (IPAQ)[95]	10 min
	Motor tests	Actigraphy and coin rotation	5 min
	Posturography	Kistler platform at Dept. of Neurology	30 min
	Cortical excitability	TMS paradigm with paired pulse stimulation	30 min
	Neuroimaging	MRI scan including structural T1, Arterial spin labelling, BOLD resting state and task fMRI	60 min
	Functional outcome	Global Assessment of Functioning GAF, Social and Occupational Functioning Scale SOFAS, UPSA-brief assessment of functional capacity	15 min
3 (week 3)	Psychopathology	Positive And Negative Syndrome Scale (PANSS)[93] interview, CASH[102] interview present part only, Brief Negative Symptom Scale (BNSS)[94], Thought and Language Disorder (TALD)[103], Neurological Evaluation Scale (NES)[104]	90 min
	Primary outcome	SRRS[92]	5 min
	Motor scales	Unified Parkinson's Disease Rating Scale (UPDRS)[105], Bush Francis Catatonia Rating Scale (BFCRS)[106], Abnormal Involuntary Movement Scale (AIMS)[107]	15 min
	Self Report	Self-Evaluation of negative symptoms (SNS)[96], International Physical Actitvity Questionnaire (IPAQ)[95]	10 min
	Motor tests	Actigraphy and coin rotation	5 min
	Posturography	Kistler platform at Dept. of Neurology	30 min
	Cortical excitability	TMS paradigm with paired pulse stimulation	30 min
	Neuroimaging	MRI scan including structural T1, Arterial spin labelling, BOLD resting state and task fMRI	60 min
	Functional outcome	Global Assessment of Functioning GAF, Social and Occupational Functioning Scale SOFAS, UPSA-brief assessment of functional capacity	15 min
	rTMS	5 rTMS daily sessions à 20 min	100 min
4 (week 4)	Primary outcome	SRRS	5 min

	rTMS	5 rTMS daily sessions à 20 min	100 min
	Check side effects	CRF	5 min
	Concomitant therapy	CRF	5 min
5 (week 5)	Primary outcome	SRRS	5 min
	rTMS	5 rTMS daily sessions à 20 min	100 min
	Check side effects	CRF	5 min
	Concomitant therapy	CRF	5 min
6 (week 6)	Psychopathology	Positive And Negative Syndrome Scale (PANSS)[93] interview, Brief Negative Symptom Scale (BNSS)[94]	30 min
	Primary outcome	SRRS[92]	5 min
	Motor scales	Unified Parkinson's Disease Rating Scale (UPDRS)[105], Bush Francis Catatonia Rating Scale (BFCRS)[106], Abnormal Involuntary Movement Scale (AIMS)[107]	15 min
	Self Report	Self-Evaluation of negative symptoms (SNS)[96], International Physical Actitvity Questionnaire (IPAQ)[95]	10 min
	Motor tests	Actigraphy and coin rotation	5 min
	Posturography	Kistler platform at Dept. of Neurology	30 min
	Cortical excitability	TMS paradigm with paired pulse stimulation	30 min
	Neuroimaging	MRI scan including structural T1, Arterial spin labelling, BOLD resting state and task fMRI	60 min
	Functional outcome	Global Assessment of Functioning GAF, Social and Occupational Functioning Scale SOFAS, UPSA-brief assessment of functional capacity	15 min
	Check side effects	CRF	5 min
	Concomitant therapy	CRF	5 min
7 (week 9)	Psychopathology	Positive And Negative Syndrome Scale (PANSS)[93] interview, Brief Negative Symptom Scale (BNSS)[94]	30 min
	Primary outcome	SRRS[92]	5 min
	Motor scales	Unified Parkinson's Disease Rating Scale (UPDRS)[105], Bush Francis Catatonia Rating Scale (BFCRS)[106], Abnormal Involuntary Movement Scale (AIMS)[107]	15 min
	Self Report	Self-Evaluation of negative symptoms (SNS)[96], International Physical Actitvity Questionnaire (IPAQ)[95]	10 min

	Motor tests	Actigraphy and coin rotation	5 min
	Cortical excitability	TMS paradigm with paired pulse stimulation	30 min
	Functional outcome	Global Assessment of Functioning GAF, Social and Occupational Functioning Scale SOFAS, UPSA-brief assessment of functional capacity	15 min
8 (week 24)	Psychopathology	Positive And Negative Syndrome Scale (PANSS)[93] interview, Brief Negative Symptom Scale (BNSS)[94]	30 min
	Primary outcome	SRRS[92]	5 min
	Motor scales	Unified Parkinson's Disease Rating Scale (UPDRS)[105], Bush Francis Catatonia Rating Scale (BFCRS)[106], Abnormal Involuntary Movement Scale (AIMS)[107]	15 min
	Self Report	Self-Evaluation of negative symptoms (SNS)[96], International Physical Actitvity Questionnaire (IPAQ)[95]	10 min
	Motor tests	Actigraphy and coin rotation	5 min
	Cortical excitability	TMS paradigm with paired pulse stimulation	30 min
	Functional outcome	Global Assessment of Functioning GAF, Social and Occupational Functioning Scale SOFAS, UPSA-brief assessment of functional capacity	15 min

9.1.3 Control subjects

Study Periods	Screening	Observation period	
Visit<br>Time (week)	1<br>-1	2<br>0	3<br>3
Proband Information and Informed Consent	x		
Demographics	x		
SCID	x		
In- /Exclusion Criteria	x		
Physical Examination	x		
Pregnancy Test	x		
Primary Variable SRRS		x	x
Motor scales UPDRS, BFCRS, AIMS, IPAQ		x	x
Actigraphy and coin rotation		x	x
Posturography		x	x
TMS cortical excitability		x	x
Cerebral MRI		x	x

Study events for controls

Visit	Information	Instrument used	Expected time
1 (Screening)	Check inclusion / exclusion criteria	CRF	5 min
	Demographic data	CRF	5 min
	Medical history	CRF	10 min
	Handedness	Edinburgh Handedness Inventory[101]	5 min
	Pregnancy test	Urine based test	10 min
	Diagnoses ascertainment	SCID interview	60 min
2 (week 0)	Primary outcome	SRRS[92]	5 min
	Motor scales	Unified Parkinson's Disease Rating Scale (UPDRS)[105], Bush Francis Catatonia Rating Scale (BFCRS)[106], Abnormal Involuntary Movement Scale (AIMS)[107]	15 min
	Self Report	International Physical Actitvity Questionnaire (IPAQ)[95]	5 min
	Motor tests	Actigraphy and coin rotation	5 min
	Posturography	Kistler platform at Dept. of Neurology	30 min
	Cortical excitability	TMS paradigm with paired pulse stimulation	30 min
	Neuroimaging	MRI scan including structural T1, Arterial spin labelling, BOLD resting state and task fMRI	60 min
3 (week 3)	Primary outcome	SRRS[92]	5 min
	Motor scales	Unified Parkinson's Disease Rating Scale (UPDRS)[105], Bush Francis Catatonia Rating Scale (BFCRS)[106], Abnormal Involuntary Movement Scale (AIMS)[107]	15 min
	Self Report	International Physical Actitvity Questionnaire (IPAQ)[95]	5 min
	Motor tests	Actigraphy and coin rotation	5 min
	Posturography	Kistler platform at Dept. of Neurology	30 min
	Cortical excitability	TMS paradigm with paired pulse stimulation	30 min
	Neuroimaging	MRI scan including structural T1, Arterial spin labelling, BOLD resting state and task fMRI	60 min

9.2 Assessments of outcomes

Assessors of outcome variables will be psychologists or psychiatrists of master-degree-level. All assessors will be trained by the PI to use the instruments correctly and to assure interrater reliability. Weekly staff meetings will ensure conformity in procedures. All assessments will be conducted blind to

rTMS treatment.

9.2.1 Assessment of primary outcome

The Salpêtrière Retardation Rating Scale (SRRS) is applied at each visit from baseline to follow-up. The scale is described in section 5.1. Raters will be trained to use the scale and blind to rTMS treatment. The score of each of the 15 items is recorded in the CRF.

9.2.2 Assessment of secondary outcomes

9.2.2.1 *Clinical outcomes*

The change in motor syndromes from baseline is assessed at several visits (see 9.1). Trained raters blind to treatment will assess motor behaviour in standardized examinations with clinical rating scales. Parkinsonism is assess with the Unified Parkinson's Disease Rating Scale (UPDRS)[105], of which we will only assess part III, the current motor behaviour. Catatonia will be assessed with the Bush Francis Catatonia Rating Scale (BFCRS)[106], an 24 item rating scale specifically designed for this purpose. The timeframe of observation is the last week. Finally, dyskinesia will be monitored with the standard rating scale for this issue, the Abnormal Involuntary Movement Scale (AIMS)[107], a 7 item scale following a standardized examination. All motor rating scales are the gold standard of their kind.

The change of general schizophrenia symptoms is assessed with the Positive And Negative Syndrome Scale (PANSS)[93]. The 30 item scale is the widely used standard assessment following a standardized clinical interview. Change in negative symptoms is specifically assessed with the Brief Negative Symptom Scale (BNSS)[94], which allows monitoring relevant dimensions of negative symptoms such as apathy and diminished expression. Furthermore, we will apply the self-evaluation of negative symptoms (SNS)[96], a valid and reliable 20 items questionnaire to capture the subjective experience of negative symptoms.

9.2.2.2 *Behavioral outcomes*

Objective gross motor behaviour will be assessed using continuous wrist actigraphy for 24 hours. The actigraphs (empathica e4) will be worn on the wrist of the non-dominant arm. Data is stored as logged electronic file. This measure is sensitive to altered motor behaviour and has been successfully applied in many studies of Prof. Walther's team[27, 35, 49, 50, 108-111]. Patients will fill a sleep activity protocol to enable separation of sleep from wake periods during recording. This also allows to check data for consistency and plausibility, because measurements are continuously performed also in periods when participants are not observed.

The fine motor performance is assessed with the coin rotation task. In this task, participants are asked to rotate a .50 CHF coin between thumb, index and middle finger as fast as possible for a total of 30 seconds. The performance is recorded on video and later analysed offline. Analysis includes the number of half turns and the number of coin drops according to a validated formula[51, 52, 112].

Self-ratings of physical activity will be conducted with the 7-item International Physical Activity Questionnaire (IPAQ)[95], which has a German Version and has great psychometric properties. The ratings cover the past 7 days and allow calculating the energy expenditure and total activity.

9.2.2.3 *Physiological outcomes*

Measures of cortical excitability will be assessed as one of the important physiological outcome parameters. Measurements will be conducted with a MagPro R30 (MagVentrue, Inc. Atlanta GA, USA). Single pulse and paired pulse TMS protocols will be conducted to measure short-interval intracortical inhibition (SICI) at 1 msec interstimulus interval (ISI) and 3msec ISI, intracortical facilitation (ICF) at 7 msec ISI and 15 ms ISI, resting motor threshold (RMT), and 1 mV motor evoked potential (MEP), according to standard protocols[67, 113]

Posturography will be conducted at the Department of Neurology (Prof. Roger Kalla). The assessments of postural sway will be scheduled immediately before or after the MRI acquisition, because this is also located at the Inselspital Bern. Mean postural sway will be calculated as outcome variable for postural stability[53, 114]. The Kistler platform will be used to calculate of the pressure dependent fluctuation (x-, y-, z- axis) of the bodies' centre of gravity[115]. Participants will be measured standing with eyes open and eyes closed according to standard procedures.

9.2.2.4 *Neuroimaging outcomes*

Neuroimaging will be acquired twice, at baseline and week 3. MRI acquisition will be performed at a

3T Siemens Magnetom Trio scanner at the Institute of Diagnostic and Interventional Neuroradiology, Bern (local collaborator Prof. Dr. med. Roland Wiest). The 64-channel head coil will be used for all MRI images. First, high-resolution T1-weighted MR images will be obtained using a 3D magnetization-prepared rapid two-gradient-echo with 2 inversion times (MP2RAGE) sequence. fMRI will be performed using a multi-slice multi-band T2*-weighted echo planar imaging (EPI) sequence for resting state and during task execution. Prior to the fMRI images a B0 will be acquired for corrections of putative field inhomogeneity. Afterwards, we will perform acquisition of cerebral blood flow (CBF) at rest using a pseudo-continuous arterial spin labelling (pCASL) sequence. Moreover, we will acquire a M0 image (M0 equilibrium magnetization of water signal) for the quantification of CBF and a B0 for corrections of putative field inhomogeneity. Finally, a set of diffusion-weighted images (DWI) that will allow reconstructing fiber tracts using the model based on diffusion tensor imaging (DTI).

Anatomy (MP2RAGE) The optimized acquisition parameters were as follows: 176 sagittal slices, 256 × 224 matrix (with a non-cubic field of view (FOV) of 256 × 224 mm2, yielding a nominal isotropic resolution of 1 mm3), 5000 ms repetition time (TR), 2.98 ms echo time (TE), 700 ms and 2500 ms inversion time (TI), flip angle 4° and 5°, GRAPPA acceleration factor 3 and a 8:22 min total acquisition time.

B0-map - The 48 EPI interleaved axial oblique slices will be positioned exactly like the fMRI slices with exactly the same slice geometry. Two amplitude and a phase image will be recorded in each subject (TR = 520 ms, TE1 = 4.92 ms, TE2 = 7.38 ms). The acquisition time will last 1 min 40 sec.

fMRI - The 48 EPI interleaved axial oblique slices will be positioned in-line with the bi-commissural axis with the following parameters: TR = 500 ms, TE = 30 ms, FA = 90, slice thickness = 3.6 mm, gap thickness = 0 mm, matrix size = 64 x 64 mm, FOV 230 x 230 mm2 resulting in a iso-voxel dimension of 3.6 mm x 3.6 mm x 3.6 mm. The sequence is driven in a 3D PACE mode (Siemens Erlangen) to enable prospective motion correction. With these sequence parameters we will cover the whole brain including the cerebellum. In total, first 720 dynamic scans will be collected for resting state fMRI (total of 6 mins) and subsequently 1200 dynamic scans will be collected during the conduction of the task (total of 10 mins). The task will consist of four blocks including two active paced blocks, a passive listening block and rest. All blocks except rest will be paced with a continuous 2Hz auditory cue. The two active blocks are **right hand finger tapping** (index finger vs. thumb) and **sequential finger-thumb opposition** (all fingers of the right hand vs. thumb). These tasks have produced reliable activation of SMA, M1, basal ganglia, and cerebellum in healthy subjects and are easy enough to be performed by schizophrenia patients with PS[62, 64, 65, 116, 117]. During one run, we will alternate active and passive blocks, i.e. finger tapping – listening – sequential finger opposition – rest. Each block will last for 15 s and each run takes 1 min. We will alternate the active blocks between runs. A total of 10 runs will be conducted. Instructions will be presented on a video goggle system. Cue tones will be delivered via headphones. Participants will be instructed and trained on the task outside the scanner.

pCASL - The 30 EPI interleaved axial oblique slices will be positioned in-line with the bi-commissural axis and acquired in sequential order with the following parameters: TR = 3000 ms, TE = 12 ms, FA = 90, slice thickness = 8.0 mm, gap thickness = 0 mm, matrix size = 64 x 64 mm, FOV 256 x 256 mm2 resulting in a voxel dimension of 4.0 mm x 4.0 mm x 8.0 mm. the sequence will additionally have the following parameters: bolus duration = 700 ms, inversion time = 2200 ms. In total 100 images will be acquired lasting in total 6 min.

9.2.3 Assessment of other outcomes of interest

Measures of social and community functioning as described in section 5.3.

9.2.4 Assessment of safety outcomes

9.2.4.1 Adverse events

After every rTMS session, participants are asked about new occurrence of adverse events. As example, they are inquired about sensations associated with the stimulation or headaches. The answers are recorded in the CRF. Furthermore, at each study visit during the intervention phase, i.e. after 5 rTMS sessions, patients will be inquired about adverse events using a standard questionnaire in the CRF[2].

In case the adverse event is not limited to stimulation, the participants will be asked at the next session (24 hours later), whether the adverse event still continues or when it was resolved. The frequency and type of adverse events will be reported in the publication of results.

9.2.4.2 *Laboratory parameters*

No laboratory parameters will be taken.

9.2.4.3 *Vital signs*

No routine measurement of vital signs is planned. If patients report dizziness, clinical routines will include measurement of blood pressure and heart rate.

9.2.5 Assessments in participants who prematurely stop the study

Participants who prematurely stop the study will be immediately assessed for adverse events. In case of adverse events reports, a physical examination will be conducted and results will be recorded accordingly. Follow-up examinations will be planned within the next 14 days in case of continuing problem related to adverse events, and these examinations will be continued every 14 days until the health issue is resolved.

9.3 Procedures at each visit

9.3.1 Screening visit

- Check the inclusion and exclusion criteria, use CRF
- Collect demographic data, use CRF
- Conduct urine pregnancy test in female participants between 18 and 50 years of age
- Assess diagnoses with SCID, document results in CRF
- Assess handedness with EHI[101], document result in CRF
- Assess medical history in CRF

See table study events at 9.1

9.3.2 Visit 2 (baseline, week 0)

- Assess current psychopathology, use PANSS interview and BNSS, document in CRF
- Assess formal thought disorder, use TALD interview, document in CRF
- Assess Neurological soft signs, use NES, document in CRF
- Assess psychiatric history with CASH interview in patients, document result in CRF
- Assess primary outcome with SRRS scale, document in CRF
- Assess motor behaviour with rating scales, use BFCRS, UPDRS, AIMS, document results in CRF
- Assess functioning, use GAF, SOFAS, and UPSA-brief, document results in CRF
- Hand out self-rating questionnaires SNS and IPAQ, collect and document in CRF
- Conduct coin rotation test, record on video
- Record gross motor activity using actigraphy
- Test cortical excitability using the TMS device according to standard procedure. Record electronic data
- Acquire MRI scans at University Institute of Diagnostic and Interventional Neuroradiology
- Acquire posturography at University Hospital, Dept. of Neurology, record electronic data
- Record current medication regime in CRF
- Schedule 5 rTMS sessions
- Schedule next visit

9.3.3 Visit 3 and 4 (week 1 and 2)

- Assess primary outcome with SRRS scale, document in CRF
- Assess side effects with questions in the CRF
- Record current medication regime in CRF
- Schedule 5 rTMS sessions
- Schedule next visit

9.3.4 Visit 5 (end of intervention, week 3)

- Assess side effects with questions in the CRF

- Assess current psychopathology, use PANSS interview and BNSS, document in CRF
- Assess primary outcome with SRRS scale, document in CRF
- Assess motor behaviour with rating scales, use BFCRS, UPDRS, AIMS, document results in CRF
- Assess functioning, use GAF, SOFAS, and UPSA-brief, document results in CRF
- Hand out self-rating questionnaires SNS and IPAQ, collect and document in CRF
- Conduct coin rotation test, record on video
- Record gross motor activity using actigraphy
- Test cortical excitability using the TMS device according to standard procedure. Record electronic data
- Acquire MRI scans at University Institute of Diagnostic and Interventional Neuroradiology
- Acquire posturography at University Hospital, Dept. of Neurology, record electronic data
- Record current medication regime in CRF
- Schedule next visit (follow-up)

9.3.5 Visit 6 and 7 (follow-up, week 6 and 24)

- Assess current psychopathology, use PANSS interview and BNSS, document in CRF
- Assess primary outcome with SRRS scale, document in CRF
- Assess motor behaviour with rating scales, use BFCRS, UPDRS, AIMS, document results in CRF
- Assess functioning, use GAF, SOFAS, and UPSA-brief, document results in CRF
- Hand out self-rating questionnaires SNS and IPAQ, collect and document in CRF
- Conduct coin rotation test, record on video
- Record gross motor activity using actigraphy
- Test cortical excitability using the TMS device according to standard procedure. Record electronic data
- Record current medication regime in CRF
- Schedule next visit (follow-up)

10. SAFETY

10.1 Medical Device Category A studies

Device deficiencies and all **adverse events (AE)** including all **serious adverse events (SAE)** are collected, fully investigated and documented in the source document and appropriate case report form (CRF) during the entire study period, i.e. from patient's informed consent until the last protocol-specific procedure, including a safety follow-up period. Documentation includes dates of event, treatment, resolution, assessment of seriousness and causal relationship to device and/or study procedure [ISO 14155, 6.4.1.].

Safety is assessed spontaneously at each stimulation session and in a structured set of questions at each visit during the intervention phase (after 5 stimulations). See 9.2.4. If adverse events occur, patients are followed-up with extra-visits after 14 days until the issue has resolved.

Typical adverse events to be expected by rTMS are:

- Mild pain or nausea (39 %)
- Mild headaches (28%)
- Mild neck pain (40%)

Rare events include hearing problems or local skin irritation.

10.1.1 Definition and Assessment of safety related events

Adverse Event (AE)

Any untoward medical occurrence, unintended disease or injury or any untoward clinical signs (including an abnormal laboratory finding) in participants, users or other persons whether or not

related to the investigational medical device [ISO 14155: 3.2].

This includes events related to the investigational device or the comparator and to the procedures involved. For users or other persons this is restricted to events related to the investigational medical device.

Adverse Device Effect (ADE)

Adverse event related to the use of an investigational medical device [ISO 14155: 3.1].

This includes any adverse event resulting from insufficient or inadequate instructions for use, deployment, implantation, installation, operation, or any malfunction of the investigational medical device. This includes any event that is a result of a use error or intentional misuse.

Serious Adverse Event (SAE) [European regulation on medical devices 2017/745, art. 58].

Any adverse event that led to any of the following:

(a) death,
(b) serious deterioration in the health of the subject that resulted in any of the following:
(i) life-threatening illness or injury,
(ii) permanent impairment of a body structure or a body function,
(iii) hospitalisation or prolongation of patient hospitalisation,
(iv) medical or surgical intervention to prevent life-threatening illness or injury or permanent impairment to a body structure or a body function,
(v) chronic disease,
(c) foetal distress, foetal death or a congenital physical or mental impairment or birth defect.

This includes device deficiencies that might have led to a serious adverse event if a) suitable action had not been taken or b) intervention had not been made or c) if circumstances had been less fortunate. These are submitted to the EC via BASEC within 7 days. A planned hospitalisation for pre-existing condition, or a procedure required by the protocol, without a serious deterioration in health, is not considered to be a serious adverse event.

Device deficiency

Inadequacy of a medical device related to its identity, quality, durability, reliability, safety or performance, such as malfunction, misuse or use error and inadequate labelling [ISO 14155: 3.15].

Health hazards that require measures

Findings in the trial that may affect the safety of study participants and, which require preventive or corrective measures intended to protect the health and safety of study participants SAE [ClinO Art. 37].

Causal Relationship of SAE [MEDDEV 2.7/3 revision 3, May 2015].

A causal relationship towards the medical device or study procedure should be rated as follows:

- **Not related:** The relationship to the device or procedures can be excluded.
- **Unlikely:** The relationship with the use of the device seems not relevant and/or the event can be reasonably explained by another cause, but additional information may be obtained.
- **Possible:** The relationship with the use of the investigational device is weak but cannot be ruled out completely. Alternative causes are also possible.
- **Probable:** The relationship with the use of the investigational device seems relevant and/or the event cannot reasonably explained by another cause.
- **Causal relationship:** The serious event is associated with the investigational device or with procedures beyond reasonable doubt.

Device deficiencies that might have led to an SAE are always related to the medical device.

10.1.2 Reporting of (Serious) Adverse Events and other safety related events

Reporting to Sponsor-Investigator:

The following events are to be reported to the Sponsor-Investigator within 24 hours upon becoming aware of the event:

- All SAEs
- Health hazards that require measures
- Device deficiencies

The Sponsor-Investigator will evaluate SAEs with regard to causality and seriousness. Device deficiencies are assessed regarding their potential to lead to an SAE.

Pregnancies

Because pregnancy tests are a prerequisite to participation and the intervention is only 3 weeks, pregnancies will not be further assessed or reported.

Reporting to Authorities [ClinO Art. 42]:

In Category A studies, the sponsor is subject to the notification requirements specified in Art. 15 of the MedDO of 17 October 2011 (SR 812.213).

It is the Investigator's responsibility to report to the Ethics Committee via BASEC **device deficiencies** that could have led to serious adverse events if suitable action had not been taken, intervention had not been made, or circumstances had been less fortunate within 7 days [ClinO Art. 42].

Health hazards that require measures are reported to the Ethics Committee via BASEC within 2 days [ClinO Art. 37].

Periodic safety reporting:

A yearly safety update-report is submitted by the Investigator to the Ethics Committee via BASEC.

A report is submitted to Swissmedic by the Sponsor-Investigator, as defined in Art. 15a,b of the MedDO of 17 October 2011 (SR 812.213).

10.1.3 Follow up of (Serious) Adverse Events

If adverse events occur, patients are followed-up with extra-visits after 14 days until the issue has resolved. This applies to patients who continue with the study but also to patients who prematurely exit the study. In case subjects are lost to followed-up, study personnel will contact the treating psychiatrists (or if unavailable, the general practitioners) in order to inform on serious adverse events and the recommendations for follow-up clinical examinations. In cases of headaches for example, care would include the prescription of a non-steroidal antirheumatic drug, i.e. pain medicine, according to the person's preferences, medical history, or interaction with existing medication.

11. STATISTICAL METHODS

A two-tailed p-value of < .05 is considered to be statistically significant in all analyses. We will also report effect sizes for the comparisons of primary and clinical secondary outcome variables between groups.

11.1 Hypotheses

Aim 1: investigate the clinical and functional neural changes following 15 sessions of daily rTMS

Hypothesis 1a (main hypothesis): If-rTMS (inhibitory) will be superior over placebo or iTBS (facilitatory) in all clinical and motor behavioral measures, e.g. greater reduction in SRRS scores from baseline, greater increase in activity levels, or coin rotations.

Hypothesis 1b: If-rTMS will reduce aberrant functional connectivity in the motor system, e.g. between thalamus and M1. If-rTMS will alter regional CBF in the motor system and increase SMA and M1 activity during the fMRI task. No relevant changes are expected in the sham group, iTBS may deteriorate neural alterations.

Hypothesis 1c: cortical excitability will be differentially changed with rTMS. Lf-rTMS will increase SICI, while iTBS will reduce SICI.

Aim 2: characterization of psychomotor slowing (PS) in schizophrenia spectrum disorders compared to healthy controls

Hypothesis 2a: Patients will have poorer performance on all motor tasks, eg. reduced activity levels, increased postural sway, and less coin rotations

Hypothesis 2b: Patients will have aberrant structural and functional connectivity within the motor system, as well as increased CBF in basal ganglia and decreased CBF in premotor/motor cortex. Applying network metrics, the functional motor network will be less efficient in schizophrenia.

Hypothesis 2c: Behavioral measures of PS will be associated with aberrant motor network structure, perfusion, function, and connectivity. For example, patients with strong PS will have increased resting state functional connectivity between thalamus and M1. PS severity will be linked to reduced structural motor network efficiency, lower M1 and SMA activity during the fMRI task, and increased SMA perfusion at rest.

Hypothesis 2d: Patients will have increased motor cortex excitability (e.g. reduced SICI), which will be linked to measures of PS.

Aim 3 characterize short term dynamics of PS

Hypothesis 3a: in very few subjects we expect relevant spontaneous improvements in PS measures. The majority will have less than 20% fluctuation of motor parameters within 3 weeks

Hypothesis 3b: we expect slight longitudinal changes in resting state functional connectivity and perfusion of the motor system, but no structural changes.

Aim 4: to describe the short-term and medium-term clinical outcome of 3 weeks of rTMS intervention

Hypothesis 4a: patients receiving inhibitory rTMS will have better outcome (less PS, less general symptom severity) shortly after the intervention (at 6 week follow-up) compared to patients receiving placebo or facilitatory iTBS

Hypothesis 4b: patients receiving inhibitory rTMS will have better outcome (less PS, less general symptom severity) and superior function (social and global) at 6-month follow-up compared to patients receiving placebo or facilitatory iTBS

Hypothesis 4c (exploratory): changes in functional connectivity within the motor system from baseline to week 3 will predict better outcome at week 6, particularly reduced M1-thalamus functional connectivity.

11.2 Determination of Sample Size

The main effects of the 3-week interventions on PS (SRRS scores) will be calculated in a repeated measures design including 2 measures (Baseline, week 3) and four groups (lf-rTMS, iTBS, sham, waiting group-lf-rTMS). Assuming a moderate effect size (f = 0.23) as indicated by our pilot data in a repeated measures ANOVA with moderate correlation between time points (0.5), a power of 0.95 and an alpha = 0.05, we would need 88 patients (22 per group).

11.3 Statistical criteria of termination of trial

No statistical stopping rules are planned.

11.4 Planned Analyses

11.4.1 Datasets to be analysed, analysis populations

All data from subjects in the intent-to-treat population, who received at least one dose of rTMS will be used in the analyses of rTMS effects. We will conduct sensitivity analyses in all trial-completers (see 11.5).

In the analyses on biological and clinical correlates of PS, we will use all data of the baseline assessment, thus including data from subjects who dropped-out prior to the first rTMS session.

11.4.2 Primary Analysis

The primary analysis will be a repeated measures ANOVA of SRRS scores with two timepoints (within subjects: baseline and week 3) and four groups (lf-rTMS, iTBS, sham, waiting group-lf-rTMS). In case of significant baseline demographic or clinical group differences, we will adjust for these in an repeated measures ANCOVA of SRRS.

The analysis will be performed by the PI and his team in SPSS using data from the RedCap database.

11.4.3 Secondary Analyses

Secondary analyses focus on the secondary outcomes (see 5.2) and hypotheses (see 11.1).

Crosstabs are used to calculate the proportion of responders (>=30% reduction in SRRS from baseline) across groups. Repeated measures ANOVAs will clarify the time-course of SRRS change from baseline to week 6 (including week 1, 2, 3 and 6) and differences between groups.

Repeated measures ANOVAs will test the change of clinical and motor rating scales between baseline and week 3, week 6, and week 24 and differences between groups.

Linear regression analyses will test the association between neuroimaging markers (resting state perfusion, connectivity, fMRI activation, ect.) and measures of PS at baseline.

Repeated measures ANOVAs will test the longitudinal changes in neuroimaging markers from baseline to week 3.

All analyses will be performed by the PI and his team or collaborators with permission of the PI.

11.4.4 Interim analyses

Interim analyses of the primary analysis are planned after enrolment of 10, 20, 30, 40, 50, 60, and 70 subjects. The scope is to estimate whether any adjustments of the treatment arms are necessary. In addition, baseline data can be tested for associations between neuroimaging data and clinical/motor measures after enrolment of 20, 40, or 60 subjects.

All interim analyses are optional and at the discretion of the PI. Interim analyses are conducted by the PI and his team.

11.4.5 Safety analysis

The study team will provide a descriptive analysis of all reported adverse events. The analysis will focus on the type of event, duration, and timing and relate the number of observed events to the number of rTMS sessions administered. Furthermore, the frequency of adverse events will be compared between treatment arms of the trial.

11.4.6 Deviation(s) from the original statistical plan

Any deviation from the statistical plan will be reported and justified in the methods of the study report.

11.5 Handling of missing data and drop-outs

We will apply the last-observation-carried-forward (LOCF) method in the final analyses. In case a patient withdraws consent before the first rTMS stimulation, we will recruit an additional patient. The number of all patients recruited and randomized will be reported in the publications.

Sensitivity analyses will be performed in order to establish whether premature dropout would influence the results. Therefore, the primary analyses will be repeated with all subjects who completed the interventional trial. Further tests will establish whether drop-outs differ from completers in basic clinical or demographic variables.

12. QUALITY ASSURANCE AND CONTROL

All study personnel will be trained by the PI to achieve consistent adherence to procedures and interrater reliability. Standard operating procedures will be written for the intervention and standardized TMS measurements. For all clinical rating scales, instructions and manuals are provided for the study personnel. All study personnel will have to read the manuals.

12.1 Data handling and record keeping / archiving

12.1.1 Case Report Forms

All relevant data is documented in paper CRFs. This data includes inclusion/exclusion criteria, demographic data, medical history, scores of clinical and motor rating scales. Furthermore, side effects, concomitant treatment, reasons for discontinuation will be recorded in the CRF. Timing and conductance of experimental measures, such as posturography, cerebral MRI, cortical excitability, and actigraphy will be recorded recorded in the CRF. However, the real data of these measures is stored electronically outside the CRF.

For each enrolled study participant a CRF is maintained. CRFs will be kept current to reflect subject status at each phase during the course of study. Participants will be coded by use of a participant number in combination with the year of birth.

The paper forms of rating scales (e.g. PANSS) and the electronic data (e.g. MRI, posturography, ect.) are considered source documents. Furthermore, in patients the medical record of our hospital is a source document for the past psychiatric history and current concomitant treatment. All other data (participant history, side effects, ect.) are directly entered into the CRF without further source.

The study personnel is authorized to enter data into the CRFs, the name of the entering person will be documented. CRF data will be recorded in an electronic database, i.e. RedCap-Database. The procedure will include data cross-check between electronic and paper CRF. Any deviation will be corrected by consensus and consulting the CRF forms. RedCap-Database has individualized logins for each member of the study team and provides logs for data entry.

The electronic database for the non-CRF data will be logged via a secure Dropbox folder, thus the person entering the data or changing data can be identified by individualized logins. The log also identifies the document changes and the time of the change. Dropbox allows for restoring each version of the documents within the study folder.

12.1.2 Specification of source documents

As described in 12.1.1. most data in the CRFs is source data. Furthermore, Informed consent form, specific rating scales and electronic data (such as MRI, actigraphy or posturography) are source data. In patients, the medical record of the hospital may contain source data on the psychiatric history. All CRFs and paper source data will be kept in folders. Any extra examination in case of additional safety assessments will be documented on paper and kept as source data in the same folders as CRF and other source data.

12.1.3 Record keeping / archiving

All study data will be archived for 10 years after study termination or premature termination of the trial at the translational research center of the University Hospital of Psychiatry, Bern.

12.2 Data management

Data of all participants will be encrypted and analysis will be performed using encrypted data only. Unblinding of participant-specific data is only possible after consent has been given by the participants and the sponsor. Data of all participants will receive a numerical identification. The key to this encryption code will be stored in a single file at the address below, and it will be accessible only by the sponsor and the principal investigator.

12.2.1 Data Management System

Describe what system (also software) is being used and who is responsible and how it is tested before the trial (may include a description of where the system is hosted).

12.2.1.1 Neuroimaging data

MRI data are anonymized digital data of appr. 1 GB per subject and scan, we will aim for 278 scans,

i.e. total of 300 GB. MRI data sets include the dicom files of the planned sequences fMRI task, fMRI rest, ASL rest, DTI and T1 anatomy. MRI data sets will be safely stored in encrypted formats at the server of the translational research center, university hospital in Bern. Data will be logged with personal logins, such that any change to the files is reproducible. Data analysis will be performed locally in computers of the institute's network. Some analyses are planned to be performed on encrypted data at computers of the study collaborator Prof. Bernard. All files generated during data processing and analysis will be stored. Total data volume is approximately 2 TB. Neuroimaging data will be stored at a dropbox-folder, which is encrypted by Boxcryptor Software and owned by the Translational Research Center, University Hospital of Psychiatry, Bern. The system has been effectively used in several studies. Access to the folder and files is secured by personal login and password. The log of the Dropbox folder enables the identification of data entry/data change according to the logins used. The log will inform on which data has been changed when by whom. Dropbox allows for restoration of each file version, thus each change can be tracked.

12.2.1.2 _motor behavioral/instrumental data_

Data from actigraphy, posturography, TMS-MEP experiments as well as video recordings of coin rotation will be stored anonymized in digital format. As neuroimaging data, storage will comply with the federal human research act and will be conducted at standard facilities of the translational research center with encrypted and logged server storage. Data access is granted upon personal logins, such that any change to the files is reproducible. Each dataset includes several measures across 4-5 time points for one study participant. We expect appr. 1 GB per subject, total of 130 GB.

12.2.1.3 _clinical and demographic data_

Clinical data including outcome measures with observer based rating scales or patients' history will be collected. This data is transferred to paper case report forms and will later be anonymously transferred to a red cap data base, hosted by the CTU Bern. Data access is only available to study staff with personalized logins. During the longitudinal assessments approximately 300 variables will be assessed for each participant.

12.2.2 Data security, access and back-up

Only the PI and authorized personnel with own logins and passwords will have access to the electronic data. The translational research center Bern runs a daily backup of the dropbox folders. The RedCap Database also has regular backups.

12.2.3 Analysis and archiving

All analyses will be performed with a final copy of the SPSS file including all clinical data. All other analyses (neuroimaging, posturography, actigraphy) will be conducted with the appropriate standard software. Only the PI and authorized personnel will have access to the data to perform the analyses.

12.2.4 Electronic and central data validation

Data will be checked for consistency, e.g. range checks for questionnaire scores or test scores, checks for date entries temporal consistency.

12.3 Monitoring

The principal investigator will check the CRFs for completeness and internal consistency (plausibility of information) and external consistency (with source data) after each participant completed the study. In addition an external monitoring will be performed by Dr. Peter.

Four monitoring dates are planned. The first before the study recruitment starts, the second after the first three participants have been included, the third after 50% of enrolment was achieved and the final monitoring is planned after the last data had been collected.

Before first recruitment: Check whether all study assessments and instruments are ready and complete.

After the first three participants: Check CRF entries and compliance with source data where possible. Clarify queries of data entry or study procedures.

After the 50% enrolment: Check whether CRFs of the all collected cases are completed and data is consistent.

After the final data collection: Check whether CRFs of the all cases collected after the last monitoring are completed and data is consistent.

12.4 Audits and Inspections

Not intended for this single center trial.

12.5 Confidentiality, Data Protection

All data will be handled strictly confidential. Direct access to the source documents will be permitted only for purposes of inspections (ICHE6, 6.10) by authorities such as the CEC or monitoring by the PI. Only the PI and his team will have access to the protocol, dataset and other study related information during and after the study.

12.6 Storage of biological material and related health data

No storage of biological material is planned.

All electronic data will be archived for 10 years (see 12.1).

13. PUBLICATION AND DISSEMINATION POLICY

Publication of results is the responsibility of the sponsor/investigator. The results of the study will be prepared for publication in peer-reviewed scientific journals, preferably as open-access articles. Authorship will follow the national guidelines. Furthermore, results will be reported at scientific conferences as posters or oral communications. The use of professional writers is not intended. After scientific publication, the results will be prepared for communication in lay man's terms in the media.

Data sharing outside of the project is not intended.

14. FUNDING AND SUPPORT

14.1 Funding

This study receives project funding by the Swiss National Science Foundation (grant #182469 to Prof. Sebastian Walther).

14.2 Other Support

Consumables and further material support is provided by the host institution, the Translational Research Center of the University Hospital of Psychiatry, University of Bern, Switzerland.

15. INSURANCE

No special insurance due to category A.

16. REFERENCES

1. Rossi S, Hallett M, Rossini PM, Pascual-Leone A, Safety of TMSCG. Safety, ethical considerations, and application guidelines for the use of transcranial magnetic stimulation in clinical practice and research. Clin Neurophysiol 2009;120:2008-2039.
2. Lefaucheur JP, Andre-Obadia N, Antal A, et al. Evidence-based guidelines on the therapeutic use of repetitive transcranial magnetic stimulation (rTMS). Clin Neurophysiol 2014;125:2150-2206.
3. Owen MJ, Sawa A, Mortensen PB. Schizophrenia. Lancet 2016;388:86-97.
4. Howes OD, Murray RM. Schizophrenia: an integrated sociodevelopmental-cognitive model. Lancet 2014;383:1677-1687.
5. Tandon R, Nasrallah HA, Keshavan MS. Schizophrenia, "just the facts" 4. Clinical features and conceptualization. Schizophr Res 2009;110:1-23.
6. Yang GJ, Murray JD, Wang XJ, et al. Functional hierarchy underlies preferential connectivity disturbances in schizophrenia. Proc Natl Acad Sci U S A 2016;113:E219-228.
7. van den Heuvel MP, Sporns O, Collin G, et al. Abnormal rich club organization and functional brain dynamics in schizophrenia. JAMA Psychiatry 2013;70:783-792.
8. Klauser P, Baker ST, Cropley VL, et al. White Matter Disruptions in Schizophrenia Are Spatially Widespread and Topologically Converge on Brain Network Hubs. Schizophr Bull 2016.
9. Friston KJ. Schizophrenia and the disconnection hypothesis. Acta Psychiatr Scand Suppl 1999;395:68-79.
10. Walther S, Mittal VA. Motor System Pathology in Psychosis. Curr Psychiatry Rep 2017;19:97.
11. Walther S, Stegmayer K, Federspiel A, Bohlhalter S, Wiest R, Viher PV. Aberrant Hyperconnectivity in the Motor System at Rest Is Linked to Motor Abnormalities in Schizophrenia Spectrum Disorders. Schizophr Bull 2017;43:982-992.
12. Mittal V, Bernard JA, Northoff G. What can Different Motor Circuits Tell Us About Psychosis? An RDoC Perspective. Schizophr Bull 2017.
13. Kindler J, Schultze-Lutter F, Michel C, et al. Abnormal involuntary movements are linked to psychosis-risk in children and adolescents: Results of a population-based study. Schizophr Res 2016;174:58-64.
14. Mittal VA, Neumann C, Saczawa M, Walker EF. Longitudinal progression of movement abnormalities in relation to psychotic symptoms in adolescents at high risk of schizophrenia. Arch Gen Psychiatry 2008;65:165-171.
15. van Harten PN, Walther S, Kent JS, Sponheim SR, Mittal VA. The clinical and prognostic value of motor abnormalities in psychosis, and the importance of instrumental assessment. Neurosci Biobehav Rev 2017;80:476-487.
16. Peralta V, Campos MS, De Jalon EG, Cuesta MJ. Motor behavior abnormalities in drug-naive patients with schizophrenia spectrum disorders. Mov Disord 2010;25:1068-1076.
17. Putzhammer A, Perfahl M, Pfeiff L, Hajak G. Correlation of subjective well-being in schizophrenic patients with gait parameters, expert-rated motor disturbances, and psychopathological status. Pharmacopsychiatry 2005;38:132-138.
18. Kao YC, Liu YP, Chou MK, Cheng TH. Subjective quality of life in patients with chronic schizophrenia: relationships between psychosocial and clinical characteristics. Compr Psychiatry 2011;52:171-180.
19. Mittal VA. Cross-Cutting Advancements Usher in a New Era for Motor Research in Psychosis. Schizophr Bull 2016;42:1322-1325.
20. Walther S, Stegmayer K, Sulzbacher J, et al. Nonverbal social communication and gesture control in schizophrenia. Schizophr Bull 2015;41:338-345.
21. Walther S, Eisenhardt S, Bohlhalter S, et al. Gesture Performance in Schizophrenia Predicts Functional Outcome After 6 Months. Schizophrenia Bull 2016;42:1326-1333.
22. Walther S. Psychomotor symptoms of schizophrenia map on the cerebral motor circuit. Psychiatry Res 2015;233:293-298.
23. Morrens M, Hulstijn W, Sabbe B. Psychomotor slowing in schizophrenia. Schizophr Bull 2007;33:1038-1053.

24. Walther S, Strik W. Motor Symptoms and Schizophrenia. Neuropsychobiology 2012;66:77-92.
25. Morrens M, Docx L, Walther S. Beyond boundaries: in search of an integrative view on motor symptoms in schizophrenia. Front Psychiatr 2014;5:145.
26. Docx L, Sabbe B, Provinciael P, Merckx N, Morrens M. Quantitative psychomotor dysfunction in schizophrenia: a loss of drive, impaired movement execution or both? Neuropsychobiology 2013;68:221-227.
27. Walther S, Federspiel A, Horn H, et al. Alterations of white matter integrity related to motor activity in schizophrenia. Neurobiol Dis 2011;42:276-283.
28. Wichniak A, Skowerska A, Chojnacka-Wojtowicz J, et al. Actigraphic monitoring of activity and rest in schizophrenic patients treated with olanzapine or risperidone. J Psychiatr Res 2011;45:1381-1386.
29. Putzhammer A, Heindl B, Broll K, Pfeiff L, Perfahl M, Hajak G. Spatial and temporal parameters of gait disturbances in schizophrenic patients. Schizophr Res 2004;69:159-166.
30. Lisi G, Nico D, Ribolsi M, et al. Asymmetries in initiation of aiming movements in schizophrenia. Neuropsychologia 2018;109:200-207.
31. Docx L, Morrens M, Bervoets C, et al. Parsing the components of the psychomotor syndrome in schizophrenia. Acta Psychiatr Scand 2012;126:256-265.
32. Morrens M, Hulstijn W, Van Hecke J, Peuskens J, Sabbe BG. Sensorimotor and cognitive slowing in schizophrenia as measured by the Symbol Digit Substitution Test. J Psychiatr Res 2006;40:200-206.
33. Fervaha G, Agid O, Takeuchi H, et al. Extrapyramidal symptoms and cognitive test performance in patients with schizophrenia. Schizophr Res 2015;161:351-356.
34. Peralta V, Cuesta MJ. Neuromotor abnormalities in neuroleptic-naive psychotic patients: antecedents, clinical correlates, and prediction of treatment response. Compr Psychiatry 2011;52:139-145.
35. Walther S, Stegmayer K, Horn H, Razavi N, Muller TJ, Strik W. Physical Activity in Schizophrenia is Higher in the First Episode than in Subsequent Ones. Front Psychiatry 2014;5:191.
36. Cuesta MJ, Sanchez-Torres AM, de Jalon EG, et al. Spontaneous parkinsonism is associated with cognitive impairment in antipsychotic-naive patients with first-episode psychosis: a 6-month follow-up study. Schizophr Bull 2014;40:1164-1173.
37. Grootens KP, Vermeeren L, Verkes RJ, et al. Psychomotor planning is deficient in recent-onset schizophrenia. Schizophr Res 2009;107:294-302.
38. Mittal VA, Gupta T, Orr JM, et al. Physical activity level and medial temporal health in youth at ultra high-risk for psychosis. J Abnorm Psychol 2013;122:1101-1110.
39. Dean DJ, Mittal VA. Spontaneous parkinsonisms and striatal impairment in neuroleptic free youth at ultrahigh risk for psychosis. NPJ Schizophr 2015;1.
40. Niendam TA, Bearden CE, Johnson JK, et al. Neurocognitive performance and functional disability in the psychosis prodrome. Schizophr Res 2006;84:100-111.
41. Stubbs B, Ku PW, Chung MS, Chen LJ. Relationship Between Objectively Measured Sedentary Behavior and Cognitive Performance in Patients With Schizophrenia Vs Controls. Schizophr Bull 2017;43:566-574.
42. Stubbs B, Chen LJ, Chung MS, Ku PW. Physical activity ameliorates the association between sedentary behavior and cardiometabolic risk among inpatients with schizophrenia: A comparison versus controls using accelerometry. Compr Psychiatry 2017;74:144-150.
43. Martin-Sierra A, Vancampfort D, Probst M, et al. Walking capacity is associated with health related quality of life and physical activity level in patients with schizophrenia: a preliminary report. Actas Esp Psiquiatr 2011;39:211-216.
44. Gur RC, Ragland JD, Moberg PJ, et al. Computerized neurocognitive scanning: II. The profile of schizophrenia. Neuropsychopharmacology 2001;25:777-788.
45. Lee RS, Hermens DF, Naismith SL, et al. Neuropsychological and functional outcomes in recent-onset major depression, bipolar disorder and schizophrenia-spectrum disorders: a longitudinal cohort study. Transl Psychiatry 2015;5:e555.
46. Boden R, Abrahamsson T, Holm G, Borg J. Psychomotor and cognitive deficits as predictors of

5-year outcome in first-episode schizophrenia. Nord J Psychiatry 2014;68:282-288.

47. Tohen M, Khalsa HM, Salvatore P, et al. The McLean-Harvard First-Episode Project: Early Course in 114 Cases of First-Episode Nonaffective Psychoses. J Clin Psychiatry 2016;77:781-788.
48. Walther S, Koschorke P, Horn H, Strik W. Objectively measured motor activity in schizophrenia challenges the validity of expert ratings. Psychiatry Res 2009;169:187-190.
49. Walther S, Ramseyer F, Horn H, Strik W, Tschacher W. Less structured movement patterns predict severity of positive syndrome, excitement, and disorganization. Schizophr Bull 2014;40:585-591.
50. Walther S, Stegmayer K, Horn H, et al. The Longitudinal Course of Gross Motor Activity in Schizophrenia - Within and between Episodes. Front Psychiatry 2015;6:10.
51. Gebhardt A, Vanbellingen T, Baronti F, Kersten B, Bohlhalter S. Poor dopaminergic response of impaired dexterity in Parkinson's disease: Bradykinesia or limb kinetic apraxia? Mov Disord 2008;23:1701-1706.
52. Walther S, Vanbellingen T, Muri R, Strik W, Bohlhalter S. Impaired pantomime in schizophrenia: Association with frontal lobe function. Cortex 2013;49:520-527.
53. Kent JS, Hong SL, Bolbecker AR, et al. Motor deficits in schizophrenia quantified by nonlinear analysis of postural sway. PLoS One 2012;7:e41808.
54. Matsuura Y, Fujino H, Hashimoto R, et al. Standing postural instability in patients with schizophrenia: Relationships with psychiatric symptoms, anxiety, and the use of neuroleptic medications. Gait Posture 2015;41:847-851.
55. Peralta V, Cuesta MJ. The effect of antipsychotic medication on neuromotor abnormalities in neuroleptic-naive nonaffective psychotic patients: a naturalistic study with haloperidol, risperidone, or olanzapine. Prim Care Companion J Clin Psychiatry 2010;12.
56. Malla A, Norman R, Scholten D, et al. A comparison of two novel antipsychotics in first episode non-affective psychosis: one-year outcome on symptoms, motor side effects and cognition. Psychiatry Res 2004;129:159-169.
57. Kopala LC, Good KP, Milliken H, et al. Treatment of a first episode of psychotic illness with quetiapine: an analysis of 2 year outcomes. Schizophr Res 2006;81:29-39.
58. Walther S, Federspiel A, Horn H, et al. Resting state cerebral blood flow and objective motor activity reveal basal ganglia dysfunction in schizophrenia. Psychiatry Res 2011;192:117-124.
59. Docx L, Emsell L, Van Hecke W, et al. White matter microstructure and volitional motor activity in schizophrenia: A diffusion kurtosis imaging study. Psychiatry Res 2016;260:29-36.
60. Bracht T, Schnell S, Federspiel A, et al. Altered cortico-basal ganglia motor pathways reflect reduced volitional motor activity in schizophrenia. Schizophr Res 2013;143:269-276.
61. Karbasforoushan H, Duffy B, Blackford JU, Woodward ND. Processing speed impairment in schizophrenia is mediated by white matter integrity. Psychol Med 2015;45:109-120.
62. Singh S, Goyal S, Modi S, et al. Motor function deficits in schizophrenia: an fMRI and VBM study. Neuroradiology 2014;56:413-422.
63. Walther S, Schappi L, Federspiel A, et al. Resting-State Hyperperfusion of the Supplementary Motor Area in Catatonia. Schizophr Bull 2017;43:972-981.
64. Scheuerecker J, Ufer S, Kapernick M, et al. Cerebral network deficits in post-acute catatonic schizophrenic patients measured by fMRI. J Psychiatr Res 2009;43:607-614.
65. Payoux P, Boulanouar K, Sarramon C, et al. Cortical motor activation in akinetic schizophrenic patients: a pilot functional MRI study. Mov Disord 2004;19:83-90.
66. Bunse T, Wobrock T, Strube W, et al. Motor cortical excitability assessed by transcranial magnetic stimulation in psychiatric disorders: a systematic review. Brain Stimul 2014;7:158-169.
67. Hasan A, Wobrock T, Grefkes C, et al. Deficient inhibitory cortical networks in antipsychotic-naive subjects at risk of developing first-episode psychosis and first-episode schizophrenia patients: a cross-sectional study. Biol Psychiatry 2012;72:744-751.
68. Du X, Kochunov P, Summerfelt A, Chiappelli J, Choa FS, Hong LE. The role of white matter microstructure in inhibitory deficits in patients with schizophrenia. Brain Stimul 2017;10:283-290.
69. Hallett M. Transcranial magnetic stimulation: a primer. Neuron 2007;55:187-199.

70. Orosz A, Jann K, Wirth M, Wiest R, Dierks T, Federspiel A. Theta burst TMS increases cerebral blood flow in the primary motor cortex during motor performance as assessed by arterial spin labeling (ASL). Neuroimage 2012;61:599-605.

71. Siebner HR, Filipovic SR, Rowe JB, et al. Patients with focal arm dystonia have increased sensitivity to slow-frequency repetitive TMS of the dorsal premotor cortex. Brain 2003;126:2710-2725.

72. Ji GJ, Yu F, Liao W, Wang K. Dynamic aftereffects in supplementary motor network following inhibitory transcranial magnetic stimulation protocols. Neuroimage 2017;149:285-294.

73. Oxley T, Fitzgerald PB, Brown TL, de Castella A, Daskalakis ZJ, Kulkarni J. Repetitive transcranial magnetic stimulation reveals abnormal plastic response to premotor cortex stimulation in schizophrenia. Biol Psychiatry 2004;56:628-633.

74. Peralta V, Cuesta MJ. Motor Abnormalities: From Neurodevelopmental to Neurodegenerative Through "Functional" (Neuro)Psychiatric Disorders. Schizophr Bull 2017;43:956-971.

75. Speer AM, Willis MW, Herscovitch P, et al. Intensity-dependent regional cerebral blood flow during 1-Hz repetitive transcranial magnetic stimulation (rTMS) in healthy volunteers studied with H215O positron emission tomography: I. Effects of primary motor cortex rTMS. Biol Psychiatry 2003;54:818-825.

76. Tallabs FA, Hammond-Tooke GD. Theta priming of 1-Hz rTMS in healthy volunteers: effects on motor inhibition. J Clin Neurophysiol 2013;30:79-85.

77. Shirota Y, Ohtsu H, Hamada M, Enomoto H, Ugawa Y, Research Committee on r TMSToPsD. Supplementary motor area stimulation for Parkinson disease: a randomized controlled study. Neurology 2013;80:1400-1405.

78. Benninger DH, Berman BD, Houdayer E, et al. Intermittent theta-burst transcranial magnetic stimulation for treatment of Parkinson disease. Neurology 2011;76:601-609.

79. Kindler J, Homan P, Jann K, et al. Reduced neuronal activity in language-related regions after transcranial magnetic stimulation therapy for auditory verbal hallucinations. Biol Psychiatry 2013;73:518-524.

80. Hoffman RE, Hawkins KA, Gueorguieva R, et al. Transcranial magnetic stimulation of left temporoparietal cortex and medication-resistant auditory hallucinations. Arch Gen Psychiatry 2003;60:49-56.

81. Ray P, Sinha VK, Tikka SK. Adjuvant low-frequency rTMS in treating auditory hallucinations in recent-onset schizophrenia: a randomized controlled study investigating the effect of high-frequency priming stimulation. Ann Gen Psychiatry 2015;14:8.

82. Bais L, Vercammen A, Stewart R, et al. Short and long term effects of left and bilateral repetitive transcranial magnetic stimulation in schizophrenia patients with auditory verbal hallucinations: a randomized controlled trial. PLoS One 2014;9:e108828.

83. Klirova M, Horacek J, Novak T, et al. Individualized rTMS neuronavigated according to regional brain metabolism ((18)FGD PET) has better treatment effects on auditory hallucinations than standard positioning of rTMS: a double-blind, sham-controlled study. Eur Arch Psychiatry Clin Neurosci 2013;263:475-484.

84. Paillere-Martinot ML, Galinowski A, Plaze M, et al. Active and placebo transcranial magnetic stimulation effects on external and internal auditory hallucinations of schizophrenia. Acta Psychiatr Scand 2017;135:228-238.

85. Kindler J, Homan P, Flury R, Strik W, Dierks T, Hubl D. Theta burst transcranial magnetic stimulation for the treatment of auditory verbal hallucinations: results of a randomized controlled study. Psychiatry Res 2013;209:114-117.

86. Vercammen A, Knegtering H, Bruggeman R, et al. Effects of bilateral repetitive transcranial magnetic stimulation on treatment resistant auditory-verbal hallucinations in schizophrenia: a randomized controlled trial. Schizophr Res 2009;114:172-179.

87. Blumberger DM, Vila-Rodriguez F, Thorpe KE, et al. Effectiveness of theta burst versus high-frequency repetitive transcranial magnetic stimulation in patients with depression (THREE-D): a randomised non-inferiority trial. Lancet 2018;391:1683-1692.

88. Duprat R, Desmyter S, Rudi de R, et al. Accelerated intermittent theta burst stimulation treatment in medication-resistant major depression: A fast road to remission? J Affect Disord 2016;200:6-14.

89. van Lutterveld R, Koops S, Schutter DJ, et al. The effect of rTMS on auditory hallucinations: clues from an EEG-rTMS study. Schizophr Res 2012;137:174-179.

90. Nahas Z, Bohning DE, Molloy MA, Oustz JA, Risch SC, George MS. Safety and feasibility of repetitive transcranial magnetic stimulation in the treatment of anxious depression in pregnancy: a case report. J Clin Psychiatry 1999;60:50-52.

91. Klirova M, Novak T, Kopecek M, Mohr P, Strunzova V. Repetitive transcranial magnetic stimulation (rTMS) in major depressive episode during pregnancy. Neuro Endocrinol Lett 2008;29:69-70.

92. Widlöcher D, Ghozlan A. The measurement of retardation in depression. In: Hindmarch I, Stonier PD, eds. Human Psychopharmacology: Measures and Methods. New York: Wiley, 1989.

93. Kay SR, Fiszbein A, Opler LA. The positive and negative syndrome scale (PANSS) for schizophrenia. Schizophr Bull 1987;13:261-276.

94. Kirkpatrick B, Strauss GP, Nguyen L, et al. The brief negative symptom scale: psychometric properties. Schizophr Bull 2011;37:300-305.

95. Craig CL, Marshall AL, Sjostrom M, et al. International physical activity questionnaire: 12-country reliability and validity. Med Sci Sports Exerc 2003;35:1381-1395.

96. Dollfus S, Mach C, Morello R. Self-Evaluation of Negative Symptoms: A Novel Tool to Assess Negative Symptoms. Schizophr Bull 2016;42:571-578.

97. Endicott J, Spitzer RL, Fleiss JL, Cohen J. The global assessment scale. A procedure for measuring overall severity of psychiatric disturbance. Arch Gen Psychiatry 1976;33:766-771.

98. Goldman HH, Skodol AE, Lave TR. Revising axis V for DSM-IV: a review of measures of social functioning. Am J Psychiatry 1992;149:1148-1156.

99. Mausbach BT, Depp CA, Bowie CR, et al. Sensitivity and specificity of the UCSD Performance-based Skills Assessment (UPSA-B) for identifying functional milestones in schizophrenia. Schizophr Res 2011;132:165-170.

100. Huang YZ, Edwards MJ, Rounis E, Bhatia KP, Rothwell JC. Theta burst stimulation of the human motor cortex. Neuron 2005;45:201-206.

101. Oldfield RC. The assessment and analysis of handedness: the Edinburgh inventory. Neuropsychologia 1971;9:97-113.

102. Andreasen NC, Flaum M, Arndt S. The Comprehensive Assessment of Symptoms and History (CASH). An instrument for assessing diagnosis and psychopathology. Arch Gen Psychiatry 1992;49:615-623.

103. Kircher T, Krug A, Stratmann M, et al. A rating scale for the assessment of objective and subjective formal Thought and Language Disorder (TALD). Schizophr Res 2014;160:216-221.

104. Buchanan RW, Heinrichs DW. The Neurological Evaluation Scale (NES): a structured instrument for the assessment of neurological signs in schizophrenia. Psychiatry Res 1989;27:335-350.

105. Fahn S, Elton RL, Members UP. Unified Parkinson's disease rating scale. In: Fahn S, Marsden CD, Goldstein M, Calne DB, eds. Recent developments in Parkinson's disease. Florham Park, NJ: Macmillan Healthcare Information, 1987.

106. Bush G, Fink M, Petrides G, Dowling F, Francis A. Catatonia. I. Rating scale and standardized examination. Acta Psychiatr Scand 1996;93:129-136.

107. Guy W. ECDEU Assessment Manual for Psychopharmacology. Rockville, MD: US Department of Health, Education and

Welfare, 1976.

108. Walther S, Federspiel A, Horn H, et al. White matter integrity associated with volitional motor activity. Neuroreport 2010;21:381-385.

109. Walther S, Horn H, Koschorke P, Muller TJ, Strik W. Increased motor activity in cycloid psychosis compared to schizophrenia. World J Biol Psychiatry 2009;10:746-751.

110. Walther S, Horn H, Razavi N, Koschorke P, Muller TJ, Strik W. Quantitative motor activity differentiates schizophrenia subtypes. Neuropsychobiology 2009;60:80-86.

111. Walther S, Horn H, Razavi N, et al. Higher motor activity in schizophrenia patients treated with olanzapine versus risperidone. J Clin Psychopharmacol 2010;30:181-184.

112. Vanbellingen T, Wapp M, Stegmayer K, et al. Theta burst stimulation over premotor cortex in Parkinson's disease: an explorative study on manual dexterity. J Neural Transm (Vienna) 2016;123:1387-1393.
113. Rossini PM, Burke D, Chen R, et al. Non-invasive electrical and magnetic stimulation of the brain, spinal cord, roots and peripheral nerves: Basic principles and procedures for routine clinical and research application. An updated report from an I.F.C.N. Committee. Clin Neurophysiol 2015;126:1071-1107.
114. Claassen J, Spiegel R, Kalla R, et al. A randomised double-blind, cross-over trial of 4-aminopyridine for downbeat nystagmus--effects on slowphase eye velocity, postural stability, locomotion and symptoms. J Neurol Neurosurg Psychiatry 2013;84:1392-1399.
115. Krafczyk S, Tietze S, Swoboda W, Valkovic P, Brandt T. Artificial neural network: a new diagnostic posturographic tool for disorders of stance. Clin Neurophysiol 2006;117:1692-1698.
116. Genzel L, Dresler M, Cornu M, et al. Medial prefrontal-hippocampal connectivity and motor memory consolidation in depression and schizophrenia. Biol Psychiatry 2015;77:177-186.
117. Yokoi A, Arbuckle SA, Diedrichsen J. The Role of Human Primary Motor Cortex in the Production of Skilled Finger Sequences. J Neurosci 2018;38:1430-1442.

17. APPENDICES

1. IB (according to ISO 14155) – “Produktinformation”

2. TMS device manual

3. Case Report Forms, v1 of November 20th 2018, patient version and control version

4. Study Information for patients, v1 of November 22nd 2018

5. Study Information for healthy controls, v1 of November 22nd 2018

Overcoming psychomotor slowing in psychosis (OCoPS-P) – A 3-week, randomized, double-blind, placebo-controlled trial of add-on repetitive transcranial magnetic stimulation for psychomotor slowing in psychosis

Clinical Study Protocol

Short Title:	OCoPS-P
Translation	Überwinden von psychomotorischer Verlangsamung bei Psychosen - eine randomisierte, doppelblinde, plazebo-kontrollierte Studie zur Wirkung von transkranieller Magnetstimulation auf die psychomotorischer Verlangsamung bei Psychosen
Study Type:	Clinical trial testing the effects of 15 sessions of repetitive transcranial magnetic stimulation on psychomotor slowing in psychosis
Study Categorisation:	Risk category A
Study Registration:	Intended registry: clinicaltrials.gov<br>Registration number (from FOPH portal) eventually other registries and numbers if applicable
Study Identifier:	OCoPS-P, BASEC-Nr: 2018-02164
Sponsor, Sponsor-Investigator or Principal Investigator:	Prof. Dr. med. Sebastian Walther, University Hospital of Psychiatry, University of Bern, Murtenstrasse 21, 3008 Switzerland<br>Phone: 031 632 8979, fax: 031 632 8950, Email: sebastian.walther@upd.unibe.ch
Investigational Product:	Transcranial Magnetic Stimulation
Protocol Version and Date:	Version 2.0 December 13th 2018

CONFIDENTIAL

The information contained in this document is confidential and the property of Prof. Dr. med. Sebastian Walther (the sponsor). The information may not - in full or in part - be transmitted, reproduced, published, or disclosed to others than the applicable Competent Ethics Committee(s) and Regulatory Authority(ies) without prior written authorisation from the sponsor except to the extent necessary to obtain informed consent from those who will participate in the study.

Signature Page(s)

Study number	BASEC 2018-02164
Study Title	Overcoming psychomotor slowing in psychosis (OCoPS-P) – A 3-week, randomized, double-blind, placebo-controlled trial of add-on repetitive transcranial magnetic stimulation for psychomotor slowing in psychosis

The Sponsor-Investigator and trial statistician have approved the protocol version 2.0 (13.12.2018), and confirm hereby to conduct the study according to the protocol, current version of the World Medical Association Declaration of Helsinki, ICH-GCP guidelines or ISO 14155 norm if applicable and the local legally applicable requirements.

Sponsor-Investigator:
Prof. Dr. med. Sebastian Walther

Bern, 20.12.2018

Place/Date

Signature

Local Principal Investigator at study site*:

I have read and understood this trial protocol and agree to conduct the trial as set out in this study protocol, the current version of the World Medical Association Declaration of Helsinki, ICH-GCP guidelines or ISO 14155 norm and the local legally applicable requirements.

Site	University Hospital of Psychiatry, Bern
Principal investigator	Prof. Dr. med. Sebastian Walther

Bern, 20.12.2018

Place/Date

Signature

*Note: In multicentre studies, this page must be individually signed by all participating Local Principal Investigators.

Table of Contents

STUDY SYNOPSIS **8**
ABBREVIATIONS **12**
STUDY SCHEDULE **13**
1. STUDY ADMINISTRATIVE STRUCTURE **15**
1.1 Sponsor, Sponsor-Investigator 15
1.2 Principal Investigator(s) 15
1.3 Statistician ("Biostatistician") 15
1.4 Laboratory 15
1.5 Monitoring institution 15
1.6 Data Safety Monitoring Committee 15
1.7 Any other relevant Committee, Person, Organisation, Institution 15
2. ETHICAL AND REGULATORY ASPECTS **16**
2.1 Study registration 16
2.2 Categorisation of study 16
2.3 Competent Ethics Committee (CEC) 16
2.4 Competent Authorities (CA) 16
2.5 Ethical Conduct of the Study 16
2.6 Declaration of interest 16
2.7 Patient Information and Informed Consent 16
2.8 Participant privacy and confidentiality 17
2.9 Early termination of the study 17
2.10 Protocol amendments 17
3. BACKGROUND AND RATIONALE **18**
3.1 Background and Rationale 18
3.1.1 Schizophrenia 18
3.1.2 Motor system pathology in schizophrenia 18
3.1.3 Psychomotor slowing 19
3.1.4 Current treatment options of psychomotor slowing 19
3.1.5 Psychomotor slowing and motor network dysfunction 19
3.1.6 Intracortical excitability 20
3.1.7 Brain stimulation for psychomotor slowing 21
3.1.8 State of research summary 21
3.2 Investigational Product (treatment, device) and Indication 22
3.3 Preclinical Evidence 22
3.4 Clinical Evidence to Date 22
3.5 Dose Rationale / Medical Device: Rationale for the intended purpose in study (pre-market MD)22
3.6 Explanation for choice of comparator (or placebo) 22
3.7 Risks / Benefits 22
3.8 Justification of choice of study population 23
4. STUDY OBJECTIVES **24**
4.1 Overall Objective 24
4.2 Primary Objective 24
4.3 Secondary Objectives 24
4.4 Safety Objectives 24

5. STUDY OUTCOMES **24**
5.1 Primary Outcome 24
5.2 Secondary Outcomes 24
5.2.1 Clinical outcomes 24
5.2.2 Behavioral outcomes 25
5.2.3 Physiological outcomes 25
5.2.4 Neuroimaging outcomes 25
5.3 Other Outcomes of Interest 25
5.4 Safety Outcomes 25
6. STUDY DESIGN **25**
6.1 General study design and justification of design 25
6.2 Methods of minimising bias 26
6.2.1 Randomisation 26
6.2.2 Blinding procedures 26
6.2.3 Other methods of minimising bias 26
6.3 Unblinding Procedures (Code break) 26
7. STUDY POPULATION **26**
7.1 Eligibility criteria 26
7.2 Recruitment and screening 27
7.3 Assignment to study groups 27
7.4 Criteria for withdrawal / discontinuation of participants 27
8. STUDY INTERVENTION **28**
8.1 Identity of Investigational Products (treatment / medical device) 28
8.1.1 Experimental Intervention (treatment / medical device) 28
8.1.2 Control Intervention (standard/routine/comparator treatment / medical device) 28
8.1.3 Packaging, Labelling and Supply (re-supply) 28
8.1.4 Storage Conditions 28
8.2 Administration of experimental and control interventions 28
8.2.1 Experimental Intervention 28
8.2.2 Control Intervention 29
8.3 Dose / Device modifications 29
8.4 Compliance with study intervention 29
8.5 Data Collection and Follow-up for withdrawn participants 29
8.6 Trial specific preventive measures 29
8.7 Concomitant Interventions (treatments) 29
8.8 Study Drug / Medical Device Accountability 29
8.9 Return or Destruction of Study Drug / Medical Device 29
9. STUDY ASSESSMENTS **30**
9.1 Study flow chart(s) / table of study procedures and assessments 30
9.1.1 Patients of treatment groups 1-3 30
9.1.2 Patients of treatment group 4 (waiting group) 33
9.1.3 Control subjects 36
9.2 Assessments of outcomes 37
9.2.1 Assessment of primary outcome 38
9.2.2 Assessment of secondary outcomes 38
9.2.3 Assessment of other outcomes of interest 39

9.2.4 Assessment of safety outcomes ... 39
9.2.5 Assessments in participants who prematurely stop the study ... 40
9.3 Procedures at each visit ... 40
9.3.1 Screening visit ... 40
9.3.2 Visit 2 (baseline, week 0) ... 40
9.3.3 Visit 3 and 4 (week 1 and 2) ... 40
9.3.4 Visit 5 (end of intervention, week 3) ... 40
9.3.5 Visit 6 and 7 (follow-up, week 6 and 24) ... 41
10. SAFETY ... 41
10.1 Medical Device Category A studies ... 41
10.1.1 Definition and Assessment of safety related events ... 41
10.1.2 Reporting of (Serious) Adverse Events and other safety related events ... 42
10.1.3 Follow up of (Serious) Adverse Events ... 43
11. STATISTICAL METHODS ... 43
11.1 Hypotheses ... 43
11.2 Determination of Sample Size ... 44
11.3 Statistical criteria of termination of trial ... 44
11.4 Planned Analyses ... 44
11.4.1 Datasets to be analysed, analysis populations ... 44
11.4.2 Primary Analysis ... 44
11.4.3 Secondary Analyses ... 45
11.4.4 Interim analyses ... 45
11.4.5 Safety analysis ... 45
11.4.6 Deviation(s) from the original statistical plan ... 45
11.5 Handling of missing data and drop-outs ... 45
12. QUALITY ASSURANCE AND CONTROL ... 46
12.1 Data handling and record keeping / archiving ... 46
12.1.1 Case Report Forms ... 46
12.1.2 Specification of source documents ... 46
12.1.3 Record keeping / archiving ... 46
12.2 Data management ... 46
12.2.1 Data Management System ... 47
12.2.2 Data security, access and back-up ... 47
12.2.3 Analysis and archiving ... 47
12.2.4 Electronic and central data validation ... 47
12.3 Monitoring ... 47
12.4 Audits and Inspections ... 48
12.5 Confidentiality, Data Protection ... 48
12.6 Storage of biological material and related health data ... 48
13. PUBLICATION AND DISSEMINATION POLICY ... 48
14. FUNDING AND SUPPORT ... 48
14.1 Funding ... 48
14.2 Other Support ... 48

15. INSURANCE 49
16. REFERENCES 50
17. APPENDICES 56

STUDY SYNOPSIS

**Sponsor / Sponsor-Investigator**	Prof. Dr. med. Sebastian Walther,<br>University Hospital of Psychiatry, University of Bern, Murtenstrasse 21, 3008 Switzerland<br>Phone: 031 632 8979, fax: 031 632 8950, Email: sebastian.walther@upd.unibe.ch
**Study Title:**	Overcoming psychomotor slowing in psychosis (OCoPS-P) – A 3-week, randomized, double-blind, placebo-controlled trial of add-on repetitive transcranial magnetic stimulation for psychomotor slowing in psychosis
**Short Title / Study ID:**	OCoPS-P, KEK 2018-02164
**Protocol Version and Date:**	Protocol version 2.0 (13th December 2018)
**Trial registration:**	Intended at clinicaltrials.gov
**Study category and Rationale**	Category A: TMS device with CE certificate and use according to guidelines and manual. In addition short cerebral MRI and TMS experiments at baseline and endpoint. Minimal risk involved.
**Clinical Phase:**	Not applicable.

**Background and Rationale:**	Schizophrenia is a chronic disorder causing tremendous burden to the patients, families and society. Besides prominent symptoms such as hallucinations, delusions, and thought disorder, the majority of patients also experiences motor abnormalities. Converging evidence links aberrant structure and function of the cerebral motor network to schizophrenia pathology, particularly to motor abnormalities. One of the most frequent motor abnormalities is psychomotor slowing (PS), which may impact both gross and fine motor behavior. While PS causes significant distress and predicts poor outcome, researchers are just starting to understand its pathobiology. First evidence points to aberrant functional and structural connectivity within the cerebral motor network in schizophrenia patients with PS, particularly in connections between premotor/motor cortex and thalamus, as well as between motor cortex and cerebellum. In addition, severe motor inhibition was linked to increased neural activity in the premotor cortex. Repetitive transcranial magnetic stimulation (rTMS) may [illegible] –thobiology of PS, however, is still unknown. This study will combine a motor battery, advanced neuroimaging, and rTMS to probe the cerebral motor network contributions to PS.<br>The aims of this study are<br>(1) to investigate clinical and neural changes following 3 weeks of rTMS treatment (inhibitory, facilitatory or placebo), (2) to characterize neural correlates of psychomotor slowing (PS) on the network level, (3) to explore short term changes of PS by testing a waiting list cohort, and (4) to test the clinical outcome of rTMS for PS at 6-month follow-up.<br><br>To reach these aims, we plan to investigate four groups of schizophrenia patients (total 88) in a randomized, double blind, 4-arm sham-controlled trial of 15 rTMS sessions in 3 weeks with pre and post intervention MRI scans and a clinical follow-up at 6 months. One group will first be kept on a 3 week waiting list and then enter the study. Longitudinal MRI scans and motor tests separated by 3 weeks will also be applied to a control group of 40 healthy subjects for comparisons with the patient groups. We hypothesize that (1) PS would be linked to increased functional connectivity in motor cortical-basal ganglia loops as well as motor cortical-cerebellar loops, (2) inhibitory rTMS to the premotor cortex will reduce motor network functional connectivity and thus alleviate PS, (3) patients on the waiting list may experience stable PS severity, and finally, (4) patients responding to rTMS treatment of PS will have superior clinical and functional outcomes at 6-month follow-up. Thus, the study will substantially contribute to the understanding of PS by describing and probing the neural alterations in the motor network in schizophrenia associated with behavioral PS. Therefore, the study will impact future treatment strategies for PS and inform on the causal network pathology in schizophrenia
**Objective(s):**	1. investigate clinical and neural changes following 3 weeks of rTMS treatment (inhibitory, facilitatory or placebo)<br>2. characterize neural correlates of psychomotor slowing (PS) on the network level<br>3. explore short term changes of PS by testing a waiting list cohort<br>4. test the clinical outcome of rTMS for PS at 6-month follow-up.

**Outcome(s):**	1. Change from baseline to week 3 in SRRS scores. Furthermore, proportion of responders per study arm after 15 sessions rTMS (response = 30% reduction in SRRS scores from baseline)<br>2. Associations between multiple measures of motor function and neuroimaging markers, e.g. resting state perfusion or functional connectivity within the motor system<br>3. Change in SRRS from baseline to week 3 within the waiting list group, also comparison of SRRS change from baseline between waiting list group and placebo group.<br>4. Change in symptoms (PANSS, BNSS, SRRS) and functioning (SOFAS) from baseline to 6-month follow-up
**Study design:**	randomised, double-blind, four-arm, placebo-controlled trial of 3 weeks add-on rTMS for psychomotor slowing in schizophrenia spectrum disorders
**Inclusion / Exclusion criteria:**	Inclusion:<br>• Right-handed subjects, ages 18–60 years.<br>• Patients: schizophrenia spectrum disorders according to DSM-5 with psychomotor slowing (SRRS score ≥ 15). Patients are necessary, because only patients have the target symptoms, i.e. psychomotor slowing<br>• Controls: only for pre-/post comparisons of neuroimaging and physiology, **no intervention in controls**<br>Exclusion:<br>• General: Substance abuse or dependence other than nicotine. Past or current medical or neurological condition associated with impaired or aberrant movement, such as brain tumors, stroke, M. Parkinson, M. Huntington, dystonia, or severe head trauma with subsequent loss of conciousness. Epilepsy or other convulsions. History of any hearing problems or ringing in the ears. Standard exclusion criteria for MRI scanning and TMS; e.g. metal implants, claustrophobia. Women who are pregnant or breast feeding<br>• Patients only: any TMS treatment in the past 3 months<br>• Controls: history of any psychiatric disorder. First-degree relatives with schizophrenia spectrum disorders.
**Measurements and procedures:**	Participants will be screened and randomized to one of four arms before baseline assessments. Intervention period will be three weeks. Each week the primary outcome variable and safety will be assessed. At baseline and end of intervention (week 3), patients will be assessed with clinical and motor rating scales, tasks assessing fine and gross motor behaviour, TMS measures of cortical excitability, posturography, MRI neuroimaging, and tests of social and community functioning. Follow-ups will be conducted at week 6 and 24 including clinical and motor measures, cortical excitability, and social and community functioning. For cross-sectional comparisons of cortical excitability and neuroimaging, a group of 40 healthy control subjects matched for age, gender, and education, will be tested longitudinally with neuroimaging, motor tests, cortical excitability and posturography at baseline and week 3. Controls will not receive any intervention.
**Study Product / Intervention:**	low-frequency rTMS has inhibitory effects on brain function. We will apply 1'000 pulses at 1 Hz over the left SMA at an intensity of 110% of the resting motor threshold (approximate duration 17 minutes) in a total of 15 daily sessions (5 per week)

**Control Intervention (if applicable):**	**Active control:** Intermittent theta burst (iTBS) enhances local brain activity. We will apply 600 pulses at 50 Hz (stimulation in 2 sec trains every 10 sec for 190 sec in total) over the left SMA at an intensity of 80% of the resting motor threshold. iTBS will be repeated after15 min totaling to 1200 pulses per session in a total of 15 daily sessions (5 per week).<br><br>**Placebo control:** We will use a placebo-coil that looks identical to the real one and makes identical noises. Stimulation parameters are the same as in the active intervention, except that no magnetic pulse is emitted. Thus, placebo coil will be placed over the left SMA with 1000 clicks at 1 Hz (approximate duration 17 minutes) in a total of 15 daily sessions (5 per week).<br><br>**Waiting list:** This group will have baseline measures at baseline and week 3 and receive the active protocol from week 3 to week 6.
**Number of Participants with Rationale:**	Number of participants in the intervention: 88 patients (22 per protocol arm). In addition, to complement neuroimaging and neurophysiological analyses (no intervention) we will include 40 healthy control subjects matched for age, gender, and education.
**Study Duration:**	Total study duration will be 4 years. Total duration of participant recruitment will be 3 years.
**Study Schedule:**	Planned 03/2019 of First-Participant-In<br>Planned 06/2022 of Last-Participant-Out
**Investigator(s):**	- see Sponsor-Investigator
**Study Centre(s):**	Single-centre trial at the University Hospital of Psychiatry, Bern
**Statistical Considerations:**	The main effects of the 3-week interventions on PS (SRRS scores) will be calculated in a repeated measures design including 2 measures (Baseline, week 3) and four groups (lf-rTMS, iTBS, sham, waiting group-lf-rTMS). Assuming a moderate effect size (f = 0.23) as indicated by our pilot data in a repeated measures ANOVA with moderate correlation between time points (0.5), a power of 0.95 and an alpha = 0.05, we would need 88 patients (22 per group).
**GCP Statement:**	This study will be conducted in compliance with the protocol, the current version of the Declaration of Helsinki, the ICH-GCP or ISO EN 14155 (as far as applicable) as well as all national legal and regulatory requirements.

ABBREVIATIONS

AE	Adverse Event
BASEC	Business Administration System for Ethical Committees, (https://submissions.swissethics.ch/en/)
BNSS	Brief Negative Symptom Scale
CA	Competent Authority (e.g. Swissmedic)
CEC	Competent Ethics Committee
CRF	Case Report Form
ClinO	Ordinance on Clinical Trials in Human Research *(in German: KlinV, in French: OClin, in Italian: OSRUm)*
eCRF	Electronic Case Report Form
CTCAE	Common terminology criteria for adverse events
DSUR	Development safety update report
GCP	Good Clinical Practice
IB	Investigator's Brochure
Ho	Null hypothesis
H1	Alternative hypothesis
HRA	Federal Act on Research involving Human Beings *(in German: HFG, in French: LRH, in Italian: LRUm)*
IMP	Investigational Medicinal Product
IIT	Investigator-initiated Trial
ISO	International Organisation for Standardisation
iTBS	Intermittent Theta Burst Stimulation
ITT	Intention to treat
MD	Medical Device
MedDO	Medical Device Ordinance *(in German: MepV, in French: ODim)*
PANSS	Positive And Negative Syndrome Scale
PI	Principal Investigator
PS	Psychomotor Slowing
rTMS	Repetitive Transcranial Magnetic Stimulation
SAE	Serious Adverse Event
SDV	Source Data Verification
SMA	Supplementary Motor Area
SOP	Standard Operating Procedure
SPC	Summary of product characteristics
SRRS	Salpêtrière Retardation Rating Scale
SOFAS	Social and Occupational Functioning Scale
SUSAR	Suspected Unexpected Serious Adverse Reaction
TMF	Trial Master File
UPSA Brief	UCSD Performance-based Skills Assessment Brief version

STUDY SCHEDULE

Study Periods	Screening	Treatment, Intervention Period				Follow-up	
Visit	1	2*	3	4	5	6	7
Time (week)	-1	0	1	2	3	6	24
Patient Information and Informed Consent	x						
Demographics	x						
CASH and SCID (history)	x						
In- /Exclusion Criteria	x						
Physical Examination	x						
Pregnancy Test	x						
Psychopathology (TALD, NES)		x					
Randomisation		x					
Administer rTMS (5 sessions per week)		x	x	x			
Primary Variable SRRS		x	x	x	x	x	x
Motor scales UPDRS, BFCRS, AIMS, IPAQ		x			x	x	x
Psychopathology (PANSS, BNSS, SNS)		x			x	x	x
Actigraphy and coin rotation		x			x	x	x
Posturography		x			x		
TMS cortical excitability		x			x	x	x
Cerebral MRI		x			x		
Functional outcome (SOFAS, GAF, UPSA-brief)		x				x	x
Concomitant Therapy		x	x	x	x		
Adverse Events		x	x	x	x		

* please note that in the waiting group assessments of visit 2 will be repeated after 3 weeks and thereafter the protocol will be identical (see below).

Study protocol for patients in the waiting group

Study Periods	Scree ning	Treatment, Intervention Period					Follow-up	
Visit	1	2	3	4	5	6	7	8
Time (week)	-1	0	3	4	5	6	9	24
Patient Information and Informed Consent	x							
Demographics	x							
CASH and SCID (history)	x							
In- /Exclusion Criteria	x							
Physical Examination	x							
Pregnancy Test	x							
Psychopathology (TALD, NES)		x						
Randomisation		x						
Administer rTMS (5 sessions per week)			x	x	x			
Primary Variable SRRS		x	x	x	x	x	x	x
Motor scales UPDRS, BFCRS, AIMS, IPAQ		x	x			x	x	x
Psychopathology (PANSS, BNSS, SNS)		x	x			x	x	x
Actigraphy and coin rotation		x	x			x	x	x
Posturography		x	x			x		
TMS cortical excitability		x	x			x	x	x
Cerebral MRI		x	x			x		
Functional outcome (SOFAS, GAF, UPSA-brief)		x	x				x	x
Concomitant Therapy		x	x	x	x	x		
Adverse Events			x	x	x	x		

Study protocol for control subjects

Study Periods	Screening	Observation period	
Visit<br>Time (week)	1<br>-1	2<br>0	3<br>3
Proband Information and Informed Consent	x		
Demographics	x		
SCID	x		
In- /Exclusion Criteria	x		
Physical Examination	x		
Pregnancy Test	x		
Primary Variable SRRS		x	x
Motor scales UPDRS, BFCRS, AIMS, IPAQ		x	x
Actigraphy and coin rotation		x	x
Posturography		x	x
TMS cortical excitability		x	x
Cerebral MRI		x	x

1. STUDY ADMINISTRATIVE STRUCTURE

1.1 Sponsor, Sponsor-Investigator

Prof. Dr. med. Sebastian Walther,

University Hospital of Psychiatry, University of Bern, Murtenstrasse 21, 3008 Bern, Switzerland, phone: 031 632 4635, fax: 031 632 8950, Email: sebastian.walther@upd.unibe.ch

Prof. Walther will be Sponsor-Investigator; no further international sites are planned

Roles: protocol, study design, supervision of data collection and management, data analysis, data interpretation and writing of the report.

1.2 Principal Investigator(s)

Identical to sponsor, see 1.1.

1.3 Statistician ("Biostatistician")

Dr. Petra Viher, PhD, University Hospital of Psychiatry, University of Bern, Bolligenstrasse 111, 3000 Bern, Switzerland. Phone: 031 930 9757, Email: petra.viher@upd.unibe.ch

1.4 Laboratory

All of the procedures except neuroimaging will be performed at the University Hospital of Psychiatry, Bern. The devices and infrastructure are provided by the Translational research center at the University Hospital of Psychiatry, Bern, Bolligenstrasse 111, 3000 Bern 60, Switzerland.

Neuroimaging acquisition (magnetic resonance imaging – MRI) will be performed at the University Hospital Inselspital Bern, Institute of Diagnostic and Interventional Neuroradiology. Collaborator Prof. Dr. med. Roland Wiest.

1.5 Monitoring institution

Dr. phil. Jessica Peter, University Hospital of Old Age Psychiatry and Psychotherapy, University of Bern, Bolligenstrasse 111, 3000 Bern, Switzerland. Phone: 031 932 8903 Email: Jessica.peter@upd.unibe.ch

1.6 Data Safety Monitoring Committee

DSMC is not needed. The study aim is not to test the efficacy of a specific product. The objective is to test whether repetitive transcranial magnetic stimulation may improve psychomotor slowing and how it interferes with neurophysiology.

1.7 Any other relevant Committee, Person, Organisation, Institution

Study collaborators

Prof. Dr. Roland Wiest, University Institute of Diagnostic and Interventional Neuroradiology, Inselspital Bern, Switzerland. MRI acquisition

Prof. Dr. Andrea Federspiel, Neuroimaging Unit, Translational Research Center, University Hospital of Psychiatry, Bern, Switzerland, MRI analyses

Prof. Dr. Jessica Bernard, Department of Psychological and Brain Sciences, Texas A & M University, College Station, TX, USA, MRI analyses support

Prof. Dr. Roger Kalla, Department of Neurology, University Hospital Inselspital, Bern, Switzerland, posturography

2. ETHICAL AND REGULATORY ASPECTS

The decision of the CEC concerning the conduct of the study will be made in writing to the Sponsor-Investigator before commencement of this study. The clinical study can only begin once approval from all required authorities has been received. Any additional requirements imposed by the authorities shall be implemented.

2.1 Study registration

Registration is planned in ClinicalTrials.gov and the Swiss KOFAM site

2.2 Categorisation of study

The trial is in Risk Category A. There is only minimal risk associated with the trial. The TMS device has CE certification and approved for clinical use. It will be applied according to the manual and guidelines[1, 2]. TMS has been widely used in neuroscience, and in clinical trials on depression, schizophrenia and chronic pain. Participants will receive 15 daily stimulations. Effects are expected to last for 2-4 weeks after the last stimulation. TMS is also safe in repeated administration[1, 2].

Assessments include standard clinical rating scales, short specific tests of motor behaviour, and a standard cerebral magnetic resonance imaging at baseline and after 3 weeks of stimulation. All assessments have been applied to schizophrenia patients before and are generally well tolerated.

2.3 Competent Ethics Committee (CEC)

The local Bern Ethics Committee is the Competent Ethics Committee (CEC). No sites outside the canton of Bern are planned.

The Bern Ethics Committee will receive reports of all changes in the research activity and all unanticipated problems involving risks to humans; including in case of planned or premature study end and the final report. No changes are made to the protocol without prior Sponsor and CEC approval, except where necessary to eliminate apparent immediate hazards to study participants. Premature study end or interruption of the study is reported within 15 days. The regular end of the study is reported to the CEC within 90 days, the final study report shall be submitted within one year after study end. Amendments are reported according to chapter 2.10.

2.4 Competent Authorities (CA)

Not applicable – Category A

2.5 Ethical Conduct of the Study

The study will be carried out in accordance to the protocol and with principles enunciated in the current version of the Declaration of Helsinki, the guidelines of Good Clinical Practice (GCP) issued by ICH, in case of medical device: the European Regulation on medical devices 2017/745 and the ISO Norm 14155 and ISO 14971, the Swiss Law and Swiss regulatory authority's requirements. The CEC and regulatory authorities will receive annual safety and interim reports and be informed about study stop/end in agreement with local requirements.

2.6 Declaration of interest

There is no conflict of interest. Funding is provided by independent grants and the Swiss National Science Foundation (see funding 14.1)

2.7 Patient Information and Informed Consent

Participants will be informed by members of the study team about the aims of the study, planned procedures and risks involved. They will receive written information on the study. This information will be provided prior to study inclusion during screening. The participants will also be informed about the compensation of 200 CHF after they completed the study procedures. The participant will be informed by the investigators that his/her medical records may be examined by authorised individuals other than their treating physician.

All participants for the study will be provided a participant information sheet and a consent form describing the study and providing sufficient information for participant to make an informed decision about their participation in the study. A minimum of 24 h time will be given to participants to decide on

whether to participate or not. Whenever necessary, the potential participant can take up to 2 weeks to decide on participation.

The patient information sheet and the consent form will be submitted to the CEC and to the competent authority (as applicable) to be reviewed and approved. The formal consent of a participant, using the approved consent form, must be obtained before the participant is submitted to any study procedure.

The participant should read and consider the statement before signing and dating the informed consent form, and should be given a copy of the signed document. The consent form must also be signed and dated by the investigator (or his designee) and it will be retained as part of the study records.

2.8 Participant privacy and confidentiality

The investigator affirms and upholds the principle of the participant's right to privacy and that they shall comply with applicable privacy laws. Especially, anonymity of the participants shall be guaranteed when presenting the data at scientific meetings or publishing them in scientific journals.

Individual subject medical information obtained as a result of this study is considered confidential and disclosure to third parties is prohibited. Subject confidentiality will be further ensured by utilising subject identification code numbers to correspond to treatment data in the computer files.

For data verification purposes, authorised representatives of the Sponsor (-Investigator) or an ethics committee may require direct access to parts of the medical records relevant to the study, including participants' medical history.

2.9 Early termination of the study

The Sponsor-Investigator may terminate the study prematurely according to certain circumstances, for example:

- ethical concerns,
- insufficient participant recruitment,
- when the safety of the participants is doubtful or at risk, respectively,
- alterations in accepted clinical practice that make the continuation of a clinical trial unwise,
- early evidence of benefit or harm of the experimental intervention

2.10 Protocol amendments

Substantial amendments are only implemented after approval of the CEC and CA respectively.

Under emergency circumstances, deviations from the protocol to protect the rights, safety and well-being of human subjects may proceed without prior approval of the sponsor and the CEC/CA. Such deviations shall be documented and reported to the sponsor and the CEC/CA as soon as possible.

All non-substantial amendments are communicated to the CA as soon as possible if applicable and to the CEC within the Annual Safety Report (ASR).

3. BACKGROUND AND RATIONALE

3.1 Background and Rationale

3.1.1 Schizophrenia

Schizophrenia is a severe disorder with a life-long course affecting approximately 2-3% of the population. Even though the outcome is heterogeneous, schizophrenia usually causes tremendous individual burden, intense costs for society, reduced quality of life, impaired occupational performance and reduction of life expectancy by 10-20 years[3]. Schizophrenia is an adolescent-onset disorder with neurodevelopmental origins[3]. Current models suggest that genetic risk, early hazards to brain maturation, social adversities during childhood and the evolution of cognitive biases predispose subjects to psychosis in times of stress[4]. The schizophrenia syndrome is characterized by symptom clusters including positive symptoms (e.g. delusions and hallucinations), negative symptoms (e.g. avolition and affective flattening), disorganized thought, motor abnormalities, mood disturbances, impaired cognition, anxiety and lack of insight[5]. Brain structure and function in schizophrenia is abnormal at multiple levels. For example, schizophrenia patients share altered organization of functional brain networks[6] and underlying white matter fiber connections[7, 8]. Therefore, schizophrenia has been conceptualized as a disconnection syndrome, which may explain some of the typical symptoms[9]. Indeed, first evidence indicates an association between abnormal motor behavior and structural as well as functional alterations in the motor network in schizophrenia[10, 11]. Researchers are just starting to understand the network alterations linked to clinical symptoms in schizophrenia[12]. **The ultimate goal** would be to **normalize altered brain function at the network level** in order to **improve the clinical significant behavioral abnormalities**.

3.1.2 Motor system pathology in schizophrenia

A set of motor abnormalities including signs such as bizarre posture, peculiar gait, facial and limb dyskinesia, immobility, rigor, or excessive movement have been reported in schizophrenia.

Motor abnormalities have long been exclusively linked to medication side effects; however, converging evidence demonstrates that motor abnormalities frequently occur before medication is commenced and even long before the onset of psychosis[10, 13-15]. Up to 67% of first episode, treatment naïve patients experience at least one motor sign[16]. The contribution of medication is heterogeneous and probably overestimated.

Motor abnormalities are key cues for stigmatization and pose significant distress on patients[17, 18]. Furthermore, recent evidence supports the predictive value of motor abnormalities in psychosis for poor functional outcome[15, 19]. For example, motor abnormalities impact nonverbal behaviors such as gestures[20], which are critical for social functioning[21]. This way, motor abnormalities contribute to poor functional outcome in schizophrenia.

A large network of the brain is devoted to motor behavior, including cortical frontal motor and premotor areas, the basal ganglia, thalamus, brainstem and cerebellum (see figure 1). Evidence across multiple neuroimaging approaches supports that schizophrenia pathobiology is tied to alterations within the cerebral motor system[22].

Figure 1. Key components of the motor network

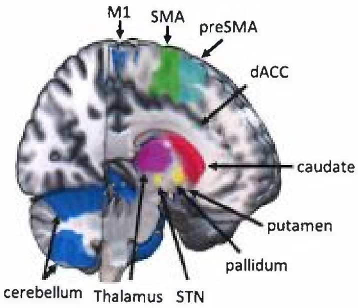

M1	primary motor cortex
SMA	supplementary motor area
pre-SMA	pre-supplementary motor area
dACC	dorsal anterior cingulate cortex
STN	subthalamic nucleus

3.1.3 Psychomotor slowing

One important domain of motor abnormalities in schizophrenia is psychomotor slowing (PS), which can be observed in fine motor behavior such as writing, gross motor behavior such as gait, and it may refer to single movements or continuous movement. PS includes movement planning, initiation, execution, timing and motor control [15, 23-25]. Typical examples of PS in schizophrenia are reduced levels of spontaneous gross motor activity as measured by actigraphy[11, 26-28], slowed gait[29], slowed aiming arm movements[30], slowing in fine motor tasks[26, 31, 32] or in bradykinesia of parkinsonism[31].

Multiple reports suggest that 30-50% of schizophrenia patients present with PS[33, 34]. Furthermore, PS is seen in all stages of the disorder. Even though, PS severity seems to increase with illness chronicity[35], it does also occur in early psychosis or in subjects at risk for psychosis[34, 36-40]. In psychosis, PS is critically linked to several disadvantages[23]. For example, PS is associated with poor cognition, sedentary behavior and cardiometabolic risks[41, 42]. Moreover, PS correlates with distress and poor quality of life[17, 43, 44]. Finally, several reports indicate that PS predicts poor outcome in terms of cognition, quality of life and real world functioning in early psychosis, although at baseline patients with PS were not specifically impaired in function[36, 45-47]. In sum, **PS is a frequently observed phenomenon across schizophrenia stages**, associated with poor quality of life, sedentary behavior and predictive of poor cognition and outcome. In order to design new treatment options to overcome this problematic symptom domain, it is essential to know the pathobiology of PS.

PS is particularly suitable for objective instrumental assessment. There are valid measures of fine motor and gross motor slowing that can be acquired by instrumentation[15]. These measures allow dimensional assessment of PS and are therefore ideal candidates when exploring associations between brain and behavior. Instrumentation is also not prone to conceptual overlap. We have applied wrist actigraphy in a series of studies and repeatedly demonstrated reduced gross motor activity in schizophrenia, which was linked to negative syndrome severity and psychomotor slowing as measured by the Salpêtrière Retardation Rating Scale (SRRS)[26, 48-50]. Likewise, finger tapping test as a measure of PS in fine motor behavior is also consistently poorer in schizophrenia patients and linked to negative symptoms[31]. In addition, the video rated coin rotation test is a simple and reliable measure of manual dexterity[51, 52]. Another interesting marker of motor behavior is increased postural sway, which results from poor cerebellar function and is increased in psychosis[53, 54]. Postural sway was correlated with negative syndrome severity in patients[53, 54]. Therefore, we expect increased postural sway to be associated with PS in schizophrenia. In sum, **PS can be reliably measured by observer ratings** such as the **SRRS** or by **instrumental measures** of gross motor behavior (**activity level** from actigraphy) or fine motor behavior (**finger tapping** and **coin rotation**).

3.1.4 Current treatment options of psychomotor slowing

As mentioned above, motor symptoms including PS are neither simply explained by antipsychotic drug effects[23] nor does PS disappear after antipsychotic drug withdrawal. An excellent study in treatment naïve first episode subjects demonstrated the heterogeneity of drug effects on motor abnormalities, with 30% of patients in whom parkinsonism or catatonia was ameliorated by antipsychotic drugs[55]. Likewise, in many studies of our own group and others we failed to detect a correlation between antipsychotic dosage and measures of PS[11, 26, 31, 49, 50]. However, symptoms tend to improve with treatment but from the naturalistic studies conducted it is currently unclear, whether the improvement is due to a direct effect on motor behavior or whether the benefit results from amelioration of other psychosis symptoms, such as disorganization or avolition[56, 57]. Our own study on the longitudinal course of PS in acute schizophrenia found an amelioration of PS with treatment, which was tightly linked to a decline of negative symptom scores[50]. Finally, there are no trials demonstrating a beneficial effect of antipsychotic medication or trainings to improve psychomotor slowing in schizophrenia. Thus, **alternative treatment options for PS are clearly needed**.

3.1.5 Psychomotor slowing and motor network dysfunction

Conceptually, various aspects of motor behavior are modulated by three key circuits: inhibition and

excitation of movements is related to a circuit including pre-/motor cortex and basal ganglia, timing of movements is linked to another circuit including motor cortex, thalamus and cerebellum, while psychomotor speed and planning involves a cortical network including medial prefrontal cortex, cingulate motor areas, SMA, M1, and posterior parietal cortex[12]. However, PS is not exclusively related to one of the abovementioned behaviors and probably involves all of the three circuits, even though the basal ganglia circuit is the most probable [22].

The current understanding of the pathology in PS is limited due to methodological issues, such as focus on single brain regions, single neuroimaging modalities, and heterogeneous patient samples[10, 22]. Two types of approaches have mainly been adopted: first, actigraphically assessed motor activity levels were correlated with structural and functional magnetic resonance imaging (MRI) markers in the brain. Results suggested that unlike in controls, motor activity levels are not associated with **structural connectivity within the basal ganglia loop** in schizophrenia. Instead, motor activity was linked to **cortical motor loops**, which was interpreted as compensatory mechanism because patients with PS (i.e. lower activity) had particularly lower CBF and reduced white matter integrity within these cortical motor loops[27, 58-60]. Likewise, white matter ultrastructure of the corpus callosum and cingulum mediated psychomotor speed in schizophrenia[61]. A second line of evidence stems from functional and structural MRI studies testing associations with finger movements such as finger tapping, sequential finger-thumb opposition, etc. These studies reported poorer tapping performance and reduced functional activation in SMA, M1, and cerebellum in schizophrenia[62]. Further evidence comes from the few studies on schizophrenia patients with catatonia, who usually present with behavioral motor inhibition. In a resting state perfusion MRI study, my group identified that catatonic schizophrenia is associated with specifically **increased cerebral blood flow (CBF)** in the supplementary motor area (**SMA**) and primary motor cortex (M1) in comparison to schizophrenia patients who had never experienced catatonia before. State CBF values were highest among those patients with severe motor inhibition. However, SMA perfusion was not different between state catatonia and controls[63]. The findings suggest a critical role of the SMA in movement initiation deficits in schizophrenia with state catatonia. However, from these data it was not possible to determine whether SMA hyperperfusion was the result of a motor network pathology that leads to inhibited motor output or whether SMA pathology drives this behavioral inhibition. Furthermore, finger tapping studies in catatonia patients indicated that M1 and SMA were less active during the task[64, 65]. Given the limitations of most previous studies focusing on one task or brain region, the next step must include **a network perspective to understand the pathobiology of motor inhibition as seen in PS**. A first cross-sectional study of my group applied resting state fMRI in order to test functional connectivity within the motor networks in schizophrenia. We found that schizophrenia was characterized by increased connectivity between key regions of the motor network, particularly between thalamus and motor/premotor cortex, M1 and cerebellum, cingulate seeds and STN[11]. Furthermore, the functional abnormalities between M1 and thalamus or cerebellum at rest were linked to observer ratings and instrumental measures of PS in schizophrenia. **Even though there is evidence suggesting that aberrant thalamocortical as well as cerebellar-cortical functional and structural connectivity may contribute to psychomotor slowing, the mechanism is still not entirely clear. This issue needs to be addressed at a network perspective and requires an exploration of the neural effects of interventions targeting psychomotor slowing.**

3.1.6 Intracortical excitability

Transcranial magnetic stimulation (TMS) allows for testing cortical neurophysiology in vivo. Converging evidence supports defective intracortical inhibition in M1 indexed by paired-pulse TMS in all stages of psychosis[66, 67]. Indeed, short-interval intracortical inhibition (SICI) is linked to GABAergic interneuron function. Reduced SICI values indicate **cortical disinhibition** in patients, which correlate with lower fractional anisotropy in motor tracts as well as lower processing speed[68]. Thus **psychomotor slowing** could be associated with **aberrant motor cortex excitability**, i.e. reduced SICI.

3.1.7 Brain stimulation for psychomotor slowing

Repetitive transcranial magnetic stimulation (rTMS) temporarily alters brain function in the targeted cortical areas. In addition, distant effects occur due to changes in network properties[69]. Depending on the frequency and type of stimulation, distinct effects on brain function are expected. Low-frequency rTMS (**lf-rTMS**) and continuous theta burst stimulation (cTBS) have **inhibitory** effects, while high-frequency rTMS (hf-rTMS) and intermittend theta burst (**iTBS**) have **facilitatory** effects. The preliminary knowledge of the pathobiology of PS suggests that rTMS could improve PS by changing aberrant motor network connectivity. Still, there are no published reports on the effects of non-invasive brain stimulation on PS. However, my group is conducting a randomized, double-blind, sham-controlled trial of rTMS for psychomotor slowing in major depressive disorder and schizophrenia (**NCT03275766**, clinicaltrials.gov). Treatment is based on rTMS in 4 arms: 1) facilitatory hf-rTMS over the left dorsolateral prefrontal cortex (DLPFC), 2) inhibitory lf-rTMS over the supplementary motor area (SMA), 3) facilitatory iTBS over the SMA, and 4) sham stimulation with a placebo coil. The primary outcome parameter is the percentage of responders (> 30% reduction in the Salpêtrière Retardation Rating Scale (SRRS) from baseline). Across the whole group of 34 randomized subjects (22 with schizophrenia and 12 with depression), there is a group difference in the number of responders after 15 rTMS sessions applying the last-observation-carried-forward method (Chi^2=11.3, df=3, p=.007) with **78% responders under lf-rTMS over SMA and no responder under iTBS over SMA**. When we exclusively focus on schizophrenia, this effect is also detected (Chi^2=6.6, df=3). Thus, lf-rTMS over SMA ameliorates PS.

The neurophysiologic effects of rTMS treatment can be probed by perfusion MRI for local effects on metabolism, by fMRI on the network level and by TMS paradigms testing changes of cortical excitability of M1. Studies on inhibitory rTMS over the left SMA indicated short-term alterations of functional connectivity from SMA to M1 in healthy subjects along with changes of local metabolic activity in focal dystonia patients and controls[70-72]. While we don't know whether similar effects would occur in patients with altered baseline functional connectivity in the motor network, we may expect to see neural changes (functional connectivity and regional perfusion) following rTMS treatment. Furthermore, single **inhibitory rTMS** for 15 mins over the premotor cortex led to **increased motor cortical excitability** in schizophrenia evidenced by reduced resting motor thresholds and increased motor evoked potentials, supporting our clinical findings of an amelioration of PS[73].

3.1.8 State of research summary

Motor abnormalities are a core feature of psychotic disorders, particularly in the schizophrenia spectrum, indicating poorer outcome[10, 15, 74]. Motor abnormalities are linked to alterations within the cerebral motor networks[22]. One important domain is psychomotor slowing (PS) that impacts gross and fine motor behavior. PS causes distress and functional disability. Moreover, PS can be reliably assessed by instrumentation[15]. But the underlying neurobiology of PS is not well understood. Prior work from correlational studies reported PS to be linked to aberrant functional and structural connectivity between M1 and thalamus or cerebellum[11, 60]. Particularly, patients with severe PS display hyperconnectivity and hyperperfusion in the premotor and motor cortices. Currently, no treatments specifically target PS in schizophrenia. But preliminary data from an rTMS study of my group indicate that inhibitory rTMS over the SMA alleviates PS. In addition, one session of inhibitory rTMS over SMA altered motor cortex excitability in psychosis[73]. **Given, that the motor circuitry is associated with PS and that inhibitory rTMS over SMA may improve PS in schizophrenia, we expect that inhibitory rTMS alters the relevant network for PS in patients**. The combination of resting state fMRI, perfusion MRI, diffusion MRI and rTMS is particularly suited to explore the neural changes in the motor networks[72]. Therefore, we will conduct a prospective RCT of rTMS for PS with neuroimaging assessments at baseline and week 3, focusing on the motor network. Furthermore, we will explore the effects of rTMS on cortical excitability, clinical and functional outcome. This is the first project to enable causal inferences on the pathobiology of PS and further supporting the use of effective rTMS treatment in schizophrenia by **proof of principle**.

3.2 Investigational Product (treatment, device) and Indication

TMS device: MagPro 30 with theta burst option

Manufacturer: MagVenture, Inc. Atlanta GA, USA

Device is intended for a broad range of neuroscientific research in humans.

There is CE conformity according to the ISO Norm 13485:2003.

3.3 Preclinical Evidence

Low-frequency rTMS (1Hz) has been tested in many studies of human neurophysiology, typically as single session rTMS with transient behavioral effects. Lf-rTMS over the SMA alters functional connectivity in the motor system in healthy subjects[72]. Likewise, lf-rTMS over M1 demonstrated increased local neural activity with increasing stimulus intensity in healthy controls, in addition to distant changes in neural activity within M1 connections[75]. Finally, the cortical excitability is altered in healthy subjects following lf-rTMS of M1[76].

3.4 Clinical Evidence to Date

rTMS treatment for psychomotor slowing has only been tested in Parkinson's disease[77, 78] and in a combined group of patients with major depression and schizophrenia (Walther et al. unpublished data). In our own randomized double-blind placebo-controlled trial, 15 sessions of 1 Hz rTMS were effective in ameliorating psychomotor slowing (see also Background). The active comparator iTBS was not effective, but also well-tolerated. There were no exceptional side-effects beyond those regularly reported in studies of rTMS.

The intended protocols (1Hz rTMS and iTBS as active control) have been widely used in neuropsychiatric cohorts to treat various symptoms[2]. Low-frequency rTMS (1Hz) is effective in reducing the severity of auditory verbal hallucinations in schizophrenia spectrum disorders[79-86]. There have been numerous reports demonstrating safety and efficacy of this protocol. The active comparator iTBS has been tested in studies in major depressive disorder and is effective in reducing depression severity with minimal side effects[87, 88]; most commonly transient headaches[2]. rTMS treatment has been applied between 10 and 30 sessions, usually 5 per week. There are international guidelines on the use of rTMS for clinical purposes, including safety measures and side effect assessments[1, 2].

3.5 Dose Rationale / Medical Device: Rationale for the intended purpose in study (pre-market MD)

As outlined in sections 3.1.7., 3.1.8., 3.3., and 3.4. rTMS has been used to modify psychomotor slowing in Parkinson's disease, depression, and schizophrenia. In addition, rTMS over the premotor and motor cortex alters brain function. Our previous study demonstrated clinical effectiveness of 1 Hz stimulation over left SMA on psychomotor slowing in 15 sessions. There are no other treatment studies on motor function in schizophrenia. However, studies applying less than 10 stimulation sessions to treat hallucinations were less effective in schizophrenia spectrum disorders[82, 89]. In major depression, rTMS studies with similar protocols usually treat patients for 4-6 weeks (20-30 sessions)[87].

3.6 Explanation for choice of comparator (or placebo)

This study will have two comparators: one placebo stimulation and an active stimulation that produces the opposite effect of the investigated protocol, i.e. iTBS over SMA which will facilitate neural activity. Both comparators are necessary to demonstrate clinical utility (placebo) and the neurophysiological changes associated with the three treatments (placebo and iTBS). Only this approach will finally allow testing whether inhibitory lf-rTMS is changing the motor system in a specific direction that is causal for clinically relevant changes of motor behavior. The waiting list will disentangle specific stimulation effects from effects of time or expectation. Placebo will disentangle specific rTMS treatment effects. Finally, iTBS will disentangle the direction of neurophysiological changes of the motor system. All stimulations will target the left SMA. Mode of application, localization, frequency of sessions and apparatus will be identical for all protocol arms. Arms will differ in the coil used (real or sham) and in the frequency of the applied stimulation (1 Hz vs. iTBS). The number of pulses is similar in all protocols (1'000-1'200).

3.7 Risks / Benefits

The study intervention is adminstered as an add-on to existing treatment in inpatient (or less often dayclinic) settings. The study procedures pose some extra-effort and burden to the patients,

particularly during baseline and week 3 assessments. However, the time and inconvenience is acceptable and comparable to those of other studies in the field. From our extensive experience with studies in these patient groups we know that assessments are acceptable and may be split to different days if needed. The neuroimaging with MRI twice has a total duration of less than 60 mins per session including the preparation. Most of the scanning time, participants will be in a resting state, i.e. have no task – just to relax lying in the scanner. This can be accomplished by many, even very ill subjects. Only a short proportion of the scanning time is devoted to a simple motor task in the scanner, when participants are asked to move their fingers. Taken together, both clinical and neuroimaging assessments are acceptable.

The study intervention (rTMS) has a minimal risk of increasing the severity of the disorder, i.e. deterioration. In our prior clinical rTMS trial with the same stimulation protocol and 15 rTMS sessions, there was no case of deterioration. Only one SAE occurred which was unrelated to the intervention. In addition, patients are under close observation by the study team and the hospital teams who see patients in the inpatient or dayclinic setting. Thus, changes are rapidly recognized and appropriate measures will be taken, as the study is only an add-on to standard treatment.

This study will follow the general recommendations for safety in rTMS protocols[1, 2] and the device manual. In addition, a screening standard questionnaire for rTMS candiates will be applied to ensure that all strict exclusion criteria for safety in the protocol will be respected. The expected main adverse effects could be transient headache, transient local pain, transient neck pain, transient toothache and transient paraesthesia. Transient hearing changes have not been reported applying theta burst TMS but are likely possible. Therefore hearing protection will be used (earplugs). Furthermore we will make prompt referral for auditory assessment in case of any individual who complains of hearing loss, tinnitus or aural fullness following the rTMS. Seizure induction is rare but possible in epilepsy patients, which are therefore excluded. Furthermore, in some patients transient impairment of working memory was reported. Taken together, if the safety regulations are followed, the risk for participants is minimal. All adverse events are only temporarily occurring and no severe adverse events have been reported in rTMS trials up to now.

Magnetic fields attenuate rapidly with distance, so it seems unlikely that the fetus of a pregnant woman might be directly affected by rTMS. Currently, no side effects to the child were reported in reports of repeated rTMS in pregnant women[90, 91]. Still, pregnant women will be excluded from this study. Prior to study entry, a pregnancy test will be performed by all women in childbearing age, who are also asked to use a simple, effective contraceptive method during the 3 week trial.

Participation in the study does not pose a particular risk for patients, as the rTMS will be added on to the existing standard medical and non-medical interventions. It is likely that patients in the experimental group will experience better outcomes. However, most patients (in control arms) and healthy controls will not have any personal benefit from participation in the study. Still, the study will be an important step towards the design of specific trials to improve psychomotor slowing in schizophrenia. Given, that the experimental arm was superior in ameliorating psychomotor slowing, treatment will result in better outcomes and quality of lives. In addition, results of this study will be used to plan further interventional trials in patients with schizophrenia, which may then introduce rTMS as a standard add-on treatment of psychomotor slowing in schizophrenia spectrum disorders.

3.8 Justification of choice of study population

This study will include a group of patients with schizophrenia spectrum disorders, who will receive the assessments and interventions. In addition, a group of healthy control subjects is included only for the assessments, but not to receive an intervention. Patients will be included if they can provide consent on their own (no minors and no patients incapable of understanding the study information will be recruited). Participation in the study does not interfere with regular treatment. Patients' interests will be safeguarded by the treating physicians who are not part of the study team.

Study of patients is necessary in order to test the rTMS effect in a group with clear psychomotor slowing. These symptoms are only found in patients with severe psychiatric disorders.

Study of controls is needed in order to compare motor behaviour abnormalities and alterations in brain network structure and function between health and disease. Controls will not receive any intervention.

4. STUDY OBJECTIVES

4.1 Overall Objective

This study investigates the neural correlates of psychomotor slowing in schizophrenia spectrum disorders and the neurophysiologic changes associated with successful application of rTMS to ameliorate slowing. To reach this aim, the study combines a randomized, double-blind, placebo-controlled trial of rTMS in patients over 3 weeks and a cross-sectional and longitudinal study focussing on motor behaviour and brain imaging. Therefore, this study has four main aims. First, we aim to **investigate the clinical and functional neural changes following 3 weeks of daily rTMS treatment** (inhibitory, facilitatory, or placebo). An exploratory aim is to describe imaging markers of treatment response. Second, we aim to **characterize the pathophysiology of psychomotor slowing** (PS) in depth combining multimodal neuroimaging and electrophysiology with instrumental and observer-based measures of PS. Third, we aim **to characterize short-term changes of PS** by observing a waiting list cohort that is later allocated to treatment. And fourth, we aim to **describe the clinical outcome** of the rTMS intervention at 6-month follow-up.

4.2 Primary Objective

The study seeks to determine the clinical and neural effects of 15 sessions of inhibitory lf-rTMS over SMA on psychomotor slowing in schizophrenia spectrum disorders compared to facilitatory iTBS, placebo or patients on a waiting list.

4.3 Secondary Objectives

The study further seeks to determine, whether lf-rTMS (inhibitory) will be superior over placebo or iTBS (facilitatory) in all clinical and motor behavioral measures, e.g. greater reduction in BFCRS scores from baseline, greater increase in activity levels, or finger taps. In addition, the study will test the effects of rTMS on the cerebral motor network connectivity and activity, as well as on cerebellar function and motor cortex excitability. Furthermore, the study will explore the neuroimaging markers of response to treatment. Moreover, the study will test the association between measures of PS and neuroimaging markers of the motor system, e.g. network connectivity, activity, and structure. Next, the study will investigate the temporal dynamics of PS by comparing a waiting list group to the placebo group. Finally, the study will test whether lf-rTMS treatment for 3 weeks will have lasting effects on PS at week 6 or 6-months follow-up.

4.4 Safety Objectives

The study will also assess the tolerability of 15 of sessions rTMS in terms of stimulation side effects and duration of stimulation effects.

5. STUDY OUTCOMES

5.1 Primary Outcome

The primary outcome variable is the SRRS score as a surrogate marker of psychomotor slowing. The primary endpoint will be the change SRRS ratings from baseline to week 3 measured with the SRRS[92]. The SRRS is administered at baseline, week 1, week 2, week 3, week 6, and week 24. It is an observer rated scale quantifying the severity of psychomotor slowing with 15 items, each ranging 0-4. The SRRS has been developed to target this behavior and has been used in previous trials. Assessments will be performed by blinded raters, who have been trained to use the scale reliably.

5.2 Secondary Outcomes

5.2.1 Clinical outcomes

Proportion of responders (>= 30% reduction from baseline SRRS) between groups at week 1, 2, 3, 6, and 24. This will provide an additional categorical measure of who benefits from the intervention in terms of the main target (psychomotor slowing).

Change in other commonly observed motor symptoms from baseline, including rating scales on

abnormal involuntary movements, Parkinsonism, and catatonia at week 3, 6, and 24. See section 9 for details on instruments.

Change in general psychopathology using the positive and negative syndrome scale PANSS[93] and the brief negative symptom scale BNSS[94] between baseline and week 3, week 6, and week 24.

Change in self-reported physical activity and experienced negative symptoms using the International Physical Activity Questionnaire (IPAQ)[95] and the self-evaluation of negative symptoms (SNS)[96].

5.2.2 Behavioral outcomes

Change in objective gross motor activity as measured with 24 hours of wrist actigraphy at baseline, week 3, 6, and 24.

Change in fine motor function as measured with the coin rotation task at baseline, week 3, 6, and 24.

5.2.3 Physiological outcomes

Changes in motor cortex excitability from baseline to week 3, 6, and 24 using a TMS paradigm of short interval intracortical inhibition (SICI), intracortical facilitation (ICF) and 1 mV motor evoked potentials (MEP).

Changes in postural sway from baseline at week 3, using the Kistler platform and assessments with eyes open as well as eyes closed.

5.2.4 Neuroimaging outcomes

Change in resting state perfusion within the motor network from baseline to week 3.

Change in motor network resting state connectivity from baseline to week 3.

Change in neural activation patterns during a finger tapping task using functional MRI at baseline and week 3.

5.3 Other Outcomes of Interest

Change in social and community functioning using the global assessment of functioning GAF[97], the Social and Occupational Functioning SOFAS[98], and the UPSA-brief[99] short assessment of functional capacity at baseline, week 6, and week 24. Social and community functioning is a relevant distal outcome for any psychiatric intervention.

5.4 Safety Outcomes

After each rTMS session, participants are inquired about stimulation side effects. After sessions 5, 10 and 15, we will apply a short rating scale to assess side effects according to the guidelines[1, 2].

6. STUDY DESIGN

6.1 General study design and justification of design

The design of this single-site study is a 4 parallel-arm, double-blind, randomized, placebo-controlled trial. Patients will be randomized to one of four treatment arms. Patients will all receive TMS over the left SMA in 15 daily sessions over 3 weeks as add-on treament. In three groups, assessments will be conducted immediately before and after the three weeks treatment (1 Hz, iTBS, and Placebo), while the fourth group will receive active treatment (1Hz) only after a 3 week waiting period with no additional intervention. After 6 months we will perform a clinical follow-up assessment. The interventions will be conducted by a small team (2 persons) who will know the stimulation parameters. All assessments will be conducted by a different team, who is completely blind to treatment and specifically trained to use the instruments and rating scales. Patients are also blind to treatment, because stimulation site is the same for all groups and there will be no change of protocols during the study. The placebo group will be stimulated with a placebo coil that looks identical and emits identical noises compared to the real TMS coil, but without any magnetic impulse emission. Assessments with clinical rating scales will be complemented by objective and instrumental motor tests that are not prone to rater bias. Therefore, both the patients and the assessors are blind to treatment protocol.

The study will include patients with schizophrenia spectrum disorders who currently have psychomotor slowing according to the SRRS. The protocol arms are chosen to test the clinical efficacy and neurophysiological changes of 15 sessions 1 Hz rTMS for psychomotor slowing in schizophrenia spectrum disorders. The placebo-arm is required to demonstrate an effect of rTMS as add-on treatment. The active control is required to demonstrate an opposite effect at the neural level compared to active treatment. Finally, the waiting list is important to characterize potential self-limitation of the condition under study. Because these subjects are referred to a waiting list, they will receive active treatment thereafter. This group will not be completely blinded, as they will know to be in the active treatment group from week 3 through week 6.

We intend to enrol 88 patients with schizophrenia spectrum disorders. For the neuroimaging and motor behavioural assessments, we also plan to include 40 matched healthy control subjects.

For each patient in group 1-3 the study is 3 weeks and a follow-up interview after 6 months. Patients in group 4 (waiting list) will have three assessments (baseline, week 3 and week 6) and a follow-up interview at 6 months. The total duration of the study is 4 years.

6.2 Methods of minimising bias

6.2.1 Randomisation

Using the free software research randomizer, we will generate for the patients a list with four numbers indicating the group allocation, i.e. stimulation types. Allocation will be 1:1:1:1 across the whole intended sample. Subsequent numbers of study inclusion will therefore determine to which study arm the patient will be randomized. The lists will only be available for the principal investigator and locked in his office. He will perform randomization and give the written group allocation to the investigators.

6.2.2 Blinding procedures

As described in 6.1, patients will receive 15 sessions of rTMS over the left SMA in a blinded fashion. They will not be able to see which protocol is used (coil placement on top of their head, machine display behind the patients). Because of the parallel arm design, they will not know how the other protocols feel. Patients will be eye-blinded and ear-plugged during rTMS.

The teams performing assessments and rTMS stimulations will be strictly distinct. Therefore, patients and outcome assessors will be blinded to treatment arm.

6.2.3 Other methods of minimising bias

Outcomes are assessed with instrumental means or validated questionnaires. All assessments will follow a standard routine. Assessors will be trained to use instruments by the PI.

6.3 Unblinding Procedures (Code break)

In case of severe adverse events, an unblinding can take place at the responsibility of the principal investigator. In this case, the participant will not be able to further participate in the study. The allocated intervention order will be kept in a sealed envelope. The sealed envelopes will be stored at a central place, i.e. the office of the lab, so they can be accessed in case of any emergency.

7. STUDY POPULATION

The aim is to test 88 subjects with schizophrenia spectrum disorders and 40 healthy control subjects. Both genders will be included, age range 18 – 65 years.

7.1 Eligibility criteria

Participants fulfilling all of the following **inclusion** criteria are eligible for the study:

- ages 18–60 years
- Right-handed subjects
- Ability and willingness to participate in the study
- Ability to provide written informed consent
- Informed Consent as documented by signature (Appendix Informed Consent Form)

- **Patients only**: schizophrenia spectrum disorders according to DSM-5 **with** psychomotor slowing (SRRS score ≥ 15)

The presence of any one of the following **exclusion** criteria will lead to exclusion of the participant:

- Substance abuse or dependence other than nicotine
- Past or current medical or neurological condition associated with impaired or aberrant movement, such as brain tumors, stroke, M. Parkinson, M. Huntington, dystonia, or severe head trauma with subsequent loss of conciousness.
- Epilepsy or other convulsions
- History of any hearing problems or ringing in the ears
- Standard exclusion criteria for MRI scanning and TMS; e.g. metal implants, claustrophobia
- Patients only: any TMS treatment in the past 3 months
- Women who are pregnant or breast feeding,
- Intention to become pregnant during the course of the study,
- Female participants who are surgically sterilised / hysterectomised or post-menopausal for longer than 2 years are not considered as being of child bearing potential.
- Previous enrolment into the current study,
- Enrolment of the investigator, his/her family members, employees and other dependent persons
- **Controls only**: history of any psychiatric disorder or first-degree relatives with schizophrenia spectrum disorders.

7.2 Recruitment and screening

Healthy participants will be recruited by word-of-mouth, an internet link at the homepage of the department and flyers at supermarkets or at the University of Bern. Staff of the University Hospital of Psychiatry Bern will not be recruited. Furthermore, patients will be asked for participation at the inpatient and outpatient departments of the University Hospital of Psychiatry, Bern. All participants will spend approximately a total of 18 hours in the study on 7 different assessment days (screening, baseline, 3x during interventions and 2 follow-ups). Thus, they will be compensated by a single payment of CHF 200,-. Screening is described in section 9.1 and performed by master-level psychologists or psychiatrists.

7.3 Assignment to study groups

Randomization procedure is described in section 6.2.1. When the investigators have recruited a patient-participant, the principal investigator will allocate the person to a study group based on the randomization list and the sequence of enrolment in the study. From the list of numbers, patients are sequentially allocated by the principal investigator to one of the four treatment arms. The principal investigator will provide written information to the investigators concerning the treatment arm. The lists with the group allocations are only accessible for the principal investigator.

7.4 Criteria for withdrawal / discontinuation of participants

Participants may discontinue the trial at any time or withdraw consent. Furthermore, the treating physicians may request study discontinuation in case of significant deterioration of the condition at any time during the intervention period. All data from subjects in the intent-to-treat population, who received at least one dose of rTMS will be further used in the analyses. We will apply the last-observation-carried-forward (LOCF) method in the final analyses. In case a patient withdraws consent before the first rTMS stimulation, we will recruit an additional patient. The number of all patients recruited and randomized will be reported in the publications. All data from neuroimaging acquisition and motor behaviour will be used whenever possible, e.g. for analyses of baseline associations between brain imaging and behavior. In case of withdrawal due to adverse events or serious adverse events there will be follow-up examinations after 14 days and repeatedly until the problem is resolved, see 9.2.5.

8. STUDY INTERVENTION

8.1 Identity of Investigational Products (treatment / medical device)

8.1.1 Experimental Intervention (treatment / medical device)

Name: MagPro R30 with theta burst option

Manufacturer: MagVentrue, Inc. Atlanta GA, USA

The device is CE certified according to ISO-Norm 13485:2003 and approved for clinical use.

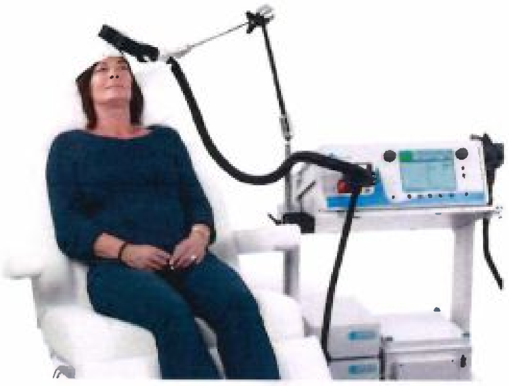

Active stimulation: 1 Hz stimulation of 17 mins over left SMA, 15 sessions in total (5 per week)

8.1.2 Control Intervention (standard/routine/comparator treatment / medical device)

All stimulations are delivered with the same device. The stimulation sites are the same (left SMA) for all groups. The iTBS protocol is shorter (3 mins), the placebo protocol will be delivered with a specific placebo-coil that looks identical, makes identical sounds but produces no magnetic pulses.

Placebo control: 1 Hz stimulation of 17 mins over left SMA without magnetic emission, 15 sessions in total (5 per week)

Active control: iTBS stimulation over left SMA, 21 mins per session, 15 sessions in total (5 per week)

Waiting list: no intervention during the first three weeks, afterwards active stimulation: 1 Hz stimulation of 17 mins over left SMA, 15 sessions in total (5 per week)

8.1.3 Packaging, Labelling and Supply (re-supply)

Not applicable.

8.1.4 Storage Conditions

TMS devices are stored in the institution (translational research center at the University Hospital of Psychiatry, Bern) according to internal regulations and the device manual.

8.2 Administration of experimental and control interventions

8.2.1 Experimental Intervention

The application of the TMS device follows the published guidelines[1, 2] and the device manual. Before each session, the resting motor threshold is determined to identify the individual intensity of stimulation. The position for the coil placement is left SMA, which is determined either via neuronavigation using individual brain anatomy or 3 cm anterior of the leg motor area, which is individually determined through single pulse stimulations causing leg movements.

Both experimenter and participants will wear earplugs for auditory safety.

Active experimental protocol:

Lf-rTMS at 1Hz will be used with 1'000 pulses at an intensity of 110% of the resting motor threshold (approximate duration 17 minutes). The protocol is identical to that of our previous study and a study in Parkinson's disease[77]. 15 sessions in total (5 per week)

8.2.2 Control Intervention

Placebo control: 1 Hz stimulation of 17 mins over left SMA without magnetic emission, using the Placebo-coil. 15 sessions in total (5 per week)

Active control: Stimulation parameters for iTBS will follow those of Huang et al.[100], including 600 pulses at 50 Hz (stimulation in 2 sec trains every 10 sec for 190 sec in total). To increase the effect, we will deliver two iTBS series in one session separated by 15 min with a total of 1200 pulses. During the experiment, iTBS pulse intensity is adjusted to 80% of the motor threshold. iTBS stimulation of 21 mins over left SMA, 15 sessions in total (5 per week)

Waiting list: same protocol as active experimental protocol but only between week 3 and 6.

8.3 Dose / Device modifications

In case of intolerable side effects, the study will be discontinued for the participant. No dose or device modifications are planned.

8.4 Compliance with study intervention

Investigators have to document the order of stimulation. This will be checked for consistency with the group allocation. No further strategies are needed. Participants are always under observation during the rTMS sessions and the planned assessments. Non-compliance on the side of the participant would lead to study discontinuation for this person. Use of concomitant medication will be retrieved from the medical files of the patients and transferred to the CRF.

8.5 Data Collection and Follow-up for withdrawn participants

If participants withdraw their consent, they will be contacted immediately to clarify whether there were intolerance issues with the study. See section 9.2.5 for safety follow-up measures. In case of withdrawal the data will be analyzed and stored encrypted as all the data of the other participants. However, we will remove the encryption key for the participants who withdrew consent and therefore it will not be possible to identify the subjects from encrypted data. Patients who withdrew consent will be invited for the 6 month follow-up interview using their preferred way of contact (Email, letter).

8.6 Trial specific preventive measures

No specific preventive measures are needed.

8.7 Concomitant Interventions (treatments)

Current medication will be recorded in the CRF at study entry and throughout the 3 week intervention period. Patients are expected to comply with the treatment they are receiving from their treating physicians.

Because this is an add-on treatment, we expect relevant changes from concomitant interventions. Therefore, we apply placebo, waiting-list, and an active control group.

8.8 Study Drug / Medical Device Accountability

Not applicable.

8.9 Return or Destruction of Study Drug / Medical Device

The TMS devices are already in use in the clinic. No study specific procedures are intended.

9. STUDY ASSESSMENTS

9.1 Study flow chart(s) / table of study procedures and assessments

Flow charts are distinct for controls and patients, because controls receive no intervention. Furthermore, three patient groups will start interventions immediately (lf-rTMS, iTBS, and Placebo; i.e. groups 1-3), but one group will be waiting for 3 weeks and then enter the intervention phase (waiting group; i.e. group 4).

9.1.1 Patients of treatment groups 1-3

Study Periods	Screening	Treatment, Intervention Period				Follow-up	
Visit	1	2	3	4	5	6	7
Time (week)	-1	0	1	2	3	6	24
Patient Information and Informed Consent	x						
Demographics	x						
CASH and SCID (history)	x						
In- /Exclusion Criteria	x						
Physical Examination	x						
Pregnancy Test	x						
Psychopathology (TALD, NES)		x					
Randomisation		x					
Administer rTMS (5 sessions per week)		x	x	x			
Primary Variable SRRS		x	x	x	x	x	x
Motor scales UPDRS, BFCRS, AIMS, IPAQ		x			x	x	x
Psychopathology (PANSS, BNSS, SNS)		x			x	x	x
Actigraphy and coin rotation		x			x	x	x
Posturography		x			x		
TMS cortical excitability		x			x	x	x
Cerebral MRI		x			x		
Functional outcome (SOFAS, GAF, UPSA-brief)		x				x	x
Concomitant Therapy		x	x	x	x		
Adverse Events		x	x	x	x		

Study events patient groups 1-3

**Visit**	**Information**	**Instrument used**	**Expected time**
1 (Screening)	Check inclusion / exclusion criteria	CRF	5 min
	Demographic data	CRF	5 min
	Medical history	CRF	10 min
	Handedness	Edinburgh Handedness Inventory[101]	5 min
	Pregnancy test	Urine based test	10 min
	Diagnoses ascertainment	SCID interview	60 min
2 (baseline)	Psychopathology	Positive And Negative Syndrome Scale (PANSS)[93] interview, CASH[102] interview, Brief Negative Symptom Scale (BNSS)[94],	90 min

		Thought and Language Disorder (TALD)[103], Neurological Evaluation Scale (NES)[104]	
	Primary outcome	SRRS[92]	5 min
	Motor scales	Unified Parkinson's Disease Rating Scale (UPDRS)[105], Bush Francis Catatonia Rating Scale (BFCRS)[106], Abnormal Involuntary Movement Scale (AIMS)[107]	15 min
	Self Report	Self-Evaluation of negative symptoms (SNS)[96], International Physical Actitvity Questionnaire (IPAQ)[95]	10 min
	Motor tests	Actigraphy and coin rotation	5 min
	Posturography	Kistler platform at Dept. of Neurology	30 min
	Cortical excitability	TMS paradigm with paired pulse stimulation	30 min
	Neuroimaging	MRI scan including structural T1, Arterial spin labelling, BOLD resting state and task fMRI	60 min
	Functional outcome	Global Assessment of Functioning GAF, Social and Occupational Functioning Scale SOFAS, UPSA-brief assessment of functional capacity	15 min
	rTMS	5 rTMS daily sessions à 20 min	100 min
3 (week 1)	Primary outcome	SRRS	5 min
	rTMS	5 rTMS daily sessions à 20 min	100 min
	Check side effects	CRF	5 min
	Concomitant therapy	CRF	5 min
4 (week 2)	Primary outcome	SRRS	5 min
	rTMS	5 rTMS daily sessions à 20 min	100 min
	Check side effects	CRF	5 min
	Concomitant therapy	CRF	5 min
5 (week 3)	Psychopathology	Positive And Negative Syndrome Scale (PANSS)[93] interview, Brief Negative Symptom Scale (BNSS)[94]	30 min
	Primary outcome	SRRS[92]	5 min
	Motor scales	Unified Parkinson's Disease Rating Scale (UPDRS)[105], Bush Francis Catatonia Rating Scale (BFCRS)[106], Abnormal Involuntary Movement Scale (AIMS)[107]	15 min
	Self Report	Self-Evaluation of negative symptoms (SNS)[96], International Physical Actitvity Questionnaire (IPAQ)[95]	10 min
	Motor tests	Actigraphy and coin rotation	5 min

	Posturography	Kistler platform at Dept. of Neurology	30 min
	Cortical excitability	TMS paradigm with paired pulse stimulation	30 min
	Neuroimaging	MRI scan including structural T1, Arterial spin labelling, BOLD resting state and task fMRI	60 min
	Functional outcome	Global Assessment of Functioning GAF, Social and Occupational Functioning Scale SOFAS, UPSA-brief assessment of functional capacity	15 min
	Check side effects	CRF	5 min
	Concomitant therapy	CRF	5 min
6 (week 6)	Psychopathology	Positive And Negative Syndrome Scale (PANSS)[93] interview, Brief Negative Symptom Scale (BNSS)[94]	30 min
	Primary outcome	SRRS[92]	5 min
	Motor scales	Unified Parkinson's Disease Rating Scale (UPDRS)[105], Bush Francis Catatonia Rating Scale (BFCRS)[106], Abnormal Involuntary Movement Scale (AIMS)[107]	15 min
	Self Report	Self-Evaluation of negative symptoms (SNS)[96], International Physical Actitvity Questionnaire (IPAQ)[95]	10 min
	Motor tests	Actigraphy and coin rotation	5 min
	Cortical excitability	TMS paradigm with paired pulse stimulation	30 min
	Functional outcome	Global Assessment of Functioning GAF, Social and Occupational Functioning Scale SOFAS, UPSA-brief assessment of functional capacity	15 min
7 (week 24)	Psychopathology	Positive And Negative Syndrome Scale (PANSS)[93] interview, Brief Negative Symptom Scale (BNSS)[94]	30 min
	Primary outcome	SRRS[92]	5 min
	Motor scales	Unified Parkinson's Disease Rating Scale (UPDRS)[105], Bush Francis Catatonia Rating Scale (BFCRS)[106], Abnormal Involuntary Movement Scale (AIMS)[107]	15 min
	Self Report	Self-Evaluation of negative symptoms (SNS)[96], International Physical Actitvity Questionnaire (IPAQ)[95]	10 min
	Motor tests	Actigraphy and coin rotation	5 min
	Cortical excitability	TMS paradigm with paired pulse stimulation	30 min
	Functional outcome	Global Assessment of Functioning GAF, Social and Occupational Functioning	15 min

		Scale SOFAS, UPSA-brief assessment of functional capacity	

9.1.2 Patients of treatment group 4 (waiting group)

Study Periods	Screening	Treatment, Intervention Period					Follow-up	
Visit	1	2	3	4	5	6	7	8
Time (week)	-1	0	3	4	5	6	9	24
Patient Information and Informed Consent	x							
Demographics	x							
CASH and SCID (history)	x							
In- /Exclusion Criteria	x							
Physical Examination	x							
Pregnancy Test	x							
Psychopathology (TALD, NES)		x						
Randomisation		x						
Administer rTMS (5 sessions per week)			x	x	x			
Primary Variable SRRS		x	x	x	x	x	x	x
Motor scales UPDRS, BFCRS, AIMS, IPAQ		x	x			x	x	x
Psychopathology (PANSS, BNSS, SNS)		x	x			x	x	x
Actigraphy and coin rotation		x	x			x	x	x
Posturography		x	x			x		
TMS cortical excitability		x	x			x	x	x
Cerebral MRI		x	x			x		
Functional outcome (SOFAS, GAF, UPSA-brief)		x	x				x	x
Concomitant Therapy		x	x	x	x	x		
Adverse Events			x	x	x	x		

Study events for patients of the waiting group

**Visit**	**Information**	**Instrument used**	**Expected time**
1 (Screening)	Check inclusion / exclusion criteria	CRF	5 min
	Demographic data	CRF	5 min
	Medical history	CRF	10 min
	Handedness	Edinburgh Handedness Inventory[101]	5 min
	Pregnancy test	Urine based test	10 min
	Diagnoses ascertainment	SCID interview	60 min
2 (baseline)	Psychopathology	Positive And Negative Syndrome Scale (PANSS)[93] interview, CASH[102] interview, Brief Negative Symptom Scale (BNSS)[94], Thought and Language Disorder (TALD)[103], Neurological Evaluation Scale (NES)[104]	90 min

	Primary outcome	SRRS[92]	5 min
	Motor scales	Unified Parkinson's Disease Rating Scale (UPDRS)[105], Bush Francis Catatonia Rating Scale (BFCRS)[106], Abnormal Involuntary Movement Scale (AIMS)[107]	15 min
	Self Report	Self-Evaluation of negative symptoms (SNS)[96], International Physical Actitvity Questionnaire (IPAQ)[95]	10 min
	Motor tests	Actigraphy and coin rotation	5 min
	Posturography	Kistler platform at Dept. of Neurology	30 min
	Cortical excitability	TMS paradigm with paired pulse stimulation	30 min
	Neuroimaging	MRI scan including structural T1, Arterial spin labelling, BOLD resting state and task fMRI	60 min
	Functional outcome	Global Assessment of Functioning GAF, Social and Occupational Functioning Scale SOFAS, UPSA-brief assessment of functional capacity	15 min
3 (week 3)	Psychopathology	Positive And Negative Syndrome Scale (PANSS)[93] interview, CASH[102] interview present part only, Brief Negative Symptom Scale (BNSS)[94], Thought and Language Disorder (TALD)[103], Neurological Evaluation Scale (NES)[104]	90 min
	Primary outcome	SRRS[92]	5 min
	Motor scales	Unified Parkinson's Disease Rating Scale (UPDRS)[105], Bush Francis Catatonia Rating Scale (BFCRS)[106], Abnormal Involuntary Movement Scale (AIMS)[107]	15 min
	Self Report	Self-Evaluation of negative symptoms (SNS)[96], International Physical Actitvity Questionnaire (IPAQ)[95]	10 min
	Motor tests	Actigraphy and coin rotation	5 min
	Posturography	Kistler platform at Dept. of Neurology	30 min
	Cortical excitability	TMS paradigm with paired pulse stimulation	30 min
	Neuroimaging	MRI scan including structural T1, Arterial spin labelling, BOLD resting state and task fMRI	60 min
	Functional outcome	Global Assessment of Functioning GAF, Social and Occupational Functioning Scale SOFAS, UPSA-brief assessment of functional capacity	15 min
	rTMS	5 rTMS daily sessions à 20 min	100 min
4 (week 4)	Primary outcome	SRRS	5 min

	rTMS	5 rTMS daily sessions à 20 min	100 min
	Check side effects	CRF	5 min
	Concomitant therapy	CRF	5 min
5 (week 5)	Primary outcome	SRRS	5 min
	rTMS	5 rTMS daily sessions à 20 min	100 min
	Check side effects	CRF	5 min
	Concomitant therapy	CRF	5 min
6 (week 6)	Psychopathology	Positive And Negative Syndrome Scale (PANSS)[93] interview, Brief Negative Symptom Scale (BNSS)[94]	30 min
	Primary outcome	SRRS[92]	5 min
	Motor scales	Unified Parkinson's Disease Rating Scale (UPDRS)[105], Bush Francis Catatonia Rating Scale (BFCRS)[106], Abnormal Involuntary Movement Scale (AIMS)[107]	15 min
	Self Report	Self-Evaluation of negative symptoms (SNS)[96], International Physical Actitvity Questionnaire (IPAQ)[95]	10 min
	Motor tests	Actigraphy and coin rotation	5 min
	Posturography	Kistler platform at Dept. of Neurology	30 min
	Cortical excitability	TMS paradigm with paired pulse stimulation	30 min
	Neuroimaging	MRI scan including structural T1, Arterial spin labelling, BOLD resting state and task fMRI	60 min
	Functional outcome	Global Assessment of Functioning GAF, Social and Occupational Functioning Scale SOFAS, UPSA-brief assessment of functional capacity	15 min
	Check side effects	CRF	5 min
	Concomitant therapy	CRF	5 min
7 (week 9)	Psychopathology	Positive And Negative Syndrome Scale (PANSS)[93] interview, Brief Negative Symptom Scale (BNSS)[94]	30 min
	Primary outcome	SRRS[92]	5 min
	Motor scales	Unified Parkinson's Disease Rating Scale (UPDRS)[105], Bush Francis Catatonia Rating Scale (BFCRS)[106], Abnormal Involuntary Movement Scale (AIMS)[107]	15 min
	Self Report	Self-Evaluation of negative symptoms (SNS)[96], International Physical Actitvity Questionnaire (IPAQ)[95]	10 min

	Motor tests	Actigraphy and coin rotation	5 min
	Cortical excitability	TMS paradigm with paired pulse stimulation	30 min
	Functional outcome	Global Assessment of Functioning GAF, Social and Occupational Functioning Scale SOFAS, UPSA-brief assessment of functional capacity	15 min
8 (week 24)	Psychopathology	Positive And Negative Syndrome Scale (PANSS)[93] interview, Brief Negative Symptom Scale (BNSS)[94]	30 min
	Primary outcome	SRRS[92]	5 min
	Motor scales	Unified Parkinson's Disease Rating Scale (UPDRS)[105], Bush Francis Catatonia Rating Scale (BFCRS)[106], Abnormal Involuntary Movement Scale (AIMS)[107]	15 min
	Self Report	Self-Evaluation of negative symptoms (SNS)[96], International Physical Actitvity Questionnaire (IPAQ)[95]	10 min
	Motor tests	Actigraphy and coin rotation	5 min
	Cortical excitability	TMS paradigm with paired pulse stimulation	30 min
	Functional outcome	Global Assessment of Functioning GAF, Social and Occupational Functioning Scale SOFAS, UPSA-brief assessment of functional capacity	15 min

9.1.3 Control subjects

Study Periods	Screening	Observation period	
Visit<br>Time (week)	1<br>-1	2<br>0	3<br>3
Proband Information and Informed Consent	x		
Demographics	x		
SCID	x		
In- /Exclusion Criteria	x		
Physical Examination	x		
Pregnancy Test	x		
Primary Variable SRRS		x	x
Motor scales UPDRS, BFCRS, AIMS, IPAQ		x	x
Actigraphy and coin rotation		x	x
Posturography		x	x
TMS cortical excitability		x	x
Cerebral MRI		x	x

Study events for controls

Visit	Information	Instrument used	Expected time
1 (Screening)	Check inclusion / exclusion criteria	CRF	5 min
	Demographic data	CRF	5 min
	Medical history	CRF	10 min
	Handedness	Edinburgh Handedness Inventory[101]	5 min
	Pregnancy test	Urine based test	10 min
	Diagnoses ascertainment	SCID interview	60 min
2 (week 0)	Primary outcome	SRRS[92]	5 min
	Motor scales	Unified Parkinson's Disease Rating Scale (UPDRS)[105], Bush Francis Catatonia Rating Scale (BFCRS)[106], Abnormal Involuntary Movement Scale (AIMS)[107]	15 min
	Self Report	International Physical Actitvity Questionnaire (IPAQ)[95]	5 min
	Motor tests	Actigraphy and coin rotation	5 min
	Posturography	Kistler platform at Dept. of Neurology	30 min
	Cortical excitability	TMS paradigm with paired pulse stimulation	30 min
	Neuroimaging	MRI scan including structural T1, Arterial spin labelling, BOLD resting state and task fMRI	60 min
3 (week 3)	Primary outcome	SRRS[92]	5 min
	Motor scales	Unified Parkinson's Disease Rating Scale (UPDRS)[105], Bush Francis Catatonia Rating Scale (BFCRS)[106], Abnormal Involuntary Movement Scale (AIMS)[107]	15 min
	Self Report	International Physical Actitvity Questionnaire (IPAQ)[95]	5 min
	Motor tests	Actigraphy and coin rotation	5 min
	Posturography	Kistler platform at Dept. of Neurology	30 min
	Cortical excitability	TMS paradigm with paired pulse stimulation	30 min
	Neuroimaging	MRI scan including structural T1, Arterial spin labelling, BOLD resting state and task fMRI	60 min

9.2 Assessments of outcomes

Assessors of outcome variables will be psychologists or psychiatrists of master-degree-level. All assessors will be trained by the PI to use the instruments correctly and to assure interrater reliability. Weekly staff meetings will ensure conformity in procedures. All assessments will be conducted blind to

rTMS treatment.

9.2.1 Assessment of primary outcome

The Salpêtrière Retardation Rating Scale (SRRS) is applied at each visit from baseline to follow-up. The scale is described in section 5.1. Raters will be trained to use the scale and blind to rTMS treatment. The score of each of the 15 items is recorded in the CRF.

9.2.2 Assessment of secondary outcomes

9.2.2.1 Clinical outcomes

The change in motor syndromes from baseline is assessed at several visits (see 9.1). Trained raters blind to treatment will assess motor behaviour in standardized examinations with clinical rating scales. Parkinsonism is assess with the Unified Parkinson's Disease Rating Scale (UPDRS)[105], of which we will only assess part III, the current motor behaviour. Catatonia will be assessed with the Bush Francis Catatonia Rating Scale (BFCRS)[106], an 24 item rating scale specifically designed for this purpose. The timeframe of observation is the last week. Finally, dyskinesia will be monitored with the standard rating scale for this issue, the Abnormal Involuntary Movement Scale (AIMS)[107], a 7 item scale following a standardized examination. All motor rating scales are the gold standard of their kind.

The change of general schizophrenia symptoms is assessed with the Positive And Negative Syndrome Scale (PANSS)[93]. The 30 item scale is the widely used standard assessment following a standardized clinical interview. Change in negative symptoms is specifically assessed with the Brief Negative Symptom Scale (BNSS)[94], which allows monitoring relevant dimensions of negative symptoms such as apathy and diminished expression. Furthermore, we will apply the self-evaluation of negative symptoms (SNS)[96], a valid and reliable 20 items questionnaire to capture the subjective experience of negative symptoms.

9.2.2.2 Behavioral outcomes

Objective gross motor behaviour will be assessed using continuous wrist actigraphy for 24 hours. The actigraphs (empathica e4) will be worn on the wrist of the non-dominant arm. Data is stored as logged electronic file. This measure is sensitive to altered motor behaviour and has been successfully applied in many studies of Prof. Walther's team[27, 35, 49, 50, 108-111]. Patients will fill a sleep activity protocol to enable separation of sleep from wake periods during recording. This also allows to check data for consistency and plausibility, because measurements are continuously performed also in periods when participants are not observed.

The fine motor performance is assessed with the coin rotation task. In this task, participants are asked to rotate a .50 CHF coin between thumb, index and middle finger as fast as possible for a total of 30 seconds. The performance is recorded on video and later analysed offline. Analysis includes the number of half turns and the number of coin drops according to a validated formula[51, 52, 112].

Self-ratings of physical activity will be conducted with the 7-item International Physical Activity Questionnaire (IPAQ)[95], which has a German Version and has great psychometric properties. The ratings cover the past 7 days and allow calculating the energy expenditure and total activity.

9.2.2.3 Physiological outcomes

Measures of cortical excitability will be assessed as one of the important physiological outcome parameters. Measurements will be conducted with a MagPro R30 (MagVentrue, Inc. Atlanta GA, USA). Single pulse and paired pulse TMS protocols will be conducted to measure short-interval intracortical inhibition (SICI) at 1 msec interstimulus interval (ISI) and 3msec ISI, intracortical facilitation (ICF) at 7 msec ISI and 15 ms ISI, resting motor threshold (RMT), and 1 mV motor evoked potential (MEP), according to standard protocols[67, 113]

Posturography will be conducted at the Department of Neurology (Prof. Roger Kalla). The assessments of postural sway will be scheduled immediately before or after the MRI acquisition, because this is also located at the Inselspital Bern. Mean postural sway will be calculated as outcome variable for postural stability[53, 114]. The Kistler platform will be used to calculate of the pressure dependent fluctuation (x-, y-, z- axis) of the bodies' centre of gravity[115]. Participants will be measured standing with eyes open and eyes closed according to standard procedures.

9.2.2.4 Neuroimaging outcomes

Neuroimaging will be acquired twice, at baseline and week 3. MRI acquisition will be performed at a

3T Siemens Magnetom Trio scanner at the Institute of Diagnostic and Interventional Neuroradiology, Bern (local collaborator Prof. Dr. med. Roland Wiest). The 64-channel head coil will be used for all MRI images. First, high-resolution T1-weighted MR images will be obtained using a 3D magnetization-prepared rapid two-gradient-echo with 2 inversion times (MP2RAGE) sequence. fMRI will be performed using a multi-slice multi-band T2*-weighted echo planar imaging (EPI) sequence for resting state and during task execution. Prior to the fMRI images a B0 will be acquired for corrections of putative field inhomogeneity. Afterwards, we will perform acquisition of cerebral blood flow (CBF) at rest using a pseudo-continuous arterial spin labelling (pCASL) sequence. Moreover, we will acquire a M0 image (M0 equilibrium magnetization of water signal) for the quantification of CBF and a B0 for corrections of putative field inhomogeneity. Finally, a set of diffusion-weighted images (DWI) that will allow reconstructing fiber tracts using the model based on diffusion tensor imaging (DTI).

Anatomy (MP2RAGE) The optimized acquisition parameters were as follows: 176 sagittal slices, 256 × 224 matrix (with a non-cubic field of view (FOV) of 256 × 224 mm2, yielding a nominal isotropic resolution of 1 mm3), 5000 ms repetition time (TR), 2.98 ms echo time (TE), 700 ms and 2500 ms inversion time (TI), flip angle 4° and 5°, GRAPPA acceleration factor 3 and a 8:22 min total acquisition time.

B0-map - The 48 EPI interleaved axial oblique slices will be positioned exactly like the fMRI slices with exactly the same slice geometry. Two amplitude and a phase image will be recorded in each subject (TR = 520 ms, TE1 = 4.92 ms, TE2 = 7.38 ms). The acquisition time will last 1 min 40 sec.

fMRI - The 48 EPI interleaved axial oblique slices will be positioned in-line with the bi-commissural axis with the following parameters: TR = 500 ms, TE = 30 ms, FA = 90, slice thickness = 3.6 mm, gap thickness = 0 mm, matrix size = 64 x 64 mm, FOV 230 x 230 mm2 resulting in a iso-voxel dimension of 3.6 mm x 3.6 mm x 3.6 mm. The sequence is driven in a 3D PACE mode (Siemens Erlangen) to enable prospective motion correction. With these sequence parameters we will cover the whole brain including the cerebellum. In total, first 720 dynamic scans will be collected for resting state fMRI (total of 6 mins) and subsequently 1200 dynamic scans will be collected during the conduction of the task (total of 10 mins). The task will consist of four blocks including two active paced blocks, a passive listening block and rest. All blocks except rest will be paced with a continuous 2Hz auditory cue. The two active blocks are **right hand finger tapping** (index finger vs. thumb) and **sequential finger-thumb opposition** (all fingers of the right hand vs. thumb). These tasks have produced reliable activation of SMA, M1, basal ganglia, and cerebellum in healthy subjects and are easy enough to be performed by schizophrenia patients with PS[62, 64, 65, 116, 117]. During one run, we will alternate active and passive blocks, i.e. finger tapping – listening – sequential finger opposition – rest. Each block will last for 15 s and each run takes 1 min. We will alternate the active blocks between runs. A total of 10 runs will be conducted. Instructions will be presented on a video goggle system. Cue tones will be delivered via headphones. Participants will be instructed and trained on the task outside the scanner.

pCASL - The 30 EPI interleaved axial oblique slices will be positioned in-line with the bi-commissural axis and acquired in sequential order with the following parameters: TR = 3000 ms, TE = 12 ms, FA = 90, slice thickness = 8.0 mm, gap thickness = 0 mm, matrix size = 64 x 64 mm, FOV 256 x 256 mm2 resulting in a voxel dimension of 4.0 mm x 4.0 mm x 8.0 mm. the sequence will additionally have the following parameters: bolus duration = 700 ms, inversion time = 2200 ms. In total 100 images will be acquired lasting in total 6 min.

9.2.3 Assessment of other outcomes of interest

Measures of social and community functioning as described in section 5.3.

9.2.4 Assessment of safety outcomes

9.2.4.1 Adverse events

After every rTMS session, participants are asked about new occurrence of adverse events. As example, they are inquired about sensations associated with the stimulation or headaches. The answers are recorded in the CRF. Furthermore, at each study visit during the intervention phase, i.e. after 5 rTMS sessions, patients will be inquired about adverse events using a standard questionnaire in the CRF[2].

In case the adverse event is not limited to stimulation, the participants will be asked at the next session (24 hours later), whether the adverse event still continues or when it was resolved. The frequency and type of adverse events will be reported in the publication of results.

9.2.4.2 *Laboratory parameters*

No laboratory parameters will be taken.

9.2.4.3 *Vital signs*

No routine measurement of vital signs is planned. If patients report dizziness, clinical routines will include measurement of blood pressure and heart rate.

9.2.5 Assessments in participants who prematurely stop the study

Participants who prematurely stop the study will be immediately assessed for adverse events. In case of adverse events reports, a physical examination will be conducted and results will be recorded accordingly. Follow-up examinations will be planned within the next 14 days in case of continuing problem related to adverse events, and these examinations will be continued every 14 days until the health issue is resolved.

9.3 Procedures at each visit

9.3.1 Screening visit

- Check the inclusion and exclusion criteria, use CRF
- Collect demographic data, use CRF
- Conduct urine pregnancy test in female participants between 18 and 50 years of age
- Assess diagnoses with SCID, document results in CRF
- Assess handedness with EHI[101], document result in CRF
- Assess medical history in CRF

See table study events at 9.1

9.3.2 Visit 2 (baseline, week 0)

- Assess current psychopathology, use PANSS interview and BNSS, document in CRF
- Assess formal thought disorder, use TALD interview, document in CRF
- Assess Neurological soft signs, use NES, document in CRF
- Assess psychiatric history with CASH interview in patients, document result in CRF
- Assess primary outcome with SRRS scale, document in CRF
- Assess motor behaviour with rating scales, use BFCRS, UPDRS, AIMS, document results in CRF
- Assess functioning, use GAF, SOFAS, and UPSA-brief, document results in CRF
- Hand out self-rating questionnaires SNS and IPAQ, collect and document in CRF
- Conduct coin rotation test, record on video
- Record gross motor activity using actigraphy
- Test cortical excitability using the TMS device according to standard procedure. Record electronic data
- Acquire MRI scans at University Institute of Diagnostic and Interventional Neuroradiology
- Acquire posturography at University Hospital, Dept. of Neurology, record electronic data
- Record current medication regime in CRF
- Schedule 5 rTMS sessions
- Schedule next visit

9.3.3 Visit 3 and 4 (week 1 and 2)

- Assess primary outcome with SRRS scale, document in CRF
- Assess side effects with questions in the CRF
- Record current medication regime in CRF
- Schedule 5 rTMS sessions
- Schedule next visit

9.3.4 Visit 5 (end of intervention, week 3)

- Assess side effects with questions in the CRF

- Assess current psychopathology, use PANSS interview and BNSS, document in CRF
- Assess primary outcome with SRRS scale, document in CRF
- Assess motor behaviour with rating scales, use BFCRS, UPDRS, AIMS, document results in CRF
- Assess functioning, use GAF, SOFAS, and UPSA-brief, document results in CRF
- Hand out self-rating questionnaires SNS and IPAQ, collect and document in CRF
- Conduct coin rotation test, record on video
- Record gross motor activity using actigraphy
- Test cortical excitability using the TMS device according to standard procedure. Record electronic data
- Acquire MRI scans at University Institute of Diagnostic and Interventional Neuroradiology
- Acquire posturography at University Hospital, Dept. of Neurology, record electronic data
- Record current medication regime in CRF
- Schedule next visit (follow-up)

9.3.5 Visit 6 and 7 (follow-up, week 6 and 24)

- Assess current psychopathology, use PANSS interview and BNSS, document in CRF
- Assess primary outcome with SRRS scale, document in CRF
- Assess motor behaviour with rating scales, use BFCRS, UPDRS, AIMS, document results in CRF
- Assess functioning, use GAF, SOFAS, and UPSA-brief, document results in CRF
- Hand out self-rating questionnaires SNS and IPAQ, collect and document in CRF
- Conduct coin rotation test, record on video
- Record gross motor activity using actigraphy
- Test cortical excitability using the TMS device according to standard procedure. Record electronic data
- Record current medication regime in CRF
- Schedule next visit (follow-up)

10. SAFETY

10.1 Medical Device Category A studies

Device deficiencies and all **adverse events (AE)** including all **serious adverse events (SAE)** are collected, fully investigated and documented in the source document and appropriate case report form (CRF) during the entire study period, i.e. from patient's informed consent until the last protocol-specific procedure, including a safety follow-up period. Documentation includes dates of event, treatment, resolution, assessment of seriousness and causal relationship to device and/or study procedure [ISO 14155, 6.4.1.].

Safety is assessed spontaneously at each stimulation session and in a structured set of questions at each visit during the intervention phase (after 5 stimulations). See 9.2.4. If adverse events occur, patients are followed-up with extra-visits after 14 days until the issue has resolved.

Typical adverse events to be expected by rTMS are:

- Mild pain or nausea (39 %)
- Mild headaches (28%)
- Mild neck pain (40%)

Rare events include hearing problems or local skin irritation.

10.1.1 Definition and Assessment of safety related events

Adverse Event (AE)

Any untoward medical occurrence, unintended disease or injury or any untoward clinical signs (including an abnormal laboratory finding) in participants, users or other persons whether or not

related to the investigational medical device [ISO 14155: 3.2].

This includes events related to the investigational device or the comparator and to the procedures involved. For users or other persons this is restricted to events related to the investigational medical device.

Adverse Device Effect (ADE)

Adverse event related to the use of an investigational medical device [ISO 14155: 3.1].

This includes any adverse event resulting from insufficient or inadequate instructions for use, deployment, implantation, installation, operation, or any malfunction of the investigational medical device. This includes any event that is a result of a use error or intentional misuse.

Serious Adverse Event (SAE) [European regulation on medical devices 2017/745, art. 58].

Any adverse event that led to any of the following:

(a) death,
(b) serious deterioration in the health of the subject that resulted in any of the following:
(i) life-threatening illness or injury,
(ii) permanent impairment of a body structure or a body function,
(iii) hospitalisation or prolongation of patient hospitalisation,
(iv) medical or surgical intervention to prevent life-threatening illness or injury or permanent impairment to a body structure or a body function,
(v) chronic disease,
(c) foetal distress, foetal death or a congenital physical or mental impairment or birth defect.

This includes device deficiencies that might have led to a serious adverse event if a) suitable action had not been taken or b) intervention had not been made or c) if circumstances had been less fortunate. These are submitted to the EC via BASEC within 7 days. A planned hospitalisation for pre-existing condition, or a procedure required by the protocol, without a serious deterioration in health, is not considered to be a serious adverse event.

Device deficiency

Inadequacy of a medical device related to its identity, quality, durability, reliability, safety or performance, such as malfunction, misuse or use error and inadequate labelling [ISO 14155: 3.15].

Health hazards that require measures

Findings in the trial that may affect the safety of study participants and, which require preventive or corrective measures intended to protect the health and safety of study participants SAE [ClinO Art. 37].

Causal Relationship of SAE [MEDDEV 2.7/3 revision 3, May 2015].

A causal relationship towards the medical device or study procedure should be rated as follows:

- **Not related:** The relationship to the device or procedures can be excluded.
- **Unlikely:** The relationship with the use of the device seems not relevant and/or the event can be reasonably explained by another cause, but additional information may be obtained.
- **Possible:** The relationship with the use of the investigational device is weak but cannot be ruled out completely. Alternative causes are also possible.
- **Probable:** The relationship with the use of the investigational device seems relevant and/or the event cannot reasonably explained by another cause.
- **Causal relationship:** The serious event is associated with the investigational device or with procedures beyond reasonable doubt.

Device deficiencies that might have led to an SAE are always related to the medical device.

10.1.2 Reporting of (Serious) Adverse Events and other safety related events

Reporting to Sponsor-Investigator:

The following events are to be reported to the Sponsor-Investigator within 24 hours upon becoming aware of the event:

- All SAEs
- Health hazards that require measures
- Device deficiencies

The Sponsor-Investigator will evaluate SAEs with regard to causality and seriousness. Device deficiencies are assessed regarding their potential to lead to an SAE.

Pregnancies

Because pregnancy tests are a prerequisite to participation and the intervention is only 3 weeks, pregnancies will not be further assessed or reported.

Reporting to Authorities [ClinO Art. 42]:

In Category A studies, the sponsor is subject to the notification requirements specified in Art. 15 of the MedDO of 17 October 2011 (SR 812.213).

It is the Investigator's responsibility to report to the Ethics Committee via BASEC **device deficiencies** that could have led to serious adverse events if suitable action had not been taken, intervention had not been made, or circumstances had been less fortunate within 7 days [ClinO Art. 42].

Health hazards that require measures are reported to the Ethics Committee via BASEC within 2 days [ClinO Art. 37].

Periodic safety reporting:

A yearly safety update-report is submitted by the Investigator to the Ethics Committee via BASEC.

10.1.3 Follow up of (Serious) Adverse Events

If adverse events occur, patients are followed-up with extra-visits after 14 days until the issue has resolved. This applies to patients who continue with the study but also to patients who prematurely exit the study. In case subjects are lost to followed-up, study personnel will contact the treating psychiatrists (or if unavailable, the general practitioners) in order to inform on serious adverse events and the recommendations for follow-up clinical examinations. In cases of headaches for example, care would include the prescription of a non-steroidal antirheumatic drug, i.e. pain medicine, according to the person's preferences, medical history, or interaction with existing medication.

11. STATISTICAL METHODS

A two-tailed p-value of $< .05$ is considered to be statistically significant in all analyses. We will also report effect sizes for the comparisons of primary and clinical secondary outcome variables between groups.

11.1 Hypotheses

Aim 1: investigate the clinical and functional neural changes following 15 sessions of daily rTMS

Hypothesis 1a (main hypothesis): lf-rTMS (inhibitory) will be superior over placebo or iTBS (facilitatory) in all clinical and motor behavioral measures, e.g. greater reduction in SRRS scores from baseline, greater increase in activity levels, or coin rotations.

Hypothesis 1b: lf-rTMS will reduce aberrant functional connectivity in the motor system, e.g. between thalamus and M1. lf-rTMS will alter regional CBF in the motor system and increase SMA and M1 activity during the fMRI task. No relevant changes are expected in the sham group, iTBS may deteriorate neural alterations.

Hypothesis 1c: cortical excitability will be differentially changed with rTMS. Lf-rTMS will increase SICI, while iTBS will reduce SICI.

Aim 2: characterization of psychomotor slowing (PS) in schizophrenia spectrum disorders compared to healthy controls

Hypothesis 2a: Patients will have poorer performance on all motor tasks, eg. reduced activity levels, increased postural sway, and less coin rotations

Hypothesis 2b: Patients will have aberrant structural and functional connectivity within the motor system, as well as increased CBF in basal ganglia and decreased CBF in premotor/motor cortex. Applying network metrics, the functional motor network will be less efficient in schizophrenia.

Hypothesis 2c: Behavioral measures of PS will be associated with aberrant motor network structure, perfusion, function, and connectivity. For example, patients with strong PS will have increased resting state functional connectivity between thalamus and M1. PS severity will be linked to reduced structural motor network efficiency, lower M1 and SMA activity during the fMRI task, and increased SMA perfusion at rest.

Hypothesis 2d: Patients will have increased motor cortex excitability (e.g. reduced SICI), which will be linked to measures of PS.

Aim 3 characterize short term dynamics of PS

Hypothesis 3a: in very few subjects we expect relevant spontaneous improvements in PS measures. The majority will have less than 20% fluctuation of motor parameters within 3 weeks

Hypothesis 3b: we expect slight longitudinal changes in resting state functional connectivity and perfusion of the motor system, but no structural changes.

Aim 4: to describe the short-term and medium-term clinical outcome of 3 weeks of rTMS intervention

Hypothesis 4a: patients receiving inhibitory rTMS will have better outcome (less PS, less general symptom severity) shortly after the intervention (at 6 week follow-up) compared to patients receiving placebo or facilitatory iTBS

Hypothesis 4b: patients receiving inhibitory rTMS will have better outcome (less PS, less general symptom severity) and superior function (social and global) at 6-month follow-up compared to patients receiving placebo or facilitatory iTBS

Hypothesis 4c (exploratory): changes in functional connectivity within the motor system from baseline to week 3 will predict better outcome at week 6, particularly reduced M1-thalamus functional connectivity.

11.2 Determination of Sample Size

The main effects of the 3-week interventions on PS (SRRS scores) will be calculated in a repeated measures design including 2 measures (Baseline, week 3) and four groups (lf-rTMS, iTBS, sham, waiting group-lf-rTMS). Assuming a moderate effect size (f = 0.23) as indicated by our pilot data in a repeated measures ANOVA with moderate correlation between time points (0.5), a power of 0.95 and an alpha = 0.05, we would need 88 patients (22 per group).

11.3 Statistical criteria of termination of trial

No statistical stopping rules are planned.

11.4 Planned Analyses

11.4.1 Datasets to be analysed, analysis populations

All data from subjects in the intent-to-treat population, who received at least one dose of rTMS will be used in the analyses of rTMS effects. We will conduct sensitivity analyses in all trial-completers (see 11.5).

In the analyses on biological and clinical correlates of PS, we will use all data of the baseline assessment, thus including data from subjects who dropped-out prior to the first rTMS session.

11.4.2 Primary Analysis

The primary analysis will be a repeated measures ANOVA of SRRS scores with two timepoints (within

subjects: baseline and week 3) and four groups (lf-rTMS, iTBS, sham, waiting group-lf-rTMS). In case of significant baseline demographic or clinical group differences, we will adjust for these in an repeated measures ANCOVA of SRRS.

The analysis will be performed by the PI and his team in SPSS using data from the RedCap database.

11.4.3 Secondary Analyses

Secondary analyses focus on the secondary outcomes (see 5.2) and hypotheses (see 11.1).

Crosstabs are used to calculate the proportion of responders (>=30% reduction in SRRS from baseline) across groups. Repeated measures ANOVAs will clarify the time-course of SRRS change from baseline to week 6 (including week 1, 2, 3 and 6) and differences between groups.

Repeated measures ANOVAs will test the change of clinical and motor rating scales between baseline and week 3, week 6, and week 24 and differences between groups.

Linear regression analyses will test the association between neuroimaging markers (resting state perfusion, connectivity, fMRI activation, ect.) and measures of PS at baseline.

Repeated measures ANOVAs will test the longitudinal changes in neuroimaging markers from baseline to week 3.

All analyses will be performed by the PI and his team or collaborators with permission of the PI.

11.4.4 Interim analyses

Interim analyses of the primary analysis are planned after enrolment of 10, 20, 30, 40, 50, 60, and 70 subjects. The scope is to estimate whether any adjustments of the treatment arms are necessary. In addition, baseline data can be tested for associations between neuroimaging data and clinical/motor measures after enrolment of 20, 40, or 60 subjects.

All interim analyses are optional and at the discretion of the PI. Interim analyses are conducted by the PI and his team.

11.4.5 Safety analysis

The study team will provide a descriptive analysis of all reported adverse events. The analysis will focus on the type of event, duration, and timing and relate the number of observed events to the number of rTMS sessions administered. Furthermore, the frequency of adverse events will be compared between treatment arms of the trial.

11.4.6 Deviation(s) from the original statistical plan

Any deviation from the statistical plan will be reported and justified in the methods of the study report.

11.5 Handling of missing data and drop-outs

We will apply the last-observation-carried-forward (LOCF) method in the final analyses. In case a patient withdraws consent before the first rTMS stimulation, we will recruit an additional patient. The number of all patients recruited and randomized will be reported in the publications.

Sensitivity analyses will be performed in order to establish whether premature dropout would influence the results. Therefore, the primary analyses will be repeated with all subjects who completed the interventional trial. Further tests will establish whether drop-outs differ from completers in basic clinical or demographic variables.

12. QUALITY ASSURANCE AND CONTROL

All study personnel will be trained by the PI to achieve consistent adherence to procedures and interrater reliability. Standard operating procedures will be written for the intervention and standardized TMS measurements. For all clinical rating scales, instructions and manuals are provided for the study personnel. All study personnel will have to read the manuals.

12.1 Data handling and record keeping / archiving

12.1.1 Case Report Forms

All relevant data is documented in paper CRFs. This data includes inclusion/exclusion criteria, demographic data, medical history, scores of clinical and motor rating scales. Furthermore, side effects, concomitant treatment, reasons for discontinuation will be recorded in the CRF. Timing and conductance of experimental measures, such as posturography, cerebral MRI, cortical excitability, and actigraphy will be recorded recorded in the CRF. However, the real data of these measures is stored electronically outside the CRF.

For each enrolled study participant a CRF is maintained. CRFs will be kept current to reflect subject status at each phase during the course of study. Participants will be coded by use of a participant number in combination with the year of birth.

The paper forms of rating scales (e.g. PANSS) and the electronic data (e.g. MRI, posturography, ect.) are considered source documents. Furthermore, in patients the medical record of our hospital is a source document for the past psychiatric history and current concomitant treatment. All other data (participant history, side effects, ect.) are directly entered into the CRF without further source.

The study personnel is authorized to enter data into the CRFs, the name of the entering person will be documented. CRF data will be recorded in an electronic database, i.e. RedCap-Database. The procedure will include data cross-check between electronic and paper CRF. Any deviation will be corrected by consensus and consulting the CRF forms. RedCap-Database has individualized logins for each member of the study team and provides logs for data entry.

The electronic database for the non-CRF data will be logged via a secure Dropbox folder, thus the person entering the data or changing data can be identified by individualized logins. The log also identifies the document changes and the time of the change. Dropbox allows for restoring each version of the documents within the study folder. A copy of the electronic neuroimaging data will be shared with Collaborator Prof. Jessica Bernard, College Station, TX, USA. At her laboratory, specific neuroimaging analyses will be performed with a subset of the electronic data. The original electronic files will be stored in Bern (see 12.1.3), while the specific neuroimaging analyses data of Prof. Bernard will be stored encrypted at her university for 10 years.

12.1.2 Specification of source documents

As described in 12.1.1. most data in the CRFs is source data. Furthermore, Informed consent form, specific rating scales and electronic data (such as MRI, actigraphy or posturography) are source data. In patients, the medical record of the hospital may contain source data on the psychiatric history. All CRFs and paper source data will be kept in folders. Any extra examination in case of additional safety assessments will be documented on paper and kept as source data in the same folders as CRF and other source data.

12.1.3 Record keeping / archiving

All study data will be archived for 10 years after study termination or premature termination of the trial at the translational research center of the University Hospital of Psychiatry, Bern. In addition, the data of specific neuroimaging analyses performed at Prof. Bernard's laboratory, will be stored encrypted for 10 years at Texas A & M University, College Station, TX, USA. Thus, source data are kept in Bern, and all further handled data will be stored under identical conditions in Bern or Texas Station.

12.2 Data management

Data of all participants will be encrypted and analysis will be performed using encrypted data only. Unblinding of participant-specific data is only possible after consent has been given by the participants and the sponsor. Data of all participants will receive a numerical identification. The key to this encryption code will be stored in a single file at the address below, and it will be accessible only by the sponsor and the principal investigator.

Office of the PI: University of Bern, University Hospital of Psychiatry, Murtenstrasse 21, 3008 Bern, Office 01-132

12.2.1 Data Management System

Describe what system (also software) is being used and who is responsible and how it is tested before the trial (may include a description of where the system is hosted).

12.2.1.1 Neuroimaging data

MRI data are anonymized digital data of appr. 1 GB per subject and scan, we will aim for 278 scans, i.e. total of 300 GB. MRI data sets include the dicom files of the planned sequences fMRI task, fMRI rest, ASL rest, DTI and T1 anatomy. MRI data sets will be safely stored in encrypted formats at the server of the translational research center, university hospital in Bern. Data will be logged with personal logins, such that any change to the files is reproducible. Data analysis will be performed locally in computers of the institute's network. Some analyses are planned to be performed on encrypted data at computers of the study collaborator Prof. Bernard. All files generated during data processing and analysis will be stored. Total data volume is approximately 2 TB. Neuroimaging data will be stored at a dropbox-folder, which is encrypted by Boxcryptor Software and owned by the Translational Research Center, University Hospital of Psychiatry, Bern. The system has been effectively used in several studies. Access to the folder and files is secured by personal login and password. The log of the Dropbox folder enables the identification of data entry/data change according to the logins used. The log will inform on which data has been changed when by whom. Dropbox allows for restoration of each file version, thus each change can be tracked.

12.2.1.2 motor behavioral/instrumental data

Data from actigraphy, posturography, TMS-MEP experiments as well as video recordings of coin rotation will be stored encrypted in digital format. As neuroimaging data, storage will comply with the federal human research act and will be conducted at standard facilities of the translational research center with encrypted and logged server storage. Data access is granted upon personal logins, such that any change to the files is reproducible. Each dataset includes several measures across 4-5 time points for one study participant. We expect appr. 1 GB per subject, total of 130 GB.

12.2.1.3 clinical and demographic data

Clinical data including outcome measures with observer based rating scales or patients' history will be collected. This data is transferred to paper case report forms and will later be anonymously transferred to a red cap data base, hosted by the CTU Bern. Data access is only available to study staff with personalized logins. During the longitudinal assessments approximately 300 variables will be assessed for each participant.

12.2.2 Data security, access and back-up

Only the PI and authorized personnel with own logins and passwords will have access to the electronic data. The translational research center Bern runs a daily backup of the dropbox folders. The RedCap Database also has regular backups.

12.2.3 Analysis and archiving

All analyses will be performed with a final copy of the SPSS file including all clinical data. All other analyses (neuroimaging, posturography, actigraphy) will be conducted with the appropriate standard software. Only the PI and authorized personnel will have access to the data to perform the analyses.

12.2.4 Electronic and central data validation

Data will be checked for consistency, e.g. range checks for questionnaire scores or test scores, checks for date entries temporal consistency.

12.3 Monitoring

The principal investigator will check the CRFs for completeness and internal consistency (plausibility of information) and external consistency (with source data) after each participant completed the study. In addition an external monitoring will be performed by Dr. Peter.

Four monitoring dates are planned. The first before the study recruitment starts, the second after the first three participants have been included, the third after 50% of enrolment was achieved and the final monitoring is planned after the last data had been collected.

Before first recruitment: Check whether all study assessments and instruments are ready and complete.

After the first three participants: Check CRF entries and compliance with source data where possible. Clarify queries of data entry or study procedures.

After the 50% enrolment: Check whether CRFs of the all collected cases are completed and data is consistent.

After the final data collection: Check whether CRFs of the all cases collected after the last monitoring are completed and data is consistent.

12.4 Audits and Inspections

Not intended for this single center trial.

12.5 Confidentiality, Data Protection

All data will be handled strictly confidential. Direct access to the source documents will be permitted only for purposes of inspections (ICHE6, 6.10) by authorities such as the CEC or monitoring by the PI. Only the PI and his team will have access to the protocol, dataset and other study related information during and after the study.

12.6 Storage of biological material and related health data

No storage of biological material is planned.

All electronic data will be archived for 10 years (see 12.1).

13. PUBLICATION AND DISSEMINATION POLICY

Publication of results is the responsibility of the sponsor/investigator. The results of the study will be prepared for publication in peer-reviewed scientific journals, preferably as open-access articles. Authorship will follow the national guidelines. Furthermore, results will be reported at scientific conferences as posters or oral communications. The use of professional writers is not intended. After scientific publication, the results will be prepared for communication in lay man's terms in the media.

Data sharing outside of the project is currently not planned. However, electronic data, particularly neuroimaging electronic data, may be later shared with national or international collaborators to address research questions that are currently unknown. The data storage must comply with the same regulations as the data storage of this study.

14. FUNDING AND SUPPORT

14.1 Funding

This study receives project funding by the Swiss National Science Foundation (grant #182469 to Prof. Sebastian Walther). Additional minor costs will be covered by the University Hospital of Psychiatry in Bern.

14.2 Other Support

Consumables and further material support is provided by the host institution, the Translational Research Center of the University Hospital of Psychiatry, University of Bern, Switzerland.

15. INSURANCE

No special insurance due to category A.

16. REFERENCES

1. Rossi S, Hallett M, Rossini PM, Pascual-Leone A, Safety of TMSCG. Safety, ethical considerations, and application guidelines for the use of transcranial magnetic stimulation in clinical practice and research. Clin Neurophysiol 2009;120:2008-2039.
2. Lefaucheur JP, Andre-Obadia N, Antal A, et al. Evidence-based guidelines on the therapeutic use of repetitive transcranial magnetic stimulation (rTMS). Clin Neurophysiol 2014;125:2150-2206.
3. Owen MJ, Sawa A, Mortensen PB. Schizophrenia. Lancet 2016;388:86-97.
4. Howes OD, Murray RM. Schizophrenia: an integrated sociodevelopmental-cognitive model. Lancet 2014;383:1677-1687.
5. Tandon R, Nasrallah HA, Keshavan MS. Schizophrenia, "just the facts" 4. Clinical features and conceptualization. Schizophr Res 2009;110:1-23.
6. Yang GJ, Murray JD, Wang XJ, et al. Functional hierarchy underlies preferential connectivity disturbances in schizophrenia. Proc Natl Acad Sci U S A 2016;113:E219-228.
7. van den Heuvel MP, Sporns O, Collin G, et al. Abnormal rich club organization and functional brain dynamics in schizophrenia. JAMA Psychiatry 2013;70:783-792.
8. Klauser P, Baker ST, Cropley VL, et al. White Matter Disruptions in Schizophrenia Are Spatially Widespread and Topologically Converge on Brain Network Hubs. Schizophr Bull 2016.
9. Friston KJ. Schizophrenia and the disconnection hypothesis. Acta Psychiatr Scand Suppl 1999;395:68-79.
10. Walther S, Mittal VA. Motor System Pathology in Psychosis. Curr Psychiatry Rep 2017;19:97.
11. Walther S, Stegmayer K, Federspiel A, Bohlhalter S, Wiest R, Viher PV. Aberrant Hyperconnectivity in the Motor System at Rest Is Linked to Motor Abnormalities in Schizophrenia Spectrum Disorders. Schizophr Bull 2017;43:982-992.
12. Mittal V, Bernard JA, Northoff G. What can Different Motor Circuits Tell Us About Psychosis? An RDoC Perspective. Schizophr Bull 2017.
13. Kindler J, Schultze-Lutter F, Michel C, et al. Abnormal involuntary movements are linked to psychosis-risk in children and adolescents: Results of a population-based study. Schizophr Res 2016;174:58-64.
14. Mittal VA, Neumann C, Saczawa M, Walker EF. Longitudinal progression of movement abnormalities in relation to psychotic symptoms in adolescents at high risk of schizophrenia. Arch Gen Psychiatry 2008;65:165-171.
15. van Harten PN, Walther S, Kent JS, Sponheim SR, Mittal VA. The clinical and prognostic value of motor abnormalities in psychosis, and the importance of instrumental assessment. Neurosci Biobehav Rev 2017;80:476-487.
16. Peralta V, Campos MS, De Jalon EG, Cuesta MJ. Motor behavior abnormalities in drug-naive patients with schizophrenia spectrum disorders. Mov Disord 2010;25:1068-1076.
17. Putzhammer A, Perfahl M, Pfeiff L, Hajak G. Correlation of subjective well-being in schizophrenic patients with gait parameters, expert-rated motor disturbances, and psychopathological status. Pharmacopsychiatry 2005;38:132-138.
18. Kao YC, Liu YP, Chou MK, Cheng TH. Subjective quality of life in patients with chronic schizophrenia: relationships between psychosocial and clinical characteristics. Compr Psychiatry 2011;52:171-180.
19. Mittal VA. Cross-Cutting Advancements Usher in a New Era for Motor Research in Psychosis. Schizophr Bull 2016;42:1322-1325.
20. Walther S, Stegmayer K, Sulzbacher J, et al. Nonverbal social communication and gesture control in schizophrenia. Schizophr Bull 2015;41:338-345.
21. Walther S, Eisenhardt S, Bohlhalter S, et al. Gesture Performance in Schizophrenia Predicts Functional Outcome After 6 Months. Schizophrenia Bull 2016;42:1326-1333.
22. Walther S. Psychomotor symptoms of schizophrenia map on the cerebral motor circuit. Psychiatry Res 2015;233:293-298.
23. Morrens M, Hulstijn W, Sabbe B. Psychomotor slowing in schizophrenia. Schizophr Bull 2007;33:1038-1053.

24. Walther S, Strik W. Motor Symptoms and Schizophrenia. Neuropsychobiology 2012;66:77-92.
25. Morrens M, Docx L, Walther S. Beyond boundaries: in search of an integrative view on motor symptoms in schizophrenia. Front Psychiatr 2014;5:145.
26. Docx L, Sabbe B, Provinciael P, Merckx N, Morrens M. Quantitative psychomotor dysfunction in schizophrenia: a loss of drive, impaired movement execution or both? Neuropsychobiology 2013;68:221-227.
27. Walther S, Federspiel A, Horn H, et al. Alterations of white matter integrity related to motor activity in schizophrenia. Neurobiol Dis 2011;42:276-283.
28. Wichniak A, Skowerska A, Chojnacka-Wojtowicz J, et al. Actigraphic monitoring of activity and rest in schizophrenic patients treated with olanzapine or risperidone. J Psychiatr Res 2011;45:1381-1386.
29. Putzhammer A, Heindl B, Broll K, Pfeiff L, Perfahl M, Hajak G. Spatial and temporal parameters of gait disturbances in schizophrenic patients. Schizophr Res 2004;69:159-166.
30. Lisi G, Nico D, Ribolsi M, et al. Asymmetries in initiation of aiming movements in schizophrenia. Neuropsychologia 2018;109:200-207.
31. Docx L, Morrens M, Bervoets C, et al. Parsing the components of the psychomotor syndrome in schizophrenia. Acta Psychiatr Scand 2012;126:256-265.
32. Morrens M, Hulstijn W, Van Hecke J, Peuskens J, Sabbe BG. Sensorimotor and cognitive slowing in schizophrenia as measured by the Symbol Digit Substitution Test. J Psychiatr Res 2006;40:200-206.
33. Fervaha G, Agid O, Takeuchi H, et al. Extrapyramidal symptoms and cognitive test performance in patients with schizophrenia. Schizophr Res 2015;161:351-356.
34. Peralta V, Cuesta MJ. Neuromotor abnormalities in neuroleptic-naive psychotic patients: antecedents, clinical correlates, and prediction of treatment response. Compr Psychiatry 2011;52:139-145.
35. Walther S, Stegmayer K, Horn H, Razavi N, Muller TJ, Strik W. Physical Activity in Schizophrenia is Higher in the First Episode than in Subsequent Ones. Front Psychiatry 2014;5:191.
36. Cuesta MJ, Sanchez-Torres AM, de Jalon EG, et al. Spontaneous parkinsonism is associated with cognitive impairment in antipsychotic-naive patients with first-episode psychosis: a 6-month follow-up study. Schizophr Bull 2014;40:1164-1173.
37. Grootens KP, Vermeeren L, Verkes RJ, et al. Psychomotor planning is deficient in recent-onset schizophrenia. Schizophr Res 2009;107:294-302.
38. Mittal VA, Gupta T, Orr JM, et al. Physical activity level and medial temporal health in youth at ultra high-risk for psychosis. J Abnorm Psychol 2013;122:1101-1110.
39. Dean DJ, Mittal VA. Spontaneous parkinsonisms and striatal impairment in neuroleptic free youth at ultrahigh risk for psychosis. NPJ Schizophr 2015;1.
40. Niendam TA, Bearden CE, Johnson JK, et al. Neurocognitive performance and functional disability in the psychosis prodrome. Schizophr Res 2006;84:100-111.
41. Stubbs B, Ku PW, Chung MS, Chen LJ. Relationship Between Objectively Measured Sedentary Behavior and Cognitive Performance in Patients With Schizophrenia Vs Controls. Schizophr Bull 2017;43:566-574.
42. Stubbs B, Chen LJ, Chung MS, Ku PW. Physical activity ameliorates the association between sedentary behavior and cardiometabolic risk among inpatients with schizophrenia: A comparison versus controls using accelerometry. Compr Psychiatry 2017;74:144-150.
43. Martin-Sierra A, Vancampfort D, Probst M, et al. Walking capacity is associated with health related quality of life and physical activity level in patients with schizophrenia: a preliminary report. Actas Esp Psiquiatr 2011;39:211-216.
44. Gur RC, Ragland JD, Moberg PJ, et al. Computerized neurocognitive scanning: II. The profile of schizophrenia. Neuropsychopharmacology 2001;25:777-788.
45. Lee RS, Hermens DF, Naismith SL, et al. Neuropsychological and functional outcomes in recent-onset major depression, bipolar disorder and schizophrenia-spectrum disorders: a longitudinal cohort study. Transl Psychiatry 2015;5:e555.
46. Boden R, Abrahamsson T, Holm G, Borg J. Psychomotor and cognitive deficits as predictors of

5-year outcome in first-episode schizophrenia. Nord J Psychiatry 2014;68:282-288.

47. Tohen M, Khalsa HM, Salvatore P, et al. The McLean-Harvard First-Episode Project: Early Course in 114 Cases of First-Episode Nonaffective Psychoses. J Clin Psychiatry 2016;77:781-788.
48. Walther S, Koschorke P, Horn H, Strik W. Objectively measured motor activity in schizophrenia challenges the validity of expert ratings. Psychiatry Res 2009;169:187-190.
49. Walther S, Ramseyer F, Horn H, Strik W, Tschacher W. Less structured movement patterns predict severity of positive syndrome, excitement, and disorganization. Schizophr Bull 2014;40:585-591.
50. Walther S, Stegmayer K, Horn H, et al. The Longitudinal Course of Gross Motor Activity in Schizophrenia - Within and between Episodes. Front Psychiatry 2015;6:10.
51. Gebhardt A, Vanbellingen T, Baronti F, Kersten B, Bohlhalter S. Poor dopaminergic response of impaired dexterity in Parkinson's disease: Bradykinesia or limb kinetic apraxia? Mov Disord 2008;23:1701-1706.
52. Walther S, Vanbellingen T, Muri R, Strik W, Bohlhalter S. Impaired pantomime in schizophrenia: Association with frontal lobe function. Cortex 2013;49:520-527.
53. Kent JS, Hong SL, Bolbecker AR, et al. Motor deficits in schizophrenia quantified by nonlinear analysis of postural sway. PLoS One 2012;7:e41808.
54. Matsuura Y, Fujino H, Hashimoto R, et al. Standing postural instability in patients with schizophrenia: Relationships with psychiatric symptoms, anxiety, and the use of neuroleptic medications. Gait Posture 2015;41:847-851.
55. Peralta V, Cuesta MJ. The effect of antipsychotic medication on neuromotor abnormalities in neuroleptic-naive nonaffective psychotic patients: a naturalistic study with haloperidol, risperidone, or olanzapine. Prim Care Companion J Clin Psychiatry 2010;12.
56. Malla A, Norman R, Scholten D, et al. A comparison of two novel antipsychotics in first episode non-affective psychosis: one-year outcome on symptoms, motor side effects and cognition. Psychiatry Res 2004;129:159-169.
57. Kopala LC, Good KP, Milliken H, et al. Treatment of a first episode of psychotic illness with quetiapine: an analysis of 2 year outcomes. Schizophr Res 2006;81:29-39.
58. Walther S, Federspiel A, Horn H, et al. Resting state cerebral blood flow and objective motor activity reveal basal ganglia dysfunction in schizophrenia. Psychiatry Res 2011;192:117-124.
59. Docx L, Emsell L, Van Hecke W, et al. White matter microstructure and volitional motor activity in schizophrenia: A diffusion kurtosis imaging study. Psychiatry Res 2016;260:29-36.
60. Bracht T, Schnell S, Federspiel A, et al. Altered cortico-basal ganglia motor pathways reflect reduced volitional motor activity in schizophrenia. Schizophr Res 2013;143:269-276.
61. Karbasforoushan H, Duffy B, Blackford JU, Woodward ND. Processing speed impairment in schizophrenia is mediated by white matter integrity. Psychol Med 2015;45:109-120.
62. Singh S, Goyal S, Modi S, et al. Motor function deficits in schizophrenia: an fMRI and VBM study. Neuroradiology 2014;56:413-422.
63. Walther S, Schappi L, Federspiel A, et al. Resting-State Hyperperfusion of the Supplementary Motor Area in Catatonia. Schizophr Bull 2017;43:972-981.
64. Scheuerecker J, Ufer S, Kapernick M, et al. Cerebral network deficits in post-acute catatonic schizophrenic patients measured by fMRI. J Psychiatr Res 2009;43:607-614.
65. Payoux P, Boulanouar K, Sarramon C, et al. Cortical motor activation in akinetic schizophrenic patients: a pilot functional MRI study. Mov Disord 2004;19:83-90.
66. Bunse T, Wobrock T, Strube W, et al. Motor cortical excitability assessed by transcranial magnetic stimulation in psychiatric disorders: a systematic review. Brain Stimul 2014;7:158-169.
67. Hasan A, Wobrock T, Grefkes C, et al. Deficient inhibitory cortical networks in antipsychotic-naive subjects at risk of developing first-episode psychosis and first-episode schizophrenia patients: a cross-sectional study. Biol Psychiatry 2012;72:744-751.
68. Du X, Kochunov P, Summerfelt A, Chiappelli J, Choa FS, Hong LE. The role of white matter microstructure in inhibitory deficits in patients with schizophrenia. Brain Stimul 2017;10:283-290.
69. Hallett M. Transcranial magnetic stimulation: a primer. Neuron 2007;55:187-199.

70. Orosz A, Jann K, Wirth M, Wiest R, Dierks T, Federspiel A. Theta burst TMS increases cerebral blood flow in the primary motor cortex during motor performance as assessed by arterial spin labeling (ASL). Neuroimage 2012;61:599-605.

71. Siebner HR, Filipovic SR, Rowe JB, et al. Patients with focal arm dystonia have increased sensitivity to slow-frequency repetitive TMS of the dorsal premotor cortex. Brain 2003;126:2710-2725.

72. Ji GJ, Yu F, Liao W, Wang K. Dynamic aftereffects in supplementary motor network following inhibitory transcranial magnetic stimulation protocols. Neuroimage 2017;149:285-294.

73. Oxley T, Fitzgerald PB, Brown TL, de Castella A, Daskalakis ZJ, Kulkarni J. Repetitive transcranial magnetic stimulation reveals abnormal plastic response to premotor cortex stimulation in schizophrenia. Biol Psychiatry 2004;56:628-633.

74. Peralta V, Cuesta MJ. Motor Abnormalities: From Neurodevelopmental to Neurodegenerative Through "Functional" (Neuro)Psychiatric Disorders. Schizophr Bull 2017;43:956-971.

75. Speer AM, Willis MW, Herscovitch P, et al. Intensity-dependent regional cerebral blood flow during 1-Hz repetitive transcranial magnetic stimulation (rTMS) in healthy volunteers studied with H215O positron emission tomography: I. Effects of primary motor cortex rTMS. Biol Psychiatry 2003;54:818-825.

76. Tallabs FA, Hammond-Tooke GD. Theta priming of 1-Hz rTMS in healthy volunteers: effects on motor inhibition. J Clin Neurophysiol 2013;30:79-85.

77. Shirota Y, Ohtsu H, Hamada M, Enomoto H, Ugawa Y, Research Committee on r TMSToPsD. Supplementary motor area stimulation for Parkinson disease: a randomized controlled study. Neurology 2013;80:1400-1405.

78. Benninger DH, Berman BD, Houdayer E, et al. Intermittent theta-burst transcranial magnetic stimulation for treatment of Parkinson disease. Neurology 2011;76:601-609.

79. Kindler J, Homan P, Jann K, et al. Reduced neuronal activity in language-related regions after transcranial magnetic stimulation therapy for auditory verbal hallucinations. Biol Psychiatry 2013;73:518-524.

80. Hoffman RE, Hawkins KA, Gueorguieva R, et al. Transcranial magnetic stimulation of left temporoparietal cortex and medication-resistant auditory hallucinations. Arch Gen Psychiatry 2003;60:49-56.

81. Ray P, Sinha VK, Tikka SK. Adjuvant low-frequency rTMS in treating auditory hallucinations in recent-onset schizophrenia: a randomized controlled study investigating the effect of high-frequency priming stimulation. Ann Gen Psychiatry 2015;14:8.

82. Bais L, Vercammen A, Stewart R, et al. Short and long term effects of left and bilateral repetitive transcranial magnetic stimulation in schizophrenia patients with auditory verbal hallucinations: a randomized controlled trial. PLoS One 2014;9:e108828.

83. Klirova M, Horacek J, Novak T, et al. Individualized rTMS neuronavigated according to regional brain metabolism ((18)FGD PET) has better treatment effects on auditory hallucinations than standard positioning of rTMS: a double-blind, sham-controlled study. Eur Arch Psychiatry Clin Neurosci 2013;263:475-484.

84. Paillere-Martinot ML, Galinowski A, Plaze M, et al. Active and placebo transcranial magnetic stimulation effects on external and internal auditory hallucinations of schizophrenia. Acta Psychiatr Scand 2017;135:228-238.

85. Kindler J, Homan P, Flury R, Strik W, Dierks T, Hubl D. Theta burst transcranial magnetic stimulation for the treatment of auditory verbal hallucinations: results of a randomized controlled study. Psychiatry Res 2013;209:114-117.

86. Vercammen A, Knegtering H, Bruggeman R, et al. Effects of bilateral repetitive transcranial magnetic stimulation on treatment resistant auditory-verbal hallucinations in schizophrenia: a randomized controlled trial. Schizophr Res 2009;114:172-179.

87. Blumberger DM, Vila-Rodriguez F, Thorpe KE, et al. Effectiveness of theta burst versus high-frequency repetitive transcranial magnetic stimulation in patients with depression (THREE-D): a randomised non-inferiority trial. Lancet 2018;391:1683-1692.

88. Duprat R, Desmyter S, Rudi de R, et al. Accelerated intermittent theta burst stimulation treatment in medication-resistant major depression: A fast road to remission? J Affect Disord 2016;200:6-14.

89. van Lutterveld R, Koops S, Schutter DJ, et al. The effect of rTMS on auditory hallucinations: clues from an EEG-rTMS study. Schizophr Res 2012;137:174-179.
90. Nahas Z, Bohning DE, Molloy MA, Oustz JA, Risch SC, George MS. Safety and feasibility of repetitive transcranial magnetic stimulation in the treatment of anxious depression in pregnancy: a case report. J Clin Psychiatry 1999;60:50-52.
91. Klirova M, Novak T, Kopecek M, Mohr P, Strunzova V. Repetitive transcranial magnetic stimulation (rTMS) in major depressive episode during pregnancy. Neuro Endocrinol Lett 2008;29:69-70.
92. Widlöcher D, Ghozlan A. The measurement of retardation in depression. In: Hindmarch I, Stonier PD, eds. Human Psychopharmacology: Measures and Methods. New York: Wiley, 1989.
93. Kay SR, Fiszbein A, Opler LA. The positive and negative syndrome scale (PANSS) for schizophrenia. Schizophr Bull 1987;13:261-276.
94. Kirkpatrick B, Strauss GP, Nguyen L, et al. The brief negative symptom scale: psychometric properties. Schizophr Bull 2011;37:300-305.
95. Craig CL, Marshall AL, Sjostrom M, et al. International physical activity questionnaire: 12-country reliability and validity. Med Sci Sports Exerc 2003;35:1381-1395.
96. Dollfus S, Mach C, Morello R. Self-Evaluation of Negative Symptoms: A Novel Tool to Assess Negative Symptoms. Schizophr Bull 2016;42:571-578.
97. Endicott J, Spitzer RL, Fleiss JL, Cohen J. The global assessment scale. A procedure for measuring overall severity of psychiatric disturbance. Arch Gen Psychiatry 1976;33:766-771.
98. Goldman HH, Skodol AE, Lave TR. Revising axis V for DSM-IV: a review of measures of social functioning. Am J Psychiatry 1992;149:1148-1156.
99. Mausbach BT, Depp CA, Bowie CR, et al. Sensitivity and specificity of the UCSD Performance-based Skills Assessment (UPSA-B) for identifying functional milestones in schizophrenia. Schizophr Res 2011;132:165-170.
100. Huang YZ, Edwards MJ, Rounis E, Bhatia KP, Rothwell JC. Theta burst stimulation of the human motor cortex. Neuron 2005;45:201-206.
101. Oldfield RC. The assessment and analysis of handedness: the Edinburgh inventory. Neuropsychologia 1971;9:97-113.
102. Andreasen NC, Flaum M, Arndt S. The Comprehensive Assessment of Symptoms and History (CASH). An instrument for assessing diagnosis and psychopathology. Arch Gen Psychiatry 1992;49:615-623.
103. Kircher T, Krug A, Stratmann M, et al. A rating scale for the assessment of objective and subjective formal Thought and Language Disorder (TALD). Schizophr Res 2014;160:216-221.
104. Buchanan RW, Heinrichs DW. The Neurological Evaluation Scale (NES): a structured instrument for the assessment of neurological signs in schizophrenia. Psychiatry Res 1989;27:335-350.
105. Fahn S, Elton RL, Members UP. Unified Parkinson's disease rating scale. In: Fahn S, Marsden CD, Goldstein M, Calne DB, eds. Recent developments in Parkinson's disease. Florham Park, NJ: Macmillan Healthcare Information, 1987.
106. Bush G, Fink M, Petrides G, Dowling F, Francis A. Catatonia. I. Rating scale and standardized examination. Acta Psychiatr Scand 1996;93:129-136.
107. Guy W. ECDEU Assessment Manual for Psychopharmacology. Rockville, MD: US Department of Health, Education and

Welfare, 1976.
108. Walther S, Federspiel A, Horn H, et al. White matter integrity associated with volitional motor activity. Neuroreport 2010;21:381-385.
109. Walther S, Horn H, Koschorke P, Muller TJ, Strik W. Increased motor activity in cycloid psychosis compared to schizophrenia. World J Biol Psychiatry 2009;10:746-751.
110. Walther S, Horn H, Razavi N, Koschorke P, Muller TJ, Strik W. Quantitative motor activity differentiates schizophrenia subtypes. Neuropsychobiology 2009;60:80-86.
111. Walther S, Horn H, Razavi N, et al. Higher motor activity in schizophrenia patients treated with olanzapine versus risperidone. J Clin Psychopharmacol 2010;30:181-184.

112. Vanbellingen T, Wapp M, Stegmayer K, et al. Theta burst stimulation over premotor cortex in Parkinson's disease: an explorative study on manual dexterity. J Neural Transm (Vienna) 2016;123:1387-1393.

113. Rossini PM, Burke D, Chen R, et al. Non-invasive electrical and magnetic stimulation of the brain, spinal cord, roots and peripheral nerves: Basic principles and procedures for routine clinical and research application. An updated report from an I.F.C.N. Committee. Clin Neurophysiol 2015;126:1071-1107.

114. Claassen J, Spiegel R, Kalla R, et al. A randomised double-blind, cross-over trial of 4-aminopyridine for downbeat nystagmus--effects on slowphase eye velocity, postural stability, locomotion and symptoms. J Neurol Neurosurg Psychiatry 2013;84:1392-1399.

115. Krafczyk S, Tietze S, Swoboda W, Valkovic P, Brandt T. Artificial neural network: a new diagnostic posturographic tool for disorders of stance. Clin Neurophysiol 2006;117:1692-1698.

116. Genzel L, Dresler M, Cornu M, et al. Medial prefrontal-hippocampal connectivity and motor memory consolidation in depression and schizophrenia. Biol Psychiatry 2015;77:177-186.

117. Yokoi A, Arbuckle SA, Diedrichsen J. The Role of Human Primary Motor Cortex in the Production of Skilled Finger Sequences. J Neurosci 2018;38:1430-1442.

17. APPENDICES

1. IB (according to ISO 14155) – "Produktinformation"

2. TMS device manual

3. Case Report Forms, v2 of December 13th 2018, patient version and control version

4. Study Information for patients, v2 of December 13th 2018

5. Study Information for healthy controls, v2 of December 13th 2018

Overcoming psychomotor slowing in psychosis (OCoPS-P) – A 3-week, randomized, double-blind, placebo-controlled trial of add-on repetitive transcranial magnetic stimulation for psychomotor slowing in psychosis

Clinical Study Protocol

Short Title:	OCoPS-P
Translation	Überwinden von psychomotorischer Verlangsamung bei Psychosen - eine randomisierte, doppelblinde, plazebo-kontrollierte Studie zur Wirkung von transkranieller Magnetstimulation auf die psychomotorischer Verlangsamung bei Psychosen

Study Type:	Clinical trial testing the effects of 15 sessions of repetitive transcranial magnetic stimulation on psychomotor slowing in psychosis
Study Categorisation:	Risk category A
Study Registration:	Clinicaltrials.gov NCT03921450
Study Identifier:	OCoPS-P, BASEC-Nr: 2018-02164
Sponsor, Sponsor-Investigator or Principal Investigator:	Prof. Dr. med. Sebastian Walther, University Hospital of Psychiatry, University of Bern, Murtenstrasse 21, 3008 Switzerland<br>Phone: 031 632 8979, fax: 031 632 8950, Email: sebastian.walther@upd.unibe.ch
Investigational Product:	Transcranial Magnetic Stimulation
Protocol Version and Date:	Version 3.0 June 10th 2020

CONFIDENTIAL

The information contained in this document is confidential and the property of Prof. Dr. med. Sebastian Walther (the sponsor). The information may not - in full or in part - be transmitted, reproduced, published, or disclosed to others than the applicable Competent Ethics Committee(s) and Regulatory Authority(ies) without prior written authorisation from the sponsor except to the extent necessary to obtain informed consent from those who will participate in the study.

Signature Page(s)

Study number	BASEC 2018-02164
Study Title	Overcoming psychomotor slowing in psychosis (OCoPS-P) – A 3-week, randomized, double-blind, placebo-controlled trial of add-on repetitive transcranial magnetic stimulation for psychomotor slowing in psychosis

The Sponsor-Investigator and trial statistician have approved the protocol version 3.0 (10.06.2020), and confirm hereby to conduct the study according to the protocol, current version of the World Medical Association Declaration of Helsinki, ICH-GCP guidelines or ISO 14155 norm if applicable and the local legally applicable requirements.

Sponsor-Investigator:
Prof. Dr. med. Sebastian Walther

Bern, 11.06.2020 S. Walther

Place/Date Signature

Local Principal Investigator at study site*:

I have read and understood this trial protocol and agree to conduct the trial as set out in this study protocol, the current version of the World Medical Association Declaration of Helsinki, ICH-GCP guidelines or ISO 14155 norm and the local legally applicable requirements.

Site	University Hospital of Psychiatry, Bern
Principal investigator	Prof. Dr. med. Sebastian Walther

______________________________ ______________________________________

Place/Date Signature

Note: In multicentre studies, this page must be individually signed by all participating Local Principal Investigators.

Table of Contents

STUDY SYNOPSIS **7**
ABBREVIATIONS **11**
STUDY SCHEDULE **12**
1. STUDY ADMINISTRATIVE STRUCTURE **14**
1.1 Sponsor, Sponsor-Investigator 14
1.2 Principal Investigator(s) 14
1.3 Statistician ("Biostatistician") 14
1.4 Laboratory 14
1.5 Monitoring institution 14
1.6 Data Safety Monitoring Committee 14
1.7 Any other relevant Committee, Person, Organisation, Institution 14
2. ETHICAL AND REGULATORY ASPECTS **15**
2.1 Study registration 15
2.2 Categorisation of study 15
2.3 Competent Ethics Committee (CEC) 15
2.4 Competent Authorities (CA) 15
2.5 Ethical Conduct of the Study 15
2.6 Declaration of interest 15
2.7 Patient Information and Informed Consent 15
2.8 Participant privacy and confidentiality 16
2.9 Early termination of the study 16
2.10 Protocol amendments 16
3. BACKGROUND AND RATIONALE **17**
3.1 Background and Rationale 17
3.1.1 Schizophrenia 17
3.1.2 Motor system pathology in schizophrenia 17
3.1.3 Psychomotor slowing 17
3.1.4 Current treatment options of psychomotor slowing 18
3.1.5 Psychomotor slowing and motor network dysfunction 18
3.1.6 Intracortical excitability 19
3.1.7 Brain stimulation for psychomotor slowing 19
3.1.8 State of research summary 20
3.2 Investigational Product (treatment, device) and Indication 20
3.3 Preclinical Evidence 20
3.4 Clinical Evidence to Date 21
3.5 Dose Rationale / Medical Device: Rationale for the intended purpose in study (pre-market MD) 21
3.6 Explanation for choice of comparator (or placebo) 21
3.7 Risks / Benefits 21
3.8 Justification of choice of study population 22
4. STUDY OBJECTIVES **23**
4.1 Overall Objective 23
4.2 Primary Objective 23
4.3 Secondary Objectives 23
4.4 Safety Objectives 23
5. STUDY OUTCOMES **23**
5.1 Primary Outcome 23

5.2 Secondary Outcomes 23
5.2.1 Clinical outcomes 23
5.2.2 Behavioral outcomes 24
5.2.3 Physiological outcomes 24
5.2.4 Neuroimaging outcomes 24
5.3 Other Outcomes of Interest 24
5.4 Safety Outcomes 24
6. STUDY DESIGN 24
6.1 General study design and justification of design 24
6.2 Methods of minimising bias 25
6.2.1 Randomisation 25
6.2.2 Blinding procedures 25
6.2.3 Other methods of minimising bias 25
6.3 Unblinding Procedures (Code break) 25
7. STUDY POPULATION 25
7.1 Eligibility criteria 25
7.2 Recruitment and screening 26
7.3 Assignment to study groups 26
7.4 Criteria for withdrawal / discontinuation of participants 26
8. STUDY INTERVENTION 27
8.1 Identity of Investigational Products (treatment / medical device) 27
8.1.1 Experimental Intervention (treatment / medical device) 27
8.1.2 Control Intervention (standard/routine/comparator treatment / medical device) 27
8.1.3 Packaging, Labelling and Supply (re-supply) 27
8.1.4 Storage Conditions 27
8.2 Administration of experimental and control interventions 27
8.2.1 Experimental Intervention 27
8.2.2 Control Intervention 28
8.3 Dose / Device modifications 28
8.4 Compliance with study intervention 28
8.5 Data Collection and Follow-up for withdrawn participants 28
8.6 Trial specific preventive measures 28
8.7 Concomitant Interventions (treatments) 28
8.8 Study Drug / Medical Device Accountability 28
8.9 Return or Destruction of Study Drug / Medical Device 28
9. STUDY ASSESSMENTS 29
9.1 Study flow chart(s) / table of study procedures and assessments 29
9.1.1 Patients of treatment groups 1-3 29
9.1.2 Patients of treatment group 4 (waiting group) 31
9.1.3 Control subjects 35
9.2 Assessments of outcomes 36
9.2.1 Assessment of primary outcome 36
9.2.2 Assessment of secondary outcomes 37
9.2.3 Assessment of other outcomes of interest 38
9.2.4 Assessment of safety outcomes 38
9.2.5 Assessments in participants who prematurely stop the study 39
9.3 Procedures at each visit 39
9.3.1 Screening visit 39

9.3.2 Visit 2 (baseline, week 0) ... 39
9.3.3 Visit 3 and 4 (week 1 and 2) ... 39
9.3.4 Visit 5 (end of intervention, week 3) ... 39
9.3.5 Visit 6 and 7 (follow-up, week 6 and 24) ... 40
10. SAFETY ... 40
10.1 Medical Device Category A studies ... 40
10.1.1 Definition and Assessment of safety related events ... 40
10.1.2 Reporting of (Serious) Adverse Events and other safety related events ... 41
10.1.3 Follow up of (Serious) Adverse Events ... 42
11. STATISTICAL METHODS ... 42
11.1 Hypotheses ... 42
11.2 Determination of Sample Size ... 43
11.3 Statistical criteria of termination of trial ... 43
11.4 Planned Analyses ... 43
11.4.1 Datasets to be analysed, analysis populations ... 43
11.4.2 Primary Analysis ... 43
11.4.3 Secondary Analyses ... 43
11.4.4 Interim analyses ... 44
11.4.5 Safety analysis ... 44
11.4.6 Deviation(s) from the original statistical plan ... 44
11.5 Handling of missing data and drop-outs ... 44
12. QUALITY ASSURANCE AND CONTROL ... 45
12.1 Data handling and record keeping / archiving ... 45
12.1.1 Case Report Forms ... 45
12.1.2 Specification of source documents ... 45
12.1.3 Record keeping / archiving ... 45
12.2 Data management ... 45
12.2.1 Data Management System ... 46
12.2.2 Data security, access and back-up ... 46
12.2.3 Analysis and archiving ... 46
12.2.4 Electronic and central data validation ... 46
12.3 Monitoring ... 46
12.4 Audits and Inspections ... 47
12.5 Confidentiality, Data Protection ... 47
12.6 Storage of biological material and related health data ... 47
13. PUBLICATION AND DISSEMINATION POLICY ... 47
14. FUNDING AND SUPPORT ... 47
14.1 Funding ... 47
14.2 Other Support ... 47
15. INSURANCE ... 48
16. REFERENCES ... 49
17. APPENDICES ... 55

STUDY SYNOPSIS

**Sponsor / Sponsor-Investigator**	Prof. Dr. med. Sebastian Walther,<br>University Hospital of Psychiatry, University of Bern, Murtenstrasse 21, 3008 Switzerland<br>Phone: 031 632 8979, fax: 031 632 8950, Email: sebastian.walther@upd.unibe.ch
**Study Title:**	Overcoming psychomotor slowing in psychosis (OCoPS-P) – A 3-week, randomized, double-blind, placebo-controlled trial of add-on repetitive transcranial magnetic stimulation for psychomotor slowing in psychosis
**Short Title / Study ID:**	OCoPS-P, KEK 2018-02164
**Protocol Version and Date:**	Protocol version 3.0 (10th June 2020)
**Trial registration:**	clinicaltrials.gov, NCT03921450
**Study category and Rationale**	Category A: TMS device with CE certificate and use according to guidelines and manual. In addition short cerebral MRI and TMS experiments at baseline and endpoint. Minimal risk involved.
**Clinical Phase:**	Not applicable.

**Background and Rationale:**	Schizophrenia is a chronic disorder causing tremendous burden to the patients, families and society. Besides prominent symptoms such as hallucinations, delusions, and thought disorder, the majority of patients also experiences motor abnormalities. Converging evidence links aberrant structure and function of the cerebral motor network to schizophrenia pathology, particularly to motor abnormalities. One of the most frequent motor abnormalities is psychomotor slowing (PS), which may impact both gross and fine motor behavior. While PS causes significant distress and predicts poor outcome, researchers are just starting to understand its pathobiology. First evidence points to aberrant functional and structural connectivity within the cerebral motor network in schizophrenia patients with PS, particularly in connections between premotor/motor cortex and thalamus, as well as between motor cortex and cerebellum. In addition, severe motor inhibition was linked to increased neural activity in the premotor cortex. Repetitive transcranial magnetic stimulation (rTMS) may temporarily alter brain activity. Pilot data from an ongoing double blind RCT indicate that 15 sessions of inhibitory rTMS on the premotor cortex alleviate PS. The pathobiology of PS, however, is still unknown. This study will combine a motor battery, advanced neuroimaging, and rTMS to probe the cerebral motor network contributions to PS.<br>The aims of this study are<br>(1) to investigate clinical and neural changes following 3 weeks of rTMS treatment (inhibitory, facilitatory or placebo), (2) to characterize neural correlates of psychomotor slowing (PS) on the network level, (3) to explore short term changes of PS by testing a waiting list cohort, and (4) to test the clinical outcome of rTMS for PS at 6-month follow-up.<br>To reach these aims, we plan to investigate four groups of schizophrenia patients (total 88) in a randomized, double blind, 4-arm sham-controlled trial of 15 rTMS sessions in 3 weeks with pre and post intervention MRI scans and a clinical follow-up at 6 months. One group will first be kept on a 3 week waiting list and then enter the study. Longitudinal MRI scans and motor tests separated by 3 weeks will also be applied to a control group of 40 healthy subjects for comparisons with the patient groups. We hypothesize that (1) PS would be linked to increased functional connectivity in motor cortical-basal ganglia loops as well as motor cortical-cerebellar loops, (2) inhibitory rTMS to the premotor cortex will reduce motor network functional connectivity and thus alleviate PS, (3) patients on the waiting list may experience stable PS severity, and finally, (4) patients responding to rTMS treatment of PS will have superior clinical and functional outcomes at 6-month follow-up. Thus, the study will substantially contribute to the understanding of PS by describing and probing the neural alterations in the motor network in schizophrenia associated with behavioral PS. Therefore, the study will impact future treatment strategies for PS and inform on the causal network pathology in schizophrenia
**Objective(s):**	1. investigate clinical and neural changes following 3 weeks of rTMS treatment (inhibitory, facilitatory or placebo)<br>2. characterize neural correlates of psychomotor slowing (PS) on the network level<br>3. explore short term changes of PS by testing a waiting list cohort<br>4. test the clinical outcome of rTMS for PS at 6-month follow-up.

**Outcome(s):**	1. Change from baseline to week 3 in SRRS scores. Furthermore, proportion of responders per study arm after 15 sessions rTMS (response = 30% reduction in SRRS scores from baseline)<br>2. Associations between multiple measures of motor function and neuroimaging markers, e.g. resting state perfusion or functional connectivity within the motor system<br>3. Change in SRRS from baseline to week 3 within the waiting list group, also comparison of SRRS change from baseline between waiting list group and placebo group.<br>4. Change in symptoms (PANSS, BNSS, SRRS) and functioning (SOFAS) from baseline to 6-month follow-up
**Study design:**	randomised, double-blind, four-arm, placebo-controlled trial of 3 weeks add-on rTMS for psychomotor slowing in schizophrenia spectrum disorders
**Inclusion / Exclusion criteria:**	Inclusion:<br>• Right-handed subjects, ages 18–60 years.<br>• Patients: schizophrenia spectrum disorders according to DSM-5 with psychomotor slowing (SRRS score ≥ 15). Patients are necessary, because only patients have the target symptoms, i.e. psychomotor slowing<br>• Controls: only for pre-/post comparisons of neuroimaging and physiology, **no intervention in controls**<br>Exclusion:<br>• General: Substance abuse or dependence other than nicotine. Past or current medical or neurological condition associated with impaired or aberrant movement, such as brain tumors, stroke, M. Parkinson, M. Huntington, dystonia, or severe head trauma with subsequent loss of conciousness. Epilepsy or other convulsions. History of any hearing problems or ringing in the ears. Standard exclusion criteria for MRI scanning and TMS; e.g. metal implants, claustrophobia. Women who are pregnant or breast feeding<br>• Patients only: any TMS treatment in the past 3 months<br>• Controls: history of any psychiatric disorder. First-degree relatives with schizophrenia spectrum disorders.
**Measurements and procedures:**	Participants will be screened and randomized to one of four arms before baseline assessments. Intervention period will be three weeks. Each week the primary outcome variable and safety will be assessed. At baseline and end of intervention (week 3), patients will be assessed with clinical and motor rating scales, tasks assessing fine and gross motor behaviour, TMS measures of cortical excitability, posturography, MRI neuroimaging, and tests of social and community functioning. Follow-ups will be conducted at week 6 and 24 including clinical and motor measures, cortical excitability, and social and community functioning. For cross-sectional comparisons of cortical excitability and neuroimaging, a group of 40 healthy control subjects matched for age, gender, and education, will be tested longitudinally with neuroimaging, motor tests, cortical excitability and posturography at baseline and week 3. Controls will not receive any intervention.
**Study Product / Intervention:**	low-frequency rTMS has inhibitory effects on brain function. We will apply 1’000 pulses at 1 Hz over the left SMA at an intensity of 110% of the resting motor threshold (approximate duration 17 minutes) in a total of 15 daily sessions (5 per week)

**Control Intervention (if applicable):**	**Active control:** Intermittent theta burst (iTBS) enhances local brain activity. We will apply 600 pulses at 50 Hz (stimulation in 2 sec trains every 10 sec for 190 sec in total) over the left SMA at an intensity of 80% of the resting motor threshold. iTBS will be repeated after15 min totaling to 1200 pulses per session in a total of 15 daily sessions (5 per week).<br><br>**Placebo control:** We will use a placebo-coil that looks identical to the real one and makes identical noises. Stimulation parameters are the same as in the active intervention, except that no magnetic pulse is emitted. Thus, placebo coil will be placed over the left SMA with 1000 clicks at 1 Hz (approximate duration 17 minutes) in a total of 15 daily sessions (5 per week).<br><br>**Waiting list:** This group will have baseline measures at baseline and week 3 and receive the active protocol from week 3 to week 6.
**Number of Participants with Rationale:**	Number of participants in the intervention: 88 patients (22 per protocol arm). In addition, to complement neuroimaging and neurophysiological analyses (no intervention) we will include 40 healthy control subjects matched for age, gender, and education.
**Study Duration:**	Total study duration will be 4 years. Total duration of participant recruitment will be 3 years.
**Study Schedule:**	Planned 03/2019 of First-Participant-In<br>Planned 06/2022 of Last-Participant-Out
**Investigator(s):**	- see Sponsor-Investigator
**Study Centre(s):**	Single-centre trial at the University Hospital of Psychiatry, Bern
**Statistical Considerations:**	The main effects of the 3-week interventions on PS (SRRS scores) will be calculated in a repeated measures design including 2 measures (Baseline, week 3) and four groups (lf-rTMS, iTBS, sham, waiting group-lf-rTMS). Assuming a moderate effect size (f = 0.23) as indicated by our pilot data in a repeated measures ANOVA with moderate correlation between time points (0.5), a power of 0.95 and an alpha = 0.05, we would need 88 patients (22 per group).
**GCP Statement:**	This study will be conducted in compliance with the protocol, the current version of the Declaration of Helsinki, the ICH-GCP or ISO EN 14155 (as far as applicable) as well as all national legal and regulatory requirements.

ABBREVIATIONS

AE	Adverse Event
BASEC	Business Administration System for Ethical Committees, (https://submissions.swissethics.ch/en/)
BNSS	Brief Negative Symptom Scale
CA	Competent Authority (e.g. Swissmedic)
CEC	Competent Ethics Committee
CRF	Case Report Form
ClinO	Ordinance on Clinical Trials in Human Research *(in German: KlinV, in French: OClin, in Italian: OSRUm)*
eCRF	Electronic Case Report Form
CTCAE	Common terminology criteria for adverse events
DSUR	Development safety update report
GCP	Good Clinical Practice
IB	Investigator's Brochure
Ho	Null hypothesis
H1	Alternative hypothesis
HRA	Federal Act on Research involving Human Beings *(in German: HFG, in French: LRH, in Italian: LRUm)*
IMP	Investigational Medicinal Product
IIT	Investigator-initiated Trial
ISO	International Organisation for Standardisation
iTBS	Intermittent Theta Burst Stimulation
ITT	Intention to treat
MD	Medical Device
MedDO	Medical Device Ordinance *(in German: MepV, in French: ODim)*
PANSS	Positive And Negative Syndrome Scale
PI	Principal Investigator
PS	Psychomotor Slowing
rTMS	Repetitive Transcranial Magnetic Stimulation
SAE	Serious Adverse Event
SDV	Source Data Verification
SMA	Supplementary Motor Area
SOP	Standard Operating Procedure
SPC	Summary of product characteristics
SRRS	Salpêtrière Retardation Rating Scale
SOFAS	Social and Occupational Functioning Scale
SUSAR	Suspected Unexpected Serious Adverse Reaction
TMF	Trial Master File
UPSA Brief	UCSD Performance-based Skills Assessment Brief version

STUDY SCHEDULE

Study Periods	Screening	Treatment, Intervention Period				Follow-up	
Visit	1	2*	3	4	5	6	7
Time (week)	-1	0	1	2	3	6	24
Patient Information and Informed Consent	x						
Demographics	x						
CASH and SCID (history)	x						
In- /Exclusion Criteria	x						
Physical Examination	x						
Pregnancy Test	x						
Psychopathology (TALD, NES)		x					
Randomisation		x					
Administer rTMS (5 sessions per week)		x	x	x			
Primary Variable SRRS		x	x	x	x	x	x
Motor scales UPDRS, BFCRS, AIMS, IPAQ		x			x	x	x
Psychopathology (PANSS, BNSS, SNS)		x			x	x	x
Actigraphy and coin rotation		x			x	x	x
Posturography		x			x		
TMS cortical excitability		x			x	x	x
Grip force measurement		x			x	x	x
Cerebral MRI		x			x		
Blood sampling		x			x		
Functional outcome (SOFAS, GAF, UPSA-brief)		x				x	x
Concomitant Therapy		x	x	x	x		
Adverse Events		x	x	x	x		

* please note that in the waiting group assessments of visit 2 will be repeated after 3 weeks and thereafter the protocol will be identical (see below).

Study protocol for patients in the waiting group

Study Periods	Scree ning	Treatment, Intervention Period					Follow-up	
Visit	1	2	3	4	5	6	7	8
Time (week)	-1	0	3	4	5	6	9	24
Patient Information and Informed Consent	x							
Demographics	x							
CASH and SCID (history)	x							
In- /Exclusion Criteria	x							
Physical Examination	x							
Pregnancy Test	x							
Psychopathology (TALD, NES)		x						
Randomisation		x						
Administer rTMS (5 sessions per week)			x	x	x			
Primary Variable SRRS		x	x	x	x	x	x	x
Motor scales UPDRS, BFCRS, AIMS, IPAQ		x	x			x	x	x
Psychopathology (PANSS, BNSS, SNS)		x	x			x	x	x
Actigraphy and coin rotation		x	x			x	x	x
Posturography		x	x			x		
TMS cortical excitability		x	x			x	x	x
Grip force measurement		X	X			X	X	X
Cerebral MRI		x	x			x		
Blood sampling		X				X		
Functional outcome (SOFAS, GAF, UPSA-brief)		x	x				x	x
Concomitant Therapy		x	x	x	x	x		
Adverse Events			x	x	x	x		

Study protocol for control subjects

Study Periods	Screening	Observation period	
Visit<br>Time (week)	1<br>-1	2<br>0	3<br>3
Proband Information and Informed Consent	x		
Demographics	x		
SCID	x		
In- /Exclusion Criteria	x		
Physical Examination	x		
Pregnancy Test	x		
Primary Variable SRRS		x	x
Motor scales UPDRS, BFCRS, AIMS, IPAQ		x	x
Actigraphy and coin rotation		x	x
Posturography		x	x
TMS cortical excitability		x	x
Grip force measurement		X	X
Cerebral MRI		X	X
Blood sampling		X	X

1. STUDY ADMINISTRATIVE STRUCTURE

1.1 Sponsor, Sponsor-Investigator

Prof. Dr. med. Sebastian Walther,

University Hospital of Psychiatry, University of Bern, Murtenstrasse 21, 3008 Bern, Switzerland, phone: 031 632 4635, fax: 031 632 8950, Email: sebastian.walther@upd.unibe.ch

Prof. Walther will be Sponsor-Investigator; no further international sites are planned

Roles: protocol, study design, supervision of data collection and management, data analysis, data interpretation and writing of the report.

1.2 Principal Investigator(s)

Identical to sponsor, see 1.1.

1.3 Statistician ("Biostatistician")

Dr. Petra Viher, PhD, University Hospital of Psychiatry, University of Bern, Bolligenstrasse 111, 3000 Bern, Switzerland. Phone: 031 930 9757, Email: petra.viher@upd.unibe.ch

1.4 Laboratory

All of the procedures except neuroimaging will be performed at the University Hospital of Psychiatry, Bern. The devices and infrastructure are provided by the Translational research center at the University Hospital of Psychiatry, Bern, Bolligenstrasse 111, 3000 Bern 60, Switzerland.

Neuroimaging acquisition (magnetic resonance imaging – MRI) will be performed at the University Hospital Inselspital Bern, Institute of Diagnostic and Interventional Neuroradiology. Collaborator Prof. Dr. med. Roland Wiest.

1.5 Monitoring institution

Dr. phil. Jessica Peter, University Hospital of Old Age Psychiatry and Psychotherapy, University of Bern, Bolligenstrasse 111, 3000 Bern, Switzerland. Phone: 031 932 8903 Email: Jessica.peter@upd.unibe.ch

1.6 Data Safety Monitoring Committee

DSMC is not needed. The study aim is not to test the efficacy of a specific product. The objective is to test whether repetitive transcranial magnetic stimulation may improve psychomotor slowing and how it interferes with neurophysiology.

1.7 Any other relevant Committee, Person, Organisation, Institution

Study collaborators

Prof. Dr. Roland Wiest, University Institute of Diagnostic and Interventional Neuroradiology, Inselspital Bern, Switzerland. MRI acquisition

Prof. Dr. Andrea Federspiel, Neuroimaging Unit, Translational Research Center, University Hospital of Psychiatry, Bern, Switzerland, MRI analyses

Prof. Dr. Jessica Bernard, Department of Psychological and Brain Sciences, Texas A & M University, College Station, TX, USA, MRI analyses support

Prof. Dr. Roger Kalla, Department of Neurology, University Hospital Inselspital, Bern, Switzerland, posturography

Prof. Kim Q. Do, Center of Psychiatric Neuroscience, Department of Psychiatry, Lausanne University Hospital, Lausanne, Switzerland. Blood Analysis

2. ETHICAL AND REGULATORY ASPECTS

The decision of the CEC concerning the conduct of the study will be made in writing to the Sponsor-Investigator before commencement of this study. The clinical study can only begin once approval from all required authorities has been received. Any additional requirements imposed by the authorities shall be implemented.

2.1 Study registration

Registration is planned in ClinicalTrials.gov and the Swiss KOFAM site

2.2 Categorisation of study

The trial is in Risk Category A. There is only minimal risk associated with the trial. The TMS device has CE certification and approved for clinical use. It will be applied according to the manual and guidelines[1, 2]. TMS has been widely used in neuroscience, and in clinical trials on depression, schizophrenia and chronic pain. Participants will receive 15 daily stimulations. Effects are expected to last for 2-4 weeks after the last stimulation. TMS is also safe in repeated administration[1, 2].

Assessments include standard clinical rating scales, short specific tests of motor behaviour, and a standard cerebral magnetic resonance imaging at baseline and after 3 weeks of stimulation. All assessments have been applied to schizophrenia patients before and are generally well tolerated. In addition, biological material is sampled for further research use.

2.3 Competent Ethics Committee (CEC)

The local Bern Ethics Committee is the Competent Ethics Committee (CEC). No sites outside the canton of Bern are planned.

The Bern Ethics Committee will receive reports of all changes in the research activity and all unanticipated problems involving risks to humans; including in case of planned or premature study end and the final report. No changes are made to the protocol without prior Sponsor and CEC approval, except where necessary to eliminate apparent immediate hazards to study participants. Premature study end or interruption of the study is reported within 15 days. The regular end of the study is reported to the CEC within 90 days, the final study report shall be submitted within one year after study end. Amendments are reported according to chapter 2.10.

2.4 Competent Authorities (CA)

Not applicable – Category A

2.5 Ethical Conduct of the Study

The study will be carried out in accordance to the protocol and with principles enunciated in the current version of the Declaration of Helsinki, the guidelines of Good Clinical Practice (GCP) issued by ICH, in case of medical device: the European Regulation on medical devices 2017/745 and the ISO Norm 14155 and ISO 14971, the Swiss Law and Swiss regulatory authority's requirements. The CEC and regulatory authorities will receive annual safety and interim reports and be informed about study stop/end in agreement with local requirements.

2.6 Declaration of interest

There is no conflict of interest. Funding is provided by independent grants and the Swiss National Science Foundation (see funding 14.1)

2.7 Patient Information and Informed Consent

Participants will be informed by members of the study team about the aims of the study, planned procedures and risks involved. They will receive written information on the study. This information will be provided prior to study inclusion during screening. The participants will also be informed about the compensation of 200 CHF after they completed the study procedures. The participant will be informed by the investigators that his/her medical records may be examined by authorised individuals other than their treating physician.

All participants for the study will be provided a participant information sheet and a consent form describing the study and providing sufficient information for participant to make an informed decision about their participation in the study. A minimum of 24 h time will be given to participants to decide on whether to participate or not. Whenever necessary, the potential participant can take up to 2 weeks to decide on participation.

The patient information sheet and the consent form will be submitted to the CEC and to the competent authority (as applicable) to be reviewed and approved. The formal consent of a participant, using the approved consent form, must be obtained before the participant is submitted to any study procedure.

The participant should read and consider the statement before signing and dating the informed consent form, and should be given a copy of the signed document. The consent form must also be signed and dated by the investigator (or his designee) and it will be retained as part of the study records.

2.8 Participant privacy and confidentiality

The investigator affirms and upholds the principle of the participant's right to privacy and that they shall comply with applicable privacy laws. Especially, anonymity of the participants shall be guaranteed when presenting the data at scientific meetings or publishing them in scientific journals.

Individual subject medical information obtained as a result of this study is considered confidential and disclosure to third parties is prohibited. Subject confidentiality will be further ensured by utilising subject identification code numbers to correspond to treatment data in the computer files.

For data verification purposes, authorised representatives of the Sponsor (-Investigator) or an ethics committee may require direct access to parts of the medical records relevant to the study, including participants' medical history.

2.9 Early termination of the study

The Sponsor-Investigator may terminate the study prematurely according to certain circumstances, for example:

- ethical concerns,
- insufficient participant recruitment,
- when the safety of the participants is doubtful or at risk, respectively,
- alterations in accepted clinical practice that make the continuation of a clinical trial unwise,
- early evidence of benefit or harm of the experimental intervention

2.10 Protocol amendments

Substantial amendments are only implemented after approval of the CEC and CA respectively.

Under emergency circumstances, deviations from the protocol to protect the rights, safety and well-being of human subjects may proceed without prior approval of the sponsor and the CEC/CA. Such deviations shall be documented and reported to the sponsor and the CEC/CA as soon as possible.

All non-substantial amendments are communicated to the CA as soon as possible if applicable and to the CEC within the Annual Safety Report (ASR).

3. BACKGROUND AND RATIONALE

3.1 Background and Rationale

3.1.1 Schizophrenia

Schizophrenia is a severe disorder with a life-long course affecting approximately 2-3% of the population. Even though the outcome is heterogeneous, schizophrenia usually causes tremendous individual burden, intense costs for society, reduced quality of life, impaired occupational performance and reduction of life expectancy by 10-20 years[3]. Schizophrenia is an adolescent-onset disorder with neurodevelopmental origins[3]. Current models suggest that genetic risk, early hazards to brain maturation, social adversities during childhood and the evolution of cognitive biases predispose subjects to psychosis in times of stress[4]. The schizophrenia syndrome is characterized by symptom clusters including positive symptoms (e.g. delusions and hallucinations), negative symptoms (e.g. avolition and affective flattening), disorganized thought, motor abnormalities, mood disturbances, impaired cognition, anxiety and lack of insight[5]. Brain structure and function in schizophrenia is abnormal at multiple levels. For example, schizophrenia patients share altered organization of functional brain networks[6] and underlying white matter fiber connections[7, 8]. Therefore, schizophrenia has been conceptualized as a disconnection syndrome, which may explain some of the typical symptoms[9]. Indeed, first evidence indicates an association between abnormal motor behavior and structural as well as functional alterations in the motor network in schizophrenia[10, 11]. Researchers are just starting to understand the network alterations linked to clinical symptoms in schizophrenia[12]. **The ultimate goal** would be to **normalize altered brain function at the network level** in order to **improve the clinical significant behavioral abnormalities**.

3.1.2 Motor system pathology in schizophrenia

A set of motor abnormalities including signs such as bizarre posture, peculiar gait, facial and limb dyskinesia, immobility, rigor, or excessive movement have been reported in schizophrenia.

Motor abnormalities have long been exclusively linked to medication side effects; however, converging evidence demonstrates that motor abnormalities frequently occur before medication is commenced and even long before the onset of psychosis[10, 13-15]. Up to 67% of first episode, treatment naïve patients experience at least one motor sign[16]. The contribution of medication is heterogeneous and probably overestimated.

Motor abnormalities are key cues for stigmatization and pose significant distress on patients[17, 18]. Furthermore, recent evidence supports the predictive value of motor abnormalities in psychosis for poor functional outcome[15, 19]. For example, motor abnormalities impact nonverbal behaviors such as gestures[20], which are critical for social functioning[21]. This way, motor abnormalities contribute to poor functional outcome in schizophrenia.

A large network of the brain is devoted to motor behavior, including cortical frontal motor and premotor areas, the basal ganglia, thalamus, brainstem and cerebellum (see figure 1). Evidence across multiple neuroimaging approaches supports that schizophrenia pathobiology is tied to alterations within the cerebral motor system[22].

Figure 1. Key components of the motor network

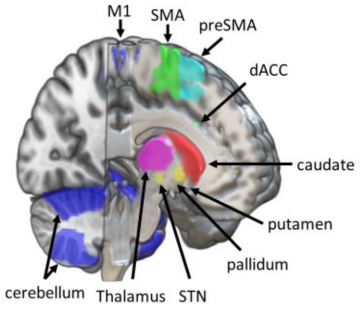

M1 primary motor cortex

SMA supplementary motor area

pre-SMA pre-supplementary motor area

dACC dorsal anterior cingulate cortex

STN subthalamic nucleus

3.1.3 Psychomotor slowing

One important domain of motor abnormalities in schizophrenia is psychomotor slowing (PS), which can

be observed in fine motor behavior such as writing, gross motor behavior such as gait, and it may refer to single movements or continuous movement. PS includes movement planning, initiation, execution, timing and motor control [15, 23-25]. Typical examples of PS in schizophrenia are reduced levels of spontaneous gross motor activity as measured by actigraphy[11, 26-28], slowed gait[29], slowed aiming arm movements[30], slowing in fine motor tasks[26, 31, 32] or in bradykinesia of parkinsonism[31].

Multiple reports suggest that 30-50% of schizophrenia patients present with PS[33, 34]. Furthermore, PS is seen in all stages of the disorder. Even though, PS severity seems to increase with illness chronicity[35], it does also occur in early psychosis or in subjects at risk for psychosis[34, 36-40]. In psychosis, PS is critically linked to several disadvantages[23]. For example, PS is associated with poor cognition, sedentary behavior and cardiometabolic risks[41, 42]. Moreover, PS correlates with distress and poor quality of life[17, 43, 44]. Finally, several reports indicate that PS predicts poor outcome in terms of cognition, quality of life and real world functioning in early psychosis, although at baseline patients with PS were not specifically impaired in function[36, 45-47]. In sum, **PS is a frequently observed phenomenon across schizophrenia stages**, associated with poor quality of life, sedentary behavior and predictive of poor cognition and outcome. In order to design new treatment options to overcome this problematic symptom domain, it is essential to know the pathobiology of PS.

PS is particularly suitable for objective instrumental assessment. There are valid measures of fine motor and gross motor slowing that can be acquired by instrumentation[15]. These measures allow dimensional assessment of PS and are therefore ideal candidates when exploring associations between brain and behavior. Instrumentation is also not prone to conceptual overlap. We have applied wrist actigraphy in a series of studies and repeatedly demonstrated reduced gross motor activity in schizophrenia, which was linked to negative syndrome severity and psychomotor slowing as measured by the Salpêtrière Retardation Rating Scale (SRRS)[26, 48-50]. Likewise, finger tapping test as a measure of PS in fine motor behavior is also consistently poorer in schizophrenia patients and linked to negative symptoms[31]. In addition, the video rated coin rotation test is a simple and reliable measure of manual dexterity[51, 52]. Another interesting marker of motor behavior is increased postural sway, which results from poor cerebellar function and is increased in psychosis[53, 54]. Postural sway was correlated with negative syndrome severity in patients[53, 54]. Therefore, we expect increased postural sway to be associated with PS in schizophrenia. In sum, **PS can be reliably measured by observer ratings** such as the **SRRS** or by **instrumental measures** of gross motor behavior (**activity level** from actigraphy) or fine motor behavior (**finger tapping** and **coin rotation**).

3.1.4 Current treatment options of psychomotor slowing

As mentioned above, motor symptoms including PS are neither simply explained by antipsychotic drug effects[23] nor does PS disappear after antipsychotic drug withdrawal. An excellent study in treatment naïve first episode subjects demonstrated the heterogeneity of drug effects on motor abnormalities, with 30% of patients in whom parkinsonism or catatonia was ameliorated by antipsychotic drugs[55]. Likewise, in many studies of our own group and others we failed to detect a correlation between antipsychotic dosage and measures of PS[11, 26, 31, 49, 50]. However, symptoms tend to improve with treatment but from the naturalistic studies conducted it is currently unclear, whether the improvement is due to a direct effect on motor behavior or whether the benefit results from amelioration of other psychosis symptoms, such as disorganization or avolition[56, 57]. Our own study on the longitudinal course of PS in acute schizophrenia found an amelioration of PS with treatment, which was tightly linked to a decline of negative symptom scores[50]. Finally, there are no trials demonstrating a beneficial effect of antipsychotic medication or trainings to improve psychomotor slowing in schizophrenia. Thus, **alternative treatment options for PS are clearly needed**.

3.1.5 Psychomotor slowing and motor network dysfunction

Conceptually, various aspects of motor behavior are modulated by three key circuits: inhibition and excitation of movements is related to a circuit including pre-/motor cortex and basal ganglia, timing of movements is linked to another circuit including motor cortex, thalamus and cerebellum, while psychomotor speed and planning involves a cortical network including medial prefrontal cortex, cingulate motor areas, SMA, M1, and posterior parietal cortex[12]. However, PS is not exclusively related to one of the abovementioned behaviors and probably involves all of the three circuits, even though the

basal ganglia circuit is the most probable [22].

The current understanding of the pathology in PS is limited due to methodological issues, such as focus on single brain regions, single neuroimaging modalities, and heterogeneous patient samples[10, 22]. Two types of approaches have mainly been adopted: first, actigraphically assessed motor activity levels were correlated with structural and functional magnetic resonance imaging (MRI) markers in the brain. Results suggested that unlike in controls, motor activity levels are not associated with **structural connectivity within the basal ganglia loop** in schizophrenia. Instead, motor activity was linked to **cortical motor loops**, which was interpreted as compensatory mechanism because patients with PS (i.e. lower activity) had particularly lower CBF and reduced white matter integrity within these cortical motor loops[27, 58-60]. Likewise, white matter ultrastructure of the corpus callosum and cingulum mediated psychomotor speed in schizophrenia[61]. A second line of evidence stems from functional and structural MRI studies testing associations with finger movements such as finger tapping, sequential finger-thumb opposition, etc. These studies reported poorer tapping performance and reduced functional activation in SMA, M1, and cerebellum in schizophrenia[62]. Further evidence comes from the few studies on schizophrenia patients with catatonia, who usually present with behavioral motor inhibition. In a resting state perfusion MRI study, my group identified that catatonic schizophrenia is associated with specifically **increased cerebral blood flow (CBF)** in the supplementary motor area (**SMA**) and primary motor cortex (M1) in comparison to schizophrenia patients who had never experienced catatonia before. State CBF values were highest among those patients with severe motor inhibition. However, SMA perfusion was not different between state catatonia and controls[63]. The findings suggest a critical role of the SMA in movement initiation deficits in schizophrenia with state catatonia. However, from these data it was not possible to determine whether SMA hyperperfusion was the result of a motor network pathology that leads to inhibited motor output or whether SMA pathology drives this behavioral inhibition. Furthermore, finger tapping studies in catatonia patients indicated that M1 and SMA were less active during the task[64, 65]. Given the limitations of most previous studies focusing on one task or brain region, the next step must include **a network perspective to understand the pathobiology of motor inhibition as seen in PS**. A first cross-sectional study of my group applied resting state fMRI in order to test functional connectivity within the motor networks in schizophrenia. We found that schizophrenia was characterized by increased connectivity between key regions of the motor network, particularly between thalamus and motor/premotor cortex, M1 and cerebellum, cingulate seeds and STN[11]. Furthermore, the functional abnormalities between M1 and thalamus or cerebellum at rest were linked to observer ratings and instrumental measures of PS in schizophrenia. **Even though there is evidence suggesting that aberrant thalamocortical as well as cerebellar-cortical functional and structural connectivity may contribute to psychomotor slowing, the mechanism is still not entirely clear. This issue needs to be addressed at a network perspective and requires an exploration of the neural effects of interventions targeting psychomotor slowing.**

3.1.6 Intracortical excitability

Transcranial magnetic stimulation (TMS) allows for testing cortical neurophysiology in vivo. Converging evidence supports defective intracortical inhibition in M1 indexed by paired-pulse TMS in all stages of psychosis[66, 67]. Indeed, short-interval intracortical inhibition (SICI) is linked to GABAergic interneuron function. Reduced SICI values indicate **cortical disinhibition** in patients, which correlate with lower fractional anisotropy in motor tracts as well as lower processing speed[68]. Thus **psychomotor slowing** could be associated with **aberrant motor cortex excitability**, i.e. reduced SICI.

3.1.7 Brain stimulation for psychomotor slowing

Repetitive transcranial magnetic stimulation (rTMS) temporarily alters brain function in the targeted cortical areas. In addition, distant effects occur due to changes in network properties[69]. Depending on the frequency and type of stimulation, distinct effects on brain function are expected. Low-frequency rTMS (**lf-rTMS**) and continuous theta burst stimulation (cTBS) have **inhibitory** effects, while high-frequency rTMS (hf-rTMS) and intermittend theta burst (**iTBS**) have **facilitatory** effects. The preliminary knowledge of the pathobiology of PS suggests that rTMS could improve PS by changing aberrant motor network connectivity. Still, there are no published reports on the effects of non-invasive brain stimulation on PS. However, my group is conducting a randomized, double-blind, sham-controlled trial of rTMS for psychomotor slowing in major depressive disorder and schizophrenia (**NCT03275766**, clinicaltrials.gov).

Treatment is based on rTMS in 4 arms: 1) facilitatory hf-rTMS over the left dorsolateral prefrontal cortex (DLPFC), 2) inhibitory lf-rTMS over the supplementary motor area (SMA), 3) facilitatory iTBS over the SMA, and 4) sham stimulation with a placebo coil. The primary outcome parameter is the percentage of responders (> 30% reduction in the Salpêtrière Retardation Rating Scale (SRRS) from baseline). Across the whole group of 34 randomized subjects (22 with schizophrenia and 12 with depression), there is a group difference in the number of responders after 15 rTMS sessions applying the last-observation-carried-forward method (Chi^2=11.3, df=3, p=.007) with **78% responders under lf-rTMS over SMA and no responder under iTBS over SMA**. When we exclusively focus on schizophrenia, this effect is also detected (Chi^2=6.6, df=3). Thus, lf-rTMS over SMA ameliorates PS.

The neurophysiologic effects of rTMS treatment can be probed by perfusion MRI for local effects on metabolism, by fMRI on the network level and by TMS paradigms testing changes of cortical excitability of M1. Studies on inhibitory rTMS over the left SMA indicated short-term alterations of functional connectivity from SMA to M1 in healthy subjects along with changes of local metabolic activity in focal dystonia patients and controls[70-72]. While we don't know whether similar effects would occur in patients with altered baseline functional connectivity in the motor network, we may expect to see neural changes (functional connectivity and regional perfusion) following rTMS treatment. Furthermore, single **inhibitory rTMS** for 15 mins over the premotor cortex led to **increased motor cortical excitability** in schizophrenia evidenced by reduced resting motor thresholds and increased motor evoked potentials, supporting our clinical findings of an amelioration of PS[73].

3.1.8 State of research summary

Motor abnormalities are a core feature of psychotic disorders, particularly in the schizophrenia spectrum, indicating poorer outcome[10, 15, 74]. Motor abnormalities are linked to alterations within the cerebral motor networks[22]. One important domain is psychomotor slowing (PS) that impacts gross and fine motor behavior. PS causes distress and functional disability. Moreover, PS can be reliably assessed by instrumentation[15]. But the underlying neurobiology of PS is not well understood. Prior work from correlational studies reported PS to be linked to aberrant functional and structural connectivity between M1 and thalamus or cerebellum[11, 60]. Particularly, patients with severe PS display hyperconnectivity and hyperperfusion in the premotor and motor cortices. Currently, no treatments specifically target PS in schizophrenia. But preliminary data from an rTMS study of my group indicate that inhibitory rTMS over the SMA alleviates PS. In addition, one session of inhibitory rTMS over SMA altered motor cortex excitability in psychosis[73]. **Given, that the motor circuitry is associated with PS and that inhibitory rTMS over SMA may improve PS in schizophrenia, we expect that inhibitory rTMS alters the relevant network for PS in patients**. The combination of resting state fMRI, perfusion MRI, diffusion MRI and rTMS is particularly suited to explore the neural changes in the motor networks[72]. Therefore, we will conduct a prospective RCT of rTMS for PS with neuroimaging assessments at baseline and week 3, focusing on the motor network. Furthermore, we will explore the effects of rTMS on cortical excitability, clinical and functional outcome. This is the first project to enable causal inferences on the pathobiology of PS and further supporting the use of effective rTMS treatment in schizophrenia by **proof of principle**.

3.2 Investigational Product (treatment, device) and Indication

TMS device: MagPro 30 with theta burst option

Manufacturer: MagVenture, Inc. Atlanta GA, USA

Device is intended for a broad range of neuroscientific research in humans.

There is CE conformity according to the ISO Norm 13485:2003.

3.3 Preclinical Evidence

Low-frequency rTMS (1Hz) has been tested in many studies of human neurophysiology, typically as single session rTMS with transient behavioral effects. Lf-rTMS over the SMA alters functional connectivity in the motor system in healthy subjects[72]. Likewise, lf-rTMS over M1 demonstrated increased local neural activity with increasing stimulus intensity in healthy controls, in addition to distant changes in neural activity within M1 connections[75]. Finally, the cortical excitability is altered in healthy subjects following lf-rTMS of M1[76].

3.4 Clinical Evidence to Date

rTMS treatment for psychomotor slowing has only been tested in Parkinson's disease[77, 78] and in a combined group of patients with major depression and schizophrenia (Walther et al. unpublished data). In our own randomized double-blind placebo-controlled trial, 15 sessions of 1 Hz rTMS were effective in ameliorating psychomotor slowing (see also Background). The active comparator iTBS was not effective, but also well-tolerated. There were no exceptional side-effects beyond those regularly reported in studies of rTMS.

The intended protocols (1Hz rTMS and iTBS as active control) have been widely used in neuropsychiatric cohorts to treat various symptoms[2]. Low-frequency rTMS (1Hz) is effective in reducing the severity of auditory verbal hallucinations in schizophrenia spectrum disorders[79-86]. There have been numerous reports demonstrating safety and efficacy of this protocol. The active comparator iTBS has been tested in studies in major depressive disorder and is effective in reducing depression severity with minimal side effects[87, 88]; most commonly transient headaches[2]. rTMS treatment has been applied between 10 and 30 sessions, usually 5 per week. There are international guidelines on the use of rTMS for clinical purposes, including safety measures and side effect assessments[1, 2].

3.5 Dose Rationale / Medical Device: Rationale for the intended purpose in study (pre-market MD)

As outlined in sections 3.1.7., 3.1.8., 3.3., and 3.4. rTMS has been used to modify psychomotor slowing in Parkinson's disease, depression, and schizophrenia. In addition, rTMS over the premotor and motor cortex alters brain function. Our previous study demonstrated clinical effectiveness of 1 Hz stimulation over left SMA on psychomotor slowing in 15 sessions. There are no other treatment studies on motor function in schizophrenia. However, studies applying less than 10 stimulation sessions to treat hallucinations were less effective in schizophrenia spectrum disorders[82, 89]. In major depression, rTMS studies with similar protocols usually treat patients for 4-6 weeks (20-30 sessions)[87].

3.6 Explanation for choice of comparator (or placebo)

This study will have two comparators: one placebo stimulation and an active stimulation that produces the opposite effect of the investigated protocol, i.e. iTBS over SMA which will facilitate neural activity. Both comparators are necessary to demonstrate clinical utility (placebo) and the neurophysiological changes associated with the three treatments (placebo and iTBS). Only this approach will finally allow testing whether inhibitory lf-rTMS is changing the motor system in a specific direction that is causal for clinically relevant changes of motor behavior. The waiting list will disentangle specific stimulation effects from effects of time or expectation. Placebo will disentangle specific rTMS treatment effects. Finally, iTBS will disentangle the direction of neurophysiological changes of the motor system. All stimulations will target the left SMA. Mode of application, localization, frequency of sessions and apparatus will be identical for all protocol arms. Arms will differ in the coil used (real or sham) and in the frequency of the applied stimulation (1 Hz vs. iTBS). The number of pulses is similar in all protocols (1'000-1'200).

3.7 Risks / Benefits

The study intervention is adminstered as an add-on to existing treatment in inpatient (or less often dayclinic) settings. The study procedures pose some extra-effort and burden to the patients, particularly during baseline and week 3 assessments. However, the time and inconvenience is acceptable and comparable to those of other studies in the field. From our extensive experience with studies in these patient groups we know that assessments are acceptable and may be split to different days if needed. The neuroimaging with MRI twice has a total duration of less than 60 mins per session including the preparation. Most of the scanning time, participants will be in a resting state, i.e. have no task – just to relax lying in the scanner. This can be accomplished by many, even very ill subjects. Only a short proportion of the scanning time is devoted to a simple motor task in the scanner, when participants are asked to move their fingers. Taken together, both clinical and neuroimaging assessments are acceptable.

The study intervention (rTMS) has a minimal risk of increasing the severity of the disorder, i.e. deterioration. In our prior clinical rTMS trial with the same stimulation protocol and 15 rTMS sessions, there was no case of deterioration. Only one SAE occurred which was unrelated to the intervention. In addition, patients are under close observation by the study team and the hospital teams who see patients in the inpatient or dayclinic setting. Thus, changes are rapidly recognized and appropriate measures will be taken, as the study is only an add-on to standard treatment.

This study will follow the general recommendations for safety in rTMS protocols[1, 2] and the device manual. In addition, a screening standard questionnaire for rTMS candiates will be applied to ensure that all strict exclusion criteria for safety in the protocol will be respected. The expected main adverse effects could be transient headache, transient local pain, transient neck pain, transient toothache and

transient paraesthesia. Transient hearing changes have not been reported applying theta burst TMS but are likely possible. Therefore hearing protection will be used (earplugs). Furthermore we will make prompt referral for auditory assessment in case of any individual who complains of hearing loss, tinnitus or aural fullness following the rTMS. Seizure induction is rare but possible in epilepsy patients, which are therefore excluded. Furthermore, in some patients transient impairment of working memory was reported. Taken together, if the safety regulations are followed, the risk for participants is minimal. All adverse events are only temporarily occurring and no severe adverse events have been reported in rTMS trials up to now.

Magnetic fields attenuate rapidly with distance, so it seems unlikely that the fetus of a pregnant woman might be directly affected by rTMS. Currently, no side effects to the child were reported in reports of repeated rTMS in pregnant women[90, 91]. Still, pregnant women will be excluded from this study. Prior to study entry, a pregnancy test will be performed by all women in childbearing age, who are also asked to use a simple, effective contraceptive method during the 3 week trial.

The procedure of blood sampling involves the physician puncturing a vein in the groin or the bend of the elbow. Occasionally, a slight internal bleeding and the formation of a hematoma can occur, but usually disappears within a few days. Other risks such as nerve injury, thrombus formation and infection because of the punctuation, are very rare and with trained professionals almost excluded.

Participation in the study does not pose a particular risk for patients, as the rTMS will be added on to the existing standard medical and non-medical interventions. It is likely that patients in the experimental group will experience better outcomes. However, most patients (in control arms) and healthy controls will not have any personal benefit from participation in the study. Still, the study will be an important step towards the design of specific trials to improve psychomotor slowing in schizophrenia. Given, that the experimental arm was superior in ameliorating psychomotor slowing, treatment will result in better outcomes and quality of lives. In addition, results of this study will be used to plan further interventional trials in patients with schizophrenia, which may then introduce rTMS as a standard add-on treatment of psychomotor slowing in schizophrenia spectrum disorders.

3.8 Justification of choice of study population

This study will include a group of patients with schizophrenia spectrum disorders, who will receive the assessments and interventions. In addition, a group of healthy control subjects is included only for the assessments, but not to receive an intervention. Patients will be included if they can provide consent on their own (no minors and no patients incapable of understanding the study information will be recruited). Participation in the study does not interfere with regular treatment. Patients' interests will be safeguarded by the treating physicians who are not part of the study team.

Study of patients is necessary in order to test the rTMS effect in a group with clear psychomotor slowing. These symptoms are only found in patients with severe psychiatric disorders.

Study of controls is needed in order to compare motor behaviour abnormalities and alterations in brain network structure and function between health and disease. Controls will not receive any intervention.

4. STUDY OBJECTIVES

4.1 Overall Objective

This study investigates the neural correlates of psychomotor slowing in schizophrenia spectrum disorders and the neurophysiologic changes associated with successful application of rTMS to ameliorate slowing. To reach this aim, the study combines a randomized, double-blind, placebo-controlled trial of rTMS in patients over 3 weeks and a cross-sectional and longitudinal study focussing on motor behaviour and brain imaging. Therefore, this study has four main aims. First, we aim to **investigate the clinical and functional neural changes following 3 weeks of daily rTMS treatment** (inhibitory, facilitatory, or placebo). An exploratory aim is to describe imaging markers of treatment response. Second, we aim to **characterize the pathophysiology of psychomotor slowing** (PS) in depth combining multimodal neuroimaging and electrophysiology with instrumental and observer-based measures of PS. Third, we aim **to characterize short-term changes of PS** by observing a waiting list cohort that is later allocated to treatment. And fourth, we aim to **describe the clinical outcome** of the rTMS intervention at 6-month follow-up.

4.2 Primary Objective

The study seeks to determine the clinical and neural effects of 15 sessions of inhibitory lf-rTMS over SMA on psychomotor slowing in schizophrenia spectrum disorders compared to facilitatory iTBS, placebo or patients on a waiting list.

4.3 Secondary Objectives

The study further seeks to determine, whether lf-rTMS (inhibitory) will be superior over placebo or iTBS (facilitatory) in all clinical and motor behavioral measures, e.g. greater reduction in BFCRS scores from baseline, greater increase in activity levels, or finger taps. In addition, the study will test the effects of rTMS on the cerebral motor network connectivity and activity, as well as on cerebellar function and motor cortex excitability. Furthermore, the study will explore the neuroimaging markers of response to treatment. Moreover, the study will test the association between measures of PS and neuroimaging markers of the motor system, e.g. network connectivity, activity, and structure. Next, the study will investigate the temporal dynamics of PS by comparing a waiting list group to the placebo group. Finally, the study will test whether lf-rTMS treatment for 3 weeks will have lasting effects on PS at week 6 or 6-months follow-up.

4.4 Safety Objectives

The study will also assess the tolerability of 15 of sessions rTMS in terms of stimulation side effects and duration of stimulation effects.

5. STUDY OUTCOMES

5.1 Primary Outcome

The primary outcome variable is the SRRS score as a surrogate marker of psychomotor slowing. The primary endpoint will be the change SRRS ratings from baseline to week 3 measured with the SRRS[92]. The SRRS is administered at baseline, week 1, week 2, week 3, week 6, and week 24. It is an observer rated scale quantifying the severity of psychomotor slowing with 15 items, each ranging 0-4. The SRRS has been developed to target this behavior and has been used in previous trials. Assessments will be performed by blinded raters, who have been trained to use the scale reliably.

5.2 Secondary Outcomes

5.2.1 Clinical outcomes

Proportion of responders (>= 30% reduction from baseline SRRS) between groups at week 1, 2, 3, 6, and 24. This will provide an additional categorical measure of who benefits from the intervention in terms of the main target (psychomotor slowing).

Change in other commonly observed motor symptoms from baseline, including rating scales on abnormal involuntary movements, Parkinsonism, and catatonia at week 3, 6, and 24. See section 9 for details on instruments.

Change in general psychopathology using the positive and negative syndrome scale PANSS[93] and the brief negative symptom scale BNSS[94] between baseline and week 3, week 6, and week 24.

Change in self-reported physical activity and experienced negative symptoms using the International Physical Activity Questionnaire (IPAQ)[95] and the self-evaluation of negative symptoms (SNS)[96].

5.2.2 Behavioral outcomes

Change in objective gross motor activity as measured with 24 hours of wrist actigraphy at baseline, week 3, 6, and 24.

Change in fine motor function as measured with the coin rotation task at baseline, week 3, 6, and 24.

Grip strength at baseline, week 3, 6, and 24.

5.2.3 Physiological outcomes

Changes in motor cortex excitability from baseline to week 3, 6, and 24 using a TMS paradigm of short interval intracortical inhibition (SICI), intracortical facilitation (ICF) and 1 mV motor evoked potentials (MEP).

Changes in postural sway from baseline at week 3, using the Kistler platform and assessments with eyes open as well as eyes closed.

5.2.4 Neuroimaging outcomes

Change in resting state perfusion within the motor network from baseline to week 3.

Change in motor network resting state connectivity from baseline to week 3.

Change in neural activation patterns during a finger tapping task using functional MRI at baseline and week 3.

5.3 Other Outcomes of Interest

Change in social and community functioning using the global assessment of functioning GAF[97], the Social and Occupational Functioning SOFAS[98], and the UPSA-brief[99] short assessment of functional capacity at baseline, week 6, and week 24. Social and community functioning is a relevant distal outcome for any psychiatric intervention. Future research questions may arise later, particularly, further analyses of the collected blood samples are possible.

5.4 Safety Outcomes

After each rTMS session, participants are inquired about stimulation side effects. After sessions 5, 10 and 15, we will apply a short rating scale to assess side effects according to the guidelines[1, 2].

6. STUDY DESIGN

6.1 General study design and justification of design

The design of this single-site study is a 4 parallel-arm, double-blind, randomized, placebo-controlled trial. Patients will be randomized to one of four treatment arms. Patients will all receive TMS over the left SMA in 15 daily sessions over 3 weeks as add-on treament. In three groups, assessments will be conducted immediately before and after the three weeks treatment (1 Hz, iTBS, and Placebo), while the fourth group will receive active treatment (1Hz) only after a 3 week waiting period with no additional intervention. After 6 months we will perform a clinical follow-up assessment. The interventions will be conducted by a small team (2 persons) who will know the stimulation parameters. All assessments will be conducted by a different team, who is completely blind to treatment and specifically trained to use the instruments and rating scales. Patients are also blind to treatment, because stimulation site is the same for all groups and there will be no change of protocols during the study. The placebo group will be stimulated with a placebo coil that looks identical and emits identical noises compared to the real TMS coil, but without any magnetic impulse emission. Assessments with clinical rating scales will be complemented by objective and instrumental motor tests that are not prone to rater bias. Therefore, both the patients and the assessors are blind to treatment protocol.

The study will include patients with schizophrenia spectrum disorders who currently have psychomotor slowing according to the SRRS. The protocol arms are chosen to test the clinical efficacy and neurophysiological changes of 15 sessions 1 Hz rTMS for psychomotor slowing in schizophrenia spectrum disorders. The placebo-arm is required to demonstrate an effect of rTMS as add-on treatment. The active control is required to demonstrate an opposite effect at the neural level compared to active treatment. Finally, the waiting list is important to characterize potential self-limitation of the condition under study. Because these subjects are referred to a waiting list, they will receive active treatment

thereafter. This group will not be completely blinded, as they will know to be in the active treatment group from week 3 through week 6.

We intend to enrol 88 patients with schizophrenia spectrum disorders. For the neuroimaging and motor behavioural assessments, we also plan to include 40 matched healthy control subjects.

For each patient in group 1-3 the study is 3 weeks and a follow-up interview after 6 months. Patients in group 4 (waiting list) will have three assessments (baseline, week 3 and week 6) and a follow-up interview at 6 months. The total duration of the study is 4 years.

6.2 Methods of minimising bias

6.2.1 Randomisation

Using the free software research randomizer, we will generate for the patients a list with four numbers indicating the group allocation, i.e. stimulation types. Allocation will be 1:1:1:1 across the whole intended sample. Subsequent numbers of study inclusion will therefore determine to which study arm the patient will be randomized. The lists will only be available for the principal investigator and locked in his office. He will perform randomization and give the written group allocation to the investigators.

6.2.2 Blinding procedures

As described in 6.1, patients will receive 15 sessions of rTMS over the left SMA in a blinded fashion. They will not be able to see which protocol is used (coil placement on top of their head, machine display behind the patients). Because of the parallel arm design, they will not know how the other protocols feel. Patients will be eye-blinded and ear-plugged during rTMS.

The teams performing assessments and rTMS stimulations will be strictly distinct. Therefore, patients and outcome assessors will be blinded to treatment arm.

6.2.3 Other methods of minimising bias

Outcomes are assessed with instrumental means or validated questionnaires. All assessments will follow a standard routine. Assessors will be trained to use instruments by the PI.

6.3 Unblinding Procedures (Code break)

In case of severe adverse events, an unblinding can take place at the responsibility of the principal investigator. In this case, the participant will not be able to further participate in the study. The allocated intervention order will be kept in a sealed envelope. The sealed envelopes will be stored at a central place, i.e. the office of the lab, so they can be accessed in case of any emergency.

7. STUDY POPULATION

The aim is to test 88 subjects with schizophrenia spectrum disorders and 40 healthy control subjects. Both genders will be included, age range 18 – 65 years.

7.1 Eligibility criteria

Participants fulfilling all of the following **inclusion** criteria are eligible for the study:

- ages 18–60 years
- Right-handed subjects
- Ability and willingness to participate in the study
- Ability to provide written informed consent
- Informed Consent as documented by signature (Appendix Informed Consent Form)
- **Patients only**: schizophrenia spectrum disorders according to DSM-5 **with** psychomotor slowing (SRRS score ≥ 15)

The presence of any one of the following **exclusion** criteria will lead to exclusion of the participant:

- Substance abuse or dependence other than nicotine
- Past or current medical or neurological condition associated with impaired or aberrant movement, such as brain tumors, stroke, M. Parkinson, M. Huntington, dystonia, or severe head trauma with subsequent loss of conciousness.
- Epilepsy or other convulsions
- History of any hearing problems or ringing in the ears

- Standard exclusion criteria for MRI scanning and TMS; e.g. metal implants, claustrophobia
- Patients only: any TMS treatment in the past 3 months
- Women who are pregnant or breast feeding,
- Intention to become pregnant during the course of the study,
- Female participants who are surgically sterilised / hysterectomised or post-menopausal for longer than 2 years are not considered as being of child bearing potential.
- Previous enrolment into the current study,
- Enrolment of the investigator, his/her family members, employees and other dependent persons
- **Controls only**: history of any psychiatric disorder or first-degree relatives with schizophrenia spectrum disorders.

7.2 Recruitment and screening

Healthy participants will be recruited by word-of-mouth, an internet link at the homepage of the department and flyers at supermarkets or at the University of Bern. Staff of the University Hospital of Psychiatry Bern will not be recruited. Furthermore, patients will be asked for participation at the inpatient and outpatient departments of the University Hospital of Psychiatry, Bern. All participants will spend approximately a total of 18 hours in the study on 7 different assessment days (screening, baseline, 3x during interventions and 2 follow-ups). Thus, they will be compensated by a single payment of CHF 200,-. Screening is described in section 9.1 and performed by master-level psychologists or psychiatrists.

7.3 Assignment to study groups

Randomization procedure is described in section 6.2.1. When the investigators have recruited a patient-participant, the principal investigator will allocate the person to a study group based on the randomization list and the sequence of enrolment in the study. From the list of numbers, patients are sequentially allocated by the principal investigator to one of the four treatment arms. The principal investigator will provide written information to the investigators concerning the treatment arm. The lists with the group allocations are only accessible for the principal investigator.

7.4 Criteria for withdrawal / discontinuation of participants

Participants may discontinue the trial at any time or withdraw consent. Furthermore, the treating physicians may request study discontinuation in case of significant deterioration of the condition at any time during the intervention period. All data from subjects in the intent-to-treat population, who received at least one dose of rTMS will be further used in the analyses. We will apply the last-observation-carried-forward (LOCF) method in the final analyses. In case a patient withdraws consent before the first rTMS stimulation, we will recruit an additional patient. The number of all patients recruited and randomized will be reported in the publications. All data from neuroimaging acquisition and motor behaviour will be used whenever possible, e.g. for analyses of baseline associations between brain imaging and behavior. In case of withdrawal due to adverse events or serious adverse events there will be follow-up examinations after 14 days and repeatedly until the problem is resolved, see 9.2.5.

8. STUDY INTERVENTION

8.1 Identity of Investigational Products (treatment / medical device)

8.1.1 Experimental Intervention (treatment / medical device)

Name: MagPro R30 with theta burst option

Manufacturer: MagVentrue, Inc. Atlanta GA, USA

The device is CE certified according to ISO-Norm 13485:2003 and approved for clinical use.

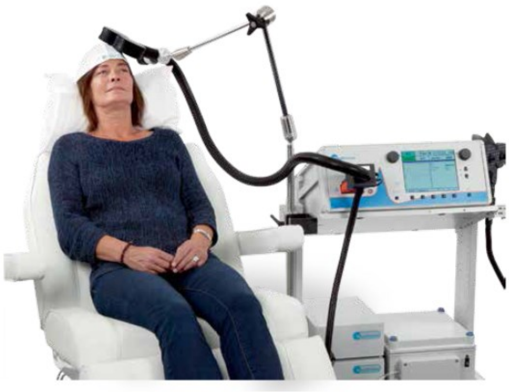

Active stimulation: 1 Hz stimulation of 17 mins over left SMA, 15 sessions in total (5 per week)

8.1.2 Control Intervention (standard/routine/comparator treatment / medical device)

All stimulations are delivered with the same device. The stimulation sites are the same (left SMA) for all groups. The iTBS protocol is shorter (3 mins), the placebo protocol will be delivered with a specific placebo-coil that looks identical, makes identical sounds but produces no magnetic pulses.

Placebo control: 1 Hz stimulation of 17 mins over left SMA without magnetic emission, 15 sessions in total (5 per week)

Active control: iTBS stimulation over left SMA, 21 mins per session, 15 sessions in total (5 per week)

Waiting list: no intervention during the first three weeks, afterwards active stimulation: 1 Hz stimulation of 17 mins over left SMA, 15 sessions in total (5 per week)

8.1.3 Packaging, Labelling and Supply (re-supply)

Not applicable.

8.1.4 Storage Conditions

TMS devices are stored in the institution (translational research center at the University Hospital of Psychiatry, Bern) according to internal regulations and the device manual.

8.2 Administration of experimental and control interventions

8.2.1 Experimental Intervention

The application of the TMS device follows the published guidelines[1, 2] and the device manual. Before each session, the resting motor threshold is determined to identify the individual intensity of stimulation. The position for the coil placement is left SMA, which is determined either via neuronavigation using individual brain anatomy or 3 cm anterior of the leg motor area, which is individually determined through single pulse stimulations causing leg movements.

Both experimenter and participants will wear earplugs for auditory safety.

Active experimental protocol:

Lf-rTMS at 1Hz will be used with 1’000 pulses at an intensity of 110% of the resting motor threshold

(approximate duration 17 minutes). The protocol is identical to that of our previous study and a study in Parkinson's disease[77]. 15 sessions in total (5 per week)

8.2.2 Control Intervention

Placebo control: 1 Hz stimulation of 17 mins over left SMA without magnetic emission, using the Placebo-coil. 15 sessions in total (5 per week)

Active control: Stimulation parameters for iTBS will follow those of Huang et al.[100], including 600 pulses at 50 Hz (stimulation in 2 sec trains every 10 sec for 190 sec in total). To increase the effect, we will deliver two iTBS series in one session separated by 15 min with a total of 1200 pulses. During the experiment, iTBS pulse intensity is adjusted to 80% of the motor threshold. iTBS stimulation of 21 mins over left SMA, 15 sessions in total (5 per week)

Waiting list: same protocol as active experimental protocol but only between week 3 and 6.

8.3 Dose / Device modifications

In case of intolerable side effects, the study will be discontinued for the participant. No dose or device modifications are planned.

8.4 Compliance with study intervention

Investigators have to document the order of stimulation. This will be checked for consistency with the group allocation. No further strategies are needed. Participants are always under observation during the rTMS sessions and the planned assessments. Non-compliance on the side of the participant would lead to study discontinuation for this person. Use of concomitant medication will be retrieved from the medical files of the patients and transferred to the CRF.

8.5 Data Collection and Follow-up for withdrawn participants

If participants withdraw their consent, they will be contacted immediately to clarify whether there were intolerance issues with the study. See section 9.2.5 for safety follow-up measures. In case of withdrawal the data will be analyzed and stored encrypted as all the data of the other participants. However, we will remove the encryption key for the participants who withdrew consent and therefore it will not be possible to identify the subjects from encrypted data. Patients who withdrew consent will be invited for the 6 month follow-up interview using their preferred way of contact (Email, letter).

8.6 Trial specific preventive measures

No specific preventive measures are needed.

8.7 Concomitant Interventions (treatments)

Current medication will be recorded in the CRF at study entry and throughout the 3 week intervention period. Patients are expected to comply with the treatment they are receiving from their treating physicians.

Because this is an add-on treatment, we expect relevant changes from concomitant interventions. Therefore, we apply placebo, waiting-list, and an active control group.

8.8 Study Drug / Medical Device Accountability

Not applicable.

8.9 Return or Destruction of Study Drug / Medical Device

The TMS devices are already in use in the clinic. No study specific procedures are intended.

9. STUDY ASSESSMENTS

9.1 Study flow chart(s) / table of study procedures and assessments

Flow charts are distinct for controls and patients, because controls receive no intervention. Furthermore, three patient groups will start interventions immediately (lf-rTMS, iTBS, and Placebo; i.e. groups 1-3), but one group will be waiting for 3 weeks and then enter the intervention phase (waiting group; i.e. group 4).

9.1.1 Patients of treatment groups 1-3

Study Periods	Screening	Treatment, Intervention Period				Follow-up	
Visit	1	2	3	4	5	6	7
Time (week)	-1	0	1	2	3	6	24
Patient Information and Informed Consent	x						
Demographics	x						
CASH and SCID (history)	x						
In- /Exclusion Criteria	x						
Physical Examination	x						
Pregnancy Test	x						
Psychopathology (TALD, NES)		x					
Randomisation		x					
Administer rTMS (5 sessions per week)		x	x	x			
Primary Variable SRRS		x	x	x	x	x	x
Motor scales UPDRS, BFCRS, AIMS, IPAQ		x			x	x	x
Psychopathology (PANSS, BNSS, SNS)		x			x	x	x
Actigraphy and coin rotation		x			x	x	x
Posturography		x			x		
TMS cortical excitability		x			x	x	x
Grip force measurement		X			X	X	X
Cerebral MRI		x			x		
Blood sampling		X			X		
Functional outcome (SOFAS, GAF, UPSA-brief)		x				x	x
Concomitant Therapy		x	x	x	x		
Adverse Events		x	x	x	x		

Study events patient groups 1-3

Visit	Information	Instrument used	Expected time
1 (Screening)	Check inclusion / exclusion criteria	CRF	5 min
	Demographic data	CRF	5 min
	Medical history	CRF	10 min
	Handedness	Edinburgh Handedness Inventory[101]	5 min
	Pregnancy test	Urine based test	10 min
	Diagnoses ascertainment	SCID interview	60 min
2 (baseline)	Psychopathology	Positive And Negative Syndrome Scale (PANSS)[93] interview, CASH[102] interview, Brief Negative Symptom Scale (BNSS)[94], Thought and Language Disorder	90 min

		(TALD)[103], Neurological Evaluation Scale (NES)[104]	
	Primary outcome	SRRS[92]	5 min
	Motor scales	Unified Parkinson's Disease Rating Scale (UPDRS)[105], Bush Francis Catatonia Rating Scale (BFCRS)[106], Abnormal Involuntary Movement Scale (AIMS)[107]	15 min
	Self Report	Self-Evaluation of negative symptoms (SNS)[96], International Physical Actitvity Questionnaire (IPAQ)[95]	10 min
	Motor tests	Actigraphy and coin rotation	5 min
	Posturography	Kistler platform at Dept. of Neurology	30 min
	Cortical excitability	TMS paradigm with paired pulse stimulation	30 min
	Grip force measurement	Measure of the strength in each hand	5 min
	Neuroimaging	MRI scan including structural T1, Arterial spin labelling, BOLD resting state and task fMRI	60 min
	Blood sampling	Blood sampling	10 min
	Functional outcome	Global Assessment of Functioning GAF, Social and Occupational Functioning Scale SOFAS, UPSA-brief assessment of functional capacity	15 min
	rTMS	5 rTMS daily sessions à 20 min	100 min
3 (week 1)	Primary outcome	SRRS	5 min
	rTMS	5 rTMS daily sessions à 20 min	100 min
	Check side effects	CRF	5 min
	Concomitant therapy	CRF	5 min
4 (week 2)	Primary outcome	SRRS	5 min
	rTMS	5 rTMS daily sessions à 20 min	100 min
	Check side effects	CRF	5 min
	Concomitant therapy	CRF	5 min
5 (week 3)	Psychopathology	Positive And Negative Syndrome Scale (PANSS)[93] interview, Brief Negative Symptom Scale (BNSS)[94]	30 min
	Primary outcome	SRRS[92]	5 min
	Motor scales	Unified Parkinson's Disease Rating Scale (UPDRS)[105], Bush Francis Catatonia Rating Scale (BFCRS)[106], Abnormal Involuntary Movement Scale (AIMS)[107]	15 min
	Self Report	Self-Evaluation of negative symptoms (SNS)[96], International Physical Actitvity Questionnaire (IPAQ)[95]	10 min
	Motor tests	Actigraphy and coin rotation	5 min
	Posturography	Kistler platform at Dept. of Neurology	30 min
	Cortical excitability	TMS paradigm with paired pulse stimulation	30 min
	Grip force measurement	Measure of the strength in each hand	5 min

	Neuroimaging	MRI scan including structural T1, Arterial spin labelling, BOLD resting state and task fMRI	60 min
	Blood sampling	Blood sampling	10 min
	Functional outcome	Global Assessment of Functioning GAF, Social and Occupational Functioning Scale SOFAS, UPSA-brief assessment of functional capacity	15 min
	Check side effects	CRF	5 min
	Concomitant therapy	CRF	5 min
6 (week 6)	Psychopathology	Positive And Negative Syndrome Scale (PANSS)[93] interview, Brief Negative Symptom Scale (BNSS)[94]	30 min
	Primary outcome	SRRS[92]	5 min
	Motor scales	Unified Parkinson's Disease Rating Scale (UPDRS)[105], Bush Francis Catatonia Rating Scale (BFCRS)[106], Abnormal Involuntary Movement Scale (AIMS)[107]	15 min
	Self Report	Self-Evaluation of negative symptoms (SNS)[96], International Physical Actitvity Questionnaire (IPAQ)[95]	10 min
	Motor tests	Actigraphy and coin rotation	5 min
	Cortical excitability	TMS paradigm with paired pulse stimulation	30 min
	Grip force measurement	Measure of the strength in each hand	5 min
	Functional outcome	Global Assessment of Functioning GAF, Social and Occupational Functioning Scale SOFAS, UPSA-brief assessment of functional capacity	15 min
7 (week 24)	Psychopathology	Positive And Negative Syndrome Scale (PANSS)[93] interview, Brief Negative Symptom Scale (BNSS)[94]	30 min
	Primary outcome	SRRS[92]	5 min
	Motor scales	Unified Parkinson's Disease Rating Scale (UPDRS)[105], Bush Francis Catatonia Rating Scale (BFCRS)[106], Abnormal Involuntary Movement Scale (AIMS)[107]	15 min
	Self Report	Self-Evaluation of negative symptoms (SNS)[96], International Physical Actitvity Questionnaire (IPAQ)[95]	10 min
	Motor tests	Actigraphy and coin rotation	5 min
	Cortical excitability	TMS paradigm with paired pulse stimulation	30 min
	Grip force measurement	Measure of the strength in each hand	5 min
	Functional outcome	Global Assessment of Functioning GAF, Social and Occupational Functioning Scale SOFAS, UPSA-brief assessment of functional capacity	15 min

9.1.2 Patients of treatment group 4 (waiting group)

Study Periods	Screening	Treatment, Intervention Period					Follow-up	
Visit	1	2	3	4	5	6	7	8
Time (week)	-1	0	3	4	5	6	9	24
Patient Information and Informed Consent	x							
Demographics	x							
CASH and SCID (history)	x							
In- /Exclusion Criteria	x							
Physical Examination	x							
Pregnancy Test	x							
Psychopathology (TALD, NES)		x						
Randomisation		x						
Administer rTMS (5 sessions per week)			x	x	x			
Primary Variable SRRS		x	x	x	x	x	x	x
Motor scales UPDRS, BFCRS, AIMS, IPAQ		x	x			x	x	x
Psychopathology (PANSS, BNSS, SNS)		x	x			x	x	x
Actigraphy and coin rotation		x	x			x	x	x
Posturography		x	x			x		
TMS cortical excitability		x	x			x	x	x
Grip force measurement		x	x			x	x	x
Cerebral MRI		x	x			x		
Blood sampling		x				x		
Functional outcome (SOFAS, GAF, UPSA-brief)		x	x				x	x
Concomitant Therapy		x	x	x	x	x		
Adverse Events			x	x	x	x		

Study events for patients of the waiting group

Visit	Information	Instrument used	Expected time
1 (Screening)	Check inclusion / exclusion criteria	CRF	5 min
	Demographic data	CRF	5 min
	Medical history	CRF	10 min
	Handedness	Edinburgh Handedness Inventory[101]	5 min
	Pregnancy test	Urine based test	10 min
	Diagnoses ascertainment	SCID interview	60 min
2 (baseline)	Psychopathology	Positive And Negative Syndrome Scale (PANSS)[93] interview, CASH[102] interview, Brief Negative Symptom Scale (BNSS)[94], Thought and Language Disorder (TALD)[103], Neurological Evaluation Scale (NES)[104]	90 min
	Primary outcome	SRRS[92]	5 min
	Motor scales	Unified Parkinson's Disease Rating Scale (UPDRS)[105], Bush Francis Catatonia Rating Scale (BFCRS)[106], Abnormal Involuntary Movement Scale (AIMS)[107]	15 min

	Self Report	Self-Evaluation of negative symptoms (SNS)[96], International Physical Actitvity Questionnaire (IPAQ)[95]	10 min
	Motor tests	Actigraphy and coin rotation	5 min
	Posturography	Kistler platform at Dept. of Neurology	30 min
	Cortical excitability	TMS paradigm with paired pulse stimulation	30 min
	Grip force measurement	Measure of the strength in each hand	5 min
	Neuroimaging	MRI scan including structural T1, Arterial spin labelling, BOLD resting state and task fMRI	60 min
	Blood sampling	Blood sampling	10 min
	Functional outcome	Global Assessment of Functioning GAF, Social and Occupational Functioning Scale SOFAS, UPSA-brief assessment of functional capacity	15 min
3 (week 3)	Psychopathology	Positive And Negative Syndrome Scale (PANSS)[93] interview, CASH[102] interview present part only, Brief Negative Symptom Scale (BNSS)[94], Thought and Language Disorder (TALD)[103], Neurological Evaluation Scale (NES)[104]	90 min
	Primary outcome	SRRS[92]	5 min
	Motor scales	Unified Parkinson's Disease Rating Scale (UPDRS)[105], Bush Francis Catatonia Rating Scale (BFCRS)[106], Abnormal Involuntary Movement Scale (AIMS)[107]	15 min
	Self Report	Self-Evaluation of negative symptoms (SNS)[96], International Physical Actitvity Questionnaire (IPAQ)[95]	10 min
	Motor tests	Actigraphy and coin rotation	5 min
	Posturography	Kistler platform at Dept. of Neurology	30 min
	Cortical excitability	TMS paradigm with paired pulse stimulation	30 min
	Grip force measurement	Measure of the strength in each hand	5 min
	Neuroimaging	MRI scan including structural T1, Arterial spin labelling, BOLD resting state and task fMRI	60 min
	Blood sampling	Blood sampling	10 min
	Functional outcome	Global Assessment of Functioning GAF, Social and Occupational Functioning Scale SOFAS, UPSA-brief assessment of functional capacity	15 min
	rTMS	5 rTMS daily sessions à 20 min	100 min
4 (week 4)	Primary outcome	SRRS	5 min
	rTMS	5 rTMS daily sessions à 20 min	100 min
	Check side effects	CRF	5 min
	Concomitant therapy	CRF	5 min

5 (week 5)	Primary outcome	SRRS	5 min
	rTMS	5 rTMS daily sessions à 20 min	100 min
	Check side effects	CRF	5 min
	Concomitant therapy	CRF	5 min
6 (week 6)	Psychopathology	Positive And Negative Syndrome Scale (PANSS)[93] interview, Brief Negative Symptom Scale (BNSS)[94]	30 min
	Primary outcome	SRRS[92]	5 min
	Motor scales	Unified Parkinson's Disease Rating Scale (UPDRS)[105], Bush Francis Catatonia Rating Scale (BFCRS)[106], Abnormal Involuntary Movement Scale (AIMS)[107]	15 min
	Self Report	Self-Evaluation of negative symptoms (SNS)[96], International Physical Actitvity Questionnaire (IPAQ)[95]	10 min
	Motor tests	Actigraphy and coin rotation	5 min
	Posturography	Kistler platform at Dept. of Neurology	30 min
	Cortical excitability	TMS paradigm with paired pulse stimulation	30 min
	Grip force measurement	Measure of the strength in each hand	5 min
	Neuroimaging	MRI scan including structural T1, Arterial spin labelling, BOLD resting state and task fMRI	60 min
	Blood sampling	Blood sampling	10 min
	Functional outcome	Global Assessment of Functioning GAF, Social and Occupational Functioning Scale SOFAS, UPSA-brief assessment of functional capacity	15 min
	Check side effects	CRF	5 min
	Concomitant therapy	CRF	5 min
7 (week 9)	Psychopathology	Positive And Negative Syndrome Scale (PANSS)[93] interview, Brief Negative Symptom Scale (BNSS)[94]	30 min
	Primary outcome	SRRS[92]	5 min
	Motor scales	Unified Parkinson's Disease Rating Scale (UPDRS)[105], Bush Francis Catatonia Rating Scale (BFCRS)[106], Abnormal Involuntary Movement Scale (AIMS)[107]	15 min
	Self Report	Self-Evaluation of negative symptoms (SNS)[96], International Physical Actitvity Questionnaire (IPAQ)[95]	10 min
	Motor tests	Actigraphy and coin rotation	5 min
	Cortical excitability	TMS paradigm with paired pulse stimulation	30 min
	Grip force measurement	Measure of the strength in each hand	5 min
	Functional outcome	Global Assessment of Functioning GAF, Social and Occupational Functioning Scale SOFAS, UPSA-brief assessment of	15 min

		functional capacity	
8 (week 24)	Psychopathology	Positive And Negative Syndrome Scale (PANSS)[93] interview, Brief Negative Symptom Scale (BNSS)[94]	30 min
	Primary outcome	SRRS[92]	5 min
	Motor scales	Unified Parkinson's Disease Rating Scale (UPDRS)[105], Bush Francis Catatonia Rating Scale (BFCRS)[106], Abnormal Involuntary Movement Scale (AIMS)[107]	15 min
	Self Report	Self-Evaluation of negative symptoms (SNS)[96], International Physical Actitvity Questionnaire (IPAQ)[95]	10 min
	Motor tests	Actigraphy and coin rotation	5 min
	Cortical excitability	TMS paradigm with paired pulse stimulation	30 min
	Grip force measurement	Measure of the strength in each hand	5 min
	Functional outcome	Global Assessment of Functioning GAF, Social and Occupational Functioning Scale SOFAS, UPSA-brief assessment of functional capacity	15 min

9.1.3 Control subjects

Study Periods	Screening	Observation period	
Visit<br>Time (week)	1<br>-1	2<br>0	3<br>3
Proband Information and Informed Consent	x		
Demographics	x		
SCID	x		
In- /Exclusion Criteria	x		
Physical Examination	x		
Pregnancy Test	x		
Primary Variable SRRS		x	x
Motor scales UPDRS, BFCRS, AIMS, IPAQ		x	x
Actigraphy and coin rotation		x	x
Posturography		x	x
TMS cortical excitability		x	x
Grip force measurement		x	x
Cerebral MRI		x	x
Blood sampling		x	x

Study events for controls

**Visit**	**Information**	**Instrument used**	**Expected time**
1 (Screening)	Check inclusion / exclusion criteria	CRF	5 min

	Demographic data	CRF	5 min
	Medical history	CRF	10 min
	Handedness	Edinburgh Handedness Inventory[101]	5 min
	Pregnancy test	Urine based test	10 min
	Diagnoses ascertainment	SCID interview	60 min
2 (week 0)	Primary outcome	SRRS[92]	5 min
	Motor scales	Unified Parkinson's Disease Rating Scale (UPDRS)[105], Bush Francis Catatonia Rating Scale (BFCRS)[106], Abnormal Involuntary Movement Scale (AIMS)[107]	15 min
	Self Report	International Physical Actitvity Questionnaire (IPAQ)[95]	5 min
	Motor tests	Actigraphy and coin rotation	5 min
	Posturography	Kistler platform at Dept. of Neurology	30 min
	Cortical excitability	TMS paradigm with paired pulse stimulation	30 min
	Grip force measurement	Measure of the strength in each hand	5 min
	Neuroimaging	MRI scan including structural T1, Arterial spin labelling, BOLD resting state and task fMRI	60 min
	Blood sampling	Blood sampling	10 min
3 (week 3)	Primary outcome	SRRS[92]	5 min
	Motor scales	Unified Parkinson's Disease Rating Scale (UPDRS)[105], Bush Francis Catatonia Rating Scale (BFCRS)[106], Abnormal Involuntary Movement Scale (AIMS)[107]	15 min
	Self Report	International Physical Actitvity Questionnaire (IPAQ)[95]	5 min
	Motor tests	Actigraphy and coin rotation	5 min
	Posturography	Kistler platform at Dept. of Neurology	30 min
	Cortical excitability	TMS paradigm with paired pulse stimulation	30 min
	Grip force measurement	Measure of the strength in each hand	5 min
	Neuroimaging	MRI scan including structural T1, Arterial spin labelling, BOLD resting state and task fMRI	60 min
	Blood sampling	Blood sampling	10 min

9.2 Assessments of outcomes

Assessors of outcome variables will be psychologists or psychiatrists of master-degree-level. All assessors will be trained by the PI to use the instruments correctly and to assure interrater reliability. Weekly staff meetings will ensure conformity in procedures. All assessments will be conducted blind to rTMS treatment.

9.2.1 Assessment of primary outcome

The Salpêtrière Retardation Rating Scale (SRRS) is applied at each visit from baseline to follow-up. The scale is described in section 5.1. Raters will be trained to use the scale and blind to rTMS treatment. The score of each of the 15 items is recorded in the CRF.

9.2.2 Assessment of secondary outcomes

9.2.2.1 Clinical outcomes

The change in motor syndromes from baseline is assessed at several visits (see 9.1). Trained raters blind to treatment will assess motor behaviour in standardized examinations with clinical rating scales. Parkinsonism is assess with the Unified Parkinson's Disease Rating Scale (UPDRS)[105], of which we will only assess part III, the current motor behaviour. Catatonia will be assessed with the Bush Francis Catatonia Rating Scale (BFCRS)[106], an 24 item rating scale specifically designed for this purpose. The timeframe of observation is the last week. Finally, dyskinesia will be monitored with the standard rating scale for this issue, the Abnormal Involuntary Movement Scale (AIMS)[107], a 7 item scale following a standardized examination. All motor rating scales are the gold standard of their kind.

The change of general schizophrenia symptoms is assessed with the Positive And Negative Syndrome Scale (PANSS)[93]. The 30 item scale is the widely used standard assessment following a standardized clinical interview. Change in negative symptoms is specifically assessed with the Brief Negative Symptom Scale (BNSS)[94], which allows monitoring relevant dimensions of negative symptoms such as apathy and diminished expression. Furthermore, we will apply the self-evaluation of negative symptoms (SNS)[96], a valid and reliable 20 items questionnaire to capture the subjective experience of negative symptoms.

9.2.2.2 Behavioral outcomes

Objective gross motor behaviour will be assessed using continuous wrist actigraphy for 24 hours. The actigraphs (empathica e4) will be worn on the wrist of the non-dominant arm. Data is stored as logged electronic file. This measure is sensitive to altered motor behaviour and has been successfully applied in many studies of Prof. Walther's team[27, 35, 49, 50, 108-111]. Patients will fill a sleep activity protocol to enable separation of sleep from wake periods during recording. This also allows to check data for consistency and plausibility, because measurements are continuously performed also in periods when participants are not observed.

The fine motor performance is assessed with the coin rotation task. In this task, participants are asked to rotate a .50 CHF coin between thumb, index and middle finger as fast as possible for a total of 30 seconds. The performance is recorded on video and later analysed offline. Analysis includes the number of half turns and the number of coin drops according to a validated formula[51, 52, 112].

Self-ratings of physical activity will be conducted with the 7-item International Physical Activity Questionnaire (IPAQ)[95], which has a German Version and has great psychometric properties. The ratings cover the past 7 days and allow calculating the energy expenditure and total activity.

9.2.2.3 Physiological outcomes

Measures of cortical excitability will be assessed as one of the important physiological outcome parameters. Measurements will be conducted with a MagPro R30 (MagVentrue, Inc. Atlanta GA, USA). Single pulse and paired pulse TMS protocols will be conducted to measure short-interval intracortical inhibition (SICI) at 1 msec interstimulus interval (ISI) and 3msec ISI, intracortical facilitation (ICF) at 7 msec ISI and 15 ms ISI, resting motor threshold (RMT), and 1 mV motor evoked potential (MEP), according to standard protocols[67, 113]

Posturography will be conducted at the Department of Neurology (Prof. Roger Kalla). The assessments of postural sway will be scheduled immediately before or after the MRI acquisition, because this is also located at the Inselspital Bern. Mean postural sway will be calculated as outcome variable for postural stability[53, 114]. The Kistler platform will be used to calculate of the pressure dependent fluctuation (x-, y-, z- axis) of the bodies' centre of gravity[115]. Participants will be measured standing with eyes open and eyes closed according to standard procedures.

9.2.2.4 Neuroimaging outcomes

Neuroimaging will be acquired twice, at baseline and week 3. MRI acquisition will be performed at a 3T Siemens Magnetom Trio scanner at the Institute of Diagnostic and Interventional Neuroradiology, Bern (local collaborator Prof. Dr. med. Roland Wiest). The 64-channel head coil will be used for all MRI images. First, high-resolution T1-weighted MR images will be obtained using a 3D magnetization-prepared rapid two-gradient-echo with 2 inversion times (MP2RAGE) sequence. fMRI will be performed using a multi-slice multi-band T2*-weighted echo planar imaging (EPI) sequence for resting state and during task execution. Prior to the fMRI images a B0 will be acquired for corrections of putative field

inhomogeneity. Afterwards, we will perform acquisition of cerebral blood flow (CBF) at rest using a pseudo-continuous arterial spin labelling (pCASL) sequence. Moreover, we will acquire a M0 image (M0 equilibrium magnetization of water signal) for the quantification of CBF and a B0 for corrections of putative field inhomogeneity. Finally, a set of diffusion-weighted images (DWI) that will allow reconstructing fiber tracts using the model based on diffusion tensor imaging (DTI).

Anatomy (MP2RAGE) The optimized acquisition parameters were as follows: 176 sagittal slices, 256 × 224 matrix (with a non-cubic field of view (FOV) of 256 × 224 mm2, yielding a nominal isotropic resolution of 1 mm3), 5000 ms repetition time (TR), 2.98 ms echo time (TE), 700 ms and 2500 ms inversion time (TI), flip angle 4° and 5°, GRAPPA acceleration factor 3 and a 8:22 min total acquisition time.

B0-map - The 48 EPI interleaved axial oblique slices will be positioned exactly like the fMRI slices with exactly the same slice geometry. Two amplitude and a phase image will be recorded in each subject (TR = 520 ms, TE1 = 4.92 ms, TE2 = 7.38 ms). The acquisition time will last 1 min 40 sec.

fMRI - The 48 EPI interleaved axial oblique slices will be positioned in-line with the bi-commissural axis with the following parameters: TR = 500 ms, TE = 30 ms, FA = 90, slice thickness = 3.6 mm, gap thickness = 0 mm, matrix size = 64 x 64 mm, FOV 230 x 230 mm2 resulting in a iso-voxel dimension of 3.6 mm x 3.6 mm x 3.6 mm. The sequence is driven in a 3D PACE mode (Siemens Erlangen) to enable prospective motion correction. With these sequence parameters we will cover the whole brain including the cerebellum. In total, first 720 dynamic scans will be collected for resting state fMRI (total of 6 mins) and subsequently 1200 dynamic scans will be collected during the conduction of the task (total of 10 mins). The task will consist of four blocks including two active paced blocks, a passive listening block and rest. All blocks except rest will be paced with a continuous 2Hz auditory cue. The two active blocks are **right hand finger tapping** (index finger vs. thumb) and **sequential finger-thumb opposition** (all fingers of the right hand vs. thumb). These tasks have produced reliable activation of SMA, M1, basal ganglia, and cerebellum in healthy subjects and are easy enough to be performed by schizophrenia patients with PS[62, 64, 65, 116, 117]. During one run, we will alternate active and passive blocks, i.e. finger tapping – listening – sequential finger opposition – rest. Each block will last for 15 s and each run takes 1 min. We will alternate the active blocks between runs. A total of 10 runs will be conducted. Instructions will be presented on a video goggle system. Cue tones will be delivered via headphones. Participants will be instructed and trained on the task outside the scanner.

pCASL - The 30 EPI interleaved axial oblique slices will be positioned in-line with the bi-commissural axis and acquired in sequential order with the following parameters: TR = 3000 ms, TE = 12 ms, FA = 90, slice thickness = 8.0 mm, gap thickness = 0 mm, matrix size = 64 x 64 mm, FOV 256 x 256 mm2 resulting in a voxel dimension of 4.0 mm x 4.0 mm x 8.0 mm. the sequence will additionally have the following parameters: bolus duration = 700 ms, inversion time = 2200 ms. In total 100 images will be acquired lasting in total 6 min.

9.2.3 Assessment of other outcomes of interest

Measures of social and community functioning as described in section 5.3.

9.2.4 Assessment of safety outcomes

9.2.4.1 Adverse events

After every rTMS session, participants are asked about new occurrence of adverse events. As example, they are inquired about sensations associated with the stimulation or headaches. The answers are recorded in the CRF. Furthermore, at each study visit during the intervention phase, i.e. after 5 rTMS sessions, patients will be inquired about adverse events using a standard questionnaire in the CRF[2].

In case the adverse event is not limited to stimulation, the participants will be asked at the next session (24 hours later), whether the adverse event still continues or when it was resolved. The frequency and type of adverse events will be reported in the publication of results.

9.2.4.2 Laboratory parameters

Blood sampling will be taken in a 4.9ml S-Monovette and will be send to and stored at the Center for Psychiatric Neuroscience, Department of Psychiatry, Lausanne University Hospital, Lausanne, Switzerland (Responsible analyzer Prof. Ph.D. Kim Do, Director of the Center of Psychiatric Neuroscience, phone: 021 643 69 49) for potential future analysis

9.2.4.3 Vital signs

No routine measurement of vital signs is planned. If patients report dizziness, clinical routines will include measurement of blood pressure and heart rate.

9.2.5 Assessments in participants who prematurely stop the study

Participants who prematurely stop the study will be immediately assessed for adverse events. In case of adverse events reports, a physical examination will be conducted and results will be recorded accordingly. Follow-up examinations will be planned within the next 14 days in case of continuing problem related to adverse events, and these examinations will be continued every 14 days until the health issue is resolved.

9.3 Procedures at each visit

9.3.1 Screening visit

- Check the inclusion and exclusion criteria, use CRF
- Collect demographic data, use CRF
- Conduct urine pregnancy test in female participants between 18 and 50 years of age
- Assess diagnoses with SCID, document results in CRF
- Assess handedness with EHI[101], document result in CRF
- Assess medical history in CRF

See table study events at 9.1

9.3.2 Visit 2 (baseline, week 0)

- Assess current psychopathology, use PANSS interview and BNSS, document in CRF
- Assess formal thought disorder, use TALD interview, document in CRF
- Assess Neurological soft signs, use NES, document in CRF
- Assess psychiatric history with CASH interview in patients, document result in CRF
- Assess primary outcome with SRRS scale, document in CRF
- Assess motor behaviour with rating scales, use BFCRS, UPDRS, AIMS, document results in CRF
- Assess functioning, use GAF, SOFAS, and UPSA-brief, document results in CRF
- Hand out self-rating questionnaires SNS and IPAQ, collect and document in CRF
- Conduct coin rotation test, record on video
- Record gross motor activity using actigraphy
- Test cortical excitability using the TMS device according to standard procedure. Record electronic data
- Grip force measurement , document result in CRF
- Blood sampling
- Acquire MRI scans at University Institute of Diagnostic and Interventional Neuroradiology
- Acquire posturography at University Hospital, Dept. of Neurology, record electronic data
- Record current medication regime in CRF
- Schedule 5 rTMS sessions
- Schedule next visit

9.3.3 Visit 3 and 4 (week 1 and 2)

- Assess primary outcome with SRRS scale, document in CRF
- Assess side effects with questions in the CRF
- Record current medication regime in CRF
- Schedule 5 rTMS sessions
- Schedule next visit

9.3.4 Visit 5 (end of intervention, week 3)

- Assess side effects with questions in the CRF
- Assess current psychopathology, use PANSS interview and BNSS, document in CRF
- Assess primary outcome with SRRS scale, document in CRF
- Assess motor behaviour with rating scales, use BFCRS, UPDRS, AIMS, document results in CRF
- Assess functioning, use GAF, SOFAS, and UPSA-brief, document results in CRF
- Hand out self-rating questionnaires SNS and IPAQ, collect and document in CRF
- Conduct coin rotation test, record on video
- Record gross motor activity using actigraphy

- Test cortical excitability using the TMS device according to standard procedure. Record electronic data
- Grip force measurement , document result in CRF
- Blood sampling
- Acquire MRI scans at University Institute of Diagnostic and Interventional Neuroradiology
- Acquire posturography at University Hospital, Dept. of Neurology, record electronic data
- Record current medication regime in CRF
- Schedule next visit (follow-up)

9.3.5 Visit 6 and 7 (follow-up, week 6 and 24)

- Assess current psychopathology, use PANSS interview and BNSS, document in CRF
- Assess primary outcome with SRRS scale, document in CRF
- Assess motor behaviour with rating scales, use BFCRS, UPDRS, AIMS, document results in CRF
- Assess functioning, use GAF, SOFAS, and UPSA-brief, document results in CRF
- Hand out self-rating questionnaires SNS and IPAQ, collect and document in CRF
- Conduct coin rotation test, record on video
- Record gross motor activity using actigraphy
- Test cortical excitability using the TMS device according to standard procedure. Record electronic data
- Grip force measurement , document result in CRF
- Record current medication regime in CRF
- Schedule next visit (follow-up)

10. SAFETY

10.1 Medical Device Category A studies

Device deficiencies and all **adverse events (AE)** including all **serious adverse events (SAE)** are collected, fully investigated and documented in the source document and appropriate case report form (CRF) during the entire study period, i.e. from patient’s informed consent until the last protocol-specific procedure, including a safety follow-up period. Documentation includes dates of event, treatment, resolution, assessment of seriousness and causal relationship to device and/or study procedure [ISO 14155, 6.4.1.].

Safety is assessed spontaneously at each stimulation session and in a structured set of questions at each visit during the intervention phase (after 5 stimulations). See 9.2.4. If adverse events occur, patients are followed-up with extra-visits after 14 days until the issue has resolved.

Typical adverse events to be expected by rTMS are:

- Mild pain or nausea (39 %)
- Mild headaches (28%)
- Mild neck pain (40%)

Rare events include hearing problems or local skin irritation.

10.1.1 Definition and Assessment of safety related events

Adverse Event (AE)

Any untoward medical occurrence, unintended disease or injury or any untoward clinical signs (including an abnormal laboratory finding) in participants, users or other persons whether or not related to the investigational medical device [ISO 14155: 3.2].

This includes events related to the investigational device or the comparator and to the procedures involved. For users or other persons this is restricted to events related to the investigational medical device.

Adverse Device Effect (ADE)

Adverse event related to the use of an investigational medical device [ISO 14155: 3.1].

This includes any adverse event resulting from insufficient or inadequate instructions for use, deployment, implantation, installation, operation, or any malfunction of the investigational medical device. This includes any

event that is a result of a use error or intentional misuse.

Serious Adverse Event (SAE) [European regulation on medical devices 2017/745, art. 58].

Any adverse event that led to any of the following:

(a) death,
(b) serious deterioration in the health of the subject that resulted in any of the following:
(i) life-threatening illness or injury,
(ii) permanent impairment of a body structure or a body function,
(iii) hospitalisation or prolongation of patient hospitalisation,
(iv) medical or surgical intervention to prevent life-threatening illness or injury or permanent impairment to a body structure or a body function,
(v) chronic disease,
(c) foetal distress, foetal death or a congenital physical or mental impairment or birth defect.

This includes device deficiencies that might have led to a serious adverse event if a) suitable action had not been taken or b) intervention had not been made or c) if circumstances had been less fortunate. These are submitted to the EC via BASEC within 7 days. A planned hospitalisation for pre-existing condition, or a procedure required by the protocol, without a serious deterioration in health, is not considered to be a serious adverse event.

Device deficiency

Inadequacy of a medical device related to its identity, quality, durability, reliability, safety or performance, such as malfunction, misuse or use error and inadequate labelling [ISO 14155: 3.15].

Health hazards that require measures

Findings in the trial that may affect the safety of study participants and, which require preventive or corrective measures intended to protect the health and safety of study participants SAE [ClinO Art. 37].

Causal Relationship of SAE [MEDDEV 2.7/3 revision 3, May 2015].

A causal relationship towards the medical device or study procedure should be rated as follows:

- **Not related:** The relationship to the device or procedures can be excluded.
- **Unlikely:** The relationship with the use of the device seems not relevant and/or the event can be reasonably explained by another cause, but additional information may be obtained.
- **Possible:** The relationship with the use of the investigational device is weak but cannot be ruled out completely. Alternative causes are also possible.
- **Probable:** The relationship with the use of the investigational device seems relevant and/or the event cannot reasonably explained by another cause.
- **Causal relationship:** The serious event is associated with the investigational device or with procedures beyond reasonable doubt.

Device deficiencies that might have led to an SAE are always related to the medical device.

10.1.2 Reporting of (Serious) Adverse Events and other safety related events

Reporting to Sponsor-Investigator:

The following events are to be reported to the Sponsor-Investigator within 24 hours upon becoming aware of the event:

- All SAEs
- Health hazards that require measures
- Device deficiencies

The Sponsor-Investigator will evaluate SAEs with regard to causality and seriousness. Device deficiencies are assessed regarding their potential to lead to an SAE.

Pregnancies

Because pregnancy tests are a prerequisite to participation and the intervention is only 3 weeks, pregnancies will not be further assessed or reported.

Reporting to Authorities [ClinO Art. 42]:

In Category A studies, the sponsor is subject to the notification requirements specified in Art. 15 of the MedDO of 17 October 2011 (SR 812.213).

It is the Investigator's responsibility to report to the Ethics Committee via BASEC **device deficiencies** that could have led to serious adverse events if suitable action had not been taken, intervention had not been made, or circumstances had been less fortunate within 7 days [ClinO Art. 42].

Health hazards that require measures are reported to the Ethics Committee via BASEC within 2 days [ClinO Art. 37].

Periodic safety reporting:

A yearly safety update-report is submitted by the Investigator to the Ethics Committee via BASEC.

10.1.3 Follow up of (Serious) Adverse Events

If adverse events occur, patients are followed-up with extra-visits after 14 days until the issue has resolved. This applies to patients who continue with the study but also to patients who prematurely exit the study. In case subjects are lost to followed-up, study personnel will contact the treating psychiatrists (or if unavailable, the general practitioners) in order to inform on serious adverse events and the recommendations for follow-up clinical examinations. In cases of headaches for example, care would include the prescription of a non-steroidal antirheumatic drug, i.e. pain medicine, according to the person's preferences, medical history, or interaction with existing medication.

11. STATISTICAL METHODS

A two-tailed p-value of < .05 is considered to be statistically significant in all analyses. We will also report effect sizes for the comparisons of primary and clinical secondary outcome variables between groups.

11.1 Hypotheses

Aim 1: investigate the clinical and functional neural changes following 15 sessions of daily rTMS

Hypothesis 1a (main hypothesis): lf-rTMS (inhibitory) will be superior over placebo or iTBS (facilitatory) in all clinical and motor behavioral measures, e.g. greater reduction in SRRS scores from baseline, greater increase in activity levels, or coin rotations.

Hypothesis 1b: lf-rTMS will reduce aberrant functional connectivity in the motor system, e.g. between thalamus and M1. lf-rTMS will alter regional CBF in the motor system and increase SMA and M1 activity during the fMRI task. No relevant changes are expected in the sham group, iTBS may deteriorate neural alterations.

Hypothesis 1c: cortical excitability will be differentially changed with rTMS. Lf-rTMS will increase SICI, while iTBS will reduce SICI.

Hypothesis 1d: at Visit 5 (end of intervention, week 3) there will be changes in blood markers of neural integrity and neuroinflammation with treatment (exploratory).

Aim 2: characterization of psychomotor slowing (PS) in schizophrenia spectrum disorders compared to healthy controls

Hypothesis 2a: Patients will have poorer performance on all motor tasks, eg. reduced activity levels, increased postural sway, and less coin rotations

Hypothesis 2b: Patients will have aberrant structural and functional connectivity within the motor system, as well as increased CBF in basal ganglia and decreased CBF in premotor/motor cortex. Applying network metrics, the functional motor network will be less efficient in schizophrenia.

Hypothesis 2c: Behavioral measures of PS will be associated with aberrant motor network structure, perfusion, function, and connectivity. For example, patients with strong PS will have increased resting state functional connectivity between thalamus and M1. PS severity will be linked to reduced structural motor network efficiency, lower M1 and SMA activity during the fMRI task, and increased SMA perfusion at rest.

Hypothesis 2d: Patients will have increased motor cortex excitability (e.g. reduced SICI), which will be linked to measures of PS.

Aim 3 characterize short term dynamics of PS

Hypothesis 3a: in very few subjects we expect relevant spontaneous improvements in PS measures. The majority will have less than 20% fluctuation of motor parameters within 3 weeks

Hypothesis 3b: we expect slight longitudinal changes in resting state functional connectivity and perfusion of the motor system, but no structural changes.

Aim 4: to describe the short-term and medium-term clinical outcome of 3 weeks of rTMS intervention

Hypothesis 4a: patients receiving inhibitory rTMS will have better outcome (less PS, less general symptom severity) shortly after the intervention (at 6 week follow-up) compared to patients receiving placebo or facilitatory iTBS

Hypothesis 4b: patients receiving inhibitory rTMS will have better outcome (less PS, less general symptom severity) and superior function (social and global) at 6-month follow-up compared to patients receiving placebo or facilitatory iTBS

Hypothesis 4c (exploratory): changes in functional connectivity within the motor system from baseline to week 3 will predict better outcome at week 6, particularly reduced M1-thalamus functional connectivity.

11.2 Determination of Sample Size

The main effects of the 3-week interventions on PS (SRRS scores) will be calculated in a repeated measures design including 2 measures (Baseline, week 3) and four groups (lf-rTMS, iTBS, sham, waiting group-lf-rTMS). Assuming a moderate effect size (f = 0.23) as indicated by our pilot data in a repeated measures ANOVA with moderate correlation between time points (0.5), a power of 0.95 and an alpha = 0.05, we would need 88 patients (22 per group).

11.3 Statistical criteria of termination of trial

No statistical stopping rules are planned.

11.4 Planned Analyses

11.4.1 Datasets to be analysed, analysis populations

All data from subjects in the intent-to-treat population, who received at least one dose of rTMS will be used in the analyses of rTMS effects. We will conduct sensitivity analyses in all trial-completers (see 11.5).

In the analyses on biological and clinical correlates of PS, we will use all data of the baseline assessment, thus including data from subjects who dropped-out prior to the first rTMS session.

11.4.2 Primary Analysis

The primary analysis will be a repeated measures ANOVA of SRRS scores with two timepoints (within subjects: baseline and week 3) and four groups (lf-rTMS, iTBS, sham, waiting group-lf-rTMS). In case of significant baseline demographic or clinical group differences, we will adjust for these in an repeated measures ANCOVA of SRRS.

The analysis will be performed by the PI and his team in SPSS using data from the RedCap database.

11.4.3 Secondary Analyses

Secondary analyses focus on the secondary outcomes (see 5.2) and hypotheses (see 11.1).

Crosstabs are used to calculate the proportion of responders (>=30% reduction in SRRS from baseline) across groups. Repeated measures ANOVAs will clarify the time-course of SRRS change from baseline to week 6 (including week 1, 2, 3 and 6) and differences between groups.

Repeated measures ANOVAs will test the change of clinical and motor rating scales between baseline and week 3, week 6, and week 24 and differences between groups.

Linear regression analyses will test the association between neuroimaging markers (resting state perfusion, connectivity, fMRI activation, ect.) and measures of PS at baseline.

Repeated measures ANOVAs will test the longitudinal changes in neuroimaging markers from baseline to week 3.

We will explore changes in blood markers of neural integrity and neuroinflammation from baseline to

week 3.

All analyses will be performed by the PI and his team or collaborators with permission of the PI.

11.4.4 Interim analyses

Interim analyses of the primary analysis are planned after enrolment of 10, 20, 30, 40, 50, 60, and 70 subjects. The scope is to estimate whether any adjustments of the treatment arms are necessary. In addition, baseline data can be tested for associations between neuroimaging data and clinical/motor measures after enrolment of 20, 40, or 60 subjects.

All interim analyses are optional and at the discretion of the PI. Interim analyses are conducted by the PI and his team.

11.4.5 Safety analysis

The study team will provide a descriptive analysis of all reported adverse events. The analysis will focus on the type of event, duration, and timing and relate the number of observed events to the number of rTMS sessions administered. Furthermore, the frequency of adverse events will be compared between treatment arms of the trial.

11.4.6 Deviation(s) from the original statistical plan

Any deviation from the statistical plan will be reported and justified in the methods of the study report.

11.5 Handling of missing data and drop-outs

We will apply the last-observation-carried-forward (LOCF) method in the final analyses. In case a patient withdraws consent before the first rTMS stimulation, we will recruit an additional patient. The number of all patients recruited and randomized will be reported in the publications.

Sensitivity analyses will be performed in order to establish whether premature dropout would influence the results. Therefore, the primary analyses will be repeated with all subjects who completed the interventional trial. Further tests will establish whether drop-outs differ from completers in basic clinical or demographic variables.

12. QUALITY ASSURANCE AND CONTROL

All study personnel will be trained by the PI to achieve consistent adherence to procedures and interrater reliability. Standard operating procedures will be written for the intervention and standardized TMS measurements. For all clinical rating scales, instructions and manuals are provided for the study personnel. All study personnel will have to read the manuals.

12.1 Data handling and record keeping / archiving

12.1.1 Case Report Forms

All relevant data is documented in paper CRFs. This data includes inclusion/exclusion criteria, demographic data, medical history, scores of clinical and motor rating scales. Furthermore, side effects, concomitant treatment, reasons for discontinuation will be recorded in the CRF. Timing and conductance of experimental measures, such as posturography, cerebral MRI, cortical excitability, and actigraphy will be recorded recorded in the CRF. However, the real data of these measures is stored electronically outside the CRF.

For each enrolled study participant a CRF is maintained. CRFs will be kept current to reflect subject status at each phase during the course of study. Participants will be coded by use of a participant number in combination with the year of birth.

The paper forms of rating scales (e.g. PANSS) and the electronic data (e.g. MRI, posturography, ect.) are considered source documents. Furthermore, in patients the medical record of our hospital is a source document for the past psychiatric history and current concomitant treatment. All other data (participant history, side effects, ect.) are directly entered into the CRF without further source.

The study personnel is authorized to enter data into the CRFs, the name of the entering person will be documented. CRF data will be recorded in an electronic database, i.e. RedCap-Database. The procedure will include data cross-check between electronic and paper CRF. Any deviation will be corrected by consensus and consulting the CRF forms. RedCap-Database has individualized logins for each member of the study team and provides logs for data entry.

The electronic database for the non-CRF data will be logged via a secure Dropbox folder, thus the person entering the data or changing data can be identified by individualized logins. The log also identifies the document changes and the time of the change. Dropbox allows for restoring each version of the documents within the study folder. A copy of the electronic neuroimaging data will be shared with Collaborator Prof. Jessica Bernard, College Station, TX, USA. At her laboratory, specific neuroimaging analyses will be performed with a subset of the electronic data. The original electronic files will be stored in Bern (see 12.1.3), while the specific neuroimaging analyses data of Prof. Bernard will be stored encrypted at her university for 10 years.

12.1.2 Specification of source documents

As described in 12.1.1. most data in the CRFs is source data. Furthermore, Informed consent form, specific rating scales and electronic data (such as MRI, actigraphy or posturography) are source data. In patients, the medical record of the hospital may contain source data on the psychiatric history. All CRFs and paper source data will be kept in folders. Any extra examination in case of additional safety assessments will be documented on paper and kept as source data in the same folders as CRF and other source data.

12.1.3 Record keeping / archiving

All study data will be archived for 10 years after study termination or premature termination of the trial at the translational research center of the University Hospital of Psychiatry, Bern. In addition, the data of specific neuroimaging analyses performed at Prof. Bernard's laboratory, will be stored encrypted for 10 years at Texas A & M University, College Station, TX, USA. Thus, source data are kept in Bern, and all further handled data will be stored under identical conditions in Bern or Texas Station.

12.2 Data management

Data of all participants will be encrypted and analysis will be performed using encrypted data only. Unblinding of participant-specific data is only possible after consent has been given by the participants and the sponsor. Data of all participants will receive a numerical identification. The key to this encryption code will be stored in a single file at the address below, and it will be accessible only by the sponsor and the principal investigator.

Office of the PI: University of Bern, University Hospital of Psychiatry, Murtenstrasse 21, 3008 Bern, Office 01-132

12.2.1 Data Management System

Describe what system (also software) is being used and who is responsible and how it is tested before the trial (may include a description of where the system is hosted).

12.2.1.1 Neuroimaging data

MRI data are anonymized digital data of appr. 1 GB per subject and scan, we will aim for 278 scans, i.e. total of 300 GB. MRI data sets include the dicom files of the planned sequences fMRI task, fMRI rest, ASL rest, DTI and T1 anatomy. MRI data sets will be safely stored in encrypted formats at the server of the translational research center, university hospital in Bern. Data will be logged with personal logins, such that any change to the files is reproducible. Data analysis will be performed locally in computers of the institute's network. Some analyses are planned to be performed on encrypted data at computers of the study collaborator Prof. Bernard. All files generated during data processing and analysis will be stored. Total data volume is approximately 2 TB. Neuroimaging data will be stored at a dropbox-folder, which is encrypted by Boxcryptor Software and owned by the Translational Research Center, University Hospital of Psychiatry, Bern. The system has been effectively used in several studies. Access to the folder and files is secured by personal login and password. The log of the Dropbox folder enables the identification of data entry/data change according to the logins used. The log will inform on which data has been changed when by whom. Dropbox allows for restoration of each file version, thus each change can be tracked.

12.2.1.2 motor behavioral/instrumental data

Data from actigraphy, posturography, TMS-MEP experiments as well as video recordings of coin rotation will be stored encrypted in digital format. As neuroimaging data, storage will comply with the federal human research act and will be conducted at standard facilities of the translational research center with encrypted and logged server storage. Data access is granted upon personal logins, such that any change to the files is reproducible. Each dataset includes several measures across 4-5 time points for one study participant. We expect appr. 1 GB per subject, total of 130 GB.

12.2.1.3 clinical and demographic data

Clinical data including outcome measures with observer based rating scales or patients' history will be collected. This data is transferred to paper case report forms and will later be anonymously transferred to a red cap data base, hosted by the CTU Bern. Data access is only available to study staff with personalized logins. During the longitudinal assessments approximately 300 variables will be assessed for each participant.

12.2.1.4 blood sampling data

Genetic blood samples will be stored, managed and controlled in Center for Psychiatric Neuroscience, Department of Psychiatry, Lausanne University Hospital, Lausanne following strictly the current Swiss legal requirements for data protection and will be performed according to the Ordinance HRO Art. 5.

12.2.2 Data security, access and back-up

Only the PI and authorized personnel with own logins and passwords will have access to the electronic data. The translational research center Bern runs a daily backup of the dropbox folders. The RedCap Database also has regular backups.

12.2.3 Analysis and archiving

All analyses will be performed with a final copy of the SPSS file including all clinical data. All other analyses (neuroimaging, posturography, actigraphy) will be conducted with the appropriate standard software. Only the PI and authorized personnel will have access to the data to perform the analyses.

12.2.4 Electronic and central data validation

Data will be checked for consistency, e.g. range checks for questionnaire scores or test scores, checks for date entries temporal consistency.

12.3 Monitoring

The principal investigator will check the CRFs for completeness and internal consistency (plausibility of information) and external consistency (with source data) after each participant completed the study. In addition an external monitoring will be performed by Dr. Peter.

Four monitoring dates are planned. The first before the study recruitment starts, the second after the first three participants have been included, the third after 50% of enrolment was achieved and the final

monitoring is planned after the last data had been collected.

Before first recruitment: Check whether all study assessments and instruments are ready and complete.

After the first three participants: Check CRF entries and compliance with source data where possible. Clarify queries of data entry or study procedures.

After the 50% enrolment: Check whether CRFs of the all collected cases are completed and data is consistent.

After the final data collection: Check whether CRFs of the all cases collected after the last monitoring are completed and data is consistent.

12.4 Audits and Inspections

Not intended for this single center trial.

12.5 Confidentiality, Data Protection

All data will be handled strictly confidential. Direct access to the source documents will be permitted only for purposes of inspections (ICHE6, 6.10) by authorities such as the CEC or monitoring by the PI. Only the PI and his team will have access to the protocol, dataset and other study related information during and after the study.

12.6 Storage of biological material and related health data

Coded genetic blood samples will be stored, managed and controlled in Center for Psychiatric Neuroscience, Department of Psychiatry, Lausanne University Hospital, Lausanne following strictly the current Swiss legal requirements for data protection and will be performed according to the Ordinance HRO Art. 5. All electronic data will be archived for 10 years (see 12.1).

Data sharing outside of the project is currently not planned. However, electronic and biological data, particularly neuroimaging electronic data and blood samples, may be later shared with national or international collaborators to address research questions that are currently unknown. The data storage must comply with the same regulations as the data storage of this study. Video data will not be shared with other groups.

13. PUBLICATION AND DISSEMINATION POLICY

Publication of results is the responsibility of the sponsor/investigator. The results of the study will be prepared for publication in peer-reviewed scientific journals, preferably as open-access articles. Authorship will follow the national guidelines. Furthermore, results will be reported at scientific conferences as posters or oral communications. The use of professional writers is not intended. After scientific publication, the results will be prepared for communication in lay man's terms in the media.

Data sharing outside of the project is currently not planned. However, electronic data, particularly neuroimaging electronic data, may be later shared with national or international collaborators to address research questions that are currently unknown. The data storage must comply with the same regulations as the data storage of this study.

14. FUNDING AND SUPPORT

14.1 Funding

This study receives project funding by the Swiss National Science Foundation (grant #182469 to Prof. Sebastian Walther). Additional minor costs will be covered by the University Hospital of Psychiatry in Bern.

14.2 Other Support

Consumables and further material support is provided by the host institution, the Translational Research Center of the University Hospital of Psychiatry, University of Bern, Switzerland.

15. INSURANCE

No special insurance due to category A.

16. REFERENCES

1. Rossi S, Hallett M, Rossini PM, Pascual-Leone A, Safety of TMSCG. Safety, ethical considerations, and application guidelines for the use of transcranial magnetic stimulation in clinical practice and research. Clin Neurophysiol 2009;120:2008-2039.
2. Lefaucheur JP, Andre-Obadia N, Antal A, et al. Evidence-based guidelines on the therapeutic use of repetitive transcranial magnetic stimulation (rTMS). Clin Neurophysiol 2014;125:2150-2206.
3. Owen MJ, Sawa A, Mortensen PB. Schizophrenia. Lancet 2016;388:86-97.
4. Howes OD, Murray RM. Schizophrenia: an integrated sociodevelopmental-cognitive model. Lancet 2014;383:1677-1687.
5. Tandon R, Nasrallah HA, Keshavan MS. Schizophrenia, "just the facts" 4. Clinical features and conceptualization. Schizophr Res 2009;110:1-23.
6. Yang GJ, Murray JD, Wang XJ, et al. Functional hierarchy underlies preferential connectivity disturbances in schizophrenia. Proc Natl Acad Sci U S A 2016;113:E219-228.
7. van den Heuvel MP, Sporns O, Collin G, et al. Abnormal rich club organization and functional brain dynamics in schizophrenia. JAMA Psychiatry 2013;70:783-792.
8. Klauser P, Baker ST, Cropley VL, et al. White Matter Disruptions in Schizophrenia Are Spatially Widespread and Topologically Converge on Brain Network Hubs. Schizophr Bull 2016.
9. Friston KJ. Schizophrenia and the disconnection hypothesis. Acta Psychiatr Scand Suppl 1999;395:68-79.
10. Walther S, Mittal VA. Motor System Pathology in Psychosis. Curr Psychiatry Rep 2017;19:97.
11. Walther S, Stegmayer K, Federspiel A, Bohlhalter S, Wiest R, Viher PV. Aberrant Hyperconnectivity in the Motor System at Rest Is Linked to Motor Abnormalities in Schizophrenia Spectrum Disorders. Schizophr Bull 2017;43:982-992.
12. Mittal V, Bernard JA, Northoff G. What can Different Motor Circuits Tell Us About Psychosis? An RDoC Perspective. Schizophr Bull 2017.
13. Kindler J, Schultze-Lutter F, Michel C, et al. Abnormal involuntary movements are linked to psychosis-risk in children and adolescents: Results of a population-based study. Schizophr Res 2016;174:58-64.
14. Mittal VA, Neumann C, Saczawa M, Walker EF. Longitudinal progression of movement abnormalities in relation to psychotic symptoms in adolescents at high risk of schizophrenia. Arch Gen Psychiatry 2008;65:165-171.
15. van Harten PN, Walther S, Kent JS, Sponheim SR, Mittal VA. The clinical and prognostic value of motor abnormalities in psychosis, and the importance of instrumental assessment. Neurosci Biobehav Rev 2017;80:476-487.
16. Peralta V, Campos MS, De Jalon EG, Cuesta MJ. Motor behavior abnormalities in drug-naive patients with schizophrenia spectrum disorders. Mov Disord 2010;25:1068-1076.
17. Putzhammer A, Perfahl M, Pfeiff L, Hajak G. Correlation of subjective well-being in schizophrenic patients with gait parameters, expert-rated motor disturbances, and psychopathological status. Pharmacopsychiatry 2005;38:132-138.
18. Kao YC, Liu YP, Chou MK, Cheng TH. Subjective quality of life in patients with chronic schizophrenia: relationships between psychosocial and clinical characteristics. Compr Psychiatry 2011;52:171-180.
19. Mittal VA. Cross-Cutting Advancements Usher in a New Era for Motor Research in Psychosis. Schizophr Bull 2016;42:1322-1325.
20. Walther S, Stegmayer K, Sulzbacher J, et al. Nonverbal social communication and gesture control in schizophrenia. Schizophr Bull 2015;41:338-345.
21. Walther S, Eisenhardt S, Bohlhalter S, et al. Gesture Performance in Schizophrenia Predicts Functional Outcome After 6 Months. Schizophrenia Bull 2016;42:1326-1333.
22. Walther S. Psychomotor symptoms of schizophrenia map on the cerebral motor circuit. Psychiatry Res 2015;233:293-298.
23. Morrens M, Hulstijn W, Sabbe B. Psychomotor slowing in schizophrenia. Schizophr Bull 2007;33:1038-1053.
24. Walther S, Strik W. Motor Symptoms and Schizophrenia. Neuropsychobiology 2012;66:77-92.
25. Morrens M, Docx L, Walther S. Beyond boundaries: in search of an integrative view on motor symptoms in schizophrenia. Front Psychiatr 2014;5:145.

26. Docx L, Sabbe B, Provinciael P, Merckx N, Morrens M. Quantitative psychomotor dysfunction in schizophrenia: a loss of drive, impaired movement execution or both? Neuropsychobiology 2013;68:221-227.
27. Walther S, Federspiel A, Horn H, et al. Alterations of white matter integrity related to motor activity in schizophrenia. Neurobiol Dis 2011;42:276-283.
28. Wichniak A, Skowerska A, Chojnacka-Wojtowicz J, et al. Actigraphic monitoring of activity and rest in schizophrenic patients treated with olanzapine or risperidone. J Psychiatr Res 2011;45:1381-1386.
29. Putzhammer A, Heindl B, Broll K, Pfeiff L, Perfahl M, Hajak G. Spatial and temporal parameters of gait disturbances in schizophrenic patients. Schizophr Res 2004;69:159-166.
30. Lisi G, Nico D, Ribolsi M, et al. Asymmetries in initiation of aiming movements in schizophrenia. Neuropsychologia 2018;109:200-207.
31. Docx L, Morrens M, Bervoets C, et al. Parsing the components of the psychomotor syndrome in schizophrenia. Acta Psychiatr Scand 2012;126:256-265.
32. Morrens M, Hulstijn W, Van Hecke J, Peuskens J, Sabbe BG. Sensorimotor and cognitive slowing in schizophrenia as measured by the Symbol Digit Substitution Test. J Psychiatr Res 2006;40:200-206.
33. Fervaha G, Agid O, Takeuchi H, et al. Extrapyramidal symptoms and cognitive test performance in patients with schizophrenia. Schizophr Res 2015;161:351-356.
34. Peralta V, Cuesta MJ. Neuromotor abnormalities in neuroleptic-naive psychotic patients: antecedents, clinical correlates, and prediction of treatment response. Compr Psychiatry 2011;52:139-145.
35. Walther S, Stegmayer K, Horn H, Razavi N, Muller TJ, Strik W. Physical Activity in Schizophrenia is Higher in the First Episode than in Subsequent Ones. Front Psychiatry 2014;5:191.
36. Cuesta MJ, Sanchez-Torres AM, de Jalon EG, et al. Spontaneous parkinsonism is associated with cognitive impairment in antipsychotic-naive patients with first-episode psychosis: a 6-month follow-up study. Schizophr Bull 2014;40:1164-1173.
37. Grootens KP, Vermeeren L, Verkes RJ, et al. Psychomotor planning is deficient in recent-onset schizophrenia. Schizophr Res 2009;107:294-302.
38. Mittal VA, Gupta T, Orr JM, et al. Physical activity level and medial temporal health in youth at ultra high-risk for psychosis. J Abnorm Psychol 2013;122:1101-1110.
39. Dean DJ, Mittal VA. Spontaneous parkinsonisms and striatal impairment in neuroleptic free youth at ultrahigh risk for psychosis. NPJ Schizophr 2015;1.
40. Niendam TA, Bearden CE, Johnson JK, et al. Neurocognitive performance and functional disability in the psychosis prodrome. Schizophr Res 2006;84:100-111.
41. Stubbs B, Ku PW, Chung MS, Chen LJ. Relationship Between Objectively Measured Sedentary Behavior and Cognitive Performance in Patients With Schizophrenia Vs Controls. Schizophr Bull 2017;43:566-574.
42. Stubbs B, Chen LJ, Chung MS, Ku PW. Physical activity ameliorates the association between sedentary behavior and cardiometabolic risk among inpatients with schizophrenia: A comparison versus controls using accelerometry. Compr Psychiatry 2017;74:144-150.
43. Martin-Sierra A, Vancampfort D, Probst M, et al. Walking capacity is associated with health related quality of life and physical activity level in patients with schizophrenia: a preliminary report. Actas Esp Psiquiatr 2011;39:211-216.
44. Gur RC, Ragland JD, Moberg PJ, et al. Computerized neurocognitive scanning: II. The profile of schizophrenia. Neuropsychopharmacology 2001;25:777-788.
45. Lee RS, Hermens DF, Naismith SL, et al. Neuropsychological and functional outcomes in recent-onset major depression, bipolar disorder and schizophrenia-spectrum disorders: a longitudinal cohort study. Transl Psychiatry 2015;5:e555.
46. Boden R, Abrahamsson T, Holm G, Borg J. Psychomotor and cognitive deficits as predictors of 5-year outcome in first-episode schizophrenia. Nord J Psychiatry 2014;68:282-288.
47. Tohen M, Khalsa HM, Salvatore P, et al. The McLean-Harvard First-Episode Project: Early Course in 114 Cases of First-Episode Nonaffective Psychoses. J Clin Psychiatry 2016;77:781-788.
48. Walther S, Koschorke P, Horn H, Strik W. Objectively measured motor activity in schizophrenia challenges the validity of expert ratings. Psychiatry Res 2009;169:187-190.
49. Walther S, Ramseyer F, Horn H, Strik W, Tschacher W. Less structured movement patterns

predict severity of positive syndrome, excitement, and disorganization. Schizophr Bull 2014;40:585-591.

50. Walther S, Stegmayer K, Horn H, et al. The Longitudinal Course of Gross Motor Activity in Schizophrenia - Within and between Episodes. Front Psychiatry 2015;6:10.

51. Gebhardt A, Vanbellingen T, Baronti F, Kersten B, Bohlhalter S. Poor dopaminergic response of impaired dexterity in Parkinson's disease: Bradykinesia or limb kinetic apraxia? Mov Disord 2008;23:1701-1706.

52. Walther S, Vanbellingen T, Muri R, Strik W, Bohlhalter S. Impaired pantomime in schizophrenia: Association with frontal lobe function. Cortex 2013;49:520-527.

53. Kent JS, Hong SL, Bolbecker AR, et al. Motor deficits in schizophrenia quantified by nonlinear analysis of postural sway. PLoS One 2012;7:e41808.

54. Matsuura Y, Fujino H, Hashimoto R, et al. Standing postural instability in patients with schizophrenia: Relationships with psychiatric symptoms, anxiety, and the use of neuroleptic medications. Gait Posture 2015;41:847-851.

55. Peralta V, Cuesta MJ. The effect of antipsychotic medication on neuromotor abnormalities in neuroleptic-naive nonaffective psychotic patients: a naturalistic study with haloperidol, risperidone, or olanzapine. Prim Care Companion J Clin Psychiatry 2010;12.

56. Malla A, Norman R, Scholten D, et al. A comparison of two novel antipsychotics in first episode non-affective psychosis: one-year outcome on symptoms, motor side effects and cognition. Psychiatry Res 2004;129:159-169.

57. Kopala LC, Good KP, Milliken H, et al. Treatment of a first episode of psychotic illness with quetiapine: an analysis of 2 year outcomes. Schizophr Res 2006;81:29-39.

58. Walther S, Federspiel A, Horn H, et al. Resting state cerebral blood flow and objective motor activity reveal basal ganglia dysfunction in schizophrenia. Psychiatry Res 2011;192:117-124.

59. Docx L, Emsell L, Van Hecke W, et al. White matter microstructure and volitional motor activity in schizophrenia: A diffusion kurtosis imaging study. Psychiatry Res 2016;260:29-36.

60. Bracht T, Schnell S, Federspiel A, et al. Altered cortico-basal ganglia motor pathways reflect reduced volitional motor activity in schizophrenia. Schizophr Res 2013;143:269-276.

61. Karbasforoushan H, Duffy B, Blackford JU, Woodward ND. Processing speed impairment in schizophrenia is mediated by white matter integrity. Psychol Med 2015;45:109-120.

62. Singh S, Goyal S, Modi S, et al. Motor function deficits in schizophrenia: an fMRI and VBM study. Neuroradiology 2014;56:413-422.

63. Walther S, Schappi L, Federspiel A, et al. Resting-State Hyperperfusion of the Supplementary Motor Area in Catatonia. Schizophr Bull 2017;43:972-981.

64. Scheuerecker J, Ufer S, Kapernick M, et al. Cerebral network deficits in post-acute catatonic schizophrenic patients measured by fMRI. J Psychiatr Res 2009;43:607-614.

65. Payoux P, Boulanouar K, Sarramon C, et al. Cortical motor activation in akinetic schizophrenic patients: a pilot functional MRI study. Mov Disord 2004;19:83-90.

66. Bunse T, Wobrock T, Strube W, et al. Motor cortical excitability assessed by transcranial magnetic stimulation in psychiatric disorders: a systematic review. Brain Stimul 2014;7:158-169.

67. Hasan A, Wobrock T, Grefkes C, et al. Deficient inhibitory cortical networks in antipsychotic-naive subjects at risk of developing first-episode psychosis and first-episode schizophrenia patients: a cross-sectional study. Biol Psychiatry 2012;72:744-751.

68. Du X, Kochunov P, Summerfelt A, Chiappelli J, Choa FS, Hong LE. The role of white matter microstructure in inhibitory deficits in patients with schizophrenia. Brain Stimul 2017;10:283-290.

69. Hallett M. Transcranial magnetic stimulation: a primer. Neuron 2007;55:187-199.

70. Orosz A, Jann K, Wirth M, Wiest R, Dierks T, Federspiel A. Theta burst TMS increases cerebral blood flow in the primary motor cortex during motor performance as assessed by arterial spin labeling (ASL). Neuroimage 2012;61:599-605.

71. Siebner HR, Filipovic SR, Rowe JB, et al. Patients with focal arm dystonia have increased sensitivity to slow-frequency repetitive TMS of the dorsal premotor cortex. Brain 2003;126:2710-2725.

72. Ji GJ, Yu F, Liao W, Wang K. Dynamic aftereffects in supplementary motor network following inhibitory transcranial magnetic stimulation protocols. Neuroimage 2017;149:285-294.

73. Oxley T, Fitzgerald PB, Brown TL, de Castella A, Daskalakis ZJ, Kulkarni J. Repetitive transcranial magnetic stimulation reveals abnormal plastic response to premotor cortex

stimulation in schizophrenia. Biol Psychiatry 2004;56:628-633.

74. Peralta V, Cuesta MJ. Motor Abnormalities: From Neurodevelopmental to Neurodegenerative Through "Functional" (Neuro)Psychiatric Disorders. Schizophr Bull 2017;43:956-971.

75. Speer AM, Willis MW, Herscovitch P, et al. Intensity-dependent regional cerebral blood flow during 1-Hz repetitive transcranial magnetic stimulation (rTMS) in healthy volunteers studied with H215O positron emission tomography: I. Effects of primary motor cortex rTMS. Biol Psychiatry 2003;54:818-825.

76. Tallabs FA, Hammond-Tooke GD. Theta priming of 1-Hz rTMS in healthy volunteers: effects on motor inhibition. J Clin Neurophysiol 2013;30:79-85.

77. Shirota Y, Ohtsu H, Hamada M, Enomoto H, Ugawa Y, Research Committee on r TMSToPsD. Supplementary motor area stimulation for Parkinson disease: a randomized controlled study. Neurology 2013;80:1400-1405.

78. Benninger DH, Berman BD, Houdayer E, et al. Intermittent theta-burst transcranial magnetic stimulation for treatment of Parkinson disease. Neurology 2011;76:601-609.

79. Kindler J, Homan P, Jann K, et al. Reduced neuronal activity in language-related regions after transcranial magnetic stimulation therapy for auditory verbal hallucinations. Biol Psychiatry 2013;73:518-524.

80. Hoffman RE, Hawkins KA, Gueorguieva R, et al. Transcranial magnetic stimulation of left temporoparietal cortex and medication-resistant auditory hallucinations. Arch Gen Psychiatry 2003;60:49-56.

81. Ray P, Sinha VK, Tikka SK. Adjuvant low-frequency rTMS in treating auditory hallucinations in recent-onset schizophrenia: a randomized controlled study investigating the effect of high-frequency priming stimulation. Ann Gen Psychiatry 2015;14:8.

82. Bais L, Vercammen A, Stewart R, et al. Short and long term effects of left and bilateral repetitive transcranial magnetic stimulation in schizophrenia patients with auditory verbal hallucinations: a randomized controlled trial. PLoS One 2014;9:e108828.

83. Klirova M, Horacek J, Novak T, et al. Individualized rTMS neuronavigated according to regional brain metabolism ((18)FGD PET) has better treatment effects on auditory hallucinations than standard positioning of rTMS: a double-blind, sham-controlled study. Eur Arch Psychiatry Clin Neurosci 2013;263:475-484.

84. Paillere-Martinot ML, Galinowski A, Plaze M, et al. Active and placebo transcranial magnetic stimulation effects on external and internal auditory hallucinations of schizophrenia. Acta Psychiatr Scand 2017;135:228-238.

85. Kindler J, Homan P, Flury R, Strik W, Dierks T, Hubl D. Theta burst transcranial magnetic stimulation for the treatment of auditory verbal hallucinations: results of a randomized controlled study. Psychiatry Res 2013;209:114-117.

86. Vercammen A, Knegtering H, Bruggeman R, et al. Effects of bilateral repetitive transcranial magnetic stimulation on treatment resistant auditory-verbal hallucinations in schizophrenia: a randomized controlled trial. Schizophr Res 2009;114:172-179.

87. Blumberger DM, Vila-Rodriguez F, Thorpe KE, et al. Effectiveness of theta burst versus high-frequency repetitive transcranial magnetic stimulation in patients with depression (THREE-D): a randomised non-inferiority trial. Lancet 2018;391:1683-1692.

88. Duprat R, Desmyter S, Rudi de R, et al. Accelerated intermittent theta burst stimulation treatment in medication-resistant major depression: A fast road to remission? J Affect Disord 2016;200:6-14.

89. van Lutterveld R, Koops S, Schutter DJ, et al. The effect of rTMS on auditory hallucinations: clues from an EEG-rTMS study. Schizophr Res 2012;137:174-179.

90. Nahas Z, Bohning DE, Molloy MA, Oustz JA, Risch SC, George MS. Safety and feasibility of repetitive transcranial magnetic stimulation in the treatment of anxious depression in pregnancy: a case report. J Clin Psychiatry 1999;60:50-52.

91. Klirova M, Novak T, Kopecek M, Mohr P, Strunzova V. Repetitive transcranial magnetic stimulation (rTMS) in major depressive episode during pregnancy. Neuro Endocrinol Lett 2008;29:69-70.

92. Widlöcher D, Ghozlan A. The measurement of retardation in depression. In: Hindmarch I, Stonier PD, eds. Human Psychopharmacology: Measures and Methods. New York: Wiley, 1989.

93. Kay SR, Fiszbein A, Opler LA. The positive and negative syndrome scale (PANSS) for schizophrenia. Schizophr Bull 1987;13:261-276.

94. Kirkpatrick B, Strauss GP, Nguyen L, et al. The brief negative symptom scale: psychometric properties. Schizophr Bull 2011;37:300-305.
95. Craig CL, Marshall AL, Sjostrom M, et al. International physical activity questionnaire: 12-country reliability and validity. Med Sci Sports Exerc 2003;35:1381-1395.
96. Dollfus S, Mach C, Morello R. Self-Evaluation of Negative Symptoms: A Novel Tool to Assess Negative Symptoms. Schizophr Bull 2016;42:571-578.
97. Endicott J, Spitzer RL, Fleiss JL, Cohen J. The global assessment scale. A procedure for measuring overall severity of psychiatric disturbance. Arch Gen Psychiatry 1976;33:766-771.
98. Goldman HH, Skodol AE, Lave TR. Revising axis V for DSM-IV: a review of measures of social functioning. Am J Psychiatry 1992;149:1148-1156.
99. Mausbach BT, Depp CA, Bowie CR, et al. Sensitivity and specificity of the UCSD Performance-based Skills Assessment (UPSA-B) for identifying functional milestones in schizophrenia. Schizophr Res 2011;132:165-170.
100. Huang YZ, Edwards MJ, Rounis E, Bhatia KP, Rothwell JC. Theta burst stimulation of the human motor cortex. Neuron 2005;45:201-206.
101. Oldfield RC. The assessment and analysis of handedness: the Edinburgh inventory. Neuropsychologia 1971;9:97-113.
102. Andreasen NC, Flaum M, Arndt S. The Comprehensive Assessment of Symptoms and History (CASH). An instrument for assessing diagnosis and psychopathology. Arch Gen Psychiatry 1992;49:615-623.
103. Kircher T, Krug A, Stratmann M, et al. A rating scale for the assessment of objective and subjective formal Thought and Language Disorder (TALD). Schizophr Res 2014;160:216-221.
104. Buchanan RW, Heinrichs DW. The Neurological Evaluation Scale (NES): a structured instrument for the assessment of neurological signs in schizophrenia. Psychiatry Res 1989;27:335-350.
105. Fahn S, Elton RL, Members UP. Unified Parkinson's disease rating scale. In: Fahn S, Marsden CD, Goldstein M, Calne DB, eds. Recent developments in Parkinson's disease. Florham Park, NJ: Macmillan Healthcare Information, 1987.
106. Bush G, Fink M, Petrides G, Dowling F, Francis A. Catatonia. I. Rating scale and standardized examination. Acta Psychiatr Scand 1996;93:129-136.
107. Guy W. ECDEU Assessment Manual for Psychopharmacology. Rockville, MD: US Department of Health, Education and

Welfare, 1976.

108. Walther S, Federspiel A, Horn H, et al. White matter integrity associated with volitional motor activity. Neuroreport 2010;21:381-385.
109. Walther S, Horn H, Koschorke P, Muller TJ, Strik W. Increased motor activity in cycloid psychosis compared to schizophrenia. World J Biol Psychiatry 2009;10:746-751.
110. Walther S, Horn H, Razavi N, Koschorke P, Muller TJ, Strik W. Quantitative motor activity differentiates schizophrenia subtypes. Neuropsychobiology 2009;60:80-86.
111. Walther S, Horn H, Razavi N, et al. Higher motor activity in schizophrenia patients treated with olanzapine versus risperidone. J Clin Psychopharmacol 2010;30:181-184.
112. Vanbellingen T, Wapp M, Stegmayer K, et al. Theta burst stimulation over premotor cortex in Parkinson's disease: an explorative study on manual dexterity. J Neural Transm (Vienna) 2016;123:1387-1393.
113. Rossini PM, Burke D, Chen R, et al. Non-invasive electrical and magnetic stimulation of the brain, spinal cord, roots and peripheral nerves: Basic principles and procedures for routine clinical and research application. An updated report from an I.F.C.N. Committee. Clin Neurophysiol 2015;126:1071-1107.
114. Claassen J, Spiegel R, Kalla R, et al. A randomised double-blind, cross-over trial of 4-aminopyridine for downbeat nystagmus--effects on slowphase eye velocity, postural stability, locomotion and symptoms. J Neurol Neurosurg Psychiatry 2013;84:1392-1399.
115. Krafczyk S, Tietze S, Swoboda W, Valkovic P, Brandt T. Artificial neural network: a new diagnostic posturographic tool for disorders of stance. Clin Neurophysiol 2006;117:1692-1698.
116. Genzel L, Dresler M, Cornu M, et al. Medial prefrontal-hippocampal connectivity and motor memory consolidation in depression and schizophrenia. Biol Psychiatry 2015;77:177-186.
117. Yokoi A, Arbuckle SA, Diedrichsen J. The Role of Human Primary Motor Cortex in the Production of Skilled Finger Sequences. J Neurosci 2018;38:1430-1442.

17. APPENDICES

1. IB (according to ISO 14155) – “Produktinformation”

2. TMS device manual

3. Case Report Forms, v2 of December 13th 2018, patient version and control version

4. Study Information for patients, v2 of December 13th 2018

5. Study Information for healthy controls, v2 of December 13th 2018

Overcoming psychomotor slowing in psychosis (OCoPS-P) – A 3-week, randomized, double-blind, placebo-controlled trial of add-on repetitive transcranial magnetic stimulation for psychomotor slowing in psychosis

Clinical Study Protocol

Short Title:	OCoPS-P
Translation:	Überwinden von psychomotorischer Verlangsamung bei Psychosen - eine randomisierte, doppelblinde, plazebo-kontrollierte Studie zur Wirkung von transkranieller Magnetstimulation auf die psychomotorischer Verlangsamung bei Psychosen

Study Type:	Clinical trial testing the effects of 15 sessions of repetitive transcranial magnetic stimulation on psychomotor slowing in psychosis
Study Categorisation:	Risk category A
Study Registration:	Clinicaltrials.gov NCT03921450
Study Identifier:	OCoPS-P, BASEC-Nr: 2018-02164
Sponsor, Sponsor-Investigator or Principal Investigator:	Prof. Dr. med. Sebastian Walther, University Hospital of Psychiatry, University of Bern, Murtenstrasse 21, 3008 Switzerland<br>Phone: 031 632 8979, fax: 031 632 8950, Email: sebastian.walther@upd.unibe.ch
Investigational Product:	Transcranial Magnetic Stimulation
Protocol Version and Date:	Version 4.0 February 18th 2021

CONFIDENTIAL

The information contained in this document is confidential and the property of Prof. Dr. med. Sebastian Walther (the sponsor). The information may not - in full or in part - be transmitted, reproduced, published, or disclosed to others than the applicable Competent Ethics Committee(s) and Regulatory Authority(ies) without prior written authorisation from the sponsor except to the extent necessary to obtain informed consent from those who will participate in the study.

Signature Page(s)

Study number	BASEC 2018-02164
Study Title	Overcoming psychomotor slowing in psychosis (OCoPS-P) – A 3-week, randomized, double-blind, placebo-controlled trial of add-on repetitive transcranial magnetic stimulation for psychomotor slowing in psychosis

The Sponsor-Investigator and trial statistician have approved the protocol version 4.0 (18.02.2021), and confirm hereby to conduct the study according to the protocol, current version of the World Medical Association Declaration of Helsinki, ICH-GCP guidelines or ISO 14155 norm if applicable and the local legally applicable requirements.

Sponsor-Investigator:

Prof. Dr. med. Sebastian Walther

Bern, 18.02.2021	
Place/Date	Signature

Local Principal Investigator at study site*:

I have read and understood this trial protocol and agree to conduct the trial as set out in this study protocol, the current version of the World Medical Association Declaration of Helsinki, ICH-GCP guidelines or ISO 14155 norm and the local legally applicable requirements.

Site	University Hospital of Psychiatry, Bern
Principal investigator	Prof. Dr. med. Sebastian Walther

Place/Date	Signature

Note: In multicentre studies, this page must be individually signed by all participating Local Principal Investigators.

Table of Contents

STUDY SYNOPSIS **7**
ABBREVIATIONS **11**
STUDY SCHEDULE **12**
1. STUDY ADMINISTRATIVE STRUCTURE **15**
1.1 Sponsor, Sponsor-Investigator 15
1.2 Principal Investigator(s) 15
1.3 Statistician ("Biostatistician") 15
1.4 Laboratory 15
1.5 Monitoring institution 15
1.6 Data Safety Monitoring Committee 15
1.7 Any other relevant Committee, Person, Organisation, Institution 15
2. ETHICAL AND REGULATORY ASPECTS **16**
2.1 Study registration 16
2.2 Categorisation of study 16
2.3 Competent Ethics Committee (CEC) 16
2.4 Competent Authorities (CA) 16
2.5 Ethical Conduct of the Study 16
2.6 Declaration of interest 16
2.7 Patient Information and Informed Consent 16
2.8 Participant privacy and confidentiality 17
2.9 Early termination of the study 17
2.10 Protocol amendments 17
3. BACKGROUND AND RATIONALE **18**
3.1 Background and Rationale 18
3.1.1 Schizophrenia 18
3.1.2 Motor system pathology in schizophrenia 18
3.1.3 Psychomotor slowing 19
3.1.4 Current treatment options of psychomotor slowing 19
3.1.5 Psychomotor slowing and motor network dysfunction 19
3.1.6 Intracortical excitability 20
3.1.7 Brain stimulation for psychomotor slowing 20
3.1.8 State of research summary 21
3.2 Investigational Product (treatment, device) and Indication 21
3.3 Preclinical Evidence 21
3.4 Clinical Evidence to Date 22
3.5 Dose Rationale / Medical Device: Rationale for the intended purpose in study (pre-market MD) 22
3.6 Explanation for choice of comparator (or placebo) 22
3.7 Risks / Benefits 22
3.8 Justification of choice of study population 23
4. STUDY OBJECTIVES **23**
4.1 Overall Objective 23
4.2 Primary Objective 24
4.3 Secondary Objectives 24
4.4 Safety Objectives 24
5. STUDY OUTCOMES **24**
5.1 Primary Outcome 24

5.2 Secondary Outcomes 24
5.2.1 Clinical outcomes 24
5.2.2 Behavioral outcomes 24
5.2.3 Physiological outcomes 24
5.2.4 Neuroimaging outcomes 25
5.3 Other Outcomes of Interest 25
5.4 Safety Outcomes 25
6. STUDY DESIGN 25
6.1 General study design and justification of design 25
6.2 Methods of minimising bias 25
6.2.1 Randomisation 25
6.2.2 Blinding procedures 26
6.2.3 Other methods of minimising bias 26
6.3 Unblinding Procedures (Code break) 26
7. STUDY POPULATION 26
7.1 Eligibility criteria 26
7.2 Recruitment and screening 27
7.3 Assignment to study groups 27
7.4 Criteria for withdrawal / discontinuation of participants 27
8. STUDY INTERVENTION 27
8.1 Identity of Investigational Products (treatment / medical device) 27
8.1.1 Experimental Intervention (treatment / medical device) 27
8.1.2 Control Intervention (standard/routine/comparator treatment / medical device) 27
8.1.3 Packaging, Labelling and Supply (re-supply) 28
8.1.4 Storage Conditions 28
8.2 Administration of experimental and control interventions 28
8.2.1 Experimental Intervention 28
8.2.2 Control Intervention 28
8.3 Dose / Device modifications 28
8.4 Compliance with study intervention 28
8.5 Data Collection and Follow-up for withdrawn participants 28
8.6 Trial specific preventive measures 29
8.7 Concomitant Interventions (treatments) 29
8.8 Study Drug / Medical Device Accountability 29
8.9 Return or Destruction of Study Drug / Medical Device 29
9. STUDY ASSESSMENTS 29
9.1 Study flow chart(s) / table of study procedures and assessments 29
9.1.1 Patients of treatment groups 1-3 29
9.1.2 Patients of treatment group 4 (waiting group) 32
9.1.3 Control subjects 36
9.2 Assessments of outcomes 38
9.2.1 Assessment of primary outcome 38
9.2.2 Assessment of secondary outcomes 38
9.2.3 Assessment of other outcomes of interest 40
9.2.4 Assessment of safety outcomes 40
9.2.5 Assessments in participants who prematurely stop the study 40
9.3 Procedures at each visit 41
9.3.1 Screening visit 41

9.3.2 Visit 2 (baseline, week 0) 41
9.3.3 Visit 3 and 4 (week 1 and 2) 41
9.3.4 Visit 5 (end of intervention, week 3) 41
9.3.5 Visit 6 and 7 (follow-up, week 6 and 24) 42
10. SAFETY 42
10.1 Medical Device Category A studies 42
10.1.1 Definition and Assessment of safety related events 42
10.1.2 Reporting of (Serious) Adverse Events and other safety related events 43
10.1.3 Follow up of (Serious) Adverse Events 44
11. STATISTICAL METHODS 44
11.1 Hypotheses 44
11.2 Determination of Sample Size 45
11.3 Statistical criteria of termination of trial 45
11.4 Planned Analyses 45
11.4.1 Datasets to be analysed, analysis populations 45
11.4.2 Primary Analysis 45
11.4.3 Secondary Analyses 45
11.4.4 Interim analyses 46
11.4.5 Safety analysis 46
11.4.6 Deviation(s) from the original statistical plan 46
11.5 Handling of missing data and drop-outs 46
12. QUALITY ASSURANCE AND CONTROL 46
12.1 Data handling and record keeping / archiving 46
12.1.1 Case Report Forms 46
12.1.2 Specification of source documents 47
12.1.3 Record keeping / archiving 47
12.2 Data management 47
12.2.1 Data Management System 47
12.2.2 Data security, access and back-up 48
12.2.3 Analysis and archiving 48
12.2.4 Electronic and central data validation 48
12.3 Monitoring 48
12.4 Audits and Inspections 48
12.5 Confidentiality, Data Protection 48
12.6 Storage of biological material and related health data 48
13. PUBLICATION AND DISSEMINATION POLICY 49
14. FUNDING AND SUPPORT 49
14.1 Funding 49
14.2 Other Support 49
15. INSURANCE 49
16. REFERENCES 49
17. APPENDICES 54

STUDY SYNOPSIS

**Sponsor / Sponsor-Investigator**	Prof. Dr. med. Sebastian Walther,<br>University Hospital of Psychiatry, University of Bern, Murtenstrasse 21, 3008 Switzerland<br>Phone: 031 632 8979, fax: 031 632 8950, Email: sebastian.walther@upd.unibe.ch
**Study Title:**	Overcoming psychomotor slowing in psychosis (OCoPS-P) – A 3-week, randomized, double-blind, placebo-controlled trial of add-on repetitive transcranial magnetic stimulation for psychomotor slowing in psychosis
**Short Title / Study ID:**	OCoPS-P, KEK 2018-02164
**Protocol Version and Date:**	Protocol version 3.0 (10th June 2020)
**Trial registration:**	clinicaltrials.gov, NCT03921450
**Study category and Rationale**	Category A: TMS device with CE certificate and use according to guidelines and manual. In addition short cerebral MRI and TMS experiments at baseline and endpoint. Minimal risk involved.
**Clinical Phase:**	Not applicable.

**Background and Rationale:**	Schizophrenia is a chronic disorder causing tremendous burden to the patients, families and society. Besides prominent symptoms such as hallucinations, delusions, and thought disorder, the majority of patients also experiences motor abnormalities. Converging evidence links aberrant structure and function of the cerebral motor network to schizophrenia pathology, particularly to motor abnormalities. One of the most frequent motor abnormalities is psychomotor slowing (PS), which may impact both gross and fine motor behavior. While PS causes significant distress and predicts poor outcome, researchers are just starting to understand its pathobiology. First evidence points to aberrant functional and structural connectivity within the cerebral motor network in schizophrenia patients with PS, particularly in connections between premotor/motor cortex and thalamus, as well as between motor cortex and cerebellum. In addition, severe motor inhibition was linked to increased neural activity in the premotor cortex. Repetitive transcranial magnetic stimulation (rTMS) may temporarily alter brain activity. Pilot data from an ongoing double blind RCT indicate that 15 sessions of inhibitory rTMS on the premotor cortex alleviate PS. The pathobiology of PS, however, is still unknown. This study will combine a motor battery, advanced neuroimaging, and rTMS to probe the cerebral motor network contributions to PS.<br><br>The aims of this study are<br><br>(1) to investigate clinical and neural changes following 3 weeks of rTMS treatment (inhibitory, facilitatory or placebo), (2) to characterize neural correlates of psychomotor slowing (PS) on the network level, (3) to explore short term changes of PS by testing a waiting list cohort, and (4) to test the clinical outcome of rTMS for PS at 6-month follow-up.<br><br>To reach these aims, we plan to investigate four groups of schizophrenia patients (total 88) in a randomized, double blind, 4-arm sham-controlled trial of 15 rTMS sessions in 3 weeks with pre and post intervention MRI scans and a clinical follow-up at 6 months. One group will first be kept on a 3 week waiting list and then enter the study. Longitudinal MRI scans and motor tests separated by 3 weeks will also be applied to a healthy control group of 40 subjects without any psychiatric illness for comparisons with the patient groups. In addition, a clinical control group of 25 patients without symptoms of PS will be scanned and tested at a single time point to distinguish between findings associated specifically with PS and findings associated with schizophrenia in general. We hypothesize that (1) PS would be linked to increased functional connectivity in motor cortical-basal ganglia loops as well as motor cortical-cerebellar loops in comparison to healthy and clinical controls, (2) inhibitory rTMS to the premotor cortex will reduce motor network functional connectivity and thus alleviate PS, (3) patients on the waiting list may experience stable PS severity, and finally, (4) patients responding to rTMS treatment of PS will have superior clinical and functional outcomes at 6-month follow-up. Thus, the study will substantially contribute to the understanding of PS by describing and probing the neural alterations in the motor network in schizophrenia associated with behavioral PS. Therefore, the study will impact future treatment strategies for PS and inform on the causal network pathology in schizophrenia
**Objective(s):**	1. investigate clinical and neural changes following 3 weeks of rTMS treatment (inhibitory, facilitatory or placebo)<br>2. characterize neural correlates of psychomotor slowing (PS) on the network level<br>3. explore short term changes of PS by testing a waiting list cohort<br>4. test the clinical outcome of rTMS for PS at 6-month follow-up.

**Outcome(s):**	1. Change from baseline to week 3 in SRRS scores. Furthermore, proportion of responders per study arm after 15 sessions rTMS (response = 30% reduction in SRRS scores from baseline)<br>2. Associations between multiple measures of motor function and neuroimaging markers, e.g. resting state perfusion or functional connectivity within the motor system<br>3. Change in SRRS from baseline to week 3 within the waiting list group, also comparison of SRRS change from baseline between waiting list group and placebo group.<br>4. Change in symptoms (PANSS, BNSS, SRRS) and functioning (SOFAS) from baseline to 6-month follow-up
**Study design:**	randomised, double-blind, four-arm, placebo-controlled trial of 3 weeks add-on rTMS for psychomotor slowing in schizophrenia spectrum disorders
**Inclusion / Exclusion criteria:**	Inclusion:<br>• Right-handed subjects, ages 18–60 years.<br>• Patients: schizophrenia spectrum disorders according to DSM-5 with psychomotor slowing (SRRS score ≥ 15). Patients are necessary, because only patients have the target symptoms, i.e. psychomotor slowing<br>• Healthy controls: only for pre-/post comparisons of neuroimaging and physiology, **no intervention in healthy controls**<br>• Clinical controls: schizophrenia spectrum disorders according to DSM-5 without psychomotor slowing (SRRS score < 15), **no intervention in clinical controls**<br>Exclusion:<br>• General: Substance abuse or dependence other than nicotine. Past or current medical or neurological condition associated with impaired or aberrant movement, such as brain tumors, stroke, M. Parkinson, M. Huntington, dystonia, or severe head trauma with subsequent loss of conciousness. Epilepsy or other convulsions. History of any hearing problems or ringing in the ears. Standard exclusion criteria for MRI scanning and TMS; e.g. metal implants, claustrophobia. Women who are pregnant or breast feeding<br>• Patients only: any TMS treatment in the past 3 months<br>• Controls: history of any psychiatric disorder. First-degree relatives with schizophrenia spectrum disorders.
**Measurements and procedures:**	Participants will be screened and randomized to one of four arms before baseline assessments. Intervention period will be three weeks. Each week the primary outcome variable and safety will be assessed. At baseline and end of intervention (week 3), patients will be assessed with clinical and motor rating scales, tasks assessing fine and gross motor behaviour, TMS measures of cortical excitability, posturography, MRI neuroimaging, and tests of social and community functioning. Follow-ups will be conducted at week 6 and 24 including clinical and motor measures, cortical excitability, and social and community functioning. For cross-sectional comparisons of cortical excitability and neuroimaging, a group of 40 healthy control subjects matched for age, gender, and education, will be tested longitudinally with neuroimaging, motor tests, cortical excitability and posturography at baseline and week 3. For the comparison of the baseline neuroimaging and physiological data a group of 25 clinical control subjects matched for age, gender, and education will be tested once at baseline. Controls will not receive any intervention.
**Study Product / Intervention:**	low-frequency rTMS has inhibitory effects on brain function. We will apply 1’000 pulses at 1 Hz over the left SMA at an intensity of 110% of the resting motor threshold (approximate duration 17 minutes) in a total of 15 daily sessions (5 per week)

**Control Intervention (if applicable):**	**Active control:** Intermittent theta burst (iTBS) enhances local brain activity. We will apply 600 pulses at 50 Hz (stimulation in 2 sec trains every 10 sec for 190 sec in total) over the left SMA at an intensity of 80% of the resting motor threshold. iTBS will be repeated after15 min totaling to 1200 pulses per session in a total of 15 daily sessions (5 per week).<br>**Placebo control:** We will use a placebo-coil that looks identical to the real one and makes identical noises. Stimulation parameters are the same as in the active intervention, except that no magnetic pulse is emitted. Thus, placebo coil will be placed over the left SMA with 1000 clicks at 1 Hz (approximate duration 17 minutes) in a total of 15 daily sessions (5 per week).<br>**Waiting list:** This group will have baseline measures at baseline and week 3 and receive the active protocol from week 3 to week 6.
**Number of Participants with Rationale:**	Number of participants in the intervention: 88 patients (22 per protocol arm). In addition, to complement neuroimaging and neurophysiological analyses (no intervention) we will include 40 healthy control subjects and 25 clinical control subjects matched for age, gender, and education.
**Study Duration:**	Total study duration will be 4 years. Total duration of participant recruitment will be 3 years.
**Study Schedule:**	Planned 03/2019 of First-Participant-In<br>Planned 06/2022 of Last-Participant-Out
**Investigator(s):**	- see Sponsor-Investigator
**Study Centre(s):**	Single-centre trial at the University Hospital of Psychiatry, Bern
**Statistical Considerations:**	The main effects of the 3-week interventions on PS (SRRS scores) will be calculated in a repeated measures design including 2 measures (Baseline, week 3) and four groups (lf-rTMS, iTBS, sham, waiting group-lf-rTMS). Assuming a moderate effect size (f = 0.23) as indicated by our pilot data in a repeated measures ANOVA with moderate correlation between time points (0.5), a power of 0.95 and an alpha = 0.05, we would need 88 patients (22 per group).
**GCP Statement:**	This study will be conducted in compliance with the protocol, the current version of the Declaration of Helsinki, the ICH-GCP or ISO EN 14155 (as far as applicable) as well as all national legal and regulatory requirements.

ABBREVIATIONS

AE	Adverse Event
BASEC	Business Administration System for Ethical Committees, (https://submissions.swissethics.ch/en/)
BNSS	Brief Negative Symptom Scale
CA	Competent Authority (e.g. Swissmedic)
CEC	Competent Ethics Committee
CRF	Case Report Form
ClinO	Ordinance on Clinical Trials in Human Research *(in German: KlinV, in French: OClin, in Italian: OSRUm)*
eCRF	Electronic Case Report Form
CTCAE	Common terminology criteria for adverse events
DSUR	Development safety update report
GCP	Good Clinical Practice
IB	Investigator's Brochure
Ho	Null hypothesis
H1	Alternative hypothesis
HRA	Federal Act on Research involving Human Beings *(in German: HFG, in French: LRH, in Italian: LRUm)*
IMP	Investigational Medicinal Product
IIT	Investigator-initiated Trial
ISO	International Organisation for Standardisation
iTBS	Intermittent Theta Burst Stimulation
ITT	Intention to treat
MD	Medical Device
MedDO	Medical Device Ordinance *(in German: MepV, in French: ODim)*
PANSS	Positive And Negative Syndrome Scale
PI	Principal Investigator
PS	Psychomotor Slowing
rTMS	Repetitive Transcranial Magnetic Stimulation
SAE	Serious Adverse Event
SDV	Source Data Verification
SMA	Supplementary Motor Area
SOP	Standard Operating Procedure
SPC	Summary of product characteristics
SRRS	Salpêtrière Retardation Rating Scale
SOFAS	Social and Occupational Functioning Scale
SUSAR	Suspected Unexpected Serious Adverse Reaction
TMF	Trial Master File
UPSA Brief	UCSD Performance-based Skills Assessment Brief version

STUDY SCHEDULE

Study Periods	Screening	Treatment, Intervention Period				Follow-up	
Visit	1	2*	3	4	5	6	7
Time (week)	-1	0	1	2	3	6	24
Patient Information and Informed Consent	x						
Demographics	x						
CASH and SCID (history)	x						
In- /Exclusion Criteria	x						
Physical Examination	x						
Pregnancy Test	x						
Psychopathology (TALD, NES)		x					
Randomisation		x					
Administer rTMS (5 sessions per week)		x	x	x			
Primary Variable SRRS		x	x	x	x	x	x
Motor scales UPDRS, BFCRS, AIMS, IPAQ		x			x	x	x
Psychopathology (PANSS, BNSS, SNS)		x			x	x	x
Actigraphy and coin rotation		x			x	x	x
Posturography		x			x		
TMS cortical excitability		x			x	x	x
Grip force measurement		x			x	x	x
Cerebral MRI		x			x		
Blood sampling		x			x		
Functional outcome (SOFAS, GAF, UPSA-brief)		x				x	x
Concomitant Therapy		x	x	x	x		
Adverse Events		x	x	x	x		

* please note that in the waiting group assessments of visit 2 will be repeated after 3 weeks and thereafter the protocol will be identical (see below).

Study protocol for patients in the waiting group

Study Periods	Scre ening	Treatment, Intervention Period					Follow-up	
Visit	1	2	3	4	5	6	7	8
Time (week)	-1	0	3	4	5	6	9	24
Patient Information and Informed Consent	x							
Demographics	x							
CASH and SCID (history)	x							
In- /Exclusion Criteria	x							
Physical Examination	x							
Pregnancy Test	x							
Psychopathology (TALD, NES)		x						
Randomisation		x						
Administer rTMS (5 sessions per week)			x	x	x			
Primary Variable SRRS		x	x	x	x	x	x	x
Motor scales UPDRS, BFCRS, AIMS, IPAQ		x	x			x	x	x
Psychopathology (PANSS, BNSS, SNS)		x	x			x	x	x
Actigraphy and coin rotation		x	x			x	x	x
Posturography		x	x			x		
TMS cortical excitability		x	x			x	x	x
Grip force measurement		x	x			x	x	x
Cerebral MRI		x	x			x		
Blood sampling		x				x		
Functional outcome (SOFAS, GAF, UPSA-brief)		x	x				x	x
Concomitant Therapy		x	x	x	x	x		
Adverse Events			x	x	x	x		

Study protocol for healthy control subjects

Study Periods	Screening	Observation period	
Visit<br>Time (week)	1<br>-1	2<br>0	3<br>3
Proband Information and Informed Consent	x		
Demographics	x		
SCID	x		
In- /Exclusion Criteria	x		
Physical Examination	x		
Pregnancy Test	x		
Primary Variable SRRS		x	x
Motor scales UPDRS, BFCRS, AIMS, IPAQ		x	x
Actigraphy and coin rotation		x	x
Posturography		x	x
TMS cortical excitability		x	x
Grip force measurement		x	x
Cerebral MRI		x	x
Blood sampling		x	x

Study protocol for clinical control subject

Study Periods	Screening	Assessment day
Visit<br>Time (week)	1<br>-1	2<br>0
Proband Information and Informed Consent	x	
Demographics	x	
SCID	x	
In- /Exclusion Criteria	x	
Physical Examination	x	
Pregnancy Test	x	
Psychopathology (TALD. NES)		x
Primary Variable SRRS		x
Motor scales UPDRS, BFCRS, AIMS, IPAQ		x
Psychopathology (PANSS, BNSS, SNS)		x
Actigraphy and coin rotation		x
Posturography		x
TMS cortical excitability		x
Grip force measurement		x
Cerebral MRI		x
Blood sampling		x
Functional outcome (SOFAS, GAF, UPSA-brief)		x
Concomitant Therapy		x

1. STUDY ADMINISTRATIVE STRUCTURE

1.1 Sponsor, Sponsor-Investigator

Prof. Dr. med. Sebastian Walther,

University Hospital of Psychiatry, University of Bern, Murtenstrasse 21, 3008 Bern, Switzerland, phone: 031 632 4635, fax: 031 632 8950, Email: sebastian.walther@upd.unibe.ch

Prof. Walther will be Sponsor-Investigator; no further international sites are planned

Roles: protocol, study design, supervision of data collection and management, data analysis, data interpretation and writing of the report.

1.2 Principal Investigator(s)

Identical to sponsor, see 1.1.

1.3 Statistician ("Biostatistician")

Dr. Petra Viher, PhD, University Hospital of Psychiatry, University of Bern, Bolligenstrasse 111, 3000 Bern, Switzerland. Phone: 031 930 9757, Email: petra.viher@upd.unibe.ch

1.4 Laboratory

All of the procedures except neuroimaging will be performed at the University Hospital of Psychiatry, Bern. The devices and infrastructure are provided by the Translational research center at the University Hospital of Psychiatry, Bern, Bolligenstrasse 111, 3000 Bern 60, Switzerland.

Neuroimaging acquisition (magnetic resonance imaging – MRI) will be performed at the University Hospital Inselspital Bern, Institute of Diagnostic and Interventional Neuroradiology. Collaborator Prof. Dr. med. Roland Wiest.

1.5 Monitoring institution

Dr. phil. Jessica Peter, University Hospital of Old Age Psychiatry and Psychotherapy, University of Bern, Bolligenstrasse 111, 3000 Bern, Switzerland. Phone: 031 932 8903 Email: Jessica.peter@upd.unibe.ch

1.6 Data Safety Monitoring Committee

DSMC is not needed. The study aim is not to test the efficacy of a specific product. The objective is to test whether repetitive transcranial magnetic stimulation may improve psychomotor slowing and how it interferes with neurophysiology.

1.7 Any other relevant Committee, Person, Organisation, Institution

Study collaborators

Prof. Dr. Roland Wiest, University Institute of Diagnostic and Interventional Neuroradiology, Inselspital Bern, Switzerland. MRI acquisition

Prof. Dr. Andrea Federspiel, Neuroimaging Unit, Translational Research Center, University Hospital of Psychiatry, Bern, Switzerland, MRI analyses

Prof. Dr. Jessica Bernard, Department of Psychological and Brain Sciences, Texas A & M University, College Station, TX, USA, MRI analyses support

Prof. Dr. Roger Kalla, Department of Neurology, University Hospital Inselspital, Bern, Switzerland, posturography

Prof. Kim Q. Do, Center of Psychiatric Neuroscience, Department of Psychiatry, Lausanne University Hospital, Lausanne, Switzerland. Blood Analysis

2. ETHICAL AND REGULATORY ASPECTS

The decision of the CEC concerning the conduct of the study will be made in writing to the Sponsor-Investigator before commencement of this study. The clinical study can only begin once approval from all required authorities has been received. Any additional requirements imposed by the authorities shall be implemented.

2.1 Study registration

Registration is planned in ClinicalTrials.gov and the Swiss KOFAM site.

2.2 Categorisation of study

The trial is in Risk Category A. There is only minimal risk associated with the trial. The TMS device has CE certification and approved for clinical use. It will be applied according to the manual and guidelines[1, 2]. TMS has been widely used in neuroscience, and in clinical trials on depression, schizophrenia and chronic pain. Participants will receive 15 daily stimulations. Effects are expected to last for 2-4 weeks after the last stimulation. TMS is also safe in repeated administration[1, 2].

Assessments include standard clinical rating scales, short specific tests of motor behaviour, and a standard cerebral magnetic resonance imaging at baseline and after 3 weeks of stimulation. All assessments have been applied to schizophrenia patients before and are generally well tolerated. In addition, biological material is sampled for further research use.

2.3 Competent Ethics Committee (CEC)

The local Bern Ethics Committee is the Competent Ethics Committee (CEC). No sites outside the canton of Bern are planned.

The Bern Ethics Committee will receive reports of all changes in the research activity and all unanticipated problems involving risks to humans; including in case of planned or premature study end and the final report. No changes are made to the protocol without prior Sponsor and CEC approval, except where necessary to eliminate apparent immediate hazards to study participants. Premature study end or interruption of the study is reported within 15 days. The regular end of the study is reported to the CEC within 90 days, the final study report shall be submitted within one year after study end. Amendments are reported according to chapter 2.10.

2.4 Competent Authorities (CA)

Not applicable – Category A

2.5 Ethical Conduct of the Study

The study will be carried out in accordance to the protocol and with principles enunciated in the current version of the Declaration of Helsinki, the guidelines of Good Clinical Practice (GCP) issued by ICH, in case of medical device: the European Regulation on medical devices 2017/745 and the ISO Norm 14155 and ISO 14971, the Swiss Law and Swiss regulatory authority's requirements. The CEC and regulatory authorities will receive annual safety and interim reports and be informed about study stop/end in agreement with local requirements.

2.6 Declaration of interest

There is no conflict of interest. Funding is provided by independent grants and the Swiss National Science Foundation (see funding 14.1).

2.7 Patient Information and Informed Consent

Participants will be informed by members of the study team about the aims of the study, planned procedures and risks involved. They will receive written information on the study. This information will be provided prior to study inclusion during screening. The participants will also be informed about the compensation of 200 CHF for patients and healthy controls and 50 CHF for clinical controls, respectively, after they completed the study procedures. The participant will be informed by the investigators that his/her medical records may be examined by authorised individuals other than their treating physician.

All participants for the study will be provided a participant information sheet and a consent form describing the study and providing sufficient information for participant to make an informed decision about their participation in the study. A minimum of 24 h time will be given to participants to decide on whether to participate or not. Whenever necessary, the potential participant can take up to 2 weeks to decide on participation.

The patient information sheet and the consent form will be submitted to the CEC and to the competent authority (as applicable) to be reviewed and approved. The formal consent of a participant, using the approved consent form, must be obtained before the participant is submitted to any study procedure.

The participant should read and consider the statement before signing and dating the informed consent form, and should be given a copy of the signed document. The consent form must also be signed and dated by the investigator (or his designee) and it will be retained as part of the study records.

2.8 Participant privacy and confidentiality

The investigator affirms and upholds the principle of the participant's right to privacy and that they shall comply with applicable privacy laws. Especially, anonymity of the participants shall be guaranteed when presenting the data at scientific meetings or publishing them in scientific journals.

Individual subject medical information obtained as a result of this study is considered confidential and disclosure to third parties is prohibited. Subject confidentiality will be further ensured by utilising subject identification code numbers to correspond to treatment data in the computer files.

For data verification purposes, authorised representatives of the Sponsor (-Investigator) or an ethics committee may require direct access to parts of the medical records relevant to the study, including participants’ medical history.

2.9 Early termination of the study

The Sponsor-Investigator may terminate the study prematurely according to certain circumstances, for example:

- ethical concerns,
- insufficient participant recruitment,
- when the safety of the participants is doubtful or at risk, respectively,
- alterations in accepted clinical practice that make the continuation of a clinical trial unwise,
- early evidence of benefit or harm of the experimental intervention

2.10 Protocol amendments

Substantial amendments are only implemented after approval of the CEC and CA respectively.

Under emergency circumstances, deviations from the protocol to protect the rights, safety and well-being of human subjects may proceed without prior approval of the sponsor and the CEC/CA. Such deviations shall be documented and reported to the sponsor and the CEC/CA as soon as possible.

All non-substantial amendments are communicated to the CA as soon as possible if applicable and to the CEC within the Annual Safety Report (ASR).

3. BACKGROUND AND RATIONALE

3.1 Background and Rationale

3.1.1 Schizophrenia

Schizophrenia is a severe disorder with a life-long course affecting approximately 2-3% of the population. Even though the outcome is heterogeneous, schizophrenia usually causes tremendous individual burden, intense costs for society, reduced quality of life, impaired occupational performance and reduction of life expectancy by 10-20 years[3]. Schizophrenia is an adolescent-onset disorder with neurodevelopmental origins[3]. Current models suggest that genetic risk, early hazards to brain maturation, social adversities during childhood and the evolution of cognitive biases predispose subjects to psychosis in times of stress[4]. The schizophrenia syndrome is characterized by symptom clusters including positive symptoms (e.g. delusions and hallucinations), negative symptoms (e.g. avolition and affective flattening), disorganized thought, motor abnormalities, mood disturbances, impaired cognition, anxiety and lack of insight[5]. Brain structure and function in schizophrenia is abnormal at multiple levels. For example, schizophrenia patients share altered organization of functional brain networks[6] and underlying white matter fiber connections[7, 8]. Therefore, schizophrenia has been conceptualized as a disconnection syndrome, which may explain some of the typical symptoms[9]. Indeed, first evidence indicates an association between abnormal motor behavior and structural as well as functional alterations in the motor network in schizophrenia[10, 11]. Researchers are just starting to understand the network alterations linked to clinical symptoms in schizophrenia[12]. **The ultimate goal** would be to **normalize altered brain function at the network level** in order to **improve the clinical significant behavioral abnormalities**.

3.1.2 Motor system pathology in schizophrenia

A set of motor abnormalities including signs such as bizarre posture, peculiar gait, facial and limb dyskinesia, immobility, rigor, or excessive movement have been reported in schizophrenia.

Motor abnormalities have long been exclusively linked to medication side effects; however, converging evidence demonstrates that motor abnormalities frequently occur before medication is commenced and even long before the onset of psychosis[10, 13-15]. Up to 67% of first episode, treatment naïve patients experience at least one motor sign[16]. The contribution of medication is heterogeneous and probably overestimated.

Motor abnormalities are key cues for stigmatization and pose significant distress on patients[17, 18]. Furthermore, recent evidence supports the predictive value of motor abnormalities in psychosis for poor functional outcome[15, 19]. For example, motor abnormalities impact nonverbal behaviors such as gestures[20], which are critical for social functioning[21]. This way, motor abnormalities contribute to poor functional outcome in schizophrenia.

A large network of the brain is devoted to motor behavior, including cortical frontal motor and premotor areas, the basal ganglia, thalamus, brainstem and cerebellum (see figure 1). Evidence across multiple neuroimaging approaches supports that schizophrenia pathobiology is tied to alterations within the cerebral motor system[22].

Figure 1. Key components of the motor network

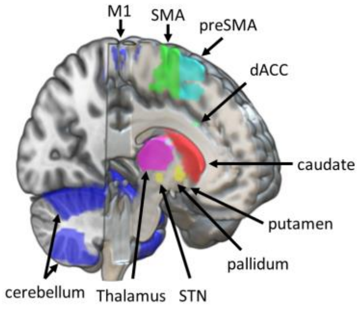

M1 primary motor cortex

SMA supplementary motor area

pre-SMA pre-supplementary motor area

dACC dorsal anterior cingulate cortex

STN subthalamic nucleus

3.1.3 Psychomotor slowing

One important domain of motor abnormalities in schizophrenia is psychomotor slowing (PS), which can be observed in fine motor behavior such as writing, gross motor behavior such as gait, and it may refer to single movements or continuous movement. PS includes movement planning, initiation, execution, timing and motor control [15, 23-25]. Typical examples of PS in schizophrenia are reduced levels of spontaneous gross motor activity as measured by actigraphy[11, 26-28], slowed gait[29], slowed aiming arm movements[30], slowing in fine motor tasks[26, 31, 32] or in bradykinesia of parkinsonism[31].

Multiple reports suggest that 30-50% of schizophrenia patients present with PS[33, 34]. Furthermore, PS is seen in all stages of the disorder. Even though, PS severity seems to increase with illness chronicity[35], it does also occur in early psychosis or in subjects at risk for psychosis[34, 36-40]. In psychosis, PS is critically linked to several disadvantages[23]. For example, PS is associated with poor cognition, sedentary behavior and cardiometabolic risks[41, 42]. Moreover, PS correlates with distress and poor quality of life[17, 43, 44]. Finally, several reports indicate that PS predicts poor outcome in terms of cognition, quality of life and real world functioning in early psychosis, although at baseline patients with PS were not specifically impaired in function[36, 45-47]. In sum, **PS is a frequently observed phenomenon across schizophrenia stages**, associated with poor quality of life, sedentary behavior and predictive of poor cognition and outcome. In order to design new treatment options to overcome this problematic symptom domain, it is essential to know the pathobiology of PS.

PS is particularly suitable for objective instrumental assessment. There are valid measures of fine motor and gross motor slowing that can be acquired by instrumentation[15]. These measures allow dimensional assessment of PS and are therefore ideal candidates when exploring associations between brain and behavior. Instrumentation is also not prone to conceptual overlap. We have applied wrist actigraphy in a series of studies and repeatedly demonstrated reduced gross motor activity in schizophrenia, which was linked to negative syndrome severity and psychomotor slowing as measured by the Salpêtrière Retardation Rating Scale (SRRS)[26, 48-50]. Likewise, finger tapping test as a measure of PS in fine motor behavior is also consistently poorer in schizophrenia patients and linked to negative symptoms[31]. In addition, the video rated coin rotation test is a simple and reliable measure of manual dexterity[51, 52]. Another interesting marker of motor behavior is increased postural sway, which results from poor cerebellar function and is increased in psychosis[53, 54]. Postural sway was correlated with negative syndrome severity in patients[53, 54]. Therefore, we expect increased postural sway to be associated with PS in schizophrenia. In sum, **PS can be reliably measured by observer ratings** such as the **SRRS** or by **instrumental measures** of gross motor behavior (**activity level** from actigraphy) or fine motor behavior (**finger tapping** and **coin rotation**).

3.1.4 Current treatment options of psychomotor slowing

As mentioned above, motor symptoms including PS are neither simply explained by antipsychotic drug effects[23] nor does PS disappear after antipsychotic drug withdrawal. An excellent study in treatment naïve first episode subjects demonstrated the heterogeneity of drug effects on motor abnormalities, with 30% of patients in whom parkinsonism or catatonia was ameliorated by antipsychotic drugs[55]. Likewise, in many studies of our own group and others we failed to detect a correlation between antipsychotic dosage and measures of PS[11, 26, 31, 49, 50]. However, symptoms tend to improve with treatment but from the naturalistic studies conducted it is currently unclear, whether the improvement is due to a direct effect on motor behavior or whether the benefit results from amelioration of other psychosis symptoms, such as disorganization or avolition[56, 57]. Our own study on the longitudinal course of PS in acute schizophrenia found an amelioration of PS with treatment, which was tightly linked to a decline of negative symptom scores[50]. Finally, there are no trials demonstrating a beneficial effect of antipsychotic medication or trainings to improve psychomotor slowing in schizophrenia. Thus, **alternative treatment options for PS are clearly needed**.

3.1.5 Psychomotor slowing and motor network dysfunction

Conceptually, various aspects of motor behavior are modulated by three key circuits: inhibition and excitation of movements is related to a circuit including pre-/motor cortex and basal ganglia, timing of movements is linked to another circuit including motor cortex, thalamus and cerebellum, while psychomotor speed and planning involves a cortical network including medial prefrontal cortex,

cingulate motor areas, SMA, M1, and posterior parietal cortex[12]. However, PS is not exclusively related to one of the abovementioned behaviors and probably involves all of the three circuits, even though the basal ganglia circuit is the most probable [22].

The current understanding of the pathology in PS is limited due to methodological issues, such as focus on single brain regions, single neuroimaging modalities, and heterogeneous patient samples[10, 22]. Two types of approaches have mainly been adopted: first, actigraphically assessed motor activity levels were correlated with structural and functional magnetic resonance imaging (MRI) markers in the brain. Results suggested that unlike in controls, motor activity levels are not associated with **structural connectivity within the basal ganglia loop** in schizophrenia. Instead, motor activity was linked to **cortical motor loops**, which was interpreted as compensatory mechanism because patients with PS (i.e. lower activity) had particularly lower CBF and reduced white matter integrity within these cortical motor loops[27, 58-60]. Likewise, white matter ultrastructure of the corpus callosum and cingulum mediated psychomotor speed in schizophrenia[61]. A second line of evidence stems from functional and structural MRI studies testing associations with finger movements such as finger tapping, sequential finger-thumb opposition, etc. These studies reported poorer tapping performance and reduced functional activation in SMA, M1, and cerebellum in schizophrenia[62]. Further evidence comes from the few studies on schizophrenia patients with catatonia, who usually present with behavioral motor inhibition. In a resting state perfusion MRI study, my group identified that catatonic schizophrenia is associated with specifically **increased cerebral blood flow (CBF)** in the supplementary motor area (**SMA**) and primary motor cortex (M1) in comparison to schizophrenia patients who had never experienced catatonia before. State CBF values were highest among those patients with severe motor inhibition. However, SMA perfusion was not different between state catatonia and controls[63]. The findings suggest a critical role of the SMA in movement initiation deficits in schizophrenia with state catatonia. However, from these data it was not possible to determine whether SMA hyperperfusion was the result of a motor network pathology that leads to inhibited motor output or whether SMA pathology drives this behavioral inhibition. Furthermore, finger tapping studies in catatonia patients indicated that M1 and SMA were less active during the task[64, 65]. Given the limitations of most previous studies focusing on one task or brain region, the next step must include **a network perspective to understand the pathobiology of motor inhibition as seen in PS**. A first cross-sectional study of my group applied resting state fMRI in order to test functional connectivity within the motor networks in schizophrenia. We found that schizophrenia was characterized by increased connectivity between key regions of the motor network, particularly between thalamus and motor/premotor cortex, M1 and cerebellum, cingulate seeds and STN[11]. Furthermore, the functional abnormalities between M1 and thalamus or cerebellum at rest were linked to observer ratings and instrumental measures of PS in schizophrenia. **Even though there is evidence suggesting that aberrant thalamocortical as well as cerebellar-cortical functional and structural connectivity may contribute to psychomotor slowing, the mechanism is still not entirely clear. This issue needs to be addressed at a network perspective and requires an exploration of the neural effects of interventions targeting psychomotor slowing.**

3.1.6 Intracortical excitability

Transcranial magnetic stimulation (TMS) allows for testing cortical neurophysiology in vivo. Converging evidence supports defective intracortical inhibition in M1 indexed by paired-pulse TMS in all stages of psychosis[66, 67]. Indeed, short-interval intracortical inhibition (SICI) is linked to GABAergic interneuron function. Reduced SICI values indicate **cortical disinhibition** in patients, which correlate with lower fractional anisotropy in motor tracts as well as lower processing speed[68]. Thus **psychomotor slowing** could be associated with **aberrant motor cortex excitability**, i.e. reduced SICI.

3.1.7 Brain stimulation for psychomotor slowing

Repetitive transcranial magnetic stimulation (rTMS) temporarily alters brain function in the targeted cortical areas. In addition, distant effects occur due to changes in network properties[69]. Depending on the frequency and type of stimulation, distinct effects on brain function are expected. Low-frequency rTMS (**lf-rTMS**) and continuous theta burst stimulation (cTBS) have **inhibitory** effects, while high-frequency rTMS (hf-rTMS) and intermittend theta burst (**iTBS**) have **facilitatory** effects. The preliminary knowledge of the pathobiology of PS suggests that rTMS could improve PS by changing aberrant motor network connectivity. Still, there are no published reports on the effects of non-invasive brain stimulation

on PS. However, my group is conducting a randomized, double-blind, sham-controlled trial of rTMS for psychomotor slowing in major depressive disorder and schizophrenia (**NCT03275766**, clinicaltrials.gov). Treatment is based on rTMS in 4 arms: 1) facilitatory hf-rTMS over the left dorsolateral prefrontal cortex (DLPFC), 2) inhibitory lf-rTMS over the supplementary motor area (SMA), 3) facilitatory iTBS over the SMA, and 4) sham stimulation with a placebo coil. The primary outcome parameter is the percentage of responders (> 30% reduction in the Salpêtrière Retardation Rating Scale (SRRS) from baseline). Across the whole group of 34 randomized subjects (22 with schizophrenia and 12 with depression), there is a group difference in the number of responders after 15 rTMS sessions applying the last-observation-carried-forward method ($Chi^2=11.3$, df=3, p=.007) with **78% responders under lf-rTMS over SMA and no responder under iTBS over SMA**. When we exclusively focus on schizophrenia, this effect is also detected ($Chi^2=6.6$, df=3). Thus, lf-rTMS over SMA ameliorates PS.

The neurophysiologic effects of rTMS treatment can be probed by perfusion MRI for local effects on metabolism, by fMRI on the network level and by TMS paradigms testing changes of cortical excitability of M1. Studies on inhibitory rTMS over the left SMA indicated short-term alterations of functional connectivity from SMA to M1 in healthy subjects along with changes of local metabolic activity in focal dystonia patients and controls[70-72]. While we don't know whether similar effects would occur in patients with altered baseline functional connectivity in the motor network, we may expect to see neural changes (functional connectivity and regional perfusion) following rTMS treatment. Furthermore, single **inhibitory rTMS** for 15 mins over the premotor cortex led to **increased motor cortical excitability** in schizophrenia evidenced by reduced resting motor thresholds and increased motor evoked potentials, supporting our clinical findings of an amelioration of PS[73].

3.1.8 State of research summary

Motor abnormalities are a core feature of psychotic disorders, particularly in the schizophrenia spectrum, indicating poorer outcome[10, 15, 74]. Motor abnormalities are linked to alterations within the cerebral motor networks[22]. One important domain is psychomotor slowing (PS) that impacts gross and fine motor behavior. PS causes distress and functional disability. Moreover, PS can be reliably assessed by instrumentation[15]. But the underlying neurobiology of PS is not well understood. Prior work from correlational studies reported PS to be linked to aberrant functional and structural connectivity between M1 and thalamus or cerebellum[11, 60]. Particularly, patients with severe PS display hyperconnectivity and hyperperfusion in the premotor and motor cortices. Currently, no treatments specifically target PS in schizophrenia. But preliminary data from an rTMS study of my group indicate that inhibitory rTMS over the SMA alleviates PS. In addition, one session of inhibitory rTMS over SMA altered motor cortex excitability in psychosis[73]. **Given, that the motor circuitry is associated with PS and that inhibitory rTMS over SMA may improve PS in schizophrenia, we expect that inhibitory rTMS alters the relevant network for PS in patients**. The combination of resting state fMRI, perfusion MRI, diffusion MRI and rTMS is particularly suited to explore the neural changes in the motor networks[72]. Therefore, we will conduct a prospective RCT of rTMS for PS with neuroimaging assessments at baseline and week 3, focusing on the motor network. Furthermore, we will explore the effects of rTMS on cortical excitability, clinical and functional outcome. This is the first project to enable causal inferences on the pathobiology of PS and further supporting the use of effective rTMS treatment in schizophrenia by **proof of principle**.

3.2 Investigational Product (treatment, device) and Indication

TMS device: MagPro 30 with theta burst option

Manufacturer: MagVenture, Inc. Atlanta GA, USA

Device is intended for a broad range of neuroscientific research in humans.

There is CE conformity according to the ISO Norm 13485:2003.

3.3 Preclinical Evidence

Low-frequency rTMS (1Hz) has been tested in many studies of human neurophysiology, typically as single session rTMS with transient behavioral effects. Lf-rTMS over the SMA alters functional connectivity in the motor system in healthy subjects[72]. Likewise, lf-rTMS over M1 demonstrated increased local neural activity with increasing stimulus intensity in healthy controls, in addition to distant changes in neural activity within M1 connections[75]. Finally, the cortical excitability is altered in healthy subjects following lf-rTMS of M1[76].

3.4 Clinical Evidence to Date

rTMS treatment for psychomotor slowing has only been tested in Parkinson's disease[77, 78] and in a combined group of patients with major depression and schizophrenia (Walther et al. unpublished data). In our own randomized double-blind placebo-controlled trial, 15 sessions of 1 Hz rTMS were effective in ameliorating psychomotor slowing (see also Background). The active comparator iTBS was not effective, but also well-tolerated. There were no exceptional side-effects beyond those regularly reported in studies of rTMS.

The intended protocols (1Hz rTMS and iTBS as active control) have been widely used in neuropsychiatric cohorts to treat various symptoms[2]. Low-frequency rTMS (1Hz) is effective in reducing the severity of auditory verbal hallucinations in schizophrenia spectrum disorders[79-86]. There have been numerous reports demonstrating safety and efficacy of this protocol. The active comparator iTBS has been tested in studies in major depressive disorder and is effective in reducing depression severity with minimal side effects[87, 88]; most commonly transient headaches[2]. rTMS treatment has been applied between 10 and 30 sessions, usually 5 per week. There are international guidelines on the use of rTMS for clinical purposes, including safety measures and side effect assessments[1, 2].

3.5 Dose Rationale / Medical Device: Rationale for the intended purpose in study (pre-market MD)

As outlined in sections 3.1.7., 3.1.8., 3.3., and 3.4. rTMS has been used to modify psychomotor slowing in Parkinson's disease, depression, and schizophrenia. In addition, rTMS over the premotor and motor cortex alters brain function. Our previous study demonstrated clinical effectiveness of 1 Hz stimulation over left SMA on psychomotor slowing in 15 sessions. There are no other treatment studies on motor function in schizophrenia. However, studies applying less than 10 stimulation sessions to treat hallucinations were less effective in schizophrenia spectrum disorders[82, 89]. In major depression, rTMS studies with similar protocols usually treat patients for 4-6 weeks (20-30 sessions)[87].

3.6 Explanation for choice of comparator (or placebo)

This study will have two comparators: one placebo stimulation and an active stimulation that produces the opposite effect of the investigated protocol, i.e. iTBS over SMA which will facilitate neural activity. Both comparators are necessary to demonstrate clinical utility (placebo) and the neurophysiological changes associated with the three treatments (placebo and iTBS). Only this approach will finally allow testing whether inhibitory lf-rTMS is changing the motor system in a specific direction that is causal for clinically relevant changes of motor behavior. The waiting list will disentangle specific stimulation effects from effects of time or expectation. Placebo will disentangle specific rTMS treatment effects. Finally, iTBS will disentangle the direction of neurophysiological changes of the motor system. All stimulations will target the left SMA. Mode of application, localization, frequency of sessions and apparatus will be identical for all protocol arms. Arms will differ in the coil used (real or sham) and in the frequency of the applied stimulation (1 Hz vs. iTBS). The number of pulses is similar in all protocols (1'000-1'200).

3.7 Risks / Benefits

The study intervention is adminstered as an add-on to existing treatment in inpatient (or less often dayclinic) settings. The study procedures pose some extra-effort and burden to the patients, particularly during baseline and week 3 assessments. However, the time and inconvenience is acceptable and comparable to those of other studies in the field. From our extensive experience with studies in these patient groups we know that assessments are acceptable and may be split to different days if needed. The neuroimaging with MRI twice has a total duration of less than 60 mins per session including the preparation. Most of the scanning time, participants will be in a resting state, i.e. have no task – just to relax lying in the scanner. This can be accomplished by many, even very ill subjects. Only a short proportion of the scanning time is devoted to a simple motor task in the scanner, when participants are asked to move their fingers. Taken together, both clinical and neuroimaging assessments are acceptable.

The study intervention (rTMS) has a minimal risk of increasing the severity of the disorder, i.e. deterioration. In our prior clinical rTMS trial with the same stimulation protocol and 15 rTMS sessions, there was no case of deterioration. Only one SAE occurred which was unrelated to the intervention. In addition, patients are under close observation by the study team and the hospital teams who see patients in the inpatient or dayclinic setting. Thus, changes are rapidly recognized and appropriate measures will be taken, as the study is only an add-on to standard treatment.

This study will follow the general recommendations for safety in rTMS protocols[1, 2] and the device manual. In addition, a screening standard questionnaire for rTMS candiates will be applied to ensure that all strict exclusion criteria for safety in the protocol will be respected. The expected main adverse effects could be transient headache, transient local pain, transient neck pain, transient toothache and

transient paraesthesia. Transient hearing changes have not been reported applying theta burst TMS but are likely possible. Therefore hearing protection will be used (earplugs). Furthermore we will make prompt referral for auditory assessment in case of any individual who complains of hearing loss, tinnitus or aural fullness following the rTMS. Seizure induction is rare but possible in epilepsy patients, which are therefore excluded. Furthermore, in some patients transient impairment of working memory was reported. Taken together, if the safety regulations are followed, the risk for participants is minimal. All adverse events are only temporarily occurring and no severe adverse events have been reported in rTMS trials up to now.

Magnetic fields attenuate rapidly with distance, so it seems unlikely that the fetus of a pregnant woman might be directly affected by rTMS. Currently, no side effects to the child were reported in reports of repeated rTMS in pregnant women[90, 91]. Still, pregnant women will be excluded from this study. Prior to study entry, a pregnancy test will be performed by all women in childbearing age, who are also asked to use a simple, effective contraceptive method during the 3 week trial.

The procedure of blood sampling involves the physician puncturing a vein in the groin or the bend of the elbow. Occasionally, a slight internal bleeding and the formation of a hematoma can occur, but usually disappears within a few days. Other risks such as nerve injury, thrombus formation and infection because of the punctuation, are very rare and with trained professionals almost excluded.

Participation in the study does not pose a particular risk for patients, as the rTMS will be added on to the existing standard medical and non-medical interventions. It is likely that patients in the experimental group will experience better outcomes. However, most patients (in control arms) and healthy controls will not have any personal benefit from participation in the study. Still, the study will be an important step towards the design of specific trials to improve psychomotor slowing in schizophrenia. Given, that the experimental arm was superior in ameliorating psychomotor slowing, treatment will result in better outcomes and quality of lives. In addition, results of this study will be used to plan further interventional trials in patients with schizophrenia, which may then introduce rTMS as a standard add-on treatment of psychomotor slowing in schizophrenia spectrum disorders.

3.8 Justification of choice of study population

This study will include a group of patients with schizophrenia spectrum disorders with symptoms of psychomotor slowing, who will receive the assessments and interventions. In addition, a group of healthy control subjects and a group of clinical control subjects is included only for the assessments, but not to receive an intervention. Patients will be included if they can provide consent on their own (no minors and no patients incapable of understanding the study information will be recruited). Participation in the study does not interfere with regular treatment. Patients' interests will be safeguarded by the treating physicians who are not part of the study team.

Study of patients is necessary in order to test the rTMS effect in a group with clear psychomotor slowing. These symptoms are only found in patients with severe psychiatric disorders.

Study of healthy controls is needed in order to compare motor behaviour abnormalities and alterations in brain network structure and function between health and disease. Healthy controls will not receive any intervention.

Study of clinical controls is needed in order to assess whether baseline findings in patients are associated specifically with psychomotor slowing or with schizophrenia in general. Clinical controls will not receive any intervention. The study procedures for this group are strictly cross-sectional with minimal discomfort for the participant, including procedures that are similar to the standard clinical work-up.

4. STUDY OBJECTIVES

4.1 Overall Objective

This study investigates the neural correlates of psychomotor slowing in schizophrenia spectrum disorders and the neurophysiologic changes associated with successful application of rTMS to ameliorate slowing. To reach this aim, the study combines a randomized, double-blind, placebo-controlled trial of rTMS in patients over 3 weeks and a cross-sectional and longitudinal study focussing on motor behaviour and brain imaging. Therefore, this study has four main aims. First, we aim to **investigate the clinical and functional neural changes following 3 weeks of daily rTMS treatment** (inhibitory, facilitatory, or placebo). An exploratory aim is to describe imaging markers of treatment response. Second, we aim to **characterize the pathophysiology of psychomotor slowing** (PS) in depth combining multimodal neuroimaging and electrophysiology with instrumental and observer-based measures of PS. Third, we aim **to characterize short-term changes of PS** by observing a waiting list cohort that is later allocated to treatment. And fourth, we aim to **describe the clinical outcome** of the rTMS intervention at 6-month follow-up.

4.2 Primary Objective

The study seeks to determine the clinical and neural effects of 15 sessions of inhibitory lf-rTMS over SMA on psychomotor slowing in schizophrenia spectrum disorders compared to facilitatory iTBS, placebo or patients on a waiting list.

4.3 Secondary Objectives

The study further seeks to determine, whether lf-rTMS (inhibitory) will be superior over placebo or iTBS (facilitatory) in all clinical and motor behavioral measures, e.g. greater reduction in BFCRS scores from baseline, greater increase in activity levels, or finger taps. In addition, the study will test the effects of rTMS on the cerebral motor network connectivity and activity, as well as on cerebellar function and motor cortex excitability. Furthermore, the study will explore the neuroimaging markers of response to treatment. Moreover, the study will test the association between measures of PS and neuroimaging markers of the motor system, e.g. network connectivity, activity, and structure. Next, the study will investigate the temporal dynamics of PS by comparing a waiting list group to the placebo group. Finally, the study will test whether lf-rTMS treatment for 3 weeks will have lasting effects on PS at week 6 or 6-months follow-up.

4.4 Safety Objectives

The study will also assess the tolerability of 15 of sessions rTMS in terms of stimulation side effects and duration of stimulation effects.

5. STUDY OUTCOMES

5.1 Primary Outcome

The primary outcome variable is the SRRS score as a surrogate marker of psychomotor slowing. The primary endpoint will be the change SRRS ratings from baseline to week 3 measured with the SRRS[92]. The SRRS is administered at baseline, week 1, week 2, week 3, week 6, and week 24. It is an observer rated scale quantifying the severity of psychomotor slowing with 15 items, each ranging 0-4. The SRRS has been developed to target this behavior and has been used in previous trials. Assessments will be performed by blinded raters, who have been trained to use the scale reliably.

5.2 Secondary Outcomes

5.2.1 Clinical outcomes

Proportion of responders (>= 30% reduction from baseline SRRS) between groups at week 1, 2, 3, 6, and 24. This will provide an additional categorical measure of who benefits from the intervention in terms of the main target (psychomotor slowing).

Change in other commonly observed motor symptoms from baseline, including rating scales on abnormal involuntary movements, Parkinsonism, and catatonia at week 3, 6, and 24. See section 9 for details on instruments.

Change in general psychopathology using the positive and negative syndrome scale PANSS[93] and the brief negative symptom scale BNSS[94] between baseline and week 3, week 6, and week 24.

Change in self-reported physical activity and experienced negative symptoms using the International Physical Activity Questionnaire (IPAQ)[95] and the self-evaluation of negative symptoms (SNS)[96].

5.2.2 Behavioral outcomes

Change in objective gross motor activity as measured with 24 hours of wrist actigraphy at baseline, week 3, 6, and 24.

Change in fine motor function as measured with the coin rotation task at baseline, week 3, 6, and 24.

Grip strength at baseline, week 3, 6, and 24.

5.2.3 Physiological outcomes

Changes in motor cortex excitability from baseline to week 3, 6, and 24 using a TMS paradigm of short interval intracortical inhibition (SICI), intracortical facilitation (ICF) and 1 mV motor evoked potentials (MEP).

Changes in postural sway from baseline at week 3, using the Kistler platform and assessments with eyes open as well as eyes closed.

5.2.4 Neuroimaging outcomes

Change in resting state perfusion within the motor network from baseline to week 3.

Change in motor network resting state connectivity from baseline to week 3.

Change in neural activation patterns during a finger tapping task using functional MRI at baseline and week 3.

5.3 Other Outcomes of Interest

Change in social and community functioning using the global assessment of functioning GAF[97], the Social and Occupational Functioning SOFAS[98], and the UPSA-brief[99] short assessment of functional capacity at baseline, week 6, and week 24. Social and community functioning is a relevant distal outcome for any psychiatric intervention. Future research questions may arise later, particularly, further analyses of the collected blood samples are possible.

5.4 Safety Outcomes

After each rTMS session, participants are inquired about stimulation side effects. After sessions 5, 10 and 15, we will apply a short rating scale to assess side effects according to the guidelines[1, 2].

6. STUDY DESIGN

6.1 General study design and justification of design

The design of this single-site study is a 4 parallel-arm, double-blind, randomized, placebo-controlled trial. Patients will be randomized to one of four treatment arms. Patients will all receive TMS over the left SMA in 15 daily sessions over 3 weeks as add-on treament. In three groups, assessments will be conducted immediately before and after the three weeks treatment (1 Hz, iTBS, and Placebo), while the fourth group will receive active treatment (1Hz) only after a 3 week waiting period with no additional intervention. After 6 months we will perform a clinical follow-up assessment. The interventions will be conducted by a small team (2 persons) who will know the stimulation parameters. All assessments will be conducted by a different team, who is completely blind to treatment and specifically trained to use the instruments and rating scales. Patients are also blind to treatment, because stimulation site is the same for all groups and there will be no change of protocols during the study. The placebo group will be stimulated with a placebo coil that looks identical and emits identical noises compared to the real TMS coil, but without any magnetic impulse emission. Assessments with clinical rating scales will be complemented by objective and instrumental motor tests that are not prone to rater bias. Therefore, both the patients and the assessors are blind to treatment protocol.

The study will include patients with schizophrenia spectrum disorders who currently have psychomotor slowing according to the SRRS. The protocol arms are chosen to test the clinical efficacy and neurophysiological changes of 15 sessions 1 Hz rTMS for psychomotor slowing in schizophrenia spectrum disorders. The placebo-arm is required to demonstrate an effect of rTMS as add-on treatment. The active control is required to demonstrate an opposite effect at the neural level compared to active treatment. Finally, the waiting list is important to characterize potential self-limitation of the condition under study. Because these subjects are referred to a waiting list, they will receive active treatment thereafter. This group will not be completely blinded, as they will know to be in the active treatment group from week 3 through week 6.

We intend to enrol 88 patients with schizophrenia spectrum disorders and psychomotor slowing. For the neuroimaging and motor behavioural assessments, we also plan to include 40 matched healthy control subjects and 25 matched clinical control subjects

For each patient in group 1-3 the study is 3 weeks and a follow-up interview after 6 months. Patients in group 4 (waiting list) will have three assessments (baseline, week 3 and week 6) and a follow-up interview at 6 months. The total duration of the study is 4 years.

6.2 Methods of minimising bias

6.2.1 Randomisation

Using the free software research randomizer, we will generate for the patients a list with four numbers indicating the group allocation, i.e. stimulation types. Allocation will be 1:1:1:1 across the whole intended sample. Subsequent numbers of study inclusion will therefore determine to which study arm the patient will be randomized. The lists will only be available for the principal investigator and locked in his office. He will perform randomization and give the written group allocation to the investigators.

6.2.2 Blinding procedures

As described in 6.1, patients will receive 15 sessions of rTMS over the left SMA in a blinded fashion. They will not be able to see which protocol is used (coil placement on top of their head, machine display behind the patients). Because of the parallel arm design, they will not know how the other protocols feel. Patients will be eye-blinded and ear-plugged during rTMS.

The teams performing assessments and rTMS stimulations will be strictly distinct. Therefore, patients and outcome assessors will be blinded to treatment arm.

6.2.3 Other methods of minimising bias

Outcomes are assessed with instrumental means or validated questionnaires. All assessments will follow a standard routine. Assessors will be trained to use instruments by the PI.

6.3 Unblinding Procedures (Code break)

In case of severe adverse events, an unblinding can take place at the responsibility of the principal investigator. In this case, the participant will not be able to further participate in the study. The allocated intervention order will be kept in a sealed envelope. The sealed envelopes will be stored at a central place, i.e. the office of the lab, so they can be accessed in case of any emergency.

7. STUDY POPULATION

The aim is to test 88 subjects with schizophrenia spectrum disorders and psychomotor slowing, 40 healthy control subjects, and 25 clinical control subjects. Both genders will be included, age range 18 – 65 years.

7.1 Eligibility criteria

Participants fulfilling all of the following **<u>inclusion</u>** criteria are eligible for the study:

- ages 18–60 years
- Right-handed subjects
- Ability and willingness to participate in the study
- Ability to provide written informed consent
- Informed Consent as documented by signature (Appendix Informed Consent Form)
- **Patients only**: schizophrenia spectrum disorders according to DSM-5 **with** psychomotor slowing (SRRS score ≥ 15)
- **Clinical controls only:** schizophrenia spectrum disorders according to DSM-5 **without** psychomotor slowing (SRRS score < 15)

The presence of any one of the following **<u>exclusion</u>** criteria will lead to exclusion of the participant:

- Substance abuse or dependence other than nicotine
- Past or current medical or neurological condition associated with impaired or aberrant movement, such as brain tumors, stroke, M. Parkinson, M. Huntington, dystonia, or severe head trauma with subsequent loss of conciousness.
- Epilepsy or other convulsions
- History of any hearing problems or ringing in the ears
- Standard exclusion criteria for MRI scanning and TMS; e.g. metal implants, claustrophobia
- Patients only: any TMS treatment in the past 3 months
- Women who are pregnant or breast feeding,
- Intention to become pregnant during the course of the study,
- Female participants who are surgically sterilised / hysterectomised or post-menopausal for longer than 2 years are not considered as being of child bearing potential.
- Previous enrolment into the current study,
- Enrolment of the investigator, his/her family members, employees and other dependent persons
- **Healthy controls only**: history of any psychiatric disorder or first-degree relatives with schizophrenia spectrum disorders.

7.2 Recruitment and screening

Healthy participants will be recruited by word-of-mouth, an internet link at the homepage of the department and flyers at supermarkets or at the University of Bern. Staff of the University Hospital of Psychiatry Bern will not be recruited. Furthermore, patients will be asked for participation at the inpatient and outpatient departments of the University Hospital of Psychiatry, Bern. All patients will spend approximately a total of 18 hours in the study on 7 different assessment days (screening, baseline, 3x during interventions and 2 follow-ups). Thus, they will be compensated by a single payment of CHF 200,-. Screening is described in section 9.1 and performed by master-level psychologists or psychiatrists.

7.3 Assignment to study groups

Randomization procedure is described in section 6.2.1. When the investigators have recruited a patient-participant, the principal investigator will allocate the person to a study group based on the randomization list and the sequence of enrolment in the study. From the list of numbers, patients are sequentially allocated by the principal investigator to one of the four treatment arms. The principal investigator will provide written information to the investigators concerning the treatment arm. The lists with the group allocations are only accessible for the principal investigator.

7.4 Criteria for withdrawal / discontinuation of participants

Participants may discontinue the trial at any time or withdraw consent. Furthermore, the treating physicians may request study discontinuation in case of significant deterioration of the condition at any time during the intervention period. All data from subjects in the intent-to-treat population, who received at least one dose of rTMS will be further used in the analyses. We will apply the last-observation-carried-forward (LOCF) method in the final analyses. In case a patient withdraws consent before the first rTMS stimulation, we will recruit an additional patient. The number of all patients recruited and randomized will be reported in the publications. All data from neuroimaging acquisition and motor behaviour will be used whenever possible, e.g. for analyses of baseline associations between brain imaging and behavior. In case of withdrawal due to adverse events or serious adverse events there will be follow-up examinations after 14 days and repeatedly until the problem is resolved, see 9.2.5.

8. STUDY INTERVENTION

8.1 Identity of Investigational Products (treatment / medical device)

8.1.1 Experimental Intervention (treatment / medical device)

Name: MagPro R30 with theta burst option

Manufacturer: MagVentrue, Inc. Atlanta GA, USA

The device is CE certified according to ISO-Norm 13485:2003 and approved for clinical use.

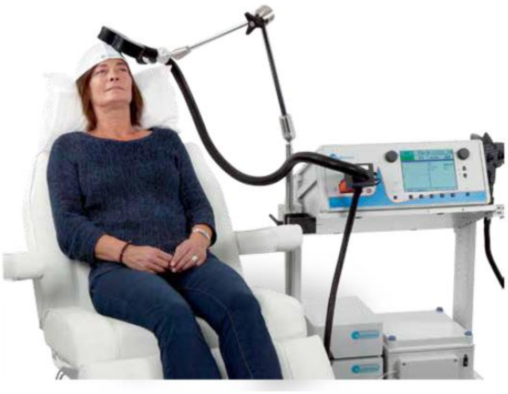

Active stimulation: 1 Hz stimulation of 17 mins over left SMA, 15 sessions in total (5 per week)

8.1.2 Control Intervention (standard/routine/comparator treatment / medical device)

All stimulations are delivered with the same device. The stimulation sites are the same (left SMA) for all groups. The iTBS protocol is shorter (3 mins), the placebo protocol will be delivered with a specific

placebo-coil that looks identical, makes identical sounds but produces no magnetic pulses.

Placebo control: 1 Hz stimulation of 17 mins over left SMA without magnetic emission, 15 sessions in total (5 per week)

Active control: iTBS stimulation over left SMA, 21 mins per session, 15 sessions in total (5 per week)

Waiting list: no intervention during the first three weeks, afterwards active stimulation: 1 Hz stimulation of 17 mins over left SMA, 15 sessions in total (5 per week)

8.1.3 Packaging, Labelling and Supply (re-supply)

Not applicable.

8.1.4 Storage Conditions

TMS devices are stored in the institution (translational research center at the University Hospital of Psychiatry, Bern) according to internal regulations and the device manual.

8.2 Administration of experimental and control interventions

8.2.1 Experimental Intervention

The application of the TMS device follows the published guidelines[1, 2] and the device manual. Before each session, the resting motor threshold is determined to identify the individual intensity of stimulation. The position for the coil placement is left SMA, which is determined either via neuronavigation using individual brain anatomy or 3 cm anterior of the leg motor area, which is individually determined through single pulse stimulations causing leg movements.

Both experimenter and participants will wear earplugs for auditory safety.

Active experimental protocol:

Lf-rTMS at 1Hz will be used with 1’000 pulses at an intensity of 110% of the resting motor threshold (approximate duration 17 minutes). The protocol is identical to that of our previous study and a study in Parkinson’s disease[77]. 15 sessions in total (5 per week)

8.2.2 Control Intervention

Placebo control: 1 Hz stimulation of 17 mins over left SMA without magnetic emission, using the Placebo-coil. 15 sessions in total (5 per week)

Active control: Stimulation parameters for iTBS will follow those of Huang et al.[100], including 600 pulses at 50 Hz (stimulation in 2 sec trains every 10 sec for 190 sec in total). To increase the effect, we will deliver two iTBS series in one session separated by 15 min with a total of 1200 pulses. During the experiment, iTBS pulse intensity is adjusted to 80% of the motor threshold. iTBS stimulation of 21 mins over left SMA, 15 sessions in total (5 per week)

Waiting list: same protocol as active experimental protocol but only between week 3 and 6.

8.3 Dose / Device modifications

In case of intolerable side effects, the study will be discontinued for the participant. No dose or device modifications are planned.

8.4 Compliance with study intervention

Investigators have to document the order of stimulation. This will be checked for consistency with the group allocation. No further strategies are needed. Participants are always under observation during the rTMS sessions and the planned assessments. Non-compliance on the side of the participant would lead to study discontinuation for this person. Use of concomitant medication will be retrieved from the medical files of the patients and transferred to the CRF.

8.5 Data Collection and Follow-up for withdrawn participants

If participants withdraw their consent, they will be contacted immediately to clarify whether there were intolerance issues with the study. See section 9.2.5 for safety follow-up measures. In case of withdrawal the data will be analyzed and stored encrypted as all the data of the other participants. However, we will remove the encryption key for the participants who withdrew consent and therefore it will not be possible to identify the subjects from encrypted data. Patients who withdrew consent will be invited for the 6 month follow-up interview using their preferred way of contact (Email, letter).

8.6 Trial specific preventive measures

No specific preventive measures are needed.

8.7 Concomitant Interventions (treatments)

Current medication will be recorded in the CRF at study entry and throughout the 3 week intervention period. Patients are expected to comply with the treatment they are receiving from their treating physicians.

Because this is an add-on treatment, we expect relevant changes from concomitant interventions. Therefore, we apply placebo, waiting-list, and an active control group.

8.8 Study Drug / Medical Device Accountability

Not applicable.

8.9 Return or Destruction of Study Drug / Medical Device

The TMS devices are already in use in the clinic. No study specific procedures are intended.

9. STUDY ASSESSMENTS

9.1 Study flow chart(s) / table of study procedures and assessments

Flow charts are distinct for healthy controls, clinical controls, and patients, because controls receive no intervention and psychopathology will only be assessed for clinical controls and patients. Furthermore, three patient groups will start interventions immediately (lf-rTMS, iTBS, and Placebo; i.e. groups 1-3), but one group will be waiting for 3 weeks and then enter the intervention phase (waiting group; i.e. group 4).

9.1.1 Patients of treatment groups 1-3

Study Periods	Screening	Treatment, Intervention Period				Follow-up	
Visit	1	2	3	4	5	6	7
Time (week)	-1	0	1	2	3	6	24
Patient Information and Informed Consent	x						
Demographics	x						
CASH and SCID (history)	x						
In- /Exclusion Criteria	x						
Physical Examination	x						
Pregnancy Test	x						
Psychopathology (TALD, NES)		x					
Randomisation		x					
Administer rTMS (5 sessions per week)		x	x	x			
Primary Variable SRRS		x	x	x	x	x	x
Motor scales UPDRS, BFCRS, AIMS, IPAQ		x			x	x	x
Psychopathology (PANSS, BNSS, SNS)		x			x	x	x
Actigraphy and coin rotation		x			x	x	x
Posturography		x			x		
TMS cortical excitability		x			x	x	x
Grip force measurement		x			x	x	x
Cerebral MRI		x			x		
Blood sampling		x			x		
Functional outcome (SOFAS, GAF, UPSA-brief)		x				x	x

Concomitant Therapy		x	x	x	x		
Adverse Events		x	x	x	x		

Study events patient groups 1-3

Visit	Information	Instrument used	Expected time
1 (Screening)	Check inclusion / exclusion criteria	CRF	5 min
	Demographic data	CRF	5 min
	Medical history	CRF	10 min
	Handedness	Edinburgh Handedness Inventory[101]	5 min
	Pregnancy test	Urine based test	10 min
	Diagnoses ascertainment	SCID interview	60 min
2 (baseline)	Psychopathology	Positive And Negative Syndrome Scale (PANSS)[93] interview, CASH[102] interview, Brief Negative Symptom Scale (BNSS)[94], Thought and Language Disorder (TALD)[103], Neurological Evaluation Scale (NES)[104]	90 min
	Primary outcome	SRRS[92]	5 min
	Motor scales	Unified Parkinson's Disease Rating Scale (UPDRS)[105], Bush Francis Catatonia Rating Scale (BFCRS)[106], Abnormal Involuntary Movement Scale (AIMS)[107]	15 min
	Self Report	Self-Evaluation of negative symptoms (SNS)[96], International Physical Actitvity Questionnaire (IPAQ)[95]	10 min
	Motor tests	Actigraphy and coin rotation	5 min
	Posturography	Kistler platform at Dept. of Neurology	30 min
	Cortical excitability	TMS paradigm with paired pulse stimulation	30 min
	Grip force measurement	Measure of the strength in each hand	5 min
	Neuroimaging	MRI scan including structural T1, Arterial spin labelling, BOLD resting state and task fMRI	60 min
	Blood sampling	Blood sampling	10 min
	Functional outcome	Global Assessment of Functioning GAF, Social and Occupational Functioning Scale SOFAS, UPSA-brief assessment of functional capacity	15 min
	rTMS	5 rTMS daily sessions à 20 min	100 min
3 (week 1)	Primary outcome	SRRS	5 min
	rTMS	5 rTMS daily sessions à 20 min	100 min
	Check side effects	CRF	5 min
	Concomitant therapy	CRF	5 min
4 (week 2)	Primary outcome	SRRS	5 min
	rTMS	5 rTMS daily sessions à 20 min	100 min

	Check side effects	CRF	5 min
	Concomitant therapy	CRF	5 min
5 (week 3)	Psychopathology	Positive And Negative Syndrome Scale (PANSS)[93] interview, Brief Negative Symptom Scale (BNSS)[94]	30 min
	Primary outcome	SRRS[92]	5 min
	Motor scales	Unified Parkinson's Disease Rating Scale (UPDRS)[105], Bush Francis Catatonia Rating Scale (BFCRS)[106], Abnormal Involuntary Movement Scale (AIMS)[107]	15 min
	Self Report	Self-Evaluation of negative symptoms (SNS)[96], International Physical Actitvity Questionnaire (IPAQ)[95]	10 min
	Motor tests	Actigraphy and coin rotation	5 min
	Posturography	Kistler platform at Dept. of Neurology	30 min
	Cortical excitability	TMS paradigm with paired pulse stimulation	30 min
	Grip force measurement	Measure of the strength in each hand	5 min
	Neuroimaging	MRI scan including structural T1, Arterial spin labelling, BOLD resting state and task fMRI	60 min
	Blood sampling	Blood sampling	10 min
	Functional outcome	Global Assessment of Functioning GAF, Social and Occupational Functioning Scale SOFAS, UPSA-brief assessment of functional capacity	15 min
	Check side effects	CRF	5 min
	Concomitant therapy	CRF	5 min
6 (week 6)	Psychopathology	Positive And Negative Syndrome Scale (PANSS)[93] interview, Brief Negative Symptom Scale (BNSS)[94]	30 min
	Primary outcome	SRRS[92]	5 min
	Motor scales	Unified Parkinson's Disease Rating Scale (UPDRS)[105], Bush Francis Catatonia Rating Scale (BFCRS)[106], Abnormal Involuntary Movement Scale (AIMS)[107]	15 min
	Self Report	Self-Evaluation of negative symptoms (SNS)[96], International Physical Actitvity Questionnaire (IPAQ)[95]	10 min
	Motor tests	Actigraphy and coin rotation	5 min
	Cortical excitability	TMS paradigm with paired pulse stimulation	30 min
	Grip force measurement	Measure of the strength in each hand	5 min
	Functional outcome	Global Assessment of Functioning GAF, Social and Occupational Functioning Scale SOFAS, UPSA-brief assessment of functional capacity	15 min
7 (week 24)	Psychopathology	Positive And Negative Syndrome Scale (PANSS)[93] interview, Brief Negative Symptom Scale (BNSS)[94]	30 min
	Primary outcome	SRRS[92]	5 min

	Motor scales	Unified Parkinson's Disease Rating Scale (UPDRS)[105], Bush Francis Catatonia Rating Scale (BFCRS)[106], Abnormal Involuntary Movement Scale (AIMS)[107]	15 min
	Self Report	Self-Evaluation of negative symptoms (SNS)[96], International Physical Actitvity Questionnaire (IPAQ)[95]	10 min
	Motor tests	Actigraphy and coin rotation	5 min
	Cortical excitability	TMS paradigm with paired pulse stimulation	30 min
	Grip force measurement	Measure of the strength in each hand	5 min
	Functional outcome	Global Assessment of Functioning GAF, Social and Occupational Functioning Scale SOFAS, UPSA-brief assessment of functional capacity	15 min

9.1.2 Patients of treatment group 4 (waiting group)

Study Periods	Screening	Treatment, Intervention Period					Follow-up	
Visit	1	2	3	4	5	6	7	8
Time (week)	-1	0	3	4	5	6	9	24
Patient Information and Informed Consent	x							
Demographics	x							
CASH and SCID (history)	x							
In- /Exclusion Criteria	x							
Physical Examination	x							
Pregnancy Test	x							
Psychopathology (TALD, NES)		x						
Randomisation		x						
Administer rTMS (5 sessions per week)			x	x	x			
Primary Variable SRRS		x	x	x	x	x	x	x
Motor scales UPDRS, BFCRS, AIMS, IPAQ		x	x			x	x	x
Psychopathology (PANSS, BNSS, SNS)		x	x			x	x	x
Actigraphy and coin rotation		x	x			x	x	x
Posturography		x	x			x		
TMS cortical excitability		x	x			x	x	x
Grip force measurement		x	x			x	x	x
Cerebral MRI		x	x			x		
Blood sampling		x				x		
Functional outcome (SOFAS, GAF, UPSA-brief)		x	x				x	x
Concomitant Therapy		x	x	x	x	x		
Adverse Events			x	x	x	x		

Study events for patients of the waiting group

Visit	Information	Instrument used	Expected time
1 (Screening)	Check inclusion / exclusion criteria	CRF	5 min
	Demographic data	CRF	5 min
	Medical history	CRF	10 min
	Handedness	Edinburgh Handedness Inventory[101]	5 min
	Pregnancy test	Urine based test	10 min
	Diagnoses ascertainment	SCID interview	60 min
2 (baseline)	Psychopathology	Positive And Negative Syndrome Scale (PANSS)[93] interview, CASH[102] interview, Brief Negative Symptom Scale (BNSS)[94], Thought and Language Disorder (TALD)[103], Neurological Evaluation Scale (NES)[104]	90 min
	Primary outcome	SRRS[92]	5 min
	Motor scales	Unified Parkinson's Disease Rating Scale (UPDRS)[105], Bush Francis Catatonia Rating Scale (BFCRS)[106], Abnormal Involuntary Movement Scale (AIMS)[107]	15 min
	Self Report	Self-Evaluation of negative symptoms (SNS)[96], International Physical Actitvity Questionnaire (IPAQ)[95]	10 min
	Motor tests	Actigraphy and coin rotation	5 min
	Posturography	Kistler platform at Dept. of Neurology	30 min
	Cortical excitability	TMS paradigm with paired pulse stimulation	30 min
	Grip force measurement	Measure of the strength in each hand	5 min
	Neuroimaging	MRI scan including structural T1, Arterial spin labelling, BOLD resting state and task fMRI	60 min
	Blood sampling	Blood sampling	10 min
	Functional outcome	Global Assessment of Functioning GAF, Social and Occupational Functioning Scale SOFAS, UPSA-brief assessment of functional capacity	15 min
3 (week 3)	Psychopathology	Positive And Negative Syndrome Scale (PANSS)[93] interview, CASH[102] interview present part only, Brief Negative Symptom Scale (BNSS)[94], Thought and Language Disorder (TALD)[103], Neurological Evaluation Scale (NES)[104]	90 min
	Primary outcome	SRRS[92]	5 min
	Motor scales	Unified Parkinson's Disease Rating Scale (UPDRS)[105], Bush Francis Catatonia Rating Scale (BFCRS)[106], Abnormal Involuntary Movement Scale (AIMS)[107]	15 min

	Self Report	Self-Evaluation of negative symptoms (SNS)[96], International Physical Actitvity Questionnaire (IPAQ)[95]	10 min
	Motor tests	Actigraphy and coin rotation	5 min
	Posturography	Kistler platform at Dept. of Neurology	30 min
	Cortical excitability	TMS paradigm with paired pulse stimulation	30 min
	Grip force measurement	Measure of the strength in each hand	5 min
	Neuroimaging	MRI scan including structural T1, Arterial spin labelling, BOLD resting state and task fMRI	60 min
	Blood sampling	Blood sampling	10 min
	Functional outcome	Global Assessment of Functioning GAF, Social and Occupational Functioning Scale SOFAS, UPSA-brief assessment of functional capacity	15 min
	rTMS	5 rTMS daily sessions à 20 min	100 min
4 (week 4)	Primary outcome	SRRS	5 min
	rTMS	5 rTMS daily sessions à 20 min	100 min
	Check side effects	CRF	5 min
	Concomitant therapy	CRF	5 min
5 (week 5)	Primary outcome	SRRS	5 min
	rTMS	5 rTMS daily sessions à 20 min	100 min
	Check side effects	CRF	5 min
	Concomitant therapy	CRF	5 min
6 (week 6)	Psychopathology	Positive And Negative Syndrome Scale (PANSS)[93] interview, Brief Negative Symptom Scale (BNSS)[94]	30 min
	Primary outcome	SRRS[92]	5 min
	Motor scales	Unified Parkinson's Disease Rating Scale (UPDRS)[105], Bush Francis Catatonia Rating Scale (BFCRS)[106], Abnormal Involuntary Movement Scale (AIMS)[107]	15 min
	Self Report	Self-Evaluation of negative symptoms (SNS)[96], International Physical Actitvity Questionnaire (IPAQ)[95]	10 min
	Motor tests	Actigraphy and coin rotation	5 min
	Posturography	Kistler platform at Dept. of Neurology	30 min
	Cortical excitability	TMS paradigm with paired pulse stimulation	30 min
	Grip force measurement	Measure of the strength in each hand	5 min
	Neuroimaging	MRI scan including structural T1, Arterial spin labelling, BOLD resting state and task fMRI	60 min
	Blood sampling	Blood sampling	10 min
	Functional outcome	Global Assessment of Functioning GAF, Social and Occupational Functioning	15 min

		Scale SOFAS, UPSA-brief assessment of functional capacity	
	Check side effects	CRF	5 min
	Concomitant therapy	CRF	5 min
7 (week 9)	Psychopathology	Positive And Negative Syndrome Scale (PANSS)[93] interview, Brief Negative Symptom Scale (BNSS)[94]	30 min
	Primary outcome	SRRS[92]	5 min
	Motor scales	Unified Parkinson's Disease Rating Scale (UPDRS)[105], Bush Francis Catatonia Rating Scale (BFCRS)[106], Abnormal Involuntary Movement Scale (AIMS)[107]	15 min
	Self Report	Self-Evaluation of negative symptoms (SNS)[96], International Physical Actitvity Questionnaire (IPAQ)[95]	10 min
	Motor tests	Actigraphy and coin rotation	5 min
	Cortical excitability	TMS paradigm with paired pulse stimulation	30 min
	Grip force measurement	Measure of the strength in each hand	5 min
	Functional outcome	Global Assessment of Functioning GAF, Social and Occupational Functioning Scale SOFAS, UPSA-brief assessment of functional capacity	15 min
8 (week 24)	Psychopathology	Positive And Negative Syndrome Scale (PANSS)[93] interview, Brief Negative Symptom Scale (BNSS)[94]	30 min
	Primary outcome	SRRS[92]	5 min
	Motor scales	Unified Parkinson's Disease Rating Scale (UPDRS)[105], Bush Francis Catatonia Rating Scale (BFCRS)[106], Abnormal Involuntary Movement Scale (AIMS)[107]	15 min
	Self Report	Self-Evaluation of negative symptoms (SNS)[96], International Physical Actitvity Questionnaire (IPAQ)[95]	10 min
	Motor tests	Actigraphy and coin rotation	5 min
	Cortical excitability	TMS paradigm with paired pulse stimulation	30 min
	Grip force measurement	Measure of the strength in each hand	5 min
	Functional outcome	Global Assessment of Functioning GAF, Social and Occupational Functioning Scale SOFAS, UPSA-brief assessment of functional capacity	15 min

9.1.3 Healthy control subjects

Study Periods	Screening	Observation period	
Visit<br>Time (week)	1<br>-1	2<br>0	3<br>3
Proband Information and Informed Consent	x		
Demographics	x		
SCID	x		
In- /Exclusion Criteria	x		
Physical Examination	x		
Pregnancy Test	x		
Primary Variable SRRS		x	x
Motor scales UPDRS, BFCRS, AIMS, IPAQ		x	x
Actigraphy and coin rotation		x	x
Posturography		x	x
TMS cortical excitability		x	x
Grip force measurement		x	x
Cerebral MRI		x	x
Blood sampling		x	x

Study events for healthy controls

**Visit**	**Information**	**Instrument used**	**Expected time**
1 (Screening)	Check inclusion / exclusion criteria	CRF	5 min
	Demographic data	CRF	5 min
	Medical history	CRF	10 min
	Handedness	Edinburgh Handedness Inventory[101]	5 min
	Pregnancy test	Urine based test	10 min
	Diagnoses ascertainment	SCID interview	60 min
2 (week 0)	Primary outcome	SRRS[92]	5 min
	Motor scales	Unified Parkinson’s Disease Rating Scale (UPDRS)[105], Bush Francis Catatonia Rating Scale (BFCRS)[106], Abnormal Involuntary Movement Scale (AIMS)[107]	15 min
	Self Report	International Physical Actitvity Questionnaire (IPAQ)[95]	5 min
	Motor tests	Actigraphy and coin rotation	5 min
	Posturography	Kistler platform at Dept. of Neurology	30 min
	Cortical excitability	TMS paradigm with paired pulse stimulation	30 min
	Grip force measurement	Measure of the strength in each hand	5 min
	Neuroimaging	MRI scan including structural T1, Arterial spin labelling, BOLD resting state and task fMRI	60 min

	Blood sampling	Blood sampling	10 min
3 (week 3)	Primary outcome	SRRS[92]	5 min
	Motor scales	Unified Parkinson's Disease Rating Scale (UPDRS)[105], Bush Francis Catatonia Rating Scale (BFCRS)[106], Abnormal Involuntary Movement Scale (AIMS)[107]	15 min
	Self Report	International Physical Activtity Questionnaire (IPAQ)[95]	5 min
	Motor tests	Actigraphy and coin rotation	5 min
	Posturography	Kistler platform at Dept. of Neurology	30 min
	Cortical excitability	TMS paradigm with paired pulse stimulation	30 min
	Grip force measurement	Measure of the strength in each hand	5 min
	Neuroimaging	MRI scan including structural T1, Arterial spin labelling, BOLD resting state and task fMRI	60 min
	Blood sampling	Blood sampling	10 min

9.1.4 Clinical control subjects

Study Periods	Screening	Assessment day
Visit<br>Time (week)	1<br>-1	2<br>0
Patient Information and Informed Consent	x	
Demographics	x	
SCID	x	
In- /Exclusion Criteria	x	
Physical Examination	x	
Pregnancy Test	x	
Psychopathology (TALD. NES)		x
Primary Variable SRRS		x
Motor scales UPDRS, BFCRS, AIMS, IPAQ		x
Psychopathology (PANSS, BNSS, SNS)		x
Actigraphy and coin rotation		x
Posturography		x
TMS cortical excitability		x
Grip force measurement		x
Cerebral MRI		x
Blood sampling		x
Functional outcome (SOFAS, GAF, UPSA-brief)		x
Concomitant Therapy		x

Study events for clinical controls

Visit	Information	Instrument used	Expected time
1 (Screening)	Check inclusion / exclusion criteria	CRF	5 min
	Demographic data	CRF	5 min
	Medical history	CRF	10 min
	Handedness	Edinburgh Handedness Inventory[101]	5 min
	Pregnancy test	Urine based test	10 min
	Diagnoses ascertainment	SCID interview	60 min
2 (week 0)	Psychopathology	Positive And Negative Syndrome Scale (PANSS)[93] interview, CASH[102] interview, Brief Negative Symptom Scale (BNSS)[94], Thought and Language Disorder (TALD)[103], Neurological Evaluation Scale (NES)[104]	90 min
	Primary outcome	SRRS[92]	5 min
	Motor scales	Unified Parkinson's Disease Rating Scale (UPDRS)[105], Bush Francis Catatonia Rating Scale (BFCRS)[106], Abnormal Involuntary Movement Scale (AIMS)[107]	15 min
	Self Report	Self-Evaluation of negative symptoms (SNS)[96], International Physical Actitvity Questionnaire (IPAQ)[95]	10 min
	Motor tests	Actigraphy and coin rotation	5 min
	Posturography	Kistler platform at Dept. of Neurology	30 min
	Cortical excitability	TMS paradigm with paired pulse stimulation	30 min
	Neuroimaging	MRI scan including structural T1, Arterial spin labelling, BOLD resting state and task fMRI	60 min
	Functional outcome	Global Assessment of Functioning GAF, Social and Occupational Functioning Scale SOFAS, UPSA-brief assessment of functional capacity	15 min
	Concomitant therapy	CRF	5 min

9.2 Assessments of outcomes

Assessors of outcome variables will be psychologists or psychiatrists of master-degree-level. All assessors will be trained by the PI to use the instruments correctly and to assure interrater reliability. Weekly staff meetings will ensure conformity in procedures. All assessments will be conducted blind to rTMS treatment.

9.2.1 Assessment of primary outcome

The Salpêtrière Retardation Rating Scale (SRRS) is applied at each visit from baseline to follow-up. The scale is described in section 5.1. Raters will be trained to use the scale and blind to rTMS treatment. The score of each of the 15 items is recorded in the CRF.

9.2.2 Assessment of secondary outcomes

9.2.2.1 Clinical outcomes

The change in motor syndromes from baseline is assessed at several visits (see 9.1). Trained raters

blind to treatment will assess motor behaviour in standardized examinations with clinical rating scales. Parkinsonism is assess with the Unified Parkinson's Disease Rating Scale (UPDRS)[105], of which we will only assess part III, the current motor behaviour. Catatonia will be assessed with the Bush Francis Catatonia Rating Scale (BFCRS)[106], an 24 item rating scale specifically designed for this purpose. The timeframe of observation is the last week. Finally, dyskinesia will be monitored with the standard rating scale for this issue, the Abnormal Involuntary Movement Scale (AIMS)[107], a 7 item scale following a standardized examination. All motor rating scales are the gold standard of their kind.

The change of general schizophrenia symptoms is assessed with the Positive And Negative Syndrome Scale (PANSS)[93]. The 30 item scale is the widely used standard assessment following a standardized clinical interview. Change in negative symptoms is specifically assessed with the Brief Negative Symptom Scale (BNSS)[94], which allows monitoring relevant dimensions of negative symptoms such as apathy and diminished expression. Furthermore, we will apply the self-evaluation of negative symptoms (SNS)[96], a valid and reliable 20 items questionnaire to capture the subjective experience of negative symptoms.

9.2.2.2 Behavioral outcomes

Objective gross motor behaviour will be assessed using continuous wrist actigraphy for 24 hours. The actigraphs (empathica e4) will be worn on the wrist of the non-dominant arm. Data is stored as logged electronic file. This measure is sensitive to altered motor behaviour and has been successfully applied in many studies of Prof. Walther's team[27, 35, 49, 50, 108-111]. Patients will fill a sleep activity protocol to enable separation of sleep from wake periods during recording. This also allows to check data for consistency and plausibility, because measurements are continuously performed also in periods when participants are not observed.

The fine motor performance is assessed with the coin rotation task. In this task, participants are asked to rotate a .50 CHF coin between thumb, index and middle finger as fast as possible for a total of 30 seconds. The performance is recorded on video and later analysed offline. Analysis includes the number of half turns and the number of coin drops according to a validated formula[51, 52, 112].

Self-ratings of physical activity will be conducted with the 7-item International Physical Activity Questionnaire (IPAQ)[95], which has a German Version and has great psychometric properties. The ratings cover the past 7 days and allow calculating the energy expenditure and total activity.

9.2.2.3 Physiological outcomes

Measures of cortical excitability will be assessed as one of the important physiological outcome parameters. Measurements will be conducted with a MagPro R30 (MagVentrue, Inc. Atlanta GA, USA). Single pulse and paired pulse TMS protocols will be conducted to measure short-interval intracortical inhibition (SICI) at 1 msec interstimulus interval (ISI) and 3msec ISI, intracortical facilitation (ICF) at 7 msec ISI and 15 ms ISI, resting motor threshold (RMT), and 1 mV motor evoked potential (MEP), according to standard protocols[67, 113]

Posturography will be conducted at the Department of Neurology (Prof. Roger Kalla). The assessments of postural sway will be scheduled immediately before or after the MRI acquisition, because this is also located at the Inselspital Bern. Mean postural sway will be calculated as outcome variable for postural stability[53, 114]. The Kistler platform will be used to calculate of the pressure dependent fluctuation (x-, y-, z- axis) of the bodies' centre of gravity[115]. Participants will be measured standing with eyes open and eyes closed according to standard procedures.

9.2.2.4 Neuroimaging outcomes

Neuroimaging will be acquired twice, at baseline and week 3. MRI acquisition will be performed at a 3T Siemens Magnetom Trio scanner at the Institute of Diagnostic and Interventional Neuroradiology, Bern (local collaborator Prof. Dr. med. Roland Wiest). The 64-channel head coil will be used for all MRI images. First, high-resolution T1-weighted MR images will be obtained using a 3D magnetization-prepared rapid two-gradient-echo with 2 inversion times (MP2RAGE) sequence. fMRI will be performed using a multi-slice multi-band T2*-weighted echo planar imaging (EPI) sequence for resting state and during task execution. Prior to the fMRI images a B0 will be acquired for corrections of putative field inhomogeneity. Afterwards, we will perform acquisition of cerebral blood flow (CBF) at rest using a pseudo-continuous arterial spin labelling (pCASL) sequence. Moreover, we will acquire a M0 image (M0 equilibrium magnetization of water signal) for the quantification of CBF and a B0 for corrections of putative field inhomogeneity. Finally, a set of diffusion-weighted images (DWI) that will allow reconstructing fiber tracts using the model based on diffusion tensor imaging (DTI).

Anatomy (MP2RAGE) The optimized acquisition parameters were as follows: 176 sagittal slices, 256 × 224 matrix (with a non-cubic field of view (FOV) of 256 × 224 mm2, yielding a nominal isotropic resolution of 1 mm3), 5000 ms repetition time (TR), 2.98 ms echo time (TE), 700 ms and 2500 ms

inversion time (TI), flip angle 4° and 5°, GRAPPA acceleration factor 3 and a 8:22 min total acquisition time.

B0-map - The 48 EPI interleaved axial oblique slices will be positioned exactly like the fMRI slices with exactly the same slice geometry. Two amplitude and a phase image will be recorded in each subject (TR = 520 ms, TE1 = 4.92 ms, TE2 = 7.38 ms). The acquisition time will last 1 min 40 sec.

fMRI - The 48 EPI interleaved axial oblique slices will be positioned in-line with the bi-commissural axis with the following parameters: TR = 500 ms, TE = 30 ms, FA = 90, slice thickness = 3.6 mm, gap thickness = 0 mm, matrix size = 64 x 64 mm, FOV 230 x 230 mm2 resulting in a iso-voxel dimension of 3.6 mm x 3.6 mm x 3.6 mm. The sequence is driven in a 3D PACE mode (Siemens Erlangen) to enable prospective motion correction. With these sequence parameters we will cover the whole brain including the cerebellum. In total, first 720 dynamic scans will be collected for resting state fMRI (total of 6 mins) and subsequently 1200 dynamic scans will be collected during the conduction of the task (total of 10 mins). The task will consist of four blocks including two active paced blocks, a passive listening block and rest. All blocks except rest will be paced with a continuous 2Hz auditory cue. The two active blocks are **right hand finger tapping** (index finger vs. thumb) and **sequential finger-thumb opposition** (all fingers of the right hand vs. thumb). These tasks have produced reliable activation of SMA, M1, basal ganglia, and cerebellum in healthy subjects and are easy enough to be performed by schizophrenia patients with PS[62, 64, 65, 116, 117]. During one run, we will alternate active and passive blocks, i.e. finger tapping – listening – sequential finger opposition – rest. Each block will last for 15 s and each run takes 1 min. We will alternate the active blocks between runs. A total of 10 runs will be conducted. Instructions will be presented on a video goggle system. Cue tones will be delivered via headphones. Participants will be instructed and trained on the task outside the scanner.

pCASL - The 30 EPI interleaved axial oblique slices will be positioned in-line with the bi-commissural axis and acquired in sequential order with the following parameters: TR = 3000 ms, TE = 12 ms, FA = 90, slice thickness = 8.0 mm, gap thickness = 0 mm, matrix size = 64 x 64 mm, FOV 256 x 256 mm2 resulting in a voxel dimension of 4.0 mm x 4.0 mm x 8.0 mm. the sequence will additionally have the following parameters: bolus duration = 700 ms, inversion time = 2200 ms. In total 100 images will be acquired lasting in total 6 min.

9.2.3 Assessment of other outcomes of interest

Measures of social and community functioning as described in section 5.3.

9.2.4 Assessment of safety outcomes

9.2.4.1 Adverse events

After every rTMS session, participants are asked about new occurrence of adverse events. As example, they are inquired about sensations associated with the stimulation or headaches. The answers are recorded in the CRF. Furthermore, at each study visit during the intervention phase, i.e. after 5 rTMS sessions, patients will be inquired about adverse events using a standard questionnaire in the CRF[2].

In case the adverse event is not limited to stimulation, the participants will be asked at the next session (24 hours later), whether the adverse event still continues or when it was resolved. The frequency and type of adverse events will be reported in the publication of results.

9.2.4.2 Laboratory parameters

Blood sampling will be taken in a 4.9ml S-Monovette and will be send to and stored at the Center for Psychiatric Neuroscience, Department of Psychiatry, Lausanne University Hospital, Lausanne, Switzerland (Responsible analyzer Prof. Ph.D. Kim Do, Director of the Center of Psychiatric Neuroscience, phone: 021 643 69 49) for potential future analysis.

9.2.4.3 Vital signs

No routine measurement of vital signs is planned. If patients report dizziness, clinical routines will include measurement of blood pressure and heart rate.

9.2.5 Assessments in participants who prematurely stop the study

Participants who prematurely stop the study will be immediately assessed for adverse events. In case of adverse events reports, a physical examination will be conducted and results will be recorded accordingly. Follow-up examinations will be planned within the next 14 days in case of continuing problem related to adverse events, and these examinations will be continued every 14 days until the health issue is resolved.

9.3 Procedures at each visit

9.3.1 Screening visit

- Check the inclusion and exclusion criteria, use CRF
- Collect demographic data, use CRF
- Conduct urine pregnancy test in female participants between 18 and 50 years of age
- Assess diagnoses with SCID, document results in CRF
- Assess handedness with EHI[101], document result in CRF
- Assess medical history in CRF

See table study events at 9.1

9.3.2 Visit 2 (baseline, week 0)

- Assess current psychopathology, use PANSS interview and BNSS, document in CRF
- Assess formal thought disorder, use TALD interview, document in CRF
- Assess Neurological soft signs, use NES, document in CRF
- Assess psychiatric history with CASH interview in patients, document result in CRF
- Assess primary outcome with SRRS scale, document in CRF
- Assess motor behaviour with rating scales, use BFCRS, UPDRS, AIMS, document results in CRF
- Assess functioning, use GAF, SOFAS, and UPSA-brief, document results in CRF
- Hand out self-rating questionnaires SNS and IPAQ, collect and document in CRF
- Conduct coin rotation test, record on video
- Record gross motor activity using actigraphy
- Test cortical excitability using the TMS device according to standard procedure. Record electronic data
- Grip force measurement , document result in CRF
- Blood sampling
- Acquire MRI scans at University Institute of Diagnostic and Interventional Neuroradiology
- Acquire posturography at University Hospital, Dept. of Neurology, record electronic data
- Record current medication regime in CRF
- Schedule 5 rTMS sessions
- Schedule next visit

9.3.3 Visit 3 and 4 (week 1 and 2)

- Assess primary outcome with SRRS scale, document in CRF
- Assess side effects with questions in the CRF
- Record current medication regime in CRF
- Schedule 5 rTMS sessions
- Schedule next visit

9.3.4 Visit 5 (end of intervention, week 3)

- Assess side effects with questions in the CRF
- Assess current psychopathology, use PANSS interview and BNSS, document in CRF
- Assess primary outcome with SRRS scale, document in CRF
- Assess motor behaviour with rating scales, use BFCRS, UPDRS, AIMS, document results in CRF
- Assess functioning, use GAF, SOFAS, and UPSA-brief, document results in CRF
- Hand out self-rating questionnaires SNS and IPAQ, collect and document in CRF
- Conduct coin rotation test, record on video
- Record gross motor activity using actigraphy
- Test cortical excitability using the TMS device according to standard procedure. Record electronic data
- Grip force measurement , document result in CRF
- Blood sampling
- Acquire MRI scans at University Institute of Diagnostic and Interventional Neuroradiology
- Acquire posturography at University Hospital, Dept. of Neurology, record electronic data
- Record current medication regime in CRF

- Schedule next visit (follow-up)

9.3.5 Visit 6 and 7 (follow-up, week 6 and 24)

- Assess current psychopathology, use PANSS interview and BNSS, document in CRF
- Assess primary outcome with SRRS scale, document in CRF
- Assess motor behaviour with rating scales, use BFCRS, UPDRS, AIMS, document results in CRF
- Assess functioning, use GAF, SOFAS, and UPSA-brief, document results in CRF
- Hand out self-rating questionnaires SNS and IPAQ, collect and document in CRF
- Conduct coin rotation test, record on video
- Record gross motor activity using actigraphy
- Test cortical excitability using the TMS device according to standard procedure. Record electronic data
- Grip force measurement, document result in CRF
- Record current medication regime in CRF
- Schedule next visit (follow-up)

10. SAFETY

10.1 Medical Device Category A studies

Device deficiencies and all **adverse events (AE)** including all **serious adverse events (SAE)** are collected, fully investigated and documented in the source document and appropriate case report form (CRF) during the entire study period, i.e. from patient's informed consent until the last protocol-specific procedure, including a safety follow-up period. Documentation includes dates of event, treatment, resolution, assessment of seriousness and causal relationship to device and/or study procedure [ISO 14155, 6.4.1.].

Safety is assessed spontaneously at each stimulation session and in a structured set of questions at each visit during the intervention phase (after 5 stimulations). See 9.2.4. If adverse events occur, patients are followed-up with extra-visits after 14 days until the issue has resolved.

Typical adverse events to be expected by rTMS are:

- Mild pain or nausea (39 %)
- Mild headaches (28%)
- Mild neck pain (40%)

Rare events include hearing problems or local skin irritation.

10.1.1 Definition and Assessment of safety related events

Adverse Event (AE)

Any untoward medical occurrence, unintended disease or injury or any untoward clinical signs (including an abnormal laboratory finding) in participants, users or other persons whether or not related to the investigational medical device [ISO 14155: 3.2].

This includes events related to the investigational device or the comparator and to the procedures involved. For users or other persons this is restricted to events related to the investigational medical device.

Adverse Device Effect (ADE)

Adverse event related to the use of an investigational medical device [ISO 14155: 3.1].

This includes any adverse event resulting from insufficient or inadequate instructions for use, deployment, implantation, installation, operation, or any malfunction of the investigational medical device. This includes any event that is a result of a use error or intentional misuse.

Serious Adverse Event (SAE) [European regulation on medical devices 2017/745, art. 58].

Any adverse event that led to any of the following:

(a) death,
(b) serious deterioration in the health of the subject that resulted in any of the following:

(i) life-threatening illness or injury,
(ii) permanent impairment of a body structure or a body function,
(iii) hospitalisation or prolongation of patient hospitalisation,
(iv) medical or surgical intervention to prevent life-threatening illness or injury or permanent impairment to a body structure or a body function,
(v) chronic disease,

(c) foetal distress, foetal death or a congenital physical or mental impairment or birth defect.

This includes device deficiencies that might have led to a serious adverse event if a) suitable action had not been taken or b) intervention had not been made or c) if circumstances had been less fortunate. These are submitted to the EC via BASEC within 7 days. A planned hospitalisation for pre-existing condition, or a procedure required by the protocol, without a serious deterioration in health, is not considered to be a serious adverse event.

Device deficiency

Inadequacy of a medical device related to its identity, quality, durability, reliability, safety or performance, such as malfunction, misuse or use error and inadequate labelling [ISO 14155: 3.15].

Health hazards that require measures

Findings in the trial that may affect the safety of study participants and, which require preventive or corrective measures intended to protect the health and safety of study participants SAE [ClinO Art. 37].

Causal Relationship of SAE [MEDDEV 2.7/3 revision 3, May 2015].

A causal relationship towards the medical device or study procedure should be rated as follows:

- **Not related:** The relationship to the device or procedures can be excluded.
- **Unlikely:** The relationship with the use of the device seems not relevant and/or the event can be reasonably explained by another cause, but additional information may be obtained.
- **Possible:** The relationship with the use of the investigational device is weak but cannot be ruled out completely. Alternative causes are also possible.
- **Probable:** The relationship with the use of the investigational device seems relevant and/or the event cannot reasonably explained by another cause.
- **Causal relationship:** The serious event is associated with the investigational device or with procedures beyond reasonable doubt.

Device deficiencies that might have led to an SAE are always related to the medical device.

10.1.2 Reporting of (Serious) Adverse Events and other safety related events

Reporting to Sponsor-Investigator:

The following events are to be reported to the Sponsor-Investigator within 24 hours upon becoming aware of the event:

- All SAEs
- Health hazards that require measures
- Device deficiencies

The Sponsor-Investigator will evaluate SAEs with regard to causality and seriousness. Device deficiencies are assessed regarding their potential to lead to an SAE.

Pregnancies

Because pregnancy tests are a prerequisite to participation and the intervention is only 3 weeks, pregnancies will not be further assessed or reported.

Reporting to Authorities [ClinO Art. 42]:

In Category A studies, the sponsor is subject to the notification requirements specified in Art. 15 of the MedDO of 17 October 2011 (SR 812.213).

It is the Investigator's responsibility to report to the Ethics Committee via BASEC **device deficiencies** that could have led to serious adverse events if suitable action had not been taken, intervention had not

been made, or circumstances had been less fortunate within 7 days [ClinO Art. 42].

Health hazards that require measures are reported to the Ethics Committee via BASEC within 2 days [ClinO Art. 37].

Periodic safety reporting:

A yearly safety update-report is submitted by the Investigator to the Ethics Committee via BASEC.

10.1.3 Follow up of (Serious) Adverse Events

If adverse events occur, patients are followed-up with extra-visits after 14 days until the issue has resolved. This applies to patients who continue with the study but also to patients who prematurely exit the study. In case subjects are lost to followed-up, study personnel will contact the treating psychiatrists (or if unavailable, the general practitioners) in order to inform on serious adverse events and the recommendations for follow-up clinical examinations. In cases of headaches for example, care would include the prescription of a non-steroidal antirheumatic drug, i.e. pain medicine, according to the person's preferences, medical history, or interaction with existing medication.

11. STATISTICAL METHODS

A two-tailed p-value of < .05 is considered to be statistically significant in all analyses. We will also report effect sizes for the comparisons of primary and clinical secondary outcome variables between groups.

11.1 Hypotheses

Aim 1: investigate the clinical and functional neural changes following 15 sessions of daily rTMS

Hypothesis 1a (main hypothesis): lf-rTMS (inhibitory) will be superior over placebo or iTBS (facilitatory) in all clinical and motor behavioral measures, e.g. greater reduction in SRRS scores from baseline, greater increase in activity levels, or coin rotations.

Hypothesis 1b: lf-rTMS will reduce aberrant functional connectivity in the motor system, e.g. between thalamus and M1. lf-rTMS will alter regional CBF in the motor system and increase SMA and M1 activity during the fMRI task. No relevant changes are expected in the sham group, iTBS may deteriorate neural alterations.

Hypothesis 1c: cortical excitability will be differentially changed with rTMS. Lf-rTMS will increase SICI, while iTBS will reduce SICI.

Hypothesis 1d: at Visit 5 (end of intervention, week 3) there will be changes in blood markers of neural integrity and neuroinflammation with treatment (exploratory).

Aim 2: characterization of psychomotor slowing (PS) in schizophrenia spectrum disorders compared to healthy controls and to clinical controls (schizophrenia without PS)

Hypothesis 2a: Patients with PS will have poorer performance on all motor tasks, eg. reduced activity levels, increased postural sway, and less coin rotations compared to healthy and clinical controls

Hypothesis 2b: Patients with PS will have aberrant structural and functional connectivity within the motor system, as well as increased CBF in basal ganglia and decreased CBF in premotor/motor cortex in comparison to healthy controls. Clinical controls will hold an intermediate position between schizophrenia with PS and healthy controls. Applying network metrics, the functional motor network will be less efficient in schizophrenia.

Hypothesis 2c: Behavioral measures of PS will be associated with aberrant motor network structure, perfusion, function, and connectivity. For example, patients with strong PS will have increased resting state functional connectivity between thalamus and M1. PS severity will be linked to reduced structural motor network efficiency, lower M1 and SMA activity during the fMRI task, and increased SMA perfusion at rest.

Hypothesis 2d: Patients with PS will have increased motor cortex excitability (e.g. reduced SICI), in comparison to healthy controls. Clinical controls will hold an intermediate position between schizophrenia with PS and healthy controls. Motor cortex excitability will be linked to measures of PS.

Aim 3 characterize short term dynamics of PS

Hypothesis 3a: in very few subjects we expect relevant spontaneous improvements in PS measures. The majority will have less than 20% fluctuation of motor parameters within 3 weeks

Hypothesis 3b: we expect slight longitudinal changes in resting state functional connectivity and perfusion of the motor system, but no structural changes.

Aim 4: to describe the short-term and medium-term clinical outcome of 3 weeks of rTMS intervention

Hypothesis 4a: patients receiving inhibitory rTMS will have better outcome (less PS, less general symptom severity) shortly after the intervention (at 6 week follow-up) compared to patients receiving placebo or facilitatory iTBS

Hypothesis 4b: patients receiving inhibitory rTMS will have better outcome (less PS, less general symptom severity) and superior function (social and global) at 6-month follow-up compared to patients receiving placebo or facilitatory iTBS

Hypothesis 4c (exploratory): changes in functional connectivity within the motor system from baseline to week 3 will predict better outcome at week 6, particularly reduced M1-thalamus functional connectivity.

11.2 Determination of Sample Size

The main effects of the 3-week interventions on PS (SRRS scores) will be calculated in a repeated measures design including 2 measures (Baseline, week 3) and four groups (lf-rTMS, iTBS, sham, waiting group-lf-rTMS). Assuming a moderate effect size (f = 0.23) as indicated by our pilot data in a repeated measures ANOVA with moderate correlation between time points (0.5), a power of 0.95 and an alpha = 0.05, we would need 88 patients (22 per group).

Cross-sectional comparison at baseline between the three groups (patients with PS, healthy controls, and clinical controls) will be tested using one way ANOVAs expecting a moderate to large effect size (f = 0.30) with an alpha-level of 0.05 and a power of 0.90 when including 144 subjects. As we aim for 88 patients and 40 controls, we would need an additional 16 patients without psychomotor slowing. However, this group would be too small for some comparisons. Therefore, we aim for 25 patients as clinical controls.

11.3 Statistical criteria of termination of trial

No statistical stopping rules are planned.

11.4 Planned Analyses

11.4.1 Datasets to be analysed, analysis populations

All data from subjects in the intent-to-treat population, who received at least one dose of rTMS will be used in the analyses of rTMS effects. We will conduct sensitivity analyses in all trial-completers (see 11.5).

In the analyses on biological and clinical correlates of PS, we will use all data of the baseline assessment, thus including data from subjects who dropped-out prior to the first rTMS session.

11.4.2 Primary Analysis

The primary analysis will be a repeated measures ANOVA of SRRS scores with two timepoints (within subjects: baseline and week 3) and four groups (lf-rTMS, iTBS, sham, waiting group-lf-rTMS). In case of significant baseline demographic or clinical group differences, we will adjust for these in an repeated measures ANCOVA of SRRS.

The analysis will be performed by the PI and his team in SPSS using data from the RedCap database.

11.4.3 Secondary Analyses

Secondary analyses focus on the secondary outcomes (see 5.2) and hypotheses (see 11.1).

Crosstabs are used to calculate the proportion of responders (>=30% reduction in SRRS from baseline) across groups. Repeated measures ANOVAs will clarify the time-course of SRRS change from baseline to week 6 (including week 1, 2, 3 and 6) and differences between groups.

Repeated measures ANOVAs will test the change of clinical and motor rating scales between baseline and week 3, week 6, and week 24 and differences between groups.

Linear regression analyses will test the association between neuroimaging markers (resting state perfusion, connectivity, fMRI activation, ect.) and measures of PS at baseline.

Repeated measures ANOVAs will test the longitudinal changes in neuroimaging markers from baseline to week 3.

We will explore changes in blood markers of neural integrity and neuroinflammation from baseline to week 3.

All analyses will be performed by the PI and his team or collaborators with permission of the PI.

11.4.4 Interim analyses

Interim analyses of the primary analysis are planned after enrolment of 10, 20, 30, 40, 50, 60, and 70 subjects. The scope is to estimate whether any adjustments of the treatment arms are necessary. In addition, baseline data can be tested for associations between neuroimaging data and clinical/motor measures after enrolment of 20, 40, or 60 subjects.

All interim analyses are optional and at the discretion of the PI. Interim analyses are conducted by the PI and his team.

11.4.5 Safety analysis

The study team will provide a descriptive analysis of all reported adverse events. The analysis will focus on the type of event, duration, and timing and relate the number of observed events to the number of rTMS sessions administered. Furthermore, the frequency of adverse events will be compared between treatment arms of the trial.

11.4.6 Deviation(s) from the original statistical plan

Any deviation from the statistical plan will be reported and justified in the methods of the study report.

11.5 Handling of missing data and drop-outs

We will apply the last-observation-carried-forward (LOCF) method in the final analyses. In case a patient withdraws consent before the first rTMS stimulation, we will recruit an additional patient. The number of all patients recruited and randomized will be reported in the publications.

Sensitivity analyses will be performed in order to establish whether premature dropout would influence the results. Therefore, the primary analyses will be repeated with all subjects who completed the interventional trial. Further tests will establish whether drop-outs differ from completers in basic clinical or demographic variables.

12. QUALITY ASSURANCE AND CONTROL

All study personnel will be trained by the PI to achieve consistent adherence to procedures and interrater reliability. Standard operating procedures will be written for the intervention and standardized TMS measurements. For all clinical rating scales, instructions and manuals are provided for the study personnel. All study personnel will have to read the manuals.

12.1 Data handling and record keeping / archiving

12.1.1 Case Report Forms

All relevant data is documented in paper CRFs. This data includes inclusion/exclusion criteria, demographic data, medical history, scores of clinical and motor rating scales. Furthermore, side effects, concomitant treatment, reasons for discontinuation will be recorded in the CRF. Timing and conductance of experimental measures, such as posturography, cerebral MRI, cortical excitability, and actigraphy will be recorded recorded in the CRF. However, the real data of these measures is stored electronically outside the CRF.

For each enrolled study participant a CRF is maintained. CRFs will be kept current to reflect subject status at each phase during the course of study. Participants will be coded by use of a participant number in combination with the year of birth.

The paper forms of rating scales (e.g. PANSS) and the electronic data (e.g. MRI, posturography, ect.) are considered source documents. Furthermore, in patients the medical record of our hospital is a source document for the past psychiatric history and current concomitant treatment. All other data (participant history, side effects, ect.) are directly entered into the CRF without further source.

The study personnel is authorized to enter data into the CRFs, the name of the entering person will be documented. CRF data will be recorded in an electronic database, i.e. RedCap-Database. The procedure will include data cross-check between electronic and paper CRF. Any deviation will be corrected by consensus and consulting the CRF forms. RedCap-Database has individualized logins for each member of the study team and provides logs for data entry.

The electronic database for the non-CRF data will be logged via a secure Dropbox folder, thus the

person entering the data or changing data can be identified by individualized logins. The log also identifies the document changes and the time of the change. Dropbox allows for restoring each version of the documents within the study folder. A copy of the electronic neuroimaging data will be shared with Collaborator Prof. Jessica Bernard, College Station, TX, USA. At her laboratory, specific neuroimaging analyses will be performed with a subset of the electronic data. The original electronic files will be stored in Bern (see 12.1.3), while the specific neuroimaging analyses data of Prof. Bernard will be stored encrypted at her university for 10 years.

12.1.2 Specification of source documents

As described in 12.1.1. most data in the CRFs is source data. Furthermore, Informed consent form, specific rating scales and electronic data (such as MRI, actigraphy or posturography) are source data. In patients, the medical record of the hospital may contain source data on the psychiatric history. All CRFs and paper source data will be kept in folders. Any extra examination in case of additional safety assessments will be documented on paper and kept as source data in the same folders as CRF and other source data.

12.1.3 Record keeping / archiving

All study data will be archived for 10 years after study termination or premature termination of the trial at the translational research center of the University Hospital of Psychiatry, Bern. In addition, the data of specific neuroimaging analyses performed at Prof. Bernard's laboratory, will be stored encrypted for 10 years at Texas A & M University, College Station, TX, USA. Thus, source data are kept in Bern, and all further handled data will be stored under identical conditions in Bern or Texas Station.

12.2 Data management

Data of all participants will be encrypted and analysis will be performed using encrypted data only. Unblinding of participant-specific data is only possible after consent has been given by the participants and the sponsor. Data of all participants will receive a numerical identification. The key to this encryption code will be stored in a single file at the address below, and it will be accessible only by the sponsor and the principal investigator.

Office of the PI: University of Bern, University Hospital of Psychiatry, Murtenstrasse 21, 3008 Bern, Office 01-132

12.2.1 Data Management System

Describe what system (also software) is being used and who is responsible and how it is tested before the trial (may include a description of where the system is hosted).

12.2.1.1 <u>Neuroimaging data</u>

MRI data are anonymized digital data of appr. 1 GB per subject and scan, we will aim for 303 scans, i.e. total of 300 GB. MRI data sets include the dicom files of the planned sequences fMRI task, fMRI rest, ASL rest, DTI and T1 anatomy. MRI data sets will be safely stored in encrypted formats at the server of the translational research center, university hospital in Bern. Data will be logged with personal logins, such that any change to the files is reproducible. Data analysis will be performed locally in computers of the institute's network. Some analyses are planned to be performed on encrypted data at computers of the study collaborator Prof. Bernard. All files generated during data processing and analysis will be stored. Total data volume is approximately 2 TB. Neuroimaging data will be stored at a dropbox-folder, which is encrypted by Boxcryptor Software and owned by the Translational Research Center, University Hospital of Psychiatry, Bern. The system has been effectively used in several studies. Access to the folder and files is secured by personal login and password. The log of the Dropbox folder enables the identification of data entry/data change according to the logins used. The log will inform on which data has been changed when by whom. Dropbox allows for restoration of each file version, thus each change can be tracked.

12.2.1.2 <u>motor behavioral/instrumental data</u>

Data from actigraphy, posturography, TMS-MEP experiments as well as video recordings of coin rotation will be stored encrypted in digital format. As neuroimaging data, storage will comply with the federal human research act and will be conducted at standard facilities of the translational research center with encrypted and logged server storage. Data access is granted upon personal logins, such that any change to the files is reproducible. Each dataset includes several measures across 4-5 time points for one study participant. We expect appr. 1 GB per subject, total of 130 GB.

12.2.1.3 <u>clinical and demographic data</u>

Clinical data including outcome measures with observer based rating scales or patients' history will be

collected. This data is transferred to paper case report forms and will later be anonymously transferred to a red cap data base, hosted by the CTU Bern. Data access is only available to study staff with personalized logins. During the longitudinal assessments approximately 300 variables will be assessed for each participant.

12.2.1.4 <u>blood sampling data</u>

Genetic blood samples will be stored, managed and controlled in Center for Psychiatric Neuroscience, Department of Psychiatry, Lausanne University Hospital, Lausanne following strictly the current Swiss legal requirements for data protection and will be performed according to the Ordinance HRO Art. 5.

12.2.2 Data security, access and back-up

Only the PI and authorized personnel with own logins and passwords will have access to the electronic data. The translational research center Bern runs a daily backup of the dropbox folders. The RedCap Database also has regular backups.

12.2.3 Analysis and archiving

All analyses will be performed with a final copy of the SPSS file including all clinical data. All other analyses (neuroimaging, posturography, actigraphy) will be conducted with the appropriate standard software. Only the PI and authorized personnel will have access to the data to perform the analyses.

12.2.4 Electronic and central data validation

Data will be checked for consistency, e.g. range checks for questionnaire scores or test scores, checks for date entries temporal consistency.

12.3 Monitoring

The principal investigator will check the CRFs for completeness and internal consistency (plausibility of information) and external consistency (with source data) after each participant completed the study. In addition an external monitoring will be performed by Dr. Peter.

Four monitoring dates are planned. The first before the study recruitment starts, the second after the first three participants have been included, the third after 50% of enrolment was achieved and the final monitoring is planned after the last data had been collected.

Before first recruitment: Check whether all study assessments and instruments are ready and complete.

After the first three participants: Check CRF entries and compliance with source data where possible. Clarify queries of data entry or study procedures.

After the 50% enrolment: Check whether CRFs of the all collected cases are completed and data is consistent.

After the final data collection: Check whether CRFs of the all cases collected after the last monitoring are completed and data is consistent.

12.4 Audits and Inspections

Not intended for this single center trial.

12.5 Confidentiality, Data Protection

All data will be handled strictly confidential. Direct access to the source documents will be permitted only for purposes of inspections (ICHE6, 6.10) by authorities such as the CEC or monitoring by the PI. Only the PI and his team will have access to the protocol, dataset and other study related information during and after the study.

12.6 Storage of biological material and related health data

Coded genetic blood samples will be stored, managed and controlled in Center for Psychiatric Neuroscience, Department of Psychiatry, Lausanne University Hospital, Lausanne following strictly the current Swiss legal requirements for data protection and will be performed according to the Ordinance HRO Art. 5. All electronic data will be archived for 10 years (see 12.1).

Data sharing outside of the project is currently not planned. However, electronic and biological data, particularly neuroimaging electronic data and blood samples, may be later shared with national or international collaborators to address research questions that are currently unknown. The data storage must comply with the same regulations as the data storage of this study. Video data will not be shared

with other groups.

13. PUBLICATION AND DISSEMINATION POLICY

Publication of results is the responsibility of the sponsor/investigator. The results of the study will be prepared for publication in peer-reviewed scientific journals, preferably as open-access articles. Authorship will follow the national guidelines. Furthermore, results will be reported at scientific conferences as posters or oral communications. The use of professional writers is not intended. After scientific publication, the results will be prepared for communication in lay man's terms in the media.

Data sharing outside of the project is currently not planned. However, electronic data, particularly neuroimaging electronic data, may be later shared with national or international collaborators to address research questions that are currently unknown. The data storage must comply with the same regulations as the data storage of this study.

14. FUNDING AND SUPPORT

14.1 Funding

This study receives project funding by the Swiss National Science Foundation (grant #182469 to Prof. Sebastian Walther). Additional minor costs will be covered by the University Hospital of Psychiatry in Bern.

14.2 Other Support

Consumables and further material support is provided by the host institution, the Translational Research Center of the University Hospital of Psychiatry, University of Bern, Switzerland.

15. INSURANCE

No special insurance due to category A.

16. REFERENCES

1. Rossi S, Hallett M, Rossini PM, Pascual-Leone A, Safety of TMSCG. Safety, ethical considerations, and application guidelines for the use of transcranial magnetic stimulation in clinical practice and research. Clin Neurophysiol 2009;120:2008-2039.
2. Lefaucheur JP, Andre-Obadia N, Antal A, et al. Evidence-based guidelines on the therapeutic use of repetitive transcranial magnetic stimulation (rTMS). Clin Neurophysiol 2014;125:2150-2206.
3. Owen MJ, Sawa A, Mortensen PB. Schizophrenia. Lancet 2016;388:86-97.
4. Howes OD, Murray RM. Schizophrenia: an integrated sociodevelopmental-cognitive model. Lancet 2014;383:1677-1687.
5. Tandon R, Nasrallah HA, Keshavan MS. Schizophrenia, "just the facts" 4. Clinical features and conceptualization. Schizophr Res 2009;110:1-23.
6. Yang GJ, Murray JD, Wang XJ, et al. Functional hierarchy underlies preferential connectivity disturbances in schizophrenia. Proc Natl Acad Sci U S A 2016;113:E219-228.
7. van den Heuvel MP, Sporns O, Collin G, et al. Abnormal rich club organization and functional brain dynamics in schizophrenia. JAMA Psychiatry 2013;70:783-792.
8. Klauser P, Baker ST, Cropley VL, et al. White Matter Disruptions in Schizophrenia Are Spatially Widespread and Topologically Converge on Brain Network Hubs. Schizophr Bull 2016.
9. Friston KJ. Schizophrenia and the disconnection hypothesis. Acta Psychiatr Scand Suppl 1999;395:68-79.
10. Walther S, Mittal VA. Motor System Pathology in Psychosis. Curr Psychiatry Rep 2017;19:97.
11. Walther S, Stegmayer K, Federspiel A, Bohlhalter S, Wiest R, Viher PV. Aberrant Hyperconnectivity in the Motor System at Rest Is Linked to Motor Abnormalities in Schizophrenia Spectrum Disorders. Schizophr Bull 2017;43:982-992.
12. Mittal V, Bernard JA, Northoff G. What can Different Motor Circuits Tell Us About Psychosis? An

RDoC Perspective. Schizophr Bull 2017.

13. Kindler J, Schultze-Lutter F, Michel C, et al. Abnormal involuntary movements are linked to psychosis-risk in children and adolescents: Results of a population-based study. Schizophr Res 2016;174:58-64.

14. Mittal VA, Neumann C, Saczawa M, Walker EF. Longitudinal progression of movement abnormalities in relation to psychotic symptoms in adolescents at high risk of schizophrenia. Arch Gen Psychiatry 2008;65:165-171.

15. van Harten PN, Walther S, Kent JS, Sponheim SR, Mittal VA. The clinical and prognostic value of motor abnormalities in psychosis, and the importance of instrumental assessment. Neurosci Biobehav Rev 2017;80:476-487.

16. Peralta V, Campos MS, De Jalon EG, Cuesta MJ. Motor behavior abnormalities in drug-naive patients with schizophrenia spectrum disorders. Mov Disord 2010;25:1068-1076.

17. Putzhammer A, Perfahl M, Pfeiff L, Hajak G. Correlation of subjective well-being in schizophrenic patients with gait parameters, expert-rated motor disturbances, and psychopathological status. Pharmacopsychiatry 2005;38:132-138.

18. Kao YC, Liu YP, Chou MK, Cheng TH. Subjective quality of life in patients with chronic schizophrenia: relationships between psychosocial and clinical characteristics. Compr Psychiatry 2011;52:171-180.

19. Mittal VA. Cross-Cutting Advancements Usher in a New Era for Motor Research in Psychosis. Schizophr Bull 2016;42:1322-1325.

20. Walther S, Stegmayer K, Sulzbacher J, et al. Nonverbal social communication and gesture control in schizophrenia. Schizophr Bull 2015;41:338-345.

21. Walther S, Eisenhardt S, Bohlhalter S, et al. Gesture Performance in Schizophrenia Predicts Functional Outcome After 6 Months. Schizophrenia Bull 2016;42:1326-1333.

22. Walther S. Psychomotor symptoms of schizophrenia map on the cerebral motor circuit. Psychiatry Res 2015;233:293-298.

23. Morrens M, Hulstijn W, Sabbe B. Psychomotor slowing in schizophrenia. Schizophr Bull 2007;33:1038-1053.

24. Walther S, Strik W. Motor Symptoms and Schizophrenia. Neuropsychobiology 2012;66:77-92.

25. Morrens M, Docx L, Walther S. Beyond boundaries: in search of an integrative view on motor symptoms in schizophrenia. Front Psychiatr 2014;5:145.

26. Docx L, Sabbe B, Provinciael P, Merckx N, Morrens M. Quantitative psychomotor dysfunction in schizophrenia: a loss of drive, impaired movement execution or both? Neuropsychobiology 2013;68:221-227.

27. Walther S, Federspiel A, Horn H, et al. Alterations of white matter integrity related to motor activity in schizophrenia. Neurobiol Dis 2011;42:276-283.

28. Wichniak A, Skowerska A, Chojnacka-Wojtowicz J, et al. Actigraphic monitoring of activity and rest in schizophrenic patients treated with olanzapine or risperidone. J Psychiatr Res 2011;45:1381-1386.

29. Putzhammer A, Heindl B, Broll K, Pfeiff L, Perfahl M, Hajak G. Spatial and temporal parameters of gait disturbances in schizophrenic patients. Schizophr Res 2004;69:159-166.

30. Lisi G, Nico D, Ribolsi M, et al. Asymmetries in initiation of aiming movements in schizophrenia. Neuropsychologia 2018;109:200-207.

31. Docx L, Morrens M, Bervoets C, et al. Parsing the components of the psychomotor syndrome in schizophrenia. Acta Psychiatr Scand 2012;126:256-265.

32. Morrens M, Hulstijn W, Van Hecke J, Peuskens J, Sabbe BG. Sensorimotor and cognitive slowing in schizophrenia as measured by the Symbol Digit Substitution Test. J Psychiatr Res 2006;40:200-206.

33. Fervaha G, Agid O, Takeuchi H, et al. Extrapyramidal symptoms and cognitive test performance in patients with schizophrenia. Schizophr Res 2015;161:351-356.

34. Peralta V, Cuesta MJ. Neuromotor abnormalities in neuroleptic-naive psychotic patients: antecedents, clinical correlates, and prediction of treatment response. Compr Psychiatry 2011;52:139-145.

35. Walther S, Stegmayer K, Horn H, Razavi N, Muller TJ, Strik W. Physical Activity in Schizophrenia is Higher in the First Episode than in Subsequent Ones. Front Psychiatry 2014;5:191.

36. Cuesta MJ, Sanchez-Torres AM, de Jalon EG, et al. Spontaneous parkinsonism is associated

with cognitive impairment in antipsychotic-naive patients with first-episode psychosis: a 6-month follow-up study. Schizophr Bull 2014;40:1164-1173.

37. Grootens KP, Vermeeren L, Verkes RJ, et al. Psychomotor planning is deficient in recent-onset schizophrenia. Schizophr Res 2009;107:294-302.

38. Mittal VA, Gupta T, Orr JM, et al. Physical activity level and medial temporal health in youth at ultra high-risk for psychosis. J Abnorm Psychol 2013;122:1101-1110.

39. Dean DJ, Mittal VA. Spontaneous parkinsonisms and striatal impairment in neuroleptic free youth at ultrahigh risk for psychosis. NPJ Schizophr 2015;1.

40. Niendam TA, Bearden CE, Johnson JK, et al. Neurocognitive performance and functional disability in the psychosis prodrome. Schizophr Res 2006;84:100-111.

41. Stubbs B, Ku PW, Chung MS, Chen LJ. Relationship Between Objectively Measured Sedentary Behavior and Cognitive Performance in Patients With Schizophrenia Vs Controls. Schizophr Bull 2017;43:566-574.

42. Stubbs B, Chen LJ, Chung MS, Ku PW. Physical activity ameliorates the association between sedentary behavior and cardiometabolic risk among inpatients with schizophrenia: A comparison versus controls using accelerometry. Compr Psychiatry 2017;74:144-150.

43. Martin-Sierra A, Vancampfort D, Probst M, et al. Walking capacity is associated with health related quality of life and physical activity level in patients with schizophrenia: a preliminary report. Actas Esp Psiquiatr 2011;39:211-216.

44. Gur RC, Ragland JD, Moberg PJ, et al. Computerized neurocognitive scanning: II. The profile of schizophrenia. Neuropsychopharmacology 2001;25:777-788.

45. Lee RS, Hermens DF, Naismith SL, et al. Neuropsychological and functional outcomes in recent-onset major depression, bipolar disorder and schizophrenia-spectrum disorders: a longitudinal cohort study. Transl Psychiatry 2015;5:e555.

46. Boden R, Abrahamsson T, Holm G, Borg J. Psychomotor and cognitive deficits as predictors of 5-year outcome in first-episode schizophrenia. Nord J Psychiatry 2014;68:282-288.

47. Tohen M, Khalsa HM, Salvatore P, et al. The McLean-Harvard First-Episode Project: Early Course in 114 Cases of First-Episode Nonaffective Psychoses. J Clin Psychiatry 2016;77:781-788.

48. Walther S, Koschorke P, Horn H, Strik W. Objectively measured motor activity in schizophrenia challenges the validity of expert ratings. Psychiatry Res 2009;169:187-190.

49. Walther S, Ramseyer F, Horn H, Strik W, Tschacher W. Less structured movement patterns predict severity of positive syndrome, excitement, and disorganization. Schizophr Bull 2014;40:585-591.

50. Walther S, Stegmayer K, Horn H, et al. The Longitudinal Course of Gross Motor Activity in Schizophrenia - Within and between Episodes. Front Psychiatry 2015;6:10.

51. Gebhardt A, Vanbellingen T, Baronti F, Kersten B, Bohlhalter S. Poor dopaminergic response of impaired dexterity in Parkinson's disease: Bradykinesia or limb kinetic apraxia? Mov Disord 2008;23:1701-1706.

52. Walther S, Vanbellingen T, Muri R, Strik W, Bohlhalter S. Impaired pantomime in schizophrenia: Association with frontal lobe function. Cortex 2013;49:520-527.

53. Kent JS, Hong SL, Bolbecker AR, et al. Motor deficits in schizophrenia quantified by nonlinear analysis of postural sway. PLoS One 2012;7:e41808.

54. Matsuura Y, Fujino H, Hashimoto R, et al. Standing postural instability in patients with schizophrenia: Relationships with psychiatric symptoms, anxiety, and the use of neuroleptic medications. Gait Posture 2015;41:847-851.

55. Peralta V, Cuesta MJ. The effect of antipsychotic medication on neuromotor abnormalities in neuroleptic-naive nonaffective psychotic patients: a naturalistic study with haloperidol, risperidone, or olanzapine. Prim Care Companion J Clin Psychiatry 2010;12.

56. Malla A, Norman R, Scholten D, et al. A comparison of two novel antipsychotics in first episode non-affective psychosis: one-year outcome on symptoms, motor side effects and cognition. Psychiatry Res 2004;129:159-169.

57. Kopala LC, Good KP, Milliken H, et al. Treatment of a first episode of psychotic illness with quetiapine: an analysis of 2 year outcomes. Schizophr Res 2006;81:29-39.

58. Walther S, Federspiel A, Horn H, et al. Resting state cerebral blood flow and objective motor activity reveal basal ganglia dysfunction in schizophrenia. Psychiatry Res 2011;192:117-124.

59. Docx L, Emsell L, Van Hecke W, et al. White matter microstructure and volitional motor activity in

schizophrenia: A diffusion kurtosis imaging study. Psychiatry Res 2016;260:29-36.

60. Bracht T, Schnell S, Federspiel A, et al. Altered cortico-basal ganglia motor pathways reflect reduced volitional motor activity in schizophrenia. Schizophr Res 2013;143:269-276.

61. Karbasforoushan H, Duffy B, Blackford JU, Woodward ND. Processing speed impairment in schizophrenia is mediated by white matter integrity. Psychol Med 2015;45:109-120.

62. Singh S, Goyal S, Modi S, et al. Motor function deficits in schizophrenia: an fMRI and VBM study. Neuroradiology 2014;56:413-422.

63. Walther S, Schappi L, Federspiel A, et al. Resting-State Hyperperfusion of the Supplementary Motor Area in Catatonia. Schizophr Bull 2017;43:972-981.

64. Scheuerecker J, Ufer S, Kapernick M, et al. Cerebral network deficits in post-acute catatonic schizophrenic patients measured by fMRI. J Psychiatr Res 2009;43:607-614.

65. Payoux P, Boulanouar K, Sarramon C, et al. Cortical motor activation in akinetic schizophrenic patients: a pilot functional MRI study. Mov Disord 2004;19:83-90.

66. Bunse T, Wobrock T, Strube W, et al. Motor cortical excitability assessed by transcranial magnetic stimulation in psychiatric disorders: a systematic review. Brain Stimul 2014;7:158-169.

67. Hasan A, Wobrock T, Grefkes C, et al. Deficient inhibitory cortical networks in antipsychotic-naive subjects at risk of developing first-episode psychosis and first-episode schizophrenia patients: a cross-sectional study. Biol Psychiatry 2012;72:744-751.

68. Du X, Kochunov P, Summerfelt A, Chiappelli J, Choa FS, Hong LE. The role of white matter microstructure in inhibitory deficits in patients with schizophrenia. Brain Stimul 2017;10:283-290.

69. Hallett M. Transcranial magnetic stimulation: a primer. Neuron 2007;55:187-199.

70. Orosz A, Jann K, Wirth M, Wiest R, Dierks T, Federspiel A. Theta burst TMS increases cerebral blood flow in the primary motor cortex during motor performance as assessed by arterial spin labeling (ASL). Neuroimage 2012;61:599-605.

71. Siebner HR, Filipovic SR, Rowe JB, et al. Patients with focal arm dystonia have increased sensitivity to slow-frequency repetitive TMS of the dorsal premotor cortex. Brain 2003;126:2710-2725.

72. Ji GJ, Yu F, Liao W, Wang K. Dynamic aftereffects in supplementary motor network following inhibitory transcranial magnetic stimulation protocols. Neuroimage 2017;149:285-294.

73. Oxley T, Fitzgerald PB, Brown TL, de Castella A, Daskalakis ZJ, Kulkarni J. Repetitive transcranial magnetic stimulation reveals abnormal plastic response to premotor cortex stimulation in schizophrenia. Biol Psychiatry 2004;56:628-633.

74. Peralta V, Cuesta MJ. Motor Abnormalities: From Neurodevelopmental to Neurodegenerative Through "Functional" (Neuro)Psychiatric Disorders. Schizophr Bull 2017;43:956-971.

75. Speer AM, Willis MW, Herscovitch P, et al. Intensity-dependent regional cerebral blood flow during 1-Hz repetitive transcranial magnetic stimulation (rTMS) in healthy volunteers studied with H215O positron emission tomography: I. Effects of primary motor cortex rTMS. Biol Psychiatry 2003;54:818-825.

76. Tallabs FA, Hammond-Tooke GD. Theta priming of 1-Hz rTMS in healthy volunteers: effects on motor inhibition. J Clin Neurophysiol 2013;30:79-85.

77. Shirota Y, Ohtsu H, Hamada M, Enomoto H, Ugawa Y, Research Committee on r TMSToPsD. Supplementary motor area stimulation for Parkinson disease: a randomized controlled study. Neurology 2013;80:1400-1405.

78. Benninger DH, Berman BD, Houdayer E, et al. Intermittent theta-burst transcranial magnetic stimulation for treatment of Parkinson disease. Neurology 2011;76:601-609.

79. Kindler J, Homan P, Jann K, et al. Reduced neuronal activity in language-related regions after transcranial magnetic stimulation therapy for auditory verbal hallucinations. Biol Psychiatry 2013;73:518-524.

80. Hoffman RE, Hawkins KA, Gueorguieva R, et al. Transcranial magnetic stimulation of left temporoparietal cortex and medication-resistant auditory hallucinations. Arch Gen Psychiatry 2003;60:49-56.

81. Ray P, Sinha VK, Tikka SK. Adjuvant low-frequency rTMS in treating auditory hallucinations in recent-onset schizophrenia: a randomized controlled study investigating the effect of high-frequency priming stimulation. Ann Gen Psychiatry 2015;14:8.

82. Bais L, Vercammen A, Stewart R, et al. Short and long term effects of left and bilateral repetitive transcranial magnetic stimulation in schizophrenia patients with auditory verbal hallucinations: a randomized controlled trial. PLoS One 2014;9:e108828.

83. Klirova M, Horacek J, Novak T, et al. Individualized rTMS neuronavigated according to regional brain metabolism ((18)FGD PET) has better treatment effects on auditory hallucinations than standard positioning of rTMS: a double-blind, sham-controlled study. Eur Arch Psychiatry Clin Neurosci 2013;263:475-484.

84. Paillere-Martinot ML, Galinowski A, Plaze M, et al. Active and placebo transcranial magnetic stimulation effects on external and internal auditory hallucinations of schizophrenia. Acta Psychiatr Scand 2017;135:228-238.

85. Kindler J, Homan P, Flury R, Strik W, Dierks T, Hubl D. Theta burst transcranial magnetic stimulation for the treatment of auditory verbal hallucinations: results of a randomized controlled study. Psychiatry Res 2013;209:114-117.

86. Vercammen A, Knegtering H, Bruggeman R, et al. Effects of bilateral repetitive transcranial magnetic stimulation on treatment resistant auditory-verbal hallucinations in schizophrenia: a randomized controlled trial. Schizophr Res 2009;114:172-179.

87. Blumberger DM, Vila-Rodriguez F, Thorpe KE, et al. Effectiveness of theta burst versus high-frequency repetitive transcranial magnetic stimulation in patients with depression (THREE-D): a randomised non-inferiority trial. Lancet 2018;391:1683-1692.

88. Duprat R, Desmyter S, Rudi de R, et al. Accelerated intermittent theta burst stimulation treatment in medication-resistant major depression: A fast road to remission? J Affect Disord 2016;200:6-14.

89. van Lutterveld R, Koops S, Schutter DJ, et al. The effect of rTMS on auditory hallucinations: clues from an EEG-rTMS study. Schizophr Res 2012;137:174-179.

90. Nahas Z, Bohning DE, Molloy MA, Oustz JA, Risch SC, George MS. Safety and feasibility of repetitive transcranial magnetic stimulation in the treatment of anxious depression in pregnancy: a case report. J Clin Psychiatry 1999;60:50-52.

91. Klirova M, Novak T, Kopecek M, Mohr P, Strunzova V. Repetitive transcranial magnetic stimulation (rTMS) in major depressive episode during pregnancy. Neuro Endocrinol Lett 2008;29:69-70.

92. Widlöcher D, Ghozlan A. The measurement of retardation in depression. In: Hindmarch I, Stonier PD, eds. Human Psychopharmacology: Measures and Methods. New York: Wiley, 1989.

93. Kay SR, Fiszbein A, Opler LA. The positive and negative syndrome scale (PANSS) for schizophrenia. Schizophr Bull 1987;13:261-276.

94. Kirkpatrick B, Strauss GP, Nguyen L, et al. The brief negative symptom scale: psychometric properties. Schizophr Bull 2011;37:300-305.

95. Craig CL, Marshall AL, Sjostrom M, et al. International physical activity questionnaire: 12-country reliability and validity. Med Sci Sports Exerc 2003;35:1381-1395.

96. Dollfus S, Mach C, Morello R. Self-Evaluation of Negative Symptoms: A Novel Tool to Assess Negative Symptoms. Schizophr Bull 2016;42:571-578.

97. Endicott J, Spitzer RL, Fleiss JL, Cohen J. The global assessment scale. A procedure for measuring overall severity of psychiatric disturbance. Arch Gen Psychiatry 1976;33:766-771.

98. Goldman HH, Skodol AE, Lave TR. Revising axis V for DSM-IV: a review of measures of social functioning. Am J Psychiatry 1992;149:1148-1156.

99. Mausbach BT, Depp CA, Bowie CR, et al. Sensitivity and specificity of the UCSD Performance-based Skills Assessment (UPSA-B) for identifying functional milestones in schizophrenia. Schizophr Res 2011;132:165-170.

100. Huang YZ, Edwards MJ, Rounis E, Bhatia KP, Rothwell JC. Theta burst stimulation of the human motor cortex. Neuron 2005;45:201-206.

101. Oldfield RC. The assessment and analysis of handedness: the Edinburgh inventory. Neuropsychologia 1971;9:97-113.

102. Andreasen NC, Flaum M, Arndt S. The Comprehensive Assessment of Symptoms and History (CASH). An instrument for assessing diagnosis and psychopathology. Arch Gen Psychiatry 1992;49:615-623.

103. Kircher T, Krug A, Stratmann M, et al. A rating scale for the assessment of objective and subjective formal Thought and Language Disorder (TALD). Schizophr Res 2014;160:216-221.

104. Buchanan RW, Heinrichs DW. The Neurological Evaluation Scale (NES): a structured instrument for the assessment of neurological signs in schizophrenia. Psychiatry Res 1989;27:335-350.

105. Fahn S, Elton RL, Members UP. Unified Parkinson's disease rating scale. In: Fahn S, Marsden CD, Goldstein M, Calne DB, eds. Recent developments in Parkinson's disease. Florham Park,

NJ: Macmillan Healthcare Information, 1987.

106. Bush G, Fink M, Petrides G, Dowling F, Francis A. Catatonia. I. Rating scale and standardized examination. Acta Psychiatr Scand 1996;93:129-136.

107. Guy W. ECDEU Assessment Manual for Psychopharmacology. Rockville, MD: US Department of Health, Education and

Welfare, 1976.

108. Walther S, Federspiel A, Horn H, et al. White matter integrity associated with volitional motor activity. Neuroreport 2010;21:381-385.

109. Walther S, Horn H, Koschorke P, Muller TJ, Strik W. Increased motor activity in cycloid psychosis compared to schizophrenia. World J Biol Psychiatry 2009;10:746-751.

110. Walther S, Horn H, Razavi N, Koschorke P, Muller TJ, Strik W. Quantitative motor activity differentiates schizophrenia subtypes. Neuropsychobiology 2009;60:80-86.

111. Walther S, Horn H, Razavi N, et al. Higher motor activity in schizophrenia patients treated with olanzapine versus risperidone. J Clin Psychopharmacol 2010;30:181-184.

112. Vanbellingen T, Wapp M, Stegmayer K, et al. Theta burst stimulation over premotor cortex in Parkinson's disease: an explorative study on manual dexterity. J Neural Transm (Vienna) 2016;123:1387-1393.

113. Rossini PM, Burke D, Chen R, et al. Non-invasive electrical and magnetic stimulation of the brain, spinal cord, roots and peripheral nerves: Basic principles and procedures for routine clinical and research application. An updated report from an I.F.C.N. Committee. Clin Neurophysiol 2015;126:1071-1107.

114. Claassen J, Spiegel R, Kalla R, et al. A randomised double-blind, cross-over trial of 4-aminopyridine for downbeat nystagmus--effects on slowphase eye velocity, postural stability, locomotion and symptoms. J Neurol Neurosurg Psychiatry 2013;84:1392-1399.

115. Krafczyk S, Tietze S, Swoboda W, Valkovic P, Brandt T. Artificial neural network: a new diagnostic posturographic tool for disorders of stance. Clin Neurophysiol 2006;117:1692-1698.

116. Genzel L, Dresler M, Cornu M, et al. Medial prefrontal-hippocampal connectivity and motor memory consolidation in depression and schizophrenia. Biol Psychiatry 2015;77:177-186.

117. Yokoi A, Arbuckle SA, Diedrichsen J. The Role of Human Primary Motor Cortex in the Production of Skilled Finger Sequences. J Neurosci 2018;38:1430-1442.

17. APPENDICES

1. IB (according to ISO 14155) – “Produktinformation”
2. TMS device manual
3. Case Report Forms, v6 of June 6th 2020, patient version and healthy control version, v1 of February 18th 2021, clinical control version
4. Study Information for patients, v5 of January 1st 2021
5. Study Information for healthy controls, v5 of January 1st 2021
6. Study information for clinical controls, v1 of February 18th 2021

ClinicalTrials.gov

Go to the classic website

Record 1 of 4

The U.S. government does not review or approve the safety and science of all studies listed on this website.

Read our full disclaimer (https://www.clinicaltrials.gov/about-site/disclaimer) for details.

COMPLETED

Overcoming Psychomotor Slowing in Psychosis (OCoPS-P) (OCoPS-P)

ClinicalTrials.gov ID NCT03921450

Sponsor University of Bern

Information provided by University of Bern (Responsible Party)

Last Update Posted 2023-02-14

Record History Tab

Study Record Versions

- This table shows the list of all the versions of this study record arranged in order by date.
- The first column shows the date the study record was updated, and the second column shows the elements in the study record that were changed.
  - To view a version of a study record, click the version date.
  - To compare versions, select two versions using the check boxes and click the "Compare" button at the bottom of the list.

	Date	Changes
✓	2019-04-16	None (earliest version on record)

Compare

Feedback

	Date	Changes
☐	2019-04-25	Study Status<br>Outcome Measures
☐	2020-04-27	Study Status<br>Study Description<br>Contacts/Locations
☐	2021-05-11	Study Status
☐	2023-02-13	Recruitment Status<br>Study Status<br>Study Design

Compare

Version 1: 2019-04-16

Study Details

Study Identification

Unique Protocol ID

2018-02164

Brief Title

Overcoming Psychomotor Slowing in Psychosis (OCoPS-P)

Official Title

Overcoming Psychomotor Slowing in Psychosis (OCoPS-P): a 3-week, Randomized, Double-blind, Placebo-controlled Trial of add-on Repetitive Transcranial Magnetic Stimulation for Psychomotor Slowing in Psychosis

Secondary IDs

Feedback

Study Status

Record Verification

2019-04

Overall Status

Recruiting

Study Start

2019-03-25 [Actual]

Primary Completion

2023-02-01 [Estimated]

Study Completion

2024-01-01 [Estimated]

First Submitted

2019-04-16

First Submitted that Met QC Criteria

2019-04-16

First Posted

2019-04-19

Last Update Submitted that Met QC Criteria

2019-04-16

Last Update Posted

2019-04-19 [Actual]

Feedback

Sponsor/Collaborator

Sponsor

University of Bern

Responsible Party

Sponsor

Collaborators

Oversight

U.S. FDA-regulated Drug

No

U.S. FDA-regulated Device

No

Data Monitoring

Study Description

Brief Summary

Psychomotor slowing is a major problem in psychosis. Aberrant function of the cerebral motor system is linked to psychomotor slowing in patients, particularly resting state hyperactivity in premotor cortices. A previous clinical trial indicated that inhibitory stimulation of the premotor cortex would reduce psychomotor slowing. The current study is further exploring this effect in a randomized, placebo-controlled, double-blind design with three arms of transcranial magnetic stimulation and measures of brain imaging and physiology prior to and after the intervention.

Detailed Description

Feedback

Conditions

Condition

Schizophrenia and Related Disorders
Schizophrenia
Schizoaffective Disorder
Brief Psychotic Disorder

Keywords

motor behavior
psychomotor slowing
psychosis

Study Design

Study Type

Interventional

Primary Purpose

Treatment

Study Phase

Not Applicable

Interventional Study Model

Parallel Assignment

Interventional Model Description

3 week intervention with 15 sessions of add-on rTMS in 4 parallel arms, randomized, double-blind, placebo-controlled

Number of Arms

4

Masking

Triple (Participant, Care Provider, Outcomes Assessor)

Masking Description

Participants will not know the stimulation protocol, neither will the outcome assessor or the mental health care provider know the protocol applied

Allocation

Feedback

Randomized
**Enrollment**
88 [Estimated]

Arms and Interventions

Feedback

Arms	Assigned Interventions
Experimental: Inhibitory repetitive	Device: 1 Hz rTMS

Outcome Measures

Primary Outcome Measures

1. Proportion of responders at week 3
   Proportion of participants with >30% reduction from baseline in the Salpetriere Retardation Rating Scale (SRRS)
   [Time Frame: Week 3]
2. Change in Salpetriere Retardation Rating Scale (SSRS) from baseline
   Change in the Salpetriere Retardation Rating Scale (SRRS) from baseline
   [Time Frame: Week 3]

Secondary Outcome Measures

Feedback

1. Change in SSRS from baseline
   Change in the Salpetriere Retardation Rating Scale (SRRS) from baseline
   [Time Frame: Week 6]
2. Change in SSRS from baseline
   Change in the Salpetriere Retardation Rating Scale (SRRS) from baseline
   [Time Frame: Week 24]
3. Change in catatonia severity from baseline to week 3
   Observer based rating of catatonia severity with the Bush Francis Catatonia Rating Scale, assessment blind to intervention
   [Time Frame: Week 3]
4. Change in catatonia severity from baseline to week 6
   Observer based rating of catatonia severity with the Bush Francis Catatonia Rating Scale, assessment blind to intervention
   [Time Frame: Week 6]
5. Change in catatonia severity from baseline to week 24
   Observer based rating of catatonia severity with the Bush Francis Catatonia Rating Scale, assessment blind to intervention
   [Time Frame: Week 24]
6. Change in negative symptoms from baseline
   Change in the Brief Negative Symptom Scale (BNSS) from baseline
   [Time Frame: Week 3]
7. Change in negative symptoms from baseline
   Change in the Brief Negative Symptom Scale (BNSS) from baseline
   [Time Frame: Week 6]
8. Change in negative symptoms from baseline
   Change in the Brief Negative Symptom Scale (BNSS) from baseline
   [Time Frame: Week 24]
9. Change in psychosis severity from baseline
   Change in the Positive And Negative Symptom Scale (PANSS) from baseline
   [Time Frame: Week 3]
10. Change in psychosis severity from baseline
    Change in the Positive And Negative Symptom Scale (PANSS) from baseline
    [Time Frame: Week 6]
11. Change in psychosis severity from baseline
    Change in the Positive And Negative Symptom Scale
    [illegible]

Feedback

(PANSS) from baseline
[Time Frame: Week 24]

12. Change in physical activity self report from baseline
    Change in the International Physical Activity Questionnaire (IPAQ)
    [Time Frame: Week 3]
13. Change in physical activity self report from baseline
    Change in the International Physical Activity Questionnaire (IPAQ)
    [Time Frame: Week 6]
14. Change in physical activity self report from baseline
    Change in the International Physical Activity Questionnaire (IPAQ)
    [Time Frame: Week 24]
15. Change in objectively measured physical activity from baseline
    Change in the activity levels using wrist actigraphy
    [Time Frame: Week 3]
16. Change in objectively measured physical activity from baseline
    Change in the activity levels using wrist actigraphy
    [Time Frame: Week 6]
17. Change in objectively measured physical activity from baseline
    Change in the activity levels using wrist actigraphy
    [Time Frame: Week 24]
18. Change in dexterity from baseline
    Change in the coin rotation task from baseline
    [Time Frame: Week 3]
19. Change in dexterity from baseline
    Change in the coin rotation task from baseline
    [Time Frame: Week 6]
20. Change in dexterity from baseline
    Change in the coin rotation task from baseline
    [Time Frame: Week 24]
21. Change in cortical excitability of the motor cortex from baseline
    Change in Short Interval Cortical Inhibition (SICI) from baseline
    [Time Frame: Week 3]
22. Change in cortical excitability of the motor cortex from baseline
    Change in Short Interval Cortical Inhibition (SICI) from baseline
    [Time Frame: Week 6]
23. Change in cortical excitability of the motor cortex from

Feedback

baseline
Change in Short Interval Cortical Inhibition (SICI) from baseline
[Time Frame: Week 24]

24. Change in social and community functioning
    Change in Social and Occupational Functional Assesment Scale (SOFAS) from baseline
    [Time Frame: Week 3]
25. Change in social and community functioning
    Change in Social and Occupational Functional Assesment Scale (SOFAS) from baseline
    [Time Frame: Week 6]
26. Change in social and community functioning

Eligibility

Minimum Age

18 Years

Maximum Age

60 Years

Sex

All

Accepts Healthy Volunteers

Yes

Criteria

Feedback

Inclusion Criteria:

- Right-handed subjects
- Ability and willingness to participate in the study
- Ability to provide written informed consent
- Informed Consent as documented by signature
- Schizophrenia spectrum disorder according to diagnostic and statistical manual version 5 (DSM-5) criteria with current psychomotor slowing according to the Salpetriere Retardation Rating Scale (SRRS), score >= 15

Exclusion Criteria:

- Substance abuse or dependence other than nicotine
- Past or current medical or neurological condition associated with impaired or aberrant movement, such as brain tumors, stroke, M. Parkinson, M. Huntington, dystonia, or severe head trauma with subsequent loss of consciousness.
- Epilepsy or other convulsions
- History of any hearing problems or ringing in the ears
- Standard exclusion criteria for MRI scanning and TMS; [illegible]

Contacts/Locations

Central Contact Person

- Name
  Sebastian Walther, MD

  Telephone
  +41316328979

  Email
  sebastian.walther@upd.unibe.ch

- Name
  Petra Viher, PhD

  Email
  petra.viher@upd.unibe.ch

Study Officials

- Name
  Sebastian Walther, MD

  Role
  Principal Investigator

  Affiliation
  University of Bern

Feedback

Location

- **Bern, Switzerland, 3000**

  Status:
  Recruiting

  Facility:
  University Hospital of Psychiatry

  Contact:
  - Contact
    - Niluja Nadesalingam, MSc
    - niluja.nadesalingam@upd.unibe.ch
  - Sub-Investigator
    - Danai Alexaki, MD
  - Principal Investigator
    - Sebastian Walther, MD

IPD Sharing

Available IPD/Information

IPD information

Plan to Share IPD:
No

References

Citations

Links

Document Section

Study Protocol and Statistical Analysis Plan

Feedback

- Document Label
  Study Protocol and Statistical Analysis Plan

  Date
  2018-12-13

  Uploaded
  2019-04-16 07:44

  File name
  Prot_SAP_000.pdf (https://storage.googleapis.com/ctgov2-large-docs/50/NCT03921450/Prot_SAP_000.pdf)

Feedback

Feedback

ClinicalTrials.gov

Go to the classic website

Record 1 of 4

The U.S. government does not review or approve the safety and science of all studies listed on this website.

Read our full disclaimer (https://www.clinicaltrials.gov/about-site/disclaimer) for details.

COMPLETED

Overcoming Psychomotor Slowing in Psychosis (OCoPS-P) (OCoPS-P)

ClinicalTrials.gov ID NCT03921450

Sponsor University of Bern

Information provided by University of Bern (Responsible Party)

Last Update Posted 2023-02-14

Record History Tab

Study Record Versions

- This table shows the list of all the versions of this study record arranged in order by date.
- The first column shows the date the study record was updated, and the second column shows the elements in the study record that were changed.
  - To view a version of a study record, click the version date.
  - To compare versions, select two versions using the check boxes and click the "Compare" button at the bottom of the list.

	Date	Changes
☐	2019-04-16	None (earliest version on record)

Compare

Feedback

	Date	Changes
☐	2019-04-25	Study Status<br>Outcome Measures
☐	2020-04-27	Study Status<br>Study Description<br>Contacts/Locations
☐	2021-05-11	Study Status
☐	2023-02-13	Recruitment Status<br>Study Status<br>Study Design

Compare

Version 2: 2019-04-25

Study Details

Study Identification

Unique Protocol ID

2018-02164

Brief Title

Overcoming Psychomotor Slowing in Psychosis (OCoPS-P)

Official Title

Overcoming Psychomotor Slowing in Psychosis (OCoPS-P): a 3-week, Randomized, Double-blind, Placebo-controlled Trial of add-on Repetitive Transcranial Magnetic Stimulation for Psychomotor Slowing in Psychosis

Secondary IDs

Feedback

Study Status

Record Verification

2019-04

Overall Status

Recruiting

Study Start

2019-03-25 [Actual]

Primary Completion

2023-02-01 [Estimated]

Study Completion

2024-01-01 [Estimated]

First Submitted

2019-04-16

First Submitted that Met QC Criteria

2019-04-16

First Posted

2019-04-19

Last Update Submitted that Met QC Criteria

2019-04-25

Last Update Posted

2019-04-29 [Actual]

Feedback

Sponsor/Collaborator

Sponsor

University of Bern

Responsible Party

Sponsor

Collaborators

Oversight

U.S. FDA-regulated Drug

No

U.S. FDA-regulated Device

No

Data Monitoring

Study Description

Brief Summary

Psychomotor slowing is a major problem in psychosis. Aberrant function of the cerebral motor system is linked to psychomotor slowing in patients, particularly resting state hyperactivity in premotor cortices. A previous clinical trial indicated that inhibitory stimulation of the premotor cortex would reduce psychomotor slowing. The current study is further exploring this effect in a randomized, placebo-controlled, double-blind design with three arms of transcranial magnetic stimulation and measures of brain imaging and physiology prior to and after the intervention.

Detailed Description

Feedback

Conditions

Condition

Schizophrenia and Related Disorders
Schizophrenia
Schizoaffective Disorder
Brief Psychotic Disorder

Keywords

motor behavior
psychomotor slowing
psychosis

Study Design

Study Type

Interventional

Primary Purpose

Treatment

Study Phase

Not Applicable

Interventional Study Model

Parallel Assignment

Interventional Model Description

3 week intervention with 15 sessions of add-on rTMS in 4 parallel arms, randomized, double-blind, placebo-controlled

Number of Arms

4

Masking

Triple (Participant, Care Provider, Outcomes Assessor)

Masking Description

Participants will not know the stimulation protocol, neither will the outcome assessor or the mental health care provider know the protocol applied

Allocation

Feedback

Randomized

Enrollment

88 [Estimated]

Arms and Interventions

Feedback

Arms	Assigned Interventions
Experimental: Inhibitory repetitive	Device: 1 Hz rTMS

Outcome Measures

Primary Outcome Measures
1. Proportion of responders at week 3<br>Proportion of participants with >30% reduction from baseline in the Salpetriere Retardation Rating Scale (SRRS)<br>[Time Frame: Week 3]<br>2. Change in Salpetriere Retardation Rating Scale (SSRS) from baseline<br>Change in the Salpetriere Retardation Rating Scale (SRRS) from baseline; the total score of 15 items is used, ranging 0-60 with higher scores indicating worse outcome<br>[Time Frame: Week 3, week 6, week 24]

Secondary Outcome Measures

Feedback

1. Change in catatonia severity from baseline to week 3
   Observer based rating of catatonia severity with the Bush Francis Catatonia Rating Scale (BFCRS), assessment blind to intervention, total score of the BFCRS is used ranging 0-69 , with higher scores indicating poorer outcome
   [Time Frame: Week 3, week 6, week 24]
2. Change in negative symptoms from baseline
   Change in the Brief Negative Symptom Scale (BNSS) from baseline, total score is used, ranging from 0-78 with higher values indicating poorer outcome, i.e. more negative symptom severity
   [Time Frame: Week 3, week 6, week 24]
3. Change in psychosis severity from baseline
   Change in the Positive And Negative Symptom Scale (PANSS) from baseline, PANSS total score assesses the severity of positive, negative and general symptoms, ranging from 30-210 with higher scores indicating increased symptom severity, i.e. poorer outcome
   [Time Frame: Week 3, week 6, week 24]
4. Change in physical activity self report from baseline
   Change in the International Physical Activity Questionnaire (IPAQ), the total score is used ranging from 0-70000 metabolic equivalent (MET)
   [Time Frame: Week 3, week 6, week 24]
5. Change in objectively measured physical activity from baseline
   Change in the activity levels using wrist actigraphy
   [Time Frame: Week 3, week 6, week 24]
6. Change in dexterity from baseline
   Change in the coin rotation task from baseline
   [Time Frame: Week 3, week 6, week 24]
7. Change in cortical excitability of the motor cortex from baseline
   Change in Short Interval Cortical Inhibition (SICI) from baseline

Eligibility

Minimum Age
18 Years

Maximum Age
60 Years

Feedback

Sex

All

Accepts Healthy Volunteers

Yes

Criteria

Inclusion Criteria:

- Right-handed subjects
- Ability and willingness to participate in the study
- Ability to provide written informed consent
- Informed Consent as documented by signature
- Schizophrenia spectrum disorder according to diagnostic and statistical manual version 5 (DSM-5) criteria with current psychomotor slowing according to the Salpetriere Retardation Rating Scale (SRRS), score >= 15

Exclusion Criteria:

- Substance abuse or dependence other than nicotine
- Past or current medical or neurological condition associated with impaired or aberrant movement, such as brain tumors, stroke, M. Parkinson, M. Huntington, dystonia, or severe head trauma with subsequent loss of consciousness.
- Epilepsy or other convulsions
- History of any hearing problems or ringing in the ears
- Standard exclusion criteria for MRI scanning and TMS; e.g. metal implants, claustrophobia
- Patients only: any TMS treatment in the past 3 months
- Women who are pregnant or breast feeding,
- Intention to become pregnant during the course of the study,
- Female participants who are surgically sterilised / hysterectomised or post-menopausal for longer than 2 years are not considered as being of child bearing potential.
- Previous enrolment into the current study,
- Enrolment of the investigator, his/her family members, employees and other dependent persons
- Controls only: history of any psychiatric disorder or first-degree relatives with schizophrenia spectrum disorders.

Feedback

Contacts/Locations

Central Contact Person

- Name
  Sebastian Walther, MD

  Telephone
  +41316328979

  Email
  sebastian.walther@upd.unibe.ch
- Name
  Petra Viher, PhD

  Email
  petra.viher@upd.unibe.ch

Study Officials

- Name
  Sebastian Walther, MD

  Role
  Principal Investigator

  Affiliation
  University of Bern

Location

- **Bern, Switzerland, 3000**

  Status:
  Recruiting

  Facility:
  University Hospital of Psychiatry

  Contact:
  - Contact
    - Niluja Nadesalingam, MSc
    - niluja.nadesalingam@upd.unibe.ch
  - Sub-Investigator
    - Danai Alexaki, MD
  - Principal Investigator
    - Sebastian Walther, MD

IPD Sharing

Available IPD/Information

IPD information

Feedback

Plan to Share IPD:
No

References

Citations

Links

Document Section

Study Protocol and Statistical Analysis Plan

- Document Label
  Study Protocol and Statistical Analysis Plan

  Date
  2018-12-13

  Uploaded
  2019-04-16 07:44

  File name
  Prot_SAP_000.pdf (https://storage.googleapis.com/ctgov2-large-docs/50/NCT03921450/Prot_SAP_000.pdf)

Feedback
