# Supplementary material for: Psychomotor Slowing in Psychosis and Inhibitory Repetitive Transcranial Magnetic Stimulation: A Randomized Clinical Trial
Source: JAMA Psychiatry. 2024 Feb 28;81(6):563–71. doi: 10.1001/jamapsychiatry.2024.0026 (PMC10902782; doi:10.1001/jamapsychiatry.2024.0026)
Supplement: Supplement 2. — eMethods 1. Sample size estimation eMethods 2. Trial registration eTable 1. Inclusion/Exclusion criteria eMethods 3. Coils eMethods 4. Secondary outcome rating scale subscores eMethods 5. Actigraphy eMethods 6. Coin rotation task eMethods 7. Randomization and allocation concealment eResults eTable 2. Dropout reasons eTable 3. Primary and secondary outcomes according to treatment arm for ITT with LOCF and covariates eTable 4. Primary and secondary outcomes according to treatment arms with LOCF (raw) eTable 5. Post-hoc-p-values of Time with SIDAK correction eTable 6. Post-hoc-p-values the interaction between Time & Treatment arm eTable 7. Follow-ups with LOCF and covariates eTable 8. Primary and secondary outcomes according to treatment arm of completers with covariates eTable 9. Primary and secondary outcomes according to treatment arm with completers (raw) eTable 10. Follow ups of completers with covariates eTable 11. Side-effects in the waiting group eFigure 1. Course of anhedonia eFigure 2. Course of global functioning eTable 12. Baseline variables in the three sample versions eTable 13. Results of the primary outcomes in n=88 (ITT) vs. n=97 (with baseline), covaried for sex, baseline and treatment medication eReferences [file jamapsychiatry-e240026-s002.pdf]

Supplemental Online Content

Walther S, Alexaki D, Weiss F, et al. Psychomotor slowing in psychosis and inhibitory repetitive transcranial magnetic stimulation: a randomized clinical trial. *JAMA Psychiatry*. Published online February 28, 2024.
doi:10.1001/jamapsychiatry.2024.0026

eMethods 1. Sample size estimation
eMethods 2. Trial registration
eTable 1. Inclusion/Exclusion criteria
eMethods 3. Coils
eMethods 4. Secondary outcome rating scale subscores
eMethods 5. Actigraphy
eMethods 6. Coin rotation task
eMethods 7. Randomization and allocation concealment
eResults
eTable 2. Dropout reasons
eTable 3. Primary and secondary outcomes according to treatment arm for ITT with LOCF and covariates
eTable 4. Primary and secondary outcomes according to treatment arms with LOCF (raw)
eTable 5. Post-hoc-p-values of Time with SIDAK correction
eTable 6. Post-hoc-p-values the interaction between Time & Treatment arm
eTable 7. Follow-ups with LOCF and covariates
eTable 8. Primary and secondary outcomes according to treatment arm of completers with covariates
eTable 9. Primary and secondary outcomes according to treatment arm with completers (raw)
eTable 10. Follow ups of completers with covariates
eTable 11. Side-effects in the waiting group
eFigure 1. Course of anhedonia
eFigure 2. Course of global functioning
eTable 12. Baseline variables in the three sample versions
eTable 13. Results of the primary outcomes in n=88 (ITT) vs. n=97 (with baseline), covaried for sex, baseline and treatment medication
eReferences

This supplemental material has been provided by the authors to give readers additional information about their work.

© 2024 Walther S et al. *JAMA Psychiatry.*

eMethods

eMethods 1. Sample size estimation

The main effects of the 3-week rTMS interventions on psychomotor slowing (SRRS scores) was calculated in a repeated measures design including 2 assessment time points (Baseline, week 3) and four groups (lf-rTMS, iTBS, sham, waiting group-lf-rTMS). Assuming a moderate effect size (f = 0.23) as indicated by our pilot data in a repeated measures ANOVA with moderate correlation between time points (0.5), a power of 0.95 and an alpha = 0.05, we needed 88 patients (22 per group).

eMethods 2. Trial registration

The trial was registered 25 days after enrolling the first participant. On March 12, 2019, IRB communicated final approval of the protocol. On March 22, 2019, the first participant provided informed consent. First rTMS treatment started on March 27, 2019. Trial registration was conducted on April 16, 2019. At this time, the first patient had just completed week 3 measurements, another two patients were receiving rTMS, while another two patients had been enrolled but withdrawn consent before starting the intervention. Thus, when the trial was registered, a total of 3 (3.4%) of the ITT population had been enrolled in the study.

Trial registration at clinicaltrials.gov included a minimal revision of the measurement organization that was conducted on April 25, 2019. No changes were made to the study design or outcomes after that.

Trial registration allowed entering the planned two primary outcomes (proportion of responders at week 3 and change in SRRS total scores from baseline to week 3). The IRB protocol only allowed for one primary outcome, i.e., change in SRRS total scores from baseline to week 3. Therefore, in the IRB protocol, the proportion of responders was stated as first of the secondary outcomes. This discrepancy between IRB and clinical trial registration was present from April 2019 onwards. In the presentation of the manuscript, we adhere to the clinical trial registration.

© 2024 Walther S et al. *JAMA Psychiatry.*

eTable 1. Inclusion/Exclusion criteria

**Detailed inclusion and exclusion criteria.**	
Inclusion criteria	- 18–60 years old<br>- Schizophrenia spectrum disorders according to DSM-5 with psychomotor slowing (SRRS score ≥ 15)
Exclusion criteria	- Substance abuse or dependence other than nicotine<br>- Past or current medical or neurological conditions associated with impaired or aberrant movement, such as brain tumors, stroke, M. Parkinson, M. Huntington, dystonia, or severe head trauma with subsequent loss of consciousness<br>- Epilepsy or other convulsions<br>- History of any hearing problems or ringing in the ears<br>- Standard exclusion criteria for MRI scanning and TMS; e.g. metal implants, claustrophobia<br>- Any TMS treatment in the past 3 months<br>- Pregnancy or breastfeeding

eMethods 3. Coils

All stimulations were delivered using either MagPro X100 including MagOption or MagPro R30 with theta burst option, both manufactured by Tonica Electronik A/S, Denmark and distributed by Magventure A/S, Denmark. Each machine was consistently used at the same building of the department (MagPro X100 at Bolligenstrasse 111, MagPro R30 at Murtenstrasse 21). Patients were treated at the rTMS machine located closest to their inpatient stay.

For the real TMS-stimulations we used the MCF-B70 coil with transducer head dimensions of 180x116x50/67, magnetic field of 12kT/s in coil center at 20mm distance from coil surface and penetration depth (70V/m) of 34.0mm. In contrast, for sham stimulations, we used the MCF-P-B65 coil with transducer head dimensions of 172x94x53. These coils look almost identical and have identical sound emissions. However, the sham coil has no magnetic emissions.

eMethods 4. Secondary outcome rating scale subscores

Next to the total scores of the Positive And Negative Syndrome Scale (PANSS) [1], the Brief Negative Symptom Scale (BNSS) [2], the Bush-Francis Catatonia Rating Scale (BFCRS) [3], we calculated secondary outcomes for subscores of those scales.

We considered PANSS positive (sum of items P1-P7), PANSS negative (sum of items N1-N7), PANSS general psychopathology (sum of items G1-G16), BNSS anhedonia, distress, asocial, avolition, affect, alogia, as well as BFCRS decreased activity (sum of

© 2024 Walther S et al. *JAMA Psychiatry.*

items 2, 3, 4, 12, 14, 19), BFCRS abnormal activity (sum of items 5, 6, 7, 8, 9, 10, 11, 13, 16, 17, 18, 20, 21) [4].

eMethods 5. Actigraphy

The change in total physical activity during waking hours was measured with wrist actigraphy on the non-dominant arm (Move4 by movisens GmbH, Karlsruhe, Germany) [5]. Participants wore the instrument for > 24 consecutive hours. To this end, we used the activity level (activity counts/h of wake time) [6], which has been calculated in multiple of our previous studies[5,7-10]. Wake time of the day was confirmed based on inspection of the raw actigraphy data and crosschecking with the sleep/activity protocol participants provided.

eMethods 6. Coin rotation task

Manual dexterity of both hands was tested using the coin rotation task[11] . We have applied this task before in samples of patients with schizophrenia and healthy subjects[12-14] Participants were asked to rotate a Swiss 50-Rappen coin similar to the size of a US dime, between the first three fingers at a maximum speed with each hand during 3 trails of 10s, while being videotaped. Videos were analyzed by raters blind to allocation and time point of assessment. The first trial was a practice trial whereas the remaining two trials' scores were averaged and considered for the analysis. The following formula was used to calculate the score of each trial: CR score=half turns−[(coin drops×.10)×half turns] [11,14]

eMethods 7. Randomization and allocation concealment

Prior to the first participant enrolled, we used a web-based tool "research randomizer" to perform permutated block randomization. Lists with random order of the four arms were generated. This was done to ensure that all four treatment arms receive the same number of participants. Permutated block randomization is preferred in sample sizes < 100 as in our trial[15]. Only the principal investigator (SW) had access to the randomization protocol that was stored on a login-secured computer.

Furthermore, group allocation of the participants was performed according to the block randomization lists by the principal investigator immediately after participants signed the consent form - and before baseline assessments. This way, information from baseline assessments were not available during the group allocation process.

© 2024 Walther S et al. *JAMA Psychiatry.*

The principal investigator was not involved in participant screening or recruitment. The principal investigator informed the part of the team who conducted the rTMS treatments (only one person for each participant) about group allocation. The psychiatrists who performed the assessments (DA, DBG, and AK) had no access to the randomization lists or treatment allocation process.

Allocation concealment was assured by keeping the treatment allocation written in sealed envelopes, locked at the lab office. In case of emergency, e.g. serious adverse event, the principal investigator could have broken the code and informed the clinical team about the treatment allocation. However, the study would have stopped in this participant. Fortunately, SAEs have never occurred during the study. See also IRB protocol chapter 6 for information on randomization, blinding, and unblinding.

In treatment arms 1 Hz, iTBS, and sham participants, raters, and the mental health professionals delivering treatment were blind to the stimulation used. Conversely, the participants randomized to the patient group knew that they were not receiving rTMS during the first three weeks. They were told to receive real rTMS in weeks 4-6. In the participant information it was stated that after the waiting period, patients would receive rTMS. But as the clinical trial registration is publicly available, participants may have noted that the waiting group would receive inhibitory stimulation through week 4-6.

.

© 2024 Walther S et al. *JAMA Psychiatry.*

eResults

eTable 2. Dropout reasons

Reason	1Hz n	iTBS n	sham n	waiting n	Total n
Withdrew consent	4	< 10*	3	6	15
Lost to follow-up	< 10*	0	0	0	< 10*
Treating psychiatrist's decision	< 10*	< 10*	0	0	< 10*
Adverse event	0	< 10*	0	0	< 10*
Note: * There were too few patients to provide an exact number without compromising data identifiability requirements.					

Primary and secondary outcomes until week 3

LOCF

eTable 3. Primary and secondary outcomes according to treatment arm for ITT with LOCF and covariates.

Outcomes	Estimated Marginal Mean ± SE 1Hz	iTBS	sham	waiting	F-stats*	p	η^2
SRRS							
BL	24.2 ± 1.3	22.7 ± 1.3	24.8 ± 1.4	24.2 ± 1.3	Time:		
Week1	17.7 ± 1.2	20.7 ± 1.2	20.9 ± 1.2	-	$F_{(3, 237)}$ = 16.5	<.001	.172
Week2	15.8 ± 1.2	18.5 ± 1.2	18.9 ± 1.3	-	Time*Group:		
Week3	14.4 ± 1.2	17.5 ± 1.2	18.9 ± 1.3	23.8 ± 1.2	$F_{(9, 237)}$= 6.3	<.001	.192
$PANSS_{total}$							
BL	76.7 ± 4.0	81.6 ± 3.9	77.2 ± 4.1	84.6 ± 3.8	$F_{(1, 79)}$ = 17.4	<.001	.181
Week3	63.9 ± 4.1	66.1 ± 4.0	66.8 ± 4.2	74.5 ± 3.9	$F_{(3, 79)}$ = 0.5	.710	.017
$PANSS_{positive}$							
BL	15.4 ± 1.3	17.1 ± 1.2	14.4 ± 1.3	16.6 ± 1.2	$F_{(1, 79)}$ = 9.4	.003	.107
Week3	12.1 ± 1.1	13.6 ± 1.0	12.6 ± 1.1	13.7 ± 1.0	$F_{(3, 79)}$ = 0.5	.716	.017
$PANSS_{negative}$							
BL	22.8 ± 1.4	23.2 ± 1.4	24.7 ± 1.4	24.6 ± 1.3	$F_{(1, 79)}$ = 5.3	.023	.063
Week3	20.0 ± 1.5	19.4 ± 1.4	21.4 ± 1.5	23.1 ± 1.4	$F_{(3, 79)}$ = 0.7	.573	.025
$PANSS_{general}$							
BL	38.5 ± 2.1	41.2 ± 2.1	38.1 ± 2.2	43.4 ± 2.0	$F_{(1, 79)}$ = 19.4	<.001	.197
Week3	31.8 ± 2.1	33.1 ± 2.1	32.7 ± 2.2	37.8 ± 2.1	$F_{(3, 79)}$ = 0.4	.773	.014
$BNSS_{total}$							
BL	40.3 ± 2.9	38.2 ± 2.8	47.6 ± 3.0	43.2 ± 2.8	$F_{(1, 79)}$ = 5.2	.025	.062
Week3	35.5 ± 2.9	31.2 ± 2.8	41.1 ± 2.9	43.5 ± 2.8	$F_{(3, 79)}$ = 1.9	.137	.067
$BNSS_{Anhedonia}$							
BL	10.3 ± 1.1	9.2 ± 1.0	11.5 ± 1.1	8.9 ± 1.0	$F_{(1, 79)}$ = 2.4	.124	.030
Week3	8.8 ± 1.0	6.9 ± 1.0	9.8 ± 1.0	9.6 ± 0.9	$F_{(3, 79)}$ = 2.8	.048	.095
$BNSS_{Distress}$							
BL	2.9 ± 0.4	2.9 ± 0.4	3.3 ± 0.4	2.6 ± 0.4	$F_{(1, 79)}$ = 2.5	.117	.031

© 2024 Walther S et al. *JAMA Psychiatry.*

Week3	2.3 ± 0.4	2.0 ± 0.4	2.2 ± 0.4	2.9 ± 0.4	$F_{(3, 79)} = 3.2$	.026	.110
$BNSS_{Asocial}$							
BL	6.8 ± 0.5	6.7 ± 0.5	7.2 ± 0.6	7.2 ± 0.5	$F_{(1, 79)} = 4.8$	.032	.057
Week3	6.3 ± 0.6	4.7 ± 0.6	6.1 ± 0.6	6.1 ± 0.6	$F_{(3, 79)} = 1.1$	.355	.040
$BNSS_{Avolition}$							
BL	6.6 ± 0.7	6.2 ± 0.7	7.1 ± 0.7	6.6 ± 0.7	$F_{(1, 79)} = 0.9$	.345	.011
Week3	6.3 ± 0.6	5.2 ± 0.6	6.5 ± 0.6	6.7 ± 0.6	$F_{(3, 79)} = 0.5$	.705	.018
$BNSS_{Affect}$							
BL	9.6 ± 0.8	9.6 ± 0.8	12.0 ± 0.8	11.9 ± 0.8	$F_{(1, 79)} = 2.3$	.133	.028
Week3	8.5 ± 0.7	8.9 ± 0.7	10.7 ± 0.8	11.8 ± 0.7	$F_{(3, 79)} = 0.4$	.747	.015
$BNSS_{Alogia}$							
BL	4.1 ± 0.7	3.5 ± 0.7	6.4 ± 0.7	6.0 ± 0.7	$F_{(1, 79)} = 1.3$	.265	.016
Week3	3.3 ± 0.7	3.5 ± 0.6	5.7 ± 0.7	6.4 ± 0.6	$F_{(3, 79)} = 0.6$	.586	.024
$BFCRS_{total}$							
BL	4.2 ± 0.8	5.4 ± 0.8	7.8 ± 0.8	4.7 ± 0.8	$F_{(1, 79)} = 11.5$	.001	.127
Week3	2.6 ± 0.9	4.0 ± 0.8	4.9 ± 0.9	6.1 ± 0.8	$F_{(3, 79)} = 5.0$	.003	.160
$BFCRS_{abnormal}$							
BL	3.1 ± 0.7	3.9 ± 0.7	5.5 ± 0.7	3.2 ± 0.7	$F_{(1, 79)} = 7.0$	.010	.081
Week3	2.0 ± 0.7	3.1 ± 0.7	3.5 ± 0.7	3.9 ± 0.7	$F_{(3, 79)} = 2.8$	.044	.097
$BFCRS_{decreased}$							
BL	0.9 ± 0.3	1.4 ± 0.3	2.4 ± 0.3	1.3 ± 0.3	$F_{(1, 79)} = 7.6$	.007	.088
Week3	0.5 ± 0.3	0.7 ± 0.3	1.4 ± 0.3	2.2 ± 0.3	$F_{(3, 79)} = 5.0$	.003	.159
UPDRS							
BL	18.0 ± 2.5	21.7 ± 2.5	22.9 ± 2.6	20.8 ± 2.4	$F_{(1, 79)} = 3.9$	.053	.047
Week3	15.1 ± 1.9	16.4 ± 1.9	17.5 ± 2.0	18.5 ± 1.9	$F_{(3, 79)} = 1.9$	.558	.026
AIMS							
BL	1.1 ± 0.5	1.1 ± 0.5	1.1 ± 0.5	0.3 ± 0.5	$F_{(1, 79)} = 0.2$	.659	.002
Week3	0.8 ± 0.3	0.5 ± 0.3	0.9 ± 0.3	0.3 ± 0.3	$F_{(3, 79)} = 0.8$	.485	.030
GAF							
BL	44.7 ± 2.8	39.6 ± 2.7	38.4 ± 2.9	41.2 ± 2.7	$F_{(1, 79)} = 13.7$	<.001	.148
Week3	52.5 ± 3.3	50.0 ± 3.2	51.8 ± 3.3	48.5 ± 3.1	$F_{(3, 79)} = 1.0$	.402	.036
SOFAS							
BL	42.5 ± 2.8	41.3 ± 2.8	36.8 ± 2.9	41.1 ± 2.7	$F_{(1, 79)} = 2.6$	.110	.032
Week3	48.1 ± 3.1	50.3 ± 3.0	44.5 ± 3.2	47.5 ± 3.0	$F_{(3, 79)} = 0.2$	.864	.009
UPSA							
BL	71.5 ± 3.5	71.6 ± 3.3	74.2 ± 3.5	74.8 ± 3.3	$F_{(1, 77)} = 8.2$	.006	.096
Week3	77.6 ± 2.9	78.8 ± 2.8	78.7 ± 2.9	79.6 ± 2.7	$F_{(3, 77)} = 0.2$	.880	.009
IPAQ							
BL	849 ± 490	795 ± 478	2209 ± 499	1038 ± 470	$F_{(1, 79)} = 5.2$	.025	.062
Week3	907 ± 701	2473 ± 684	2080 ± 714	1957 ± 673	$F_{(3, 79)} = 2.0$	.120	.071
SNS							

© 2024 Walther S et al. *JAMA Psychiatry.*

BL	18.4 ± 1.9	20.9 ± 1.9	22.8 ± 1.9	19.7 ± 1.8	$F_{(1, 79)} = 1.0$	.330	.012
Week3	18.1 ± 2.1	18.0 ± 2.0	19.2 ± 2.1	18.2 ± 2.0	$F_{(3, 79)} = 1.0$	.398	.037
Actigraphy							
BL	13383 ±1110	14256±1088	11469±1099	11431±1016	$F_{(1, 74)} = 0.5$	.488	.007
Week3	14037±1400	17027±1372	12235±1387	12276±1282	$F_{(3, 74)} = 0.6$	.602	.025
$CR_{dominant}$							
BL	10.7 ± 0.8	10.9 ± 0.8	12.3 ± 0.9	10.9 ± 0.8	$F_{(1, 78)} = 3.1$	.082	.038
Week3	11.0 ± 0.8	11.8 ± 0.8	12.9 ± 0.8	11.3 ± 0.7	$F_{(3, 78)} = 0.3$	.838	.011
$CR_{nondominant}$							
BL	9.4 ± 0.8	9.3 ± 0.8	10.7 ± 0.8	9.4 ± 0.7	$F_{(1, 78)} = 0.0$	.991	.000
Week3	9.9 ± 0.8	10.1 ± 0.8	9.9 ± 0.8	9.8 ± 0.7	$F_{(3, 78)} = 1.7$	.175	.061

© 2024 Walther S et al. *JAMA Psychiatry.*

eTable 4. Primary and secondary outcomes according to treatment arms with LOCF (raw).

Outcomes	Mean ± SD 1Hz	iTBS	sham	waiting
SRRS				
BL	24.7 ± 5.8	23.3 ± 7.1	24.2 ± 5.7	23.7 ± 5.0
Week1	18.5 ± 6.2	21.1 ± 7.4	20.5 ± 4.1	-
Week2	16.7 ± 6.5	18.8 ± 7.3	18.6 ± 4.7	-
Week3	16.1 ± 5.8	17.9 ± 7.7	18.0 ± 3.8	22.5 ± 6.4
$PANSS_{total}$				
BL	76.3 ± 17.1	81.8 ± 19.9	77.3 ± 14.1	84.6 ± 17.8
Week3	66.6 ± 15.2	67.0 ± 21.8	64.8 ± 16.5	72.8 ± 17.9
$PANSS_{positive}$				
BL	14.8 ± 5.1	16.9 ± 6.4	15.0 ± 4.2	16.9 ± 5.8
Week3	12.0 ± 3.5	13.5 ± 5.9	12.9 ± 4.2	13.6 ± 4.4
$PANSS_{negative}$				
BL	22.9 ± 5.4	23.2 ± 6.1	24.7 ± 6.8	24.5 ± 6.0
Week3	20.8 ± 5.0	19.9 ± 6.8	20.5 ± 7.1	22.7 ± 6.5
$PANSS_{general}$				
BL	38.6 ± 10.2	41.7 ± 11.1	37.6 ± 7.3	43.2 ± 9.0
Week3	33.8 ± 9.1	33.6 ± 11.3	31.4 ± 8.9	36.5 ± 9.1
$BNSS_{total}$				
BL	42.0 ± 14.4	39.3 ± 12.2	45.7 ± 11.8	42.2 ± 12.4
Week3	39.0 ± 11.6	32.5 ± 15.2	38.3 ± 12.4	41.5 ± 13.5
$BNSS_{Anhedonia}$				
BL	11.1 ± 4.5	9.7 ± 4.5	10.7 ± 4.4	8.5 ± 5.1
Week3	10.0 ± 4.7	7.4 ± 4.3	8.8 ± 4.5	9.0 ± 4.4
$BNSS_{Distress}$				
BL	2.9 ± 1.8	3.0 ± 1.7	3.2 ± 1.5	2.6 ± 1.7
Week3	2.4 ± 1.6	2.0 ± 1.7	2.1 ± 1.6	2.9 ± 1.6
$BNSS_{Asocial}$				
BL	6.9 ± 2.3	6.5 ± 2.7	7.4 ± 2.5	7.1 ± 1.9.
Week3	6.6 ± 2.6	4.8 ± 2.8	6.0 ± 2.6	5.9 ± 2.8
$BNSS_{Avolition}$				
BL	6.8 ± 3.0	6.3 ± 3.0	6.9 ± 3.1	6.6 ± 2.8
Week3	6.6 ± 2.2	5.4 ± 3.1	6.2 ± 2.7	6.6 ± 2.6
$BNSS_{Affect}$				
BL	10.1 ± 4.0	9.9 ± 3.6	11.6 ± 3.4	11.6 ± 4.0
Week3	9.4 ± 3.4	9.1 ± 3.9	10.1 ± 3.4	11.2 ± 3.3
$BNSS_{Alogia}$				
BL	4.3 ± 3.4	3.9 ± 3.3	6.0 ± 2.8	5.8 ± 3.2
Week3	4.0 ± 3.2	3.9 ± 3.1	5.1 ± 2.8	6.0 ± 2.9

© 2024 Walther S et al. *JAMA Psychiatry.*

BFCRS$_{total}$				
BL	4.9 ± 3.7	5.9 ± 4.9	7.1 ± 3.7	4.2 ± 3.0
Week3	3.5 ± 3.6	4.2 ± 4.7	4.5 ± 4.2	5.4 ± 3.6
BFCRS$_{abnormal}$				
BL	3.6 ± 2.7	4.2 ± 4.1	5.0 ± 3.0	2.9 ± 2.8
Week3	2.7 ± 2.5	3.2 ± 4.0	3.3 ± 3.3	3.4 ± 2.6
BFCRS$_{decreased}$				
BL	1.1 ± 1.8	1.6 ± 1.5	2.0 ± 1.6	1.2 ± 1.2
Week3	0.8 ± 1.5	0.9 ± 1.0	1.1 ± 1.4	2.0 ± 1.9
UPDRS				
BL	18.7 ± 8.7	21.3 ± 13.8	23.4 ± 11.4	20.0 ± 9.7
Week3	15.2 ± 7.4	15.7 ± 10.3	18.6 ± 7.2	18.0 ± 9.4
AIMS				
BL	1.4 ± 3.1	1.1 ± 2.0	0.9 ± 2.2	0.1 ± 0.4
Week3	0.9 ± 1.7	0.5 ± 1.3	0.8 ± 1.5	0.3 ± 0.8
GAF				
BL	43.7 ± 11.7	39.1 ± 14.1	39.0 ± 9.9	42.1 ± 12.7
Week3	50.9 ± 11.9	50.5 ± 18.6	51.6 ± 12.2	49.8 ± 14.7
SOFAS				
BL	41.0 ± 14.9	40.3 ± 12.6	38.0 ± 10.7	42.2 ± 11.7
Week3	46.1 ± 12.3	50.1 ± 16.2	45.5 ± 9.6	48.7 ± 15.6
UPSA				
BL	70.6 ± 13.9	71.2 ± 17.9	74.9 ± 14.6	75.3 ± 12.0
Week3	76.0 ± 12.9	78.8 ± 13.9	79.1 ± 12.2	80.6 ± 10.3
IPAQ				
BL	1008 ± 1283	815 ± 940	2191 ± 3732	875 ± 1304
Week3	1091 ± 1570	2356 ± 4019	2114 ± 3738	1857 ± 2000
SNS				
BL	18.9 ± 9.3	21.1 ± 8.0	22.0 ± 8.0	19.7 ± 7.8
Week3	18.3 ± 10.0	18.0 ± 8.9	18.7 ± 9.0	18.5 ± 8.0
Actigraphy				
BL	12978 ± 4795	13548 ± 4961	12028 ± 3493	11921 ± 4924
Week3	13568 ± 5312	15851 ± 8122	13212 ± 4758	12873 ± 4390
CR$_{dominant}$				
BL	10.8 ± 3.2	10.9 ± 3.5	12.2 ± 4.4	10.9 ± 3.4
Week3	11.2 ± 2.8	12.0 ± 2.8	12.5 ± 4.3	11.3 ± 3.6
CR$_{nondominant}$				
BL	9.6 ± 3.8	9.8 ± 3.3	10.1 ± 4.3	9.4 ± 2.3
Week3	10.0 ± 3.2	10.5 ± 3.3	9.5 ± 3.9	9.7 ± 2.7

© 2024 Walther S et al. *JAMA Psychiatry.*

Post-hoc tests of outcomes from baseline to week 3

eTable 5. Post-hoc-p-values of Time with SIDAK correction.				
	**1Hz**	**iTBS**	**sham**	**waiting**
**SRRS**	<.001***	<.001***	<.001***	.756
**$PANSS_{total}$**	.001***	<.001***	.006**	.005**
**$PANSS_{positive}$**	.003**	.001***	.116	.006**
**$PANSS_{negative}$**	.037*	.004**	.017*	.252
**$PANSS_{general}$**	.001***	<.001***	.010*	.004**
**$BNSS_{total}$**	.062	.006**	.015*	.904
**$BNSS_{asocial}$**	.295	.001***	.067	.048*
**$BFCRS_{total}$**	.058	.093	.001***	.083
**$BFCRS_{abnormal}$**	.117	.264	.005**	.304
**$BFCRS_{decreased}$**	.249	.047*	.017*	.023*
**GAF**	.010*	<.001***	<.001***	.012*
**UPSA**	.022*	.004**	.093	.050*
**IPAQ**	.921	.005**	.831	.108

eTable 6. Post-hoc-p-values the interaction between Time & Treatment arm.						
	**1Hz vs iTBS**	**1Hz vs. sham**	**1Hz vs. waiting**	**iTBS vs. sham**	**iTBS vs. waiting**	**sham vs. waiting**
**LSD correction**						
SRRS Week1	.071	.078	<.001***	.916	.025*	.033*
SRRS Week2	.106	.091	<.001***	.834	.001***	.001***
SRRS Week3	.072	.017*	<.001***	.448	<.001***	.005**
SRRS Follow up1	.966	.070	.389	.063	.347	.280
SRRS Follow up2	.982	.209	.748	.209	.755	.296
$BNSS_{anhedonia}$	.156	.509	.606	.050*	.053	.859
$BNSS_{distress}$	.531	.854	.230	.694	.063	.147
$BFCRS_{total}$	.231	.064	.005**	.440	.074	.331
$BFCRS_{abnormal}$	.261	.166	.064	.717	.407	.659
$BFCRS_{decreased}$	.623	.042*	<.001***	.109	.001***	.087
**SIDAK correction**						
SRRS Week1	.356	.386	.001***	1.000	.142	.182
SRRS Week2	.489	.437	<.001***	.489	.004**	.008**
SRRS Week3	.361	.100	<.001***	.972	.003**	. 031*
SRRS Follow up1	1.000	.354	.948	.323	.923	.861
SRRS Follow up2	1.000	.747	1.000	.756	1.000	.878
$BNSS_{anhedonia}$	.638	.986	.996	.266	.279	1.000
$BFCRS_{total}$	.794	.328	.030*	.969	.369	.911
$BFCRS_{decreased}$	.997	.228	.002**	.498	.005**	.421

© 2024 Walther S et al. *JAMA Psychiatry.*

Follow up data

LOCF n = 88

eTable 7. Follow-ups with LOCF and covariates.

Outcomes	Estimated Marginal Mean ± SE 1Hz	iTBS	sham	waiting	F-stats*	p	Eta
SRRS							
BL	24.2 ± 1.3	22.7 ± 1.3	24.8 ± 1.4	24.2 ± 1.3	Time:		
Week1	17.7 ± 1.2	20.7 ± 1.2	20.9 ± 1.2	-	$F_{(8, 632)}$ = 10.2	<.001	.114
Week2	15.8 ± 1.2	18.5 ± 1.2	18.9 ± 1.3	-	Time*Group:		
Week3	14.4 ± 1.2	17.5 ± 1.2	18.9 ± 1.3	23.8 ± 1.2	$F_{(24, 632)}$= 4.8	<.001	.154
Week4	-	-	-	21.8 ± 1.2			
Week5	-	-	-	19.7 ± 1.2			
Week6	-	-	-	17.9 ± 1.2			
Followup1	15.4 ± 1.3	15.3 ± 1.3	18.9 ± 1.3	17.0 ± 1.3			
Followup2	15.0 ± 1.4	15.1 ± 1.4	17.7 ± 1.4	15.7 ± 1.4			
$PANSS_{total}$							
BL	76.7 ± 4.0	81.6 ± 3.9	77.2 ± 4.1	84.6 ± 3.8	$F_{(4, 316)}$ = 12.0	<.001	.132
Week3	63.9 ± 4.1	66.1 ± 4.0	66.8 ± 4.2	74.5 ± 3.9	$F_{(12, 316)}$ = 2.8	.001	.095
Week6	-	-	-	63.5 ± 3.8			
Followup1	62.7 ± 3.9	63.6 ± 3.8	68.2 ± 4.0	60.0 ± 3.7			
Followup2	63.6 ± 4.0	62.9 ± 3.9	67.5 ± 4.0	59.6 ± 3.8			
$PANSS_{positive}$							
BL	15.4 ± 1.3	17.1 ± 1.2	14.4 ± 1.3	16.6 ± 1.2	$F_{(4, 316)}$ = 7.0	<.001	.081
Week3	12.1 ± 1.1	13.6 ± 1.0	12.6 ± 1.1	13.7 ± 1.0	$F_{(12, 316)}$ = 1.8	.054	.063
Week6	-	-	-	11.2 ± 0.9			
Followup1	12.2 ± 1.0	13.0 ± 1.0	12.2 ± 1.1	10.5 ± 1.0			
Followup2	12.1 ± 1.0	12.7 ± 1.0	12.7 ± 1.0	10.7 ± 1.0			
$PANSS_{negative}$							
BL	22.8 ± 1.4	23.2 ± 1.4	24.7 ± 1.4	24.6 ± 1.3	$F_{(4, 316)}$ = 4.9	<.001	.059
Week3	20.0 ± 1.5	19.4 ± 1.4	21.4 ± 1.5	23.1 ± 1.4	$F_{(12, 316)}$ = 1.9	.039	.066
Week6	-	-	-	20.6 ± 1.4			
Followup1	18.8 ± 1.5	19.7 ± 1.5	22.9 ± 1.5	19.5 ± 1.4			
Followup2	19.4 ± 1.6	19.1 ± 1.5	21.8 ± 1.6	18.2 ± 1.5			
$PANSS_{general}$							
BL	38.5 ± 2.1	41.2 ± 2.1	38.1 ± 2.2	43.4 ± 2.0	$F_{(4, 316)}$ = 12.6	<.001	.138
Week3	31.8 ± 2.1	33.1 ± 2.1	32.7 ± 2.2	37.8 ± 2.1	$F_{(12, 316)}$ = 2.7	.002	.092
Week6	-	-	-	31.7 ± 2.0			
Followup1	31.7 ± 2.0	30.9 ± 1.9	33.1 ± 2.0	30.0 ± 1.9			
Followup2	32.2 ± 2.0	31.1 ± 1.9	33.0 ± 2.0	30.7 ± 1.9			
$BNSS_{total}$							
BL	40.3 ± 2.9	38.2 ± 2.8	47.6 ± 3.0	43.2 ± 2.8	$F_{(4, 316)}$ = 4.0	.003	.049

Week3	35.5 ± 2.9	31.2 ± 2.8	41.1 ± 2.9	43.5 ± 2.8	$F_{(12, 316)}$ = 1.7	.070	.060
Week6	-	-	-	36.7 ± 2.9			
Followup1	35.3 ± 3.2	29.4 ± 3.1	41.0 ± 3.2	35.4 ± 3.0			
Followup2	34.9 ± 3.3	29.6 ± 3.2	39.1 ± 3.4	31.4 ± 3.2			
$BNSS_{Anhedonia}$							
BL	10.3 ± 1.1	9.2 ± 1.0	11.5 ± 1.1	8.9 ± 1.0	$F_{(4, 316)}$ = 2.0	.094	.025
Week3	8.8 ± 1.0	6.9 ± 1.0	9.8 ± 1.0	9.6 ± 0.9	$F_{(12, 316)}$ = 1.5	.116	.055
Week6	-	-	-	7.2 ± 1.0			
Followup1	8.8 ± 1.0	6.0 ± 1.0	10.2 ± 1.1	6.8 ± 1.0			
Followup2	8.7 ± 1.1	6.5 ± 1.0	9.7 ± 1.1	5.8 ± 1.0			
$BNSS_{Distress}$							
BL	2.9 ± 0.4	2.9 ± 0.4	3.3 ± 0.4	2.6 ± 0.4	$F_{(4, 316)}$ = 1.6	.185	.019
Week3	2.3 ± 0.4	2.0 ± 0.4	2.2 ± 0.4	2.9 ± 0.4	$F_{(12, 316)}$ = 1.6	.078	.059
Week6	-	-	-	2.2 ± 0.4			
Followup1	2.6 ± 0.4	2.5 ± 0.4	2.6 ± 0.4	2.3 ± 0.3			
Followup2	2.6 ± 0.4	2.6 ± 0.4	2.4 ± 0.4	1.9 ± 0.4			
$BNSS_{Asocial}$							
BL	6.8 ± 0.5	6.7 ± 0.5	7.2 ± 0.6	7.2 ± 0.5	$F_{(4, 316)}$ = 2.5	.042	.031
Week3	6.3 ± 0.6	4.7 ± 0.6	6.1 ± 0.6	6.1 ± 0.6	$F_{(12, 316)}$ = 2.0	.022	.071
Week6	-	-	-	4.4 ± 0.6			
Followup1	5.5 ± 0.7	4.6 ± 0.7	5.7 ± 0.7	4.4 ± 0.7			
Followup2	6.0 ± 0.7	4.7 ± 0.6	5.6 ± 0.7	3.9 ± 0.6			
$BNSS_{Avolition}$							
BL	6.6 ± 0.7	6.2 ± 0.7	7.1 ± 0.7	6.6 ± 0.7	$F_{(4, 316)}$ = 2.9	.021	.036
Week3	6.3 ± 0.6	5.2 ± 0.6	6.5 ± 0.6	6.7 ± 0.6	$F_{(12, 316)}$ = 2.1	.020	.072
Week6	-	-	-	4.4 ± 0.6			
Followup1	6.2 ± 0.7	4.3 ± 0.7	6.0 ± 0.7	4.2 ± 0.7			
Followup2	6.2 ± 0.7	4.2 ± 0.7	5.7 ± 0.7	3.5 ± 0.6			
$BNSS_{Affect}$							
BL	9.6 ± 0.8	9.6 ± 0.8	12.0 ± 0.8	11.9 ± 0.8	$F_{(4, 316)}$ = 3.2	.013	.039
Week3	8.5 ± 0.7	8.9 ± 0.7	10.7 ± 0.8	11.8 ± 0.7	$F_{(12, 316)}$ = 2.3	.009	.079
Week6	-	-	-	9.6 ± 0.8			
Followup1	8.8 ± 0.9	8.5 ± 0.8	10.9 ± 0.9	8.7 ± 0.8			
Followup2	8.2 ± 0.9	8.2 ± 0.9	10.4 ± 0.9	8.2 ± 0.9			
$BNSS_{Alogia}$							
BL	4.1 ± 0.7	3.5 ± 0.7	6.4 ± 0.7	6.0 ± 0.7	$F_{(4, 316)}$ = 2.3	.062	.028
Week3	3.3 ± 0.7	3.5 ± 0.6	5.7 ± 0.7	6.4 ± 0.6	$F_{(12, 316)}$ = 1.8	.072	.059
Week6	-	-	-	4.6 ± 0.7			
Followup1	3.6 ± 0.7	3.6 ± 0.7	5.3 ± 0.7	4.6 ± 0.7			
Followup2	3.4 ± 0.7	3.5 ± 0.7	5.1 ± 0.7	3.8 ± 0.7			
$BFCRS_{total}$							

© 2024 Walther S et al. *JAMA Psychiatry.*

BL	4.2 ± 0.8	5.4 ± 0.8	7.8 ± 0.8	4.7 ± 0.8	$F_{(4, 316)} = 9.4$	<.001	.107
Week3	2.6 ± 0.9	4.0 ± 0.8	4.9 ± 0.9	6.1 ± 0.8	$F_{(12, 316)} = 3.1$	<.001	.106
Week6	-	-	-	3.7 ± 0.8			
Followup1	2.5 ± 0.8	2.7 ± 0.7	4.6 ± 0.8	3.6 ± 0.7			
Followup2	3.1 ± 0.8	2.4 ± 0.8	4.6 ± 0.8	3.1 ± 0.7			
$BFCRS_{abnormal}$							
BL	3.1 ± 0.7	3.9 ± 0.7	5.5 ± 0.7	3.2 ± 0.7	$F_{(4, 316)} = 5.3$	<.001	.063
Week3	2.0 ± 0.7	3.1 ± 0.7	3.5 ± 0.7	3.9 ± 0.7	$F_{(12, 316)} = 2.0$	.022	.071
Week6	-	-	-	2.2 ± 0.6			
Followup1	1.9 ± 0.6	2.0 ± 0.6	3.5 ± 0.6	2.4 ± 0.6			
Followup2	2.5 ± 0.6	1.8 ± 0.6	3.6 ± 0.6	2.0 ± 0.6			
$BFCRS_{decreased}$							
BL	0.9 ± 0.3	1.4 ± 0.3	2.4 ± 0.3	1.3 ± 0.3	$F_{(4, 316)} = 6.6$	<.001	.077
Week3	0.5 ± 0.3	0.7 ± 0.3	1.4 ± 0.3	2.2 ± 0.3	$F_{(12, 316)} = 2.4$	.006	.083
Week6	-	-	-	±			
Followup1	0.6 ± 0.3	0.6 ± 0.3	1.1 ± 0.3	1.2 ± 0.3			
Followup2	0.5 ± 0.3	0.5 ± 0.3	1.0 ± 0.3	1.1 ± 0.3			
UPDRS							
BL	18.0 ± 2.5	21.7 ± 2.5	22.9 ± 2.6	20.8 ± 2.4	$F_{(4, 316)} = 3.0$	.020	.036
Week3	15.1 ± 1.9	16.4 ± 1.9	17.5 ± 2.0	18.5 ± 1.9	$F_{(12, 316)} = 1.3$	.209	.047
Week6	-	-	-	15.1 ± 1.9			
Followup1	15.5 ± 2.0	14.4 ± 2.0	20.4 ± 2.1	15.8 ± 1.9			
Followup2	15.2 ± 2.1	14.6 ± 2.0	18.9 ± 2.1	15.5 ± 2.0			
AIMS							
BL	1.1 ± 0.5	1.1 ± 0.5	1.1 ± 0.5	0.3 ± 0.5	$F_{(4, 316)} = 1.2$	.332	.014
Week3	0.8 ± 0.3	0.5 ± 0.3	0.9 ± 0.3	0.3 ± 0.3	$F_{(12, 316)} = 0.8$	.644	.030
Week6	-	-	-	0.1 ± 0.3			
Followup1	0.7 ± 0.4	0.7 ± 0.4	1.0 ± 0.4	0.1 ± 0.3			
Followup2	1.2 ± 0.4	0.7 ± 0.4	0.6 ± 0.4	0.3 ± 0.4			
GAF							
BL	44.7 ± 2.8	39.6 ± 2.7	38.4 ± 2.9	41.2 ± 2.7	$F_{(4, 316)} = 10.5$	<.001	.117
Week3	52.5 ± 3.3	50.0 ± 3.2	51.8 ± 3.3	48.5 ± 3.1	$F_{(12, 316)} = 1.5$	.131	.053
Week6	-	-	-	55.5 ± 3.3			
Followup1	56.4 ± 3.5	53.5 ± 3.4	49.0 ± 3.5	55.7 ± 3.3			
Followup2	55.8 ± 3.7	54.0 ± 3.6	51.1 ± 3.7	57.7 ± 3.5			
SOFAS							
BL	42.5 ± 2.8	41.3 ± 2.8	36.8 ± 2.9	41.1 ± 2.7	$F_{(4, 316)} = 2.6$	.038	.031
Week3	48.1 ± 3.1	50.3 ± 3.0	44.5 ± 3.2	47.5 ± 3.0	$F_{(12, 316)} = 1.2$	.261	.045
Week6	-	-	-	54.0 ± 3.1			
Followup1	53.1 ± 3.3	51.6 ± 3.2	42.2 ± 3.3	54.7 ± 3.1			
Followup2	53.2 ± 3.6	53.4 ± 3.5	45.7 ± 3.6	55.5 ± 3.4			

© 2024 Walther S et al. *JAMA Psychiatry.*

UPSA							
BL	71.5 ± 3.5	71.6 ± 3.3	74.2 ± 3.5	74.8 ± 3.3	$F_{(4, 308)} = 4.4$	.002	.054
Week3	77.6 ± 2.9	78.8 ± 2.8	78.7 ± 2.9	79.6 ± 2.7	$F_{(12, 308)} = 0.4$	.961	.016
Week6	-	-	-	81.5 ± 2.7			
Followup1	77.1 ± 2.9	78.8 ± 2.7	78.7 ± 2.9	81.8 ± 2.7			
Followup2	76.6 ± 2.8	81.6 ± 2.7	80.4 ± 2.9	84.3 ± 2.7			
IPAQ							
BL	849 ± 490	795 ± 478	2209 ± 499	1038 ± 470	$F_{(4, 316)} = 2.6$	.034	.032
Week3	907 ± 701	2473 ± 684	2080 ± 714	1957 ± 673	$F_{(12, 316)} = 1.3$	.240	.046
Week6	-	-	-	2148 ± 689			
Followup1	924 ± 495	1232 ± 483	2259 ± 504	1622 ± 475			
Followup2	1393 ± 509	1302 ± 497	2154 ± 519	1784 ± 489			
SNS							
BL	18.4 ± 1.9	20.9 ± 1.9	22.8 ± 1.9	19.7 ± 1.8	$F_{(4, 316)} = 1.4$	.249	.017
Week3	18.1 ± 2.1	18.0 ± 2.0	19.2 ± 2.1	18.2 ± 2.0	$F_{(12, 316)} = 1.6$	.093	.057
Week6	-	-	-	17.1 ± 2.1			
Followup1	18.4 ± 2.1	15.4 ± 2.1	18.5 ± 2.2	16.2 ± 2.1			
Followup2	18.8 ± 2.1	15.0 ± 2.1	18.7 ± 2.1	14.9 ± 2.0			
Actigraphy							
BL	13383±1110	14256±1088	11469±1099	11431±1016	$F_{(4, 296)} = 1.0$	.435	.013
Week3	14037±1400	17027±1372	12235±1387	12276±1282	$F_{(12, 296)} = 0.6$	.835	.024
Week6	-	-	-	13172±1449			
Followup1	14037±1472	14516±1443	12725±1458	13338±1347			
Followup2	14761±1451	15510±1423	12962±1437	13139±1329			
$CR_{dominant}$							
BL	10.7 ± 0.8	10.9 ± 0.8	12.3 ± 0.9	10.9 ± 0.8	$F_{(4, 316)} = 3.2$	.012	.040
Week3	11.0 ± 0.8	11.8 ± 0.8	12.9 ± 0.8	11.3 ± 0.7	$F_{(12, 316)} = 1.2$	.261	.045
Week6	-	-	-	11.9 ± 0.7			
Followup1	10.7 ± 0.8	11.9 ± 0.8	11.8 ± 0.8	11.3 ± 0.8			
Followup2	10.2 ± 0.8	12.2 ± 0.8	12.0 ± 0.8	11.5 ± 0.8			
$CR_{nondominant}$							
BL	9.4 ± 0.8	9.3 ± 0.8	10.7 ± 0.8	9.4 ± 0.7	$F_{(4, 316)} = 2.3$	.061	.028
Week3	9.9 ± 0.8	10.1 ± 0.8	9.9 ± 0.8	9.8 ± 0.7	$F_{(12, 316)} = 1.9$	.035	.068
Week6	-	-	-	10.4 ± 0.7			
Followup1	10.2 ± 0.8	10.8 ± 0.8	9.7 ± 0.9	10.0 ± 0.8			
Followup2	9.7 ± 0.9	11.3 ± 0.9	10.0 ± 0.9	10.0 ± 0.8			

© 2024 Walther S et al. *JAMA Psychiatry.*

Completer data

Completer week 3

eTable 8. Primary and secondary outcomes according to treatment arm of completers with covariates.							
	Estimated Marginal Mean ± SE						
Outcomes	1Hz	iTBS	sham	waiting	F-stats*	p	Eta
SRRS							
BL	23.8 ± 1.4	22.7 ± 1.4	24.2 ± 1.4	24.7 ± 1.2	Time:		
Week1	17.5 ± 1.3	20.6 ± 1.3	21.0 ± 1.3	-	$F_{(3, 195)}$ = 17.3	<.001	.210
Week2	15.2 ± 1.4	17.9 ± 1.4	18.3 ± 1.4	-	Time*Group:		
Week3	13.2 ± 1.4	16.2 ± 1.4	18.5 ± 1.4	24.2 ± 1.2	$F_{(9, 195)}$= 5.6	<.001	.204
$PANSS_{total}$							
BL	76.8 ± 4.7	80.2 ± 4.6	75.6 ± 4.6	86.1 ± 4.0	$F_{(1, 65)}$ = 22.4	<.001	.257
Week3	60.6 ± 4.4	62.2 ± 4.4	62.0 ± 4.3	75.7 ± 3.8	$F_{(3, 65)}$ = 0.6	.605	.028
$PANSS_{positive}$							
BL	15.7 ± 1.5	17.2 ± 1.5	15.0 ± 1.5	16.9 ± 1.3	$F_{(1, 65)}$ = 11.0	.002	.144
Week3	11.4 ± 1.5	13.0 ± 1.2	12.7 ± 1.2	13.8 ± 1.1	$F_{(3, 65)}$ = 0.4	.770	.017
$PANSS_{negative}$							
BL	22.6 ± 1.6	22.8 ± 1.6	23.6 ± 1.5	25.0 ± 1.4	$F_{(1, 65)}$ = 8.4	.005	.115
Week3	19.1 ± 1.5	18.4 ± 1.5	19.3 ± 1.5	23.7 ± 1.3	$F_{(3, 65)}$ = 1.0	.387	.045
$PANSS_{general}$							
BL	38.6 ± 2.5	40.2 ± 2.4	37.0 ± 2.4	44.2 ± 2.1	$F_{(1, 65)}$ = 22.6	<.001	.258
Week3	30.0 ± 2.3	30.8 ± 2.3	30.1 ± 2.3	38.2 ± 2.0	$F_{(3, 65)}$ = 0.4	.743	.019
$BNSS_{total}$							
BL	38.4 ± 3.4	37.7 ± 3.3	45.9 ± 3.3	43.5 ± 2.9	$F_{(1, 65)}$ = 6.6	.012	.092
Week3	32.4 ± 3.2	29.0 ± 3.2	38.3 ± 3.2	44.1 ± 2.8	$F_{(3, 65)}$ = 2.2	.097	.092
$BNSS_{Anhedonia}$							
BL	9.8 ± 1.3	8.9 ± 1.3	11.0 ± 1.2	8.8 ± 1.1	$F_{(1, 65)}$ = 2.4	.124	.036
Week3	8.2 ± 1.2	6.4 ± 1.1	8.8 ± 1.1	9.4 ± 1.0	$F_{(3, 65)}$ = 2.3	.083	.097
$BNSS_{Distress}$							
BL	2.8 ± 0.5	2.9 ± 0.4	3.4 ± 0.4	2.7 ± 0.4	$F_{(1, 65)}$ = 3.1	.085	.045
Week3	2.2 ± 0.4	1.7 ± 0.4	2.0 ± 0.4	3.1 ± 0.4	$F_{(3, 65)}$ = 3.3	.024	.134
$BNSS_{Asocial}$							
BL	6.6 ± 0.6	6.6 ± 0.6	7.3 ± 0.6	7.4 ± 0.5	$F_{(1, 65)}$ = 5.2	.026	.074
Week3	5.8 ± 0.7	4.3 ± 0.7	6.1 ± 0.7	6.3 ± 0.6	$F_{(3, 65)}$ = 0.9	.426	.042
$BNSS_{Avolition}$							
BL	6.6 ± 0.8	5.7 ± 0.8	6.9 ± 0.8	6.9 ± 0.7	$F_{(1, 65)}$ = 1.2	.275	.018
Week3	6.2 ± 0.7	4.4 ± 0.7	6.3 ± 0.6	7.0 ± 0.6	$F_{(3, 65)}$ = 0.5	.669	.024
$BNSS_{Affect}$							
BL	9.0 ± 1.0	9.8 ± 1.0	11.1 ± 1.0	11.9 ± 0.9	$F_{(1, 65)}$ = 3.0	.088	.044

© 2024 Walther S et al. *JAMA Psychiatry.*

Week3	7.6 ± 0.9	8.8 ± 0.8	9.6 ± 0.8	11.8 ± 0.7	$F_{(3, 65)} = 0.5$	.655	.024
$BNSS_{Alogia}$							
BL	3.5 ± 0.8	3.8 ± 0.8	6.2 ± 0.8	5.8 ± 0.7	$F_{(1, 65)} = 2.3$	.135	.034
Week3	2.5 ± 0.7	3.3 ± 0.7	5.5 ± 0.7	6.4 ± 0.6	$F_{(3, 65)} = 0.8$	.505	.035
$BFCRS_{total}$							
BL	4.5 ± 0.9	5.6 ± 0.9	7.5 ± 0.9	4.3 ± 0.8	$F_{(1, 65)} = 11.3$	.001	.148
Week3	2.6 ± 1.0	4.1 ± 1.0	3.9 ± 1.0	5.7 ± 0.9	$F_{(3, 65)} = 5.0$	.004	.187
$BFCRS_{abnormal}$							
BL	3.5 ± 0.8	3.9 ± 0.8	5.1 ± 0.8	3.0 ± 0.7	$F_{(1, 65)} = 5.7$	.020	.081
Week3	2.3 ± 0.8	3.3 ± 0.8	2.6 ± 0.8	3.5 ± 0.7	$F_{(3, 65)} = 2.7$	.051	.112
$BFCRS_{decreased}$							
BL	0.9 ± 0.4	1.5 ± 0.4	2.4 ± 0.4	1.3 ± 0.3	$F_{(1, 65)} = 9.8$	.003	.131
Week3	0.2 ± 0.4	0.6 ± 0.4	1.3 ± 0.4	2.2 ± 0.3	$F_{(3, 65)} = 5.1$	.003	.191
UPDRS							
BL	17.7 ± 3.1	20.0 ± 3.0	23.3 ± 3.0	21.8 ± 2.6	$F_{(1, 65)} = 3.7$	.058	.054
Week3	13.9 ± 2.3	15.4 ± 2.2	16.1 ± 2.2	18.7 ± 1.9	$F_{(3, 65)} = 0.6$	.610	.027
AIMS							
BL	1.3 ± 0.6	0.8 ± 0.6	1.2 ± 0.6	0.5 ± 0.5	$F_{(1, 65)} = 0.6$	.423	.010
Week3	0.9 ± 0.4	0.3 ± 0.4	1.0 ± 0.4	0.4 ± 0.3	$F_{(3, 65)} = 0.2$	.883	.010
GAF							
BL	44.4 ± 3.4	39.7 ± 3.3	39.1 ± 3.3	41.7 ± 2.9	$F_{(1, 65)} = 12.4$	<.001	.161
Week3	53.0 ± 3.8	51.9 ± 3.7	55.9 ± 3.7	50.1 ± 3.3	$F_{(3, 65)} = 1.4$	.240	.062
SOFAS							
BL	42.9 ± 3.4	42.5 ± 3.4	36.8 ± 3.4	41.2 ± 3.0	$F_{(1, 65)} = 1.9$	.169	.029
Week3	48.8 ± 3.7	52.4 ± 3.6	47.1 ± 3.6	48.9 ± 3.2	$F_{(3, 65)} = 0.3$	.819	.014
UPSA							
BL	71.4 ± 4.2	70.3 ± 4.1	72.7 ± 4.0	74.1 ± 3.6	$F_{(1, 62)} = 6.6$	.012	.096
Week3	78.6 ± 3.4	79.0 ± 3.4	78.1 ± 3.3	80.1 ± 2.9	$F_{(3, 62)} = 0.2$	.908	.009
IPAQ							
BL	847 ± 479	761 ± 472	1687 ± 470	950 ± 413	$F_{(1, 65)} = 6.0$	.017	.084
Week3	1216 ± 781	2795 ± 769	1408 ± 766	1776 ± 673	$F_{(3, 65)} = 1.7$	.176	.073
SNS							
BL	17.4 ± 2.2	20.6 ± 2.1	22.6 ± 2.1	19.9 ± 1.9	$F_{(1, 65)} = 0.7$	.411	.010
Week3	17.6 ± 2.4	18.6 ± 2.3	17.8 ± 2.3	18.1 ± 2.1	$F_{(3, 65)} = 1.3$	.287	.056
Actigraphy							
BL	13912±1213	13226±1217	12341±1172	11287±1044	$F_{(1, 62)} = 0.8$	.360	.014
Week3	15028±1594	17170±1598	12880±1540	12091±1372	$F_{(3, 62)} = 1.0$	.408	.045
$CR_{dominant}$							
BL	10.4 ± 1.0	11.5 ± 1.0	11.8 ± 1.0	10.5 ± 0.9	$F_{(1, 62)} = 2.3$	.137	.035
Week3	10.7 ± 0.9	12.2 ± 1.0	12.7 ± 0.9	11.1 ± 0.8	$F_{(3, 62)} = 0.1$	.932	.007
$CR_{nondominant}$							

© 2024 Walther S et al. *JAMA Psychiatry.*

BL	8.7 ± 0.9	9.5 ± 0.9	10.6 ± 0.9	9.1 ± 0.8	$F_{(1, 63)}$ = 0.1	.813	.001
Week3	9.5 ± 0.9	10.4 ± 0.9	9.7 ± 0.9	9.5 ± 0.8	$F_{(3, 63)}$ = 1.5	.216	.068

© 2024 Walther S et al. *JAMA Psychiatry.*

eTable 9. Primary and secondary outcomes according to treatment arm with completers (raw).				
	Mean ± SD			
Outcomes	1Hz	iTBS	sham	waiting
SRRS				
BL	23.8 ± 6.2	24.5 ± 7.2	23.3 ± 4.9	23.9 ± 5.0
Week1	17.5 ± 5.7	22.5 ± 7.5	20.3 ± 4.1	-
Week2	15.2 ± 5.2	19.2 ± 8.0	18.0 ± 4.6	-
Week3	14.3 ± 3.5	18.2 ± 8.5	17.3 ± 3.4	22.7 ± 6.6
$PANSS_{total}$				
BL	75.8 ± 17.2	82.5 ± 22.0	75.3 ± 13.2	85.2 ± 18.1
Week3	62.6 ± 12.1	65.8 ± 23.2	60.1 ± 11.7	72.8 ± 18.3
$PANSS_{positive}$				
BL	15.2 ± 5.6	17.1 ± 7.0	15.5 ± 4.1	16.9 ± 5.9
Week3	11.4 ± 3.2	13.2 ± 6.1	12.9 ± 4.4	13.4 ± 4.5
$PANSS_{negative}$				
BL	22.4 ± 5.5	23.5 ± 6.7	23.3 ± 5.6	24.8 ± 6.0
Week3	19.8 ± 4.6	19.9 ± 7.4	18.2 ± 3.6	22.9 ± 6.6
$PANSS_{general}$				
BL	38.2 ± 9.8	41.9 ± 12.2	36.4 ± 6.9	43.5 ± 9.1
Week3	31.4 ± 6.7	32.7 ± 12.0	29.0 ± 6.8	36.5 ± 9.4
$BNSS_{total}$				
BL	39.6 ± 15.6	40.5 ± 13.0	43.4 ± 10.3	42.4 ± 12.6
Week3	35.4 ± 10.6	32.8 ± 16.7	34.8 ± 8.4	41.7 ± 13.8
$BNSS_{Anhedonia}$				
BL	10.6 ± 4.9	9.9 ± 4.7	9.9 ± 4.2	8.3 ± 5.2
Week3	9.0 ± 5.0	7.3 ± 4.5	7.8 ± 3.8	8.9 ± 4.5
$BNSS_{Distress}$				
BL	2.9 ± 1.9	3.2 ± 1.7	3.1 ± 1.3	2.6 ± 1.7
Week3	2.3 ± 1.6	2.0 ± 1.7	1.8 ± 1.3	3.0 ± 1.6
$BNSS_{Asocial}$				
BL	6.8 ± 2.2	6.8 ± 2.8	7.3 ± 2.4	7.2 ± 1.9
Week3	6.3 ± 2.7	4.8 ± 2.9	5.7 ± 2.3	5.9 ± 2.9
$BNSS_{Avolition}$				
BL	6.9 ± 3.0	6.3 ± 3.3	6.4 ± 3.0	6.6 ± 2.9
Week3	6.8 ± 1.6	5.2 ± 3.4	5.6 ± 2.3	6.6 ± 2.7
$BNSS_{Affect}$				
BL	9.0 ± 4.1	10.1 ± 4.0	10.9 ± 3.2	11.8 ± 3.9
Week3	8.0 ± 2.8	9.3 ± 4.2	9.3 ± 2.7	11.4 ± 3.2
$BNSS_{Alogia}$				
BL	3.4 ± 3.2	4.2 ± 3.5	5.7 ± 2.8	5.9 ± 3.2
Week3	3.0 ± 2.8	4.2 ± 3.3	4.6 ± 2.8	6.0 ± 2.9

© 2024 Walther S et al. *JAMA Psychiatry.*

$BFCRS_{total}$				
BL	4.5 ± 3.2	6.4 ± 5.2	6.8 ± 3.1	4.2 ± 3.0
Week3	2.7 ± 2.5	4.4 ± 5.0	3.8 ± 3.3	5.4 ± 3.6
$BFCRS_{abnormal}$				
BL	3.4 ± 2.3	4.4 ± 4.3	4.8 ± 2.4	2.9 ± 2.9
Week3	2.3 ± 1.9	3.3 ± 4.3	2.8 ± 2.5	3.4 ± 2.7
$BFCRS_{decreased}$				
BL	0.9 ± 1.7	1.8 ± 1.5	2.1 ± 1.6	1.3 ± 1.2
Week3	0.4 ± 1.3	0.9 ± 1.1	1.0 ± 1.3	2.0 ± 1.9
UPDRS				
BL	18.3 ± 9.7	21.1 ± 14.9	23.6 ± 11.8	20.1 ± 9.9
Week3	13.5 ± 7.4	14.5 ± 10.3	18.1 ± 6.7	18.0 ± 9.6
AIMS				
BL	1.8 ± 3.6	1.1 ± 2.1	0.9 ± 2.3	0.1 ± 0.4
Week3	1.1 ± 2.0	0.4 ± 1.2	0.8 ± 1.6	0.3 ± 0.8
GAF				
BL	44.1 ± 12.4	38.8 ± 14.0	39.2 ± 10.6	42.6 ± 12.8
Week3	54.1 ± 11.0	52.6 ± 18.7	53.7 ± 11.7	50.7 ± 14.5
SOFAS				
BL	41.6 ± 16.6	39.9 ± 13.1	38.7 ± 11.3	42.8 ± 11.7
Week3	48.6 ± 12.5	51.6 ± 16.8	47.3 ± 8.8	49.6 ± 15.4
UPSA				
BL	71.1 ± 15.1	68.1 ± 18.8	73.7 ± 14.6	74.7 ± 12.4
Week3	79.2 ± 12.5	77.6 ± 15.0	78.4 ± 12.4	80.5 ± 10.8
IPAQ				
BL	887 ± 1239	675 ± 958	1776 ± 2847	911 ± 1325
Week3	1001 ± 1645	2500 ± 4446	1687 ± 2842	1939 ± 2010
SNS				
BL	18.3 ± 9.6	21.9 ± 7.7	20.9 ± 7.6	19.5 ± 8.0
Week3	17.6 ± 10.4	19.2 ± 9.4	17.1 ± 8.2	18.2 ± 8.1
Actigraphy				
BL	13727 ± 4922	12732 ± 4541	12520 ± 3277	11675 ± 4715
Week3	14473 ± 5442	15857 ± 8597	13828 ± 4561	12722 ± 4143
$CR_{dominant}$				
BL	10.6 ± 3.3	11.2 ± 3.4	11.8 ± 4.3	10.5 ± 3.4
Week3	11.2 ± 2.7	12.5 ± 2.5	12.2 ± 4.2	11.0 ± 3.7
$CR_{nondominant}$				
BL	9.3 ± 4.3	10.0 ± 3.3	9.8 ± 4.4	9.1 ± 2.2
Week3	9.8 ± 3.6	10.7 ± 3.4	9.2 ± 3.9	9.5 ± 2.7

© 2024 Walther S et al. *JAMA Psychiatry.*

Completer n = 32 at follow-up

eTable 10. Follow ups of completers with covariates.

Outcomes	Estimated Marginal Mean ± SE 1Hz	iTBS	sham	waiting	F-stats*	p	Eta
SRRS							
BL	21.7 ± 2.5	22.8 ± 2.8	24.7 ± 2.3	24.5 ± 1.8	Time:		
Week1	17.0 ± 2.7	19.1 ± 2.9	21.1 ± 2.5	-	$F_{(8, 184)}$ = 5.0	<.001	.178
Week2	16.6 ± 2.7	18.3 ± 3.0	18.7 ± 2.5	-	Time*Group:		
Week3	13.4 ± 2.5	12.4 ± 2.7	18.4 ± 2.3	26.0 ± 1.8	$F_{(24, 184)}$= 2.8	<.001	.270
Week4	-	-	-	20.8 ± 1.7			
Week5	-	-	-	18.5 ± 2.0			
Week6	-	-	-	16.9 ± 1.7			
Followup1	14.7 ± 2.5	10.2 ± 2.7	20.1 ± 2.3	14.9 ± 1.8			
Followup2	14.6 ± 2.8	9.6 ± 3.1	15.9 ± 2.6	12.0 ± 2.0			
$PANSS_{total}$							
BL	67.5 ± 7.9	77.3 ± 8.6	79.3 ± 7.3	93.5 ± 5.7	$F_{(4, 92)}$ = 5.1	<.001	.182
Week3	62.5 ± 7.0	51.5 ± 7.7	70.5 ± 6.5	81.1 ± 5.1	$F_{(12, 92)}$ = 3.2	.001	.293
Week6	-	-	-	62.6 ± 4.4			
Followup1	58.1 ± 5.0	49.1± 5.5	70.6 ± 4.7	56.7 ± 3.6			
Followup2	62.7 ± 7.0	47.7 ± 7.7	67.1 ± 6.5	55.4 ± 5.1			
$PANSS_{positive}$							
BL	12.6 ± 2.5	20.5 ± 2.7	14.0 ± 2.3	18.2 ± 1.8	$F_{(4, 92)}$ = 1.1	.383	.044
Week3	11.5 ± 1.9	10.6 ± 2.0	14.8 ± 1.7	15.2 ± 1.3	$F_{(12, 92)}$ = 3.1	.001	.287
Week6	-	-	-	10.9 ± 1.0			
Followup1	11.8 ± 1.6	11.2 ± 1.8	13.3 ± 1.5	9.8 ± 1.2			
Followup2	11.5 ± 1.7	10.4 ± 1.9	14.7 ± 1.6	10.1 ± 1.3			
$PANSS_{negative}$							
BL	22.2 ± 2.8	19.5 ± 3.0	25.1 ± 2.6	26.7 ± 2.0	$F_{(4, 92)}$ = 4.5	.002	.164
Week3	20.1 ± 2.8	16.2 ± 3.0	20.9 ± 2.5	24.9 ± 2.0	$F_{(12, 92)}$ = 1.5	.146	.162
Week6	-	-	-	20.8 ± 1.9			
Followup1	15.6 ± 2.5	14.7 ± 2.7	23.4 ± 2.3	19.1 ± 1.8			
Followup2	18.2 ± 2.7	13.0 ± 3.0	19.6 ± 2.5	16.4 ± 2.0			
$PANSS_{general}$							
BL	32.7 ± 3.6	37.3 ± 3.9	40.3 ± 3.3	48.5 ± 2.6	$F_{(4, 92)}$ = 6.4	<.001	.217
Week3	30.8 ± 3.5	24.7 ± 3.8	34.8 ± 3.2	41.0 ± 2.5	$F_{(12, 92)}$ = 3.7	<.001	.327
Week6	-	-	-	30.9 ± 2.1			
Followup1	30.7 ± 2.6	23.3 ± 2.8	33.8 ± 2.4	27.7 ± 1.9			
Followup2	33.0 ± 3.4	24.3 ± 3.7	32.8 ± 3.1	28.9 ± 2.4			
$BNSS_{total}$							
BL	34.9 ± 6.5	32.4 ± 7.1	50.2 ± 6.0	44.3 ± 4.7	$F_{(4, 92)}$ = 4.7	.002	.170
Week3	34.1 ± 6.1	26.4 ± 6.7	40.9 ± 5.7	44.0 ± 4.4	$F_{(12, 92)}$ = 1.4	.166	.157

© 2024 Walther S et al. *JAMA Psychiatry.*

Week6	-	-	-	35.7 ± 4.3			
Followup1	30.3 ± 5.6	16.1 ± 6.1	43.6 ± 5.1	34.0 ± 4.0			
Followup2	30.5 ± 6.4	18.3 ± 7.0	37.2 ± 5.9	25.4 ± 4.6			
$BNSS_{Anhedonia}$							
BL	8.7 ± 2.3	9.0 ± 2.5	13.5 ± 2.1	8.9 ± 1.6	$F_{(4, 92)} = 4.1$	.004	.151
Week3	7.7 ± 2.3	7.7 ± 2.5	10.6 ± 2.1	9.0 ± 1.6	$F_{(12, 92)} = 1.0$	.452	.116
Week6	-	-	-	7.5 ± 1.5			
Followup1	7.1 ± 1.9	2.5 ± 2.1	11.3 ± 1.8	7.5 ± 1.4			
Followup2	7.4 ± 2.2	4.4 ± 2.4	9.5 ± 2.0	5.2 ± 1.6			
$BNSS_{Distress}$							
BL	2.9 ± 0.8	2.7 ± 0.9	3.5 ± 0.7	2.7 ± 0.6	$F_{(4, 92)} = 0.5$	.709	.023
Week3	1.8 ± 0.9	2.3 ± 0.9	2.3 ± 0.8	2.8 ± 0.6	$F_{(12, 92)} = 0.4$	.940	.055
Week6	-	-	-	2.3 ± 0.6			
Followup1	1.8 ± 0.5	1.6 ± 0.6	2.9 ± 0.5	2.7 ± 0.4			
Followup2	1.9 ± 0.8	2.2 ± 0.8	2.3 ± 0.7	1.8 ± 0.5			
$BNSS_{Asocial}$							
BL	6.4 ± 1.0	5.9 ± 1.1	8.9 ± 1.0	8.1 ± 0.7	$F_{(4, 92)} = 3.6$	.009	.135
Week3	5.8 ± 1.2	4.1 ± 1.3	6.9 ± 1.1	6.7 ± 0.9	$F_{(12, 92)} = 1.3$	.234	.145
Week6	-	-	-	5.0 ± 0.9			
Followup1	4.3 ± 1.3	3.2 ± 1.4	5.5 ± 1.2	5.3 ± 0.9			
Followup2	6.2 ± 1.3	3.7 ± 1.5	5.3 ± 1.2	4.0 ± 1.0			
$BNSS_{Avolition}$							
BL	5.9 ± 1.3	5.8 ± 1.4	7.4 ± 1.2	7.6 ± 0.9	$F_{(4, 92)} = 3.2$	.015	.124
Week3	6.3 ± 1.0	2.7 ± 1.1	6.7 ± 1.0	7.1 ± 0.8	$F_{(12, 92)} = 1.4$	.192	.152
Week6	-	-	-	5.5 ± 0.8			
Followup1	5.0 ± 1.2	0.5 ± 1.3	6.9 ± 1.1	5.0 ± 0.8			
Followup2	5.0 ± 1.2	1.3 ± 1.3	5.8 ± 1.1	3.2 ± 0.8			
$BNSS_{Affect}$							
BL	7.8 ± 1.9	6.5 ± 2.0	11.1 ± 1.7	11.6 ± 1.3	$F_{(4, 92)} = 1.5$	.214	.060
Week3	8.9 ± 1.3	6.5 ± 1.4	9.4 ± 1.2	12.4 ± 0.9	$F_{(12, 92)} = 1.5$	.150	.161
Week6	-	-	-	11.5 ± 1.0			
Followup1	8.8 ± 1.5	6.4 ± 1.6	11.9 ± 1.4	9.5 ± 1.1			
Followup2	7.2 ± 1.7	5.5 ± 1.9	9.9 ± 1.6	8.6 ± 1.3			
$BNSS_{Alogia}$							
BL	3.2 ± 1.6	2.6 ± 1.7	5.7 ± 1.4	5.4 ± 1.1	$F_{(4, 92)} = 2.1$	.085	.084
Week3	3.6 ± 1.3	3.1 ± 1.5	5.1 ± 1.3	5.9 ± 1.0	$F_{(12, 92)} = 0.5$	.897	.063
Week6	-	-	-	3.9 ± 0.9			
Followup1	3.3 ± 1.4	1.9 ± 1.6	5.2 ± 1.3	4.0 ± 1.0			
Followup2	2.8 ± 1.1	1.2 ± 1.2	4.5 ± 1.0	2.6 ± 0.8			
$BFCRS_{total}$							
BL	4.1 ± 1.6	3.6 ± 1.7	8.2 ± 1.5	5.1 ± 1.1	$F_{(4, 92)} = 5.6$	<.001	.195

© 2024 Walther S et al. *JAMA Psychiatry.*

Week3	3.3 ± 1.4	1.3 ± 1.6	3.1 ± 1.3	6.4 ± 1.0	$F_{(12, 92)} = 2.2$	.019	.221
Week6	-	-	-	2.7 ± 0.9			
Followup1	2.0 ± 1.2	1.7 ± 1.3	3.6 ± 1.1	3.2 ± 0.9			
Followup2	4.5 ± 1.2	0.5 ± 1.3	3.2 ± 1.1	2.1 ± 0.9			
$BFCRS_{abnormal}$							
BL	4.1 ± 1.3	1.8 ± 1.5	5.6 ± 1.2	3.6 ± 1.0	$F_{(4, 92)} = 2.6$	.043	.101
Week3	2.8 ± 1.1	1.5 ± 1.2	1.8 ± 1.0	3.5 ± 0.8	$F_{(12, 92)} = 1.2$	.268	.139
Week6	-	-	-	1.6 ± 0.6			
Followup1	1.7 ± 1.1	1.1 ± 1.2	2.8 ± 1.0	2.4 ± 0.8			
Followup2	4.0 ± 1.1	0.4 ± 1.2	2.7 ± 1.0	1.6 ± 0.8			
$BFCRS_{decreased}$							
BL	0.1 ± 0.6	1.3 ± 0.7	2.7 ± 0.6	1.3 ± 0.4	$F_{(4, 92)} = 5.4$	<.001	.189
Week3	0.5 ± 0.6	-0.3 ± 0.7	1.3 ± 0.6	2.8 ± 0.5	$F_{(12, 92)} = 2.2$	.016	.226
Week6	-	-	-	1.1 ± 0.5			
Followup1	0.2 ± 0.4	0.6 ± 0.5	0.8 ± 0.4	0.8 ± 0.3			
Followup2	0.2 ± 0.3	0.1 ± 0.3	0.5 ± 0.3	0.5 ± 0.2			
UPDRS							
BL	18.1 ± 4.8	16.9 ± 5.3	28.4 ± 4.5	24.3 ± 3.5	$F_{(4, 92)} = 1.9$	.110	.078
Week3	13.6 ± 3.5	13.1 ± 3.8	14.6 ± 3.2	19.8 ± 2.5	$F_{(12, 92)} = 1.4$	.171	.156
Week6	-	-	-	14.1 ± 2.4			
Followup1	17.3 ± 3.5	7.0 ± 3.8	21.7 ± 3.2	15.1 ± 2.5			
Followup2	16.5 ± 3.8	9.2 ± 4.2	16.6 ± 3.5	14.2 ± 2.7			
AIMS							
BL	2.9 ± 1.2	0.1 ± 1.3	1.6 ± 1.1	0.5 ± 0.9	$F_{(4, 92)} = 0.6$	.659	.026
Week3	1.6 ± 0.8	-0.4 ± 0.9	1.6 ± 0.7	0.8 ± 0.6	$F_{(12, 92)} = 1.0$	.614	.120
Week6	-	-	-	0.1 ± 0.5			
Followup1	1.4 ± 1.0	-0.2 ± 1.1	1.7 ± 0.9	0.2 ± 0.7			
Followup2	3.1 ± 1.2	-0.2 ± 1.3	0.5 ± 1.1	0.6 ± 0.8			
GAF							
BL	49.0 ± 5.9	48.0 ± 6.4	29.6 ± 5.4	38.0 ± 4.2	$F_{(4, 92)} = 6.9$	<.001	.231
Week3	51.5 ± 6.1	63.5 ± 6.6	54.3 ± 5.6	47.0 ± 4.4	$F_{(12, 92)} = 1.9$	.044	.199
Week6	-	-	-	57.3 ± 4.9			
Followup1	62.4 ± 6.7	69.3 ± 7.3	43.0 ± 6.2	56.1 ± 4.8			
Followup2	59.7 ± 8.1	67.8 ± 8.9	50.7 ± 7.5	61.2 ± 5.9			
SOFAS							
BL	48.4 ± 6.5	52.2 ± 7.1	25.0 ± 6.0	40.1 ± 4.7	$F_{(4, 92)} = 4.5$	.002	.163
Week3	50.6 ± 6.1	63.7 ± 6.7	43.7 ± 5.7	48.4 ± 4.4	$F_{(12, 92)} = 1.0$	.436	.118
Week6	-	-	-	-			
Followup1	59.9 ± 6.6	67.3 ± 7.2	36.1 ± 6.1	57.6 ± 4.7			
Followup2	57.7 ± 8.2	71.8 ± 9.0	47.4 ± 7.6	60.6 ± 6.0			
UPSA							

© 2024 Walther S et al. *JAMA Psychiatry.*

BL	70.0 ± 7.0	83.6 ± 8.5	73.6 ± 6.3	73.2 ± 5.0	$F_{(4, 88)}$ = 2.6	.042	.105
Week3	73.5 ± 5.3	92.0 ± 6.3	77.9 ± 4.7	80.5 ± 3.7	$F_{(12, 88)}$ = 0.3	.984	.042
Week6	-	-	-	83.3 ± 3.6			
Followup1	76.4 ± 5.5	90.6 ± 6.6	78.1 ± 4.9	82.0 ± 3.9			
Followup2	76.0 ± 4.8	91.4 ± 5.8	83.9 ± 4.3	87.4 ± 3.4			
IPAQ							
BL	663 ± 1122	1656 ± 1231	2466 ± 1040	1442 ± 812	$F_{(4, 92)}$ = 0.2	.950	.008
Week3	681 ± 1015	1968 ± 1113	1502 ± 940	2475 ± 734	$F_{(12, 92)}$ = 1.0	.460	.115
Week6	-	-	-	2257 ± 701			
Followup1	863 ± 839	1669 ± 920	1041 ± 777	1484 ± 607			
Followup2	2166 ± 839	1896 ± 920	768 ± 777	1921 ± 607			
SNS							
BL	16.9 ± 3.6	20.7 ± 4.0	26.5 ± 3.4	±	$F_{(4, 92)}$ = 2.7	.036	.105
Week3	21.5 ± 4.2	14.8 ± 4.6	20.3 ± 3.9	±	$F_{(12, 92)}$ = 1.3	.215	.148
Week6	-	-	-	±			
Followup1	19.3 ± 4.4	14.1 ± 4.8	17.4 ± 4.1	±			
Followup2	21.0 ± 4.1	12.2 ± 4.5	18.3 ± 3.8	±			
Actigraphy							
BL	16940±1904	13223±1756	12180±1772	10501±1259	$F_{(4, 72)}$ = 0.2	.940	.011
Week3	15460±2608	16570±2406	14724±2427	11743±1725	$F_{(12, 72)}$ = 0.7	.769	.101
Week6	-	-	-	11958±2045			
Followup1	13750±3004	12059±2771	15644±2795	14121±1986			
Followup2	16504±2918	16121±2691	15398±2715	13987±1929			
$CR_{dominant}$							
BL	10.5 ± 1.4	12.5 ± 1.6	13.8 ± 1.3	10.0 ± 1.0	$F_{(4, 92)}$ = 2.6	.041	.102
Week3	11.3 ± 1.5	13.5 ± 1.6	13.0 ± 1.4	10.9 ± 1.1	$F_{(12, 92)}$ = 1.7	.090	.178
Week6	-	-	-	11.1 ± 1.0			
Followup1	12.2 ± 1.3	12.9 ± 1.4	11.7 ± 1.2	10.1 ± 0.9			
Followup2	9.7 ± 1.2	14.6 ± 1.3	12.0 ± 1.1	10.8 ± 0.9			
$CR_{nondominant}$							
BL	7.2 ± 1.3	12.1 ± 1.4	11.9 ± 1.2	8.8 ± 0.9	$F_{(4, 92)}$ = 1.1	.366	.045
Week3	8.3 ± 1.5	12.3 ± 1.7	10.1 ± 1.4	9.7 ± 1.1	$F_{(12, 92)}$ = 1.6	.118	.169
Week6	-	-	-	9.6 ± 1.1			
Followup1	9.3 ± 1.6	12.7 ± 1.7	10.3 ± 1.5	9.3 ± 1.2			
Followup2	7.4 ± 1.5	15.1 ± 1.7	11.1 ± 1.4	9.4 ± 1.1			

© 2024 Walther S et al. *JAMA Psychiatry.*

eTable 11. Side-effects in the waiting group

	**Waiting period**<br>n=22	**1Hz over SMA post waiting period**<br>n=16
No side-effects, n (%)	-	4 (25%)
Dizziness, n (%)	-	< 10 (62.5%)*
Headache and neck pain, n (%)	-	7 (44%)
Fatigue, n (%)	-	8 (50%)
Other, n (%)	-	< 10 (62.5%)*<br>- Perceived balance impairment<br>- Tinnitus<br>- Dry mouth

Note: * There were too few patients to provide an exact number without compromising data identifiability requirements.

© 2024 Walther S et al. *JAMA Psychiatry.*

eFigure 1. Course of anhedonia.

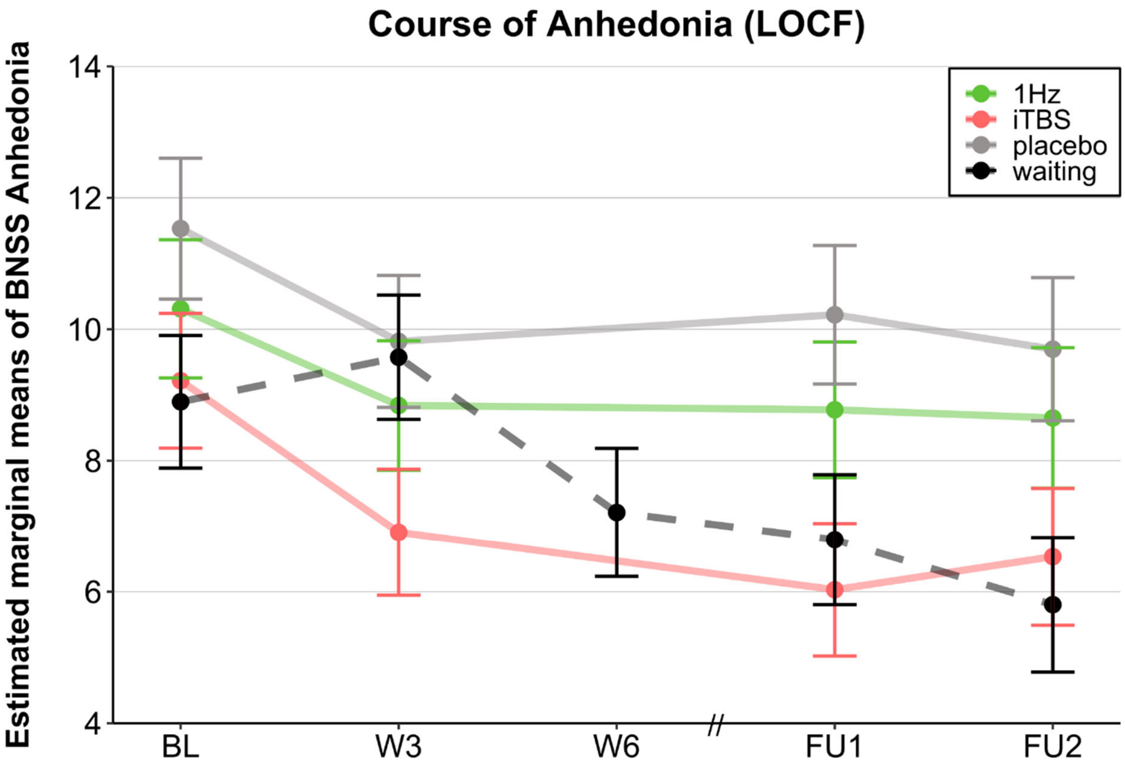

© 2024 Walther S et al. *JAMA Psychiatry.*

eFigure 2. Course of global functioning.

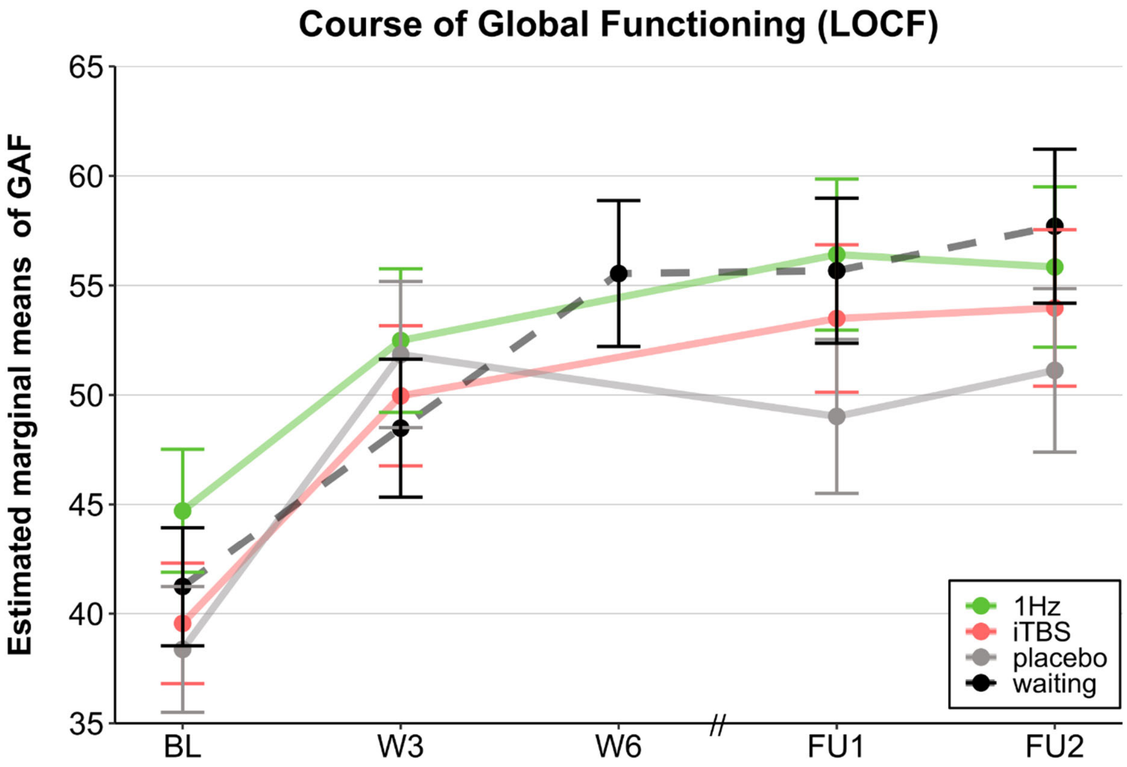

© 2024 Walther S et al. *JAMA Psychiatry.*

eTable 12. Baseline variables in the three sample versions.

Variable	ITT (≥1 stimulations, n = 88)				With baseline (n = 97)				With consent (n = 103)			
	1Hz	iTBS	sham	wait	1Hz	iTBS	sham	wait	1Hz	iTBS	Sham	wait
N	22	22	22	22	25	23	27	22	26	25	28	24
% male	72.7	54.5	40.9	36.4	72.0	56.5	40.7	36.4	69.2	60.0	39.3	37.5
Age in y	39.5	33.5	38.2	33.8	40.1	34.5	37.3	33.8	40.0	34.6	37.9	33.5
SRRS bl	24.7	23.3	24.2	23.7	24.8	24.1	23.7	23.7	No data without baseline			
blOLZ	13.3	13.4	22.6	15.8	13.1	13.9	21.9	15.8	No data without baseline			
blDE	6.1	5.1	.5	.5	5.4	4.9	1.2	.5	No data without baseline			
mOLZ	14.5	12.0	22.9	15.4	14.2	12.6	22.2	15.4	No data without baseline			
mDE	3.6	2.3	.7	.1	3.2	2.2	1.4	.1	No data without baseline			

Note: SRRS bl: baseline value of the Salpetriere Retardation Rating Scale score; blOLZ: baseline olanzapine equivalents in mg; blDE: baseline diazepam equivalents in mg; mOLZ: mean olanzapine equivalents in mg; mDE: mean diazepam equivalents in mg.

© 2024 Walther S et al. *JAMA Psychiatry.*

**eTable 13. Results of the primary outcomes in n=88 (ITT) vs. n=97 (with baseline), covaried for sex, baseline and treatment medication.**				
			**n = 88**	**n = 97**
**Response rates in %**		1 Hz	68.2	60.0
		iTBS	36.4	34.8
		sham	31.8	25.9
		waiting	18.2	18.2
**Chi²-test, df=3**		X^2	12.11	10.09
		p-value	.007	.017
**Logistic regression adjusted for sex, baseline and treatment medication**		Wald X^2	13.55	10.79
		p-value	.004	.013
		p-value OR 1Hz vs. iTBS	.005	.04
		p-value OR 1Hz vs. Sham	.01	.03
		p-value OR 1 Hz vs. waiting	<.001	.002
**ANCOVA SRRS scores at baseline and week 3** df=3		F (time x treatment arm interaction)	8.5	6.05
		p-value	<.001	<.001
**Post-hoc tests**	SRRS Week3	1Hz vs iTBS	.072	.179
		1Hz vs. sham	.017*	.192
		1Hz vs. waiting	<.001***	<.001***
		iTBS vs. sham	.448	.982
		iTBS vs. waiting	<.001***	.017*
		sham vs. waiting	.005**	.014*
Note: ITT n = 88 includes patients receiving ≥ 1 stimulations (ITT). n = 97 includes all patients with baseline assessments. All missing data were computed with last observation carried forward (LOCF).				

© 2024 Walther S et al. *JAMA Psychiatry.*

References

1. Kay SR, Fiszbein A, Opler LA. The positive and negative syndrome scale (PANSS) for schizophrenia. *Schizophr Bull*. 1987;13(2):261-76.
2. Kirkpatrick B, Strauss GP, Nguyen L, et al. The brief negative symptom scale: psychometric properties. *Schizophr Bull*. 2011;37(2):300-5.
3. Bush G, Fink M, Petrides G, Dowling F, Francis A. Catatonia. I. Rating scale and standardized examination. *Acta Psychiatr Scand*. 1996;93(2):129-36.
4. Walther S, Stegmayer K, Wilson JE, Heckers S. Structure and neural mechanisms of catatonia. *Lancet Psychiatry*. 2019;6(7):610-619.
5. Walther S, Ramseyer F, Horn H, Strik W, Tschacher W. Less structured movement patterns predict severity of positive syndrome, excitement, and disorganization. *Schizophr Bull*. 2014;40(3):585-91.
6. Middelkoop HA, van Dam EM, Smilde-van den Doel DA, Van Dijk G. 45-hour continuous quintuple-site actimetry: relations between trunk and limb movements and effects of circadian sleep-wake rhythmicity. *Psychophysiology*. 1997;34(2):199-203.
7. Walther S, Horn H, Razavi N, Koschorke P, Muller TJ, Strik W. Quantitative motor activity differentiates schizophrenia subtypes. *Neuropsychobiology*. 2009;60(2):80-6.
8. Walther S, Horn H, Razavi N, et al. Higher motor activity in schizophrenia patients treated with olanzapine versus risperidone. *J Clin Psychopharmacol*. 2010;30(2):181-4.
9. Walther S, Koschorke P, Horn H, Strik W. Objectively measured motor activity in schizophrenia challenges the validity of expert ratings. *Psychiatry Res*. 2009;169(3):187-90.
10. Walther S, Vladimirova I, Alexaki D, et al. Low physical activity is associated with two hypokinetic motor abnormalities in psychosis. *J Psychiatr Res*. 2022;146:258-263.
11. Gebhardt A, Vanbellingen T, Baronti F, Kersten B, Bohlhalter S. Poor dopaminergic response of impaired dexterity in Parkinson's disease: Bradykinesia or limb kinetic apraxia? *Mov Disord*. 2008;23(12):1701-6.

© 2024 Walther S et al. *JAMA Psychiatry.*

12. Walther S, Kunz M, Muller M, et al. Single Session Transcranial Magnetic Stimulation Ameliorates Hand Gesture Deficits in Schizophrenia. *Schizophr Bull*. 2020;46(2):286-293.
13. Walther S, Stegmayer K, Sulzbacher J, et al. Nonverbal social communication and gesture control in schizophrenia. *Schizophr Bull*. 2015;41(2):338-45.
14. Walther S, Vanbellingen T, Muri R, Strik W, Bohlhalter S. Impaired pantomime in schizophrenia: Association with frontal lobe function. *Cortex*. 2013;49(2):520-7.
15. Broglio K. Randomization in Clinical Trials: Permuted Blocks and Stratification. *JAMA*. 2018;319(21):2223-2224.

© 2024 Walther S et al. *JAMA Psychiatry.*
