# Supplementary material for: Psychomotor Slowing in Psychosis and Inhibitory Repetitive Transcranial Magnetic Stimulation: A Randomized Clinical Trial
Source: JAMA Psychiatry. 2024 Feb 28;81(6):563–71. doi: 10.1001/jamapsychiatry.2024.0026 (PMC10902782; doi:10.1001/jamapsychiatry.2024.0026)
Supplement: Supplement 3. — Data sharing statement [file jamapsychiatry-e240026-s003.pdf]

Data Sharing Statement

Data
Data available: No

Additional Information
Explanation for why data not available: participants have not provided consent to broad data sharing of their health related data
